# Host allometry influences the evolution of parasite host-generalism: theory and meta-analysis

**DOI:** 10.1098/rstb.2016.0089

**Published:** 2017-03-13

**Authors:** Josephine G. Walker, Amy Hurford, Jo Cable, Amy R. Ellison, Stephen J. Price, Clayton E. Cressler

**Affiliations:** 1 School of Biological Sciences, University of Bristol, Life Sciences Building, 24 Tyndall Avenue, Bristol BS8 1TQ, UK; 2 School of Social and Community Medicine, University of Bristol, Oakfield House, Oakfield Grove, Bristol BS8 2BN, UK; 3 Department of Biology, Memorial University of Newfoundland, St John's, Newfoundland, Canada A1B 3X9; 4 Department of Mathematics and Statistics, Memorial University of Newfoundland, St John's, Newfoundland, Canada A1C 5S7; 5 School of Biosciences, Cardiff University, Cardiff CF10 3AX, UK; 6 UCL Genetics Institute, Gower Street, London WC1E 6BT, UK; 7 School of Biological Sciences, University of Nebraska-Lincoln, 424 Manter Hall, 1104 T St, Lincoln, NE 68588-0118, USA

**Keywords:** fish parasites, transmission, host range, specialism, invasion analysis

## Abstract

Parasites vary widely in the diversity of hosts they infect: some parasite species are specialists—infecting just a single host species, while others are generalists, capable of infecting many. Understanding the factors that drive parasite host-generalism is of basic biological interest, but also directly relevant to predicting disease emergence in new host species, identifying parasites that are likely to have unidentified additional hosts, and assessing transmission risk. Here, we use mathematical models to investigate how variation in host body size and environmental temperature affect the evolution of parasite host-generalism. We predict that parasites are more likely to evolve a generalist strategy when hosts are large-bodied, when variation in host body size is large, and in cooler environments. We then explore these predictions using a newly updated database of over 20 000 fish–macroparasite associations. Within the database we see some evidence supporting these predictions, but also highlight mismatches between theory and data. By combining these two approaches, we establish a theoretical basis for interpreting empirical data on parasites' host specificity and identify key areas for future work that will help untangle the drivers of parasite host-generalism.

This article is part of the themed issue ‘Opening the black box: re-examining the ecology and evolution of parasite transmission’.

## 1. Introduction

The diversity of hosts infected by a parasite species is a key factor affecting transmission. Parasites that infect a diverse range of host species are more resilient to changing conditions, and reservoir hosts are often crucial for the maintenance of transmission [1,2]. The hosts a parasite infects are predicted to affect parasite virulence through mechanisms including relative host availability, maladaptive virulence and fitness costs associated with infecting novel hosts [3]. In addition, the ability of a parasite to infect multiple host species, particularly across taxonomic orders, is a risk factor for emerging infectious diseases (EIDs) of humans and livestock [4].

Despite evidence for the importance of a parasite's host diversity for transmission, it remains unclear whether most parasites are generalists (infecting more than one host species) or specialists (infecting one host species), and what factors might influence evolutionary switches between generalism and specialism. On one hand, EID studies, ranging from microbes to macroparasites, suggest that most parasites are generalists: 60% of human infectious diseases are zoonotic and 80% of pathogens of domestic animals infect multiple host species [4–6]. However, theory suggests that fitness trade-offs between host range and parasite performance on each host can lead to the evolution of host specialization [7], and such trade-offs are common (although not universal) for parasites [8–10]. Ecological specialization is often considered an ‘evolutionary dead-end’, such that specialist parasites have a reduced potential to adapt to novel hosts, and parasites that are highly host-specific will have higher extinction and lower speciation rates than generalist parasites [11]. However, empirical evidence suggests that evolutionary transitions between specialism and generalism are bidirectional [12,13]. Rather few studies have examined whether specialism or generalism is the ancestral state for macroparasites of animals; for both feather lice (arthropods) of doves and gill monogeneans (platyhelminthes) of African freshwater fish host generalism appears to have derived from ancestral specialism [14,15].

Attempts to understand the ecological drivers of the evolution of a parasite's host diversity have examined a number of parasite or host traits and environmental factors (table 1). In this study, we focus on understanding how host body size and environmental temperature affect host diversity. The relationship between host size and parasitism has been explored in depth, often with reference to island biogeography theory (IBT). IBT predicts that the number of parasite species infecting a host will increase with host body size, as larger-bodied hosts represent larger habitat patches with more niches [28–30]. Most work on this relationship, however, has been host-centric. Few studies have considered the question from the parasite perspective; that is, are parasites that infect large-bodied hosts also generalists infecting a wide range of hosts [24,25,27]? There are, however, several reasons to suspect that host body size might influence a parasite's host diversity. Larger hosts support higher within-host parasite abundances [31], which may influence between-host transmission, for example, by positively or negatively affecting parasite shedding [32]. Host body size also affects key host characteristics, such as longevity and ecological carrying capacity [20], that may affect host availability to parasites.

**Table 1.** Host and parasite traits predicted to affect the evolution of parasite generalism that are explored in this study.

trait	levels	previous hypotheses or observations
host seeking behaviour	*active*: e.g. mobile parasites that seek out hosts; *passive*: e.g. parasites transmitted during host–host contact or via ingestion	parasites that actively seek out hosts should be more specific than parasites that are transmitted by direct contact between hosts. Parasites transmitted via ingestion should be less specific than parasites infecting through other routes [16].
infection site	*endoparasite*: lives inside the host; *ectoparasite*: lives on the surface of the host	infection site will give different opportunities for transmission mode; for example, the mobility of infective stages may affect the evolution of generalism [16]. higher number of host species per parasite and network connectance observed for endoparasites compared with ectoparasites of fish [17].
life cycle	*complex*—transmission involves one or more intermediate hosts *direct*—no intermediate hosts	parasites with complex life cycles exhibit more range in acceptable hosts and may be more likely to evolve generalism [16]. direct life cycle parasites of primates are less host-specific than complex life cycle parasites [18].
trophic transmission	*yes*—for parasites that have complex life cycles, trophic transmission occurs when the intermediate host is ingested by the terminal host *no*—transmission to the terminal host does not involve ingestion	trophic transmission restricts exposure of intermediate parasite stages to definitive hosts according to the structure of host food webs, so the generalism of the parasites will be dependent on the dietary generalism of their definitive hosts and/or the breadth of predators of their intermediate hosts [19].
host geographic range as proxy for temperature	geographic regions: Africa; Antarctica; Australia; Indopacific; Nearctic; Neotropical; Palearctic.	allometric relationships exist between temperature and life-history parameters [20]. higher species diversity in the tropics [21,22]. digenean parasites of marine fish in tropical seas infect fewer hosts than those that parasitize fish in colder seas [21]. no relationship is observed between latitude and generalism for Monogeneans [23].
host body size	continuous (here, maximum length of fish host)	specialist Monogenean parasites tend to be found on large-bodied fish hosts [24–26]. variance in phylogenetic diversity of host species infected by fleas is negatively correlated with mean host body size [27].

Temperature can influence the hosts that a parasite infects through a number of processes. Globally, species diversity of both hosts and parasites tends to increase near the tropics [21], an increase that can be explained by increased temperature [22]. Parasites' host diversity may therefore increase with environmental temperature simply because there are more host species available to be parasitized. Temperature can also affect the survival and infectivity of parasites' free-living stages [33]. Given the importance of free-living transmission stages for many parasites, temperature may therefore have an important effect on evolution of parasites' host diversity. Finally, as with body size, temperature can affect important host characteristics that might affect host availability [20,34].

General predictions regarding correlations between characteristics of host, parasite or environment and a parasites' host diversity largely come from simple verbal models, and empirical tests of these predictions are often equivocal [10,35]. Here, we use invasion analyses [36] to predict when generalist parasites can invade a multi-host system with multiple specialist parasites. We use allometric scaling relationships to characterize the body size– and temperature-dependence of key host traits, and use the model to predict how host body size, temperature and transmission mode affect the evolution of parasite host-generalism. We then calculate structural and phylogenetic generalism metrics [37] from an extensive dataset of macroparasites of fish [38] to test these predictions. With this approach, we aim to improve our understanding of the ecological and evolutionary factors that contribute to parasite host-generalism.

## 2. Model derivation

We develop a model to predict under what conditions a generalist parasite can invade a system already occupied by a specialist parasite. We begin by considering the dynamics of a community of *H* hosts, where each host species *H_j_* can be infected by a specialist parasite *P_j_*, which also has a free-living stage in the environment. We let *N_j_* be the total number of hosts of species *j.* These hosts can be found in three infection classes: *S_j_* is the number of susceptible (uninfected) hosts, *I_j,s_* is the number of hosts that are singly infected with the specialist parasite, and *D_j,s,s_* is the number of hosts that are doubly infected with the specialist parasite. Double infections by the specialist parasite do not mean that only two individual parasites are present in a host, but simply allow for re-infection of an already infected hosts to avoid bias when the generalist parasite is introduced: for co-infection models, if the resident strain (in this case, the specialist) cannot produce double infections, the co-infection model is biased [39]. Without this, an invading strain has an advantage when increasing from rarity because it can infect all susceptible hosts and all hosts that are infected with the resident strain, whereas the resident can only infect susceptible hosts because hosts that are singly infected with the invading strain are rare. This creates a negative frequency-dependent fitness advantage.

Infected hosts are assumed to shed parasites into the environment at a host-specific, per-parasite rate of *λ_j_*, with *P_j_* representing the abundance of specialist parasites of host *j* in the environment. The full dynamics of the system for each of the *j* host species (*j* = 1, … ,*H*) are defined below:
2.1
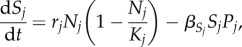

2.2
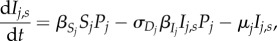

2.3
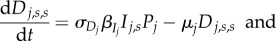

2.4
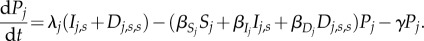


In the absence of any infection, we assume that each host population grows logistically, at a maximum per-capita rate of *r_j_* and with a carrying capacity *K_j_*. Infection occurs through contact with parasites in the environment. We assume that parasites actively seek out hosts, but we allow for the possibility that the contact rate depends on the host infection status (if, for example, parasites avoid already-infected hosts). Thus, 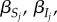 and 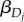 are the contact rates between parasites and susceptible, singly infected hosts and doubly infected hosts, respectively. We also allow for variation in the susceptibility of singly infected hosts to becoming double-infected with the parameter 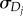, which is the probability of double infection. For example, if 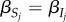 and 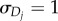, the parasite does not discriminate between susceptible and singly infected hosts, and all contacts with singly infected hosts lead to double infections. On the other hand, if 
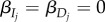
, then the parasite avoids already infected hosts, and *D_j,s,s_* = 0. We assume that infected hosts die at the host-specific rate *μ_j_*, a rate that is independent of infection status.

As we discuss more below, we assume that the parasite shedding rate *λ_j_* depends on the abundance of parasites within the host, which we assume is set by host traits. Thus, the shedding rate is the same for single and double infections. Parasites are removed from the environment due to contact with hosts, and are also lost at the per-capita rate *γ*. Note that contact with a host will result in the removal of the parasite from the environment, even if it does not result in a new infection. For example, if 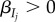 but 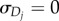, contact with a singly infected host results in removal of the parasite from the environment but not double infection.

To study the evolution of generalism, we take an invasion analysis approach [36] and ask whether a generalist parasite could invade this community of hosts and specialist parasites. The generalist parasite may infect all, or only a subset, of the hosts in the community. To account for the generalist parasite, the equations for any host species *k* that can be infected by the generalist become:
2.5
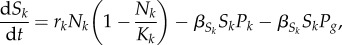

2.6
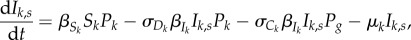

2.7
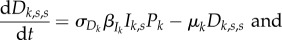

2.8
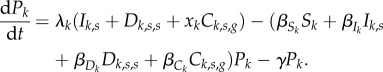


Susceptible hosts of species *k* can now become infected with the generalist parasite, *P_k_*. We assume, for simplicity, that the contact rates of the generalist parasite with hosts 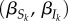
are the same as those of the specialist parasite. We also allow hosts that are singly infected with the specialist parasite to become co-infected on contact with the generalist parasite, with 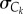 being the probability of co-infection, given contact. Hosts that are doubly infected with the specialist parasite cannot be infected by the generalist parasite. Because we assume that the total abundance of parasites is set by host traits, we introduce the parameter *x_k_* to account for competition between the strains for host resources. If *x_k_* = 0.5, then the co-infecting strains equally partition host resources, and each is shed at half the rate it attains in single infection. As above, if a parasite in the environment does not avoid contact with co-infected hosts, it is removed from the environment at the rate 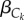.

We also need to consider the dynamics of host species *k* individuals that are singly infected with the generalist parasite (*I*_*k*,*g*_)or are co-infected with the specialist and generalist parasite (*C*_*k*,*s*,*g*_):
2.9
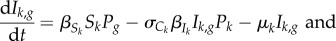

2.10
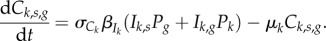


Note that we do not consider the dynamics of hosts that are doubly infected with the generalist parasite. This is because, in an invasion analysis, we are interested in whether the generalist parasite can invade the community when it is very rare (so 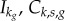, and *P_g_* are all assumed to be very close to 0). In such an analysis, we can ignore the dynamics of any variable that depends on products of 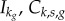, or *P_g_* [39]. Since double infections require contact between hosts that are singly infected with the generalist parasite and generalist parasites in the environment, we can ignore this variable.

Finally, we consider the dynamics of the generalist parasite in the environment:
2.11
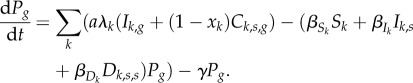


Again, we can ignore the loss of generalist parasites from the environment due to contact with singly infected (*I_k_*,*_g_*) and co-infected (*C*_*k*,*s*,*g*_) hosts because such contacts can be assumed to be very rare during the invasion. We assume that the generalist parasite sheds parasites at the rate *aλ_k_*, where *a* accounts for the cost of generalism. In the absence of such costs, the generalist would always be able to invade. Note that such costs could be accounted for by assuming that the contact rates for generalist parasites were lower than those of specialist parasites.

### (a) Invasion analysis and host allometry

To study the evolution of generalism, we determine whether the generalist parasite can invade the community by studying the stability of the epidemiological equilibrium where all of the variables involving the generalist parasite are equal to 0 (i.e. for each host species *k* that can be infected with the generalist parasite, *I_k_*_,*g*_ = *C_k_*_,*s*,*g*_ = 0, and *P_g_* = 0). We are interested in knowing when this equilibrium is unstable, that is, when the generalist parasite can increase from rarity and invade the system. Mathematically, this is governed by the eigenvalues of the Jacobian matrix for the full system (equations (2.1)–(2.11)). It can be shown (appendix A, electronic supplementary material), after applying the Next Generation Theorem [40], that the invasion condition for the generalist parasite is *R_m_* > 1 where


2.12
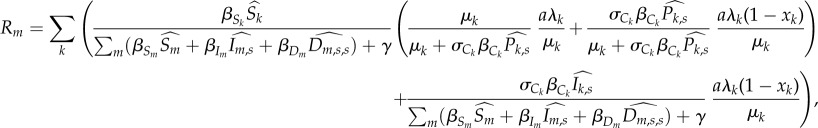


*R_m_* is therefore very analogous to the familiar basic reproductive rate for a parasite *R*_0_, except that *R*_0_ is the expected production of new infections per infection when the parasite is invading a community that is fully susceptible, whereas *R_m_* is the expected number of new generalist parasites per parasite when the generalist is invading a community where the number of susceptible hosts is set by the specialist parasites.

This expression is complex, but has an intuitive biological interpretation. The first term is the probability that a generalist parasite infects a susceptible host of species *k*. The ^ over a host variable denotes that the host variable is at its equilibrium value, as determined by the host's interaction with its specialist parasite. The second term is the probability that a singly infected host remains singly infected for its lifetime, multiplied by the expected number of parasites that a singly infected host will shed. The third term is the probability that the host becomes co-infected, multiplied by the expected number of parasites shed by a co-infected host. The fourth term is the probability that a parasite co-infects a host infected by the specialist, multiplied by the expected number of parasites shed by a co-infected host.

The above derivation is general for a community with any number of hosts, but we focus our analysis on the simpler case where there are only two hosts. We are interested in understanding how host traits, parasite traits and the environment influence the magnitude of *R_m_*. If increasing the value of some host trait increases *R_m_*, then this trait makes it easier for the generalist parasite to invade, and we conclude that the trait has a positive effect on the evolution of generalism. Mathematically, this is equivalent to asking about the derivative of *R_m_* with respect to the trait of interest.

To facilitate a comparison between the model and data, we focus our analysis on the effect of host body size and temperature, taking advantage of the fact that many key parameters of the model are likely to be allometric functions of host body size and temperature. In particular, host carrying capacities (*K_j_*), maximum per-capita reproductive rates (*r_j_*) and mortality rates (*μ_j_*) will scale with host body size and temperature [20] as

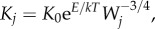


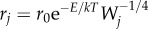


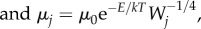
where e^−*E*/*kT*^ is the Boltzmann factor, which describes how temperature affects reaction kinetics (e.g. metabolic rate), *W_j_* is the body mass of host *j,* and *K*_0_, *r*_0_ and *μ*_0_ are proportionality constants. *E* is the average activation energy of rate-limiting biochemical metabolic reactions, *k* is Boltzmann's constant and *T* is temperature. Since our dataset deals with parasites of ectotherms, we assume that *T* is the temperature of the environment, and that it is also the same for both hosts. Increasing mass will decrease the carrying capacity and the reproductive and mortality rates, whereas increasing temperature will decrease carrying capacity but increase reproductive and mortality rates.

Host body size and temperature should also affect parasite abundance. For endoparasites, abundance will scale with body mass, because those parasites depend on volume (whether of the body or of a specific organ), whereas for ectoparasites, abundance will scale with body mass to the two-third power, because those parasites depend on surface area. Hechinger [31] extensively developed metabolic scaling equations for parasite abundance. He showed that the density of parasites should be assessed relative to their use of host space. Thus, the density of internal parasites should be proportional to host mass *W*, whereas the density of external parasites should be proportional to *W*^2/3^. Moreover, parasite abundance should be limited by the availability of resources; he assumes that resources are provided to parasites at a rate proportional to mass-specific metabolic rate (*W*^−1/4^). Therefore, total parasite abundance should be proportional to the product of these two mass-specific quantities. We therefore assume that parasite abundance scales with mass to the three-quarter power for endoparasites and mass to the five-twelfth power for ectoparasites. We assume that shedding rate scales linearly with parasite abundance, giving

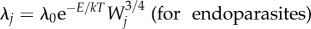


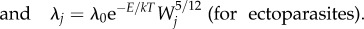


Note that *λ_j_* is a product of two parameters: a parameter that defines how parasite abundance scales with host body size and a parameter that defines the shedding rate per parasite.

If we add these expressions into the *R_m_* expression above, we attain host body size–, temperature- and infection site–dependent criteria for the evolution of generalism. Note that we use the infection site to mean inside (endo) versus on the surface of the host (ecto). By taking the derivative of *R_m_* with respect to host mass and temperature, we can investigate how these key parameters influence the evolution of generalism.

Moreover, by varying the values of other parameters, we can explore very different infection scenarios. In particular, we vary the number of specialist parasites in the system, whether co-infection occurs, and whether the parasite avoids contact with hosts that are not susceptible to infection, and investigate how these changes affect the evolution of generalism. Table 2 reports the parameter values needed to construct these different scenarios and the relevant state variables.

**Table 2.** Effect of increasing body size and temperature on *R_m_* for directly transmitted parasites under model variants considered in the text and in appendix A (electronic supplementary material).

case	no. specialist parasites	co-infection?	avoidance of non-susceptible hosts?	constant host population size?	effect of increased body size on *R_m_*	effect of increased temperature **on** *R_m_*
1	1	no	yes	no	increase (endoparasite) unimodal (ectoparasite)	decrease
2	2	no	yes	no	increase (both)	none
3	2	no	no	no	generalist cannot invade	generalist cannot invade
4	1	yes	yes	no	increase (endoparasite) unimodal (ectoparasite)	decrease
5	2	yes	yes	no	increase (both)	increase
6	2	yes	no	no	generalist cannot invade	generalist cannot invade
7	2	no	yes	yes	increase (both)	none
8	2	no	no	yes	increase (both)	increase
9	2	yes	yes	yes	variable (both)	variable
10	2	yes	no	yes	increase (both)	increase

We explicitly consider the first case in table 2. Substituting these parameters into equation (2.12), we find that the specialist-only system will be unstable (i.e. generalism will evolve) whenever
2.13
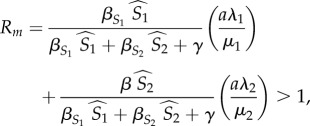
where 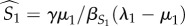 and 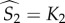 are the equilibrium host abundances when only the resident parasite is present.

Substituting the equilibrium abundances of the primary and secondary host simplifies the *R_m_* expression to

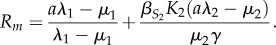


In particular, it is immediately clear that, *all else being equal*, *R_m_* will be larger for endoparasites than ectoparasites because the shedding rate will be higher. Thus, generalism is more likely to evolve for endoparasites than ectoparasites.

For simplicity, we let the mass of the secondary host be *fW*, where *W* is the mass of the primary host. To investigate how the evolution of generalism is affected by host body size (*W*), the body size ratio between the two hosts (*f*) and the temperature of the environment (*T*), we look at the derivatives of *R_m_* with respect to *W*, *f* and *T*. We will consider these derivatives for both endoparasites and ectoparasites.

For endoparasites, *R_m_* is an increasing function of host body size *W*:

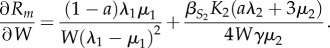


Thus we predict that parasites infecting large-bodied hosts are more likely to be generalists than parasites infecting small-bodied hosts. Specifically, when looking across a large number of host–parasite associations, the model predicts that there will be a positive correlation between host-generalism and the body size of each parasite's largest host. Moreover, there will be a positive correlation between host-generalism and mean host body size: mean body size is 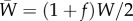, and 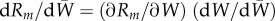, where 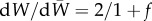, which must be positive.

Similarly, *R_m_* is an increasing function of *f*, the relative difference in body size between hosts:

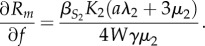


Increasing *f* increases the size of the secondary host; *R_m_* is the sum of terms dealing with infection in the primary and secondary host, and, as we have already shown, increasing host mass increases *R_m_*. Thus we predict that there should also be a positive correlation between host-generalism and the coefficient of variation (CV) in host body size**.** The CV is a better metric for this prediction than the raw variance because the variance in body sizes among hosts will be positively correlated with mean body size among hosts.

For ectoparasites, the response of *R_m_* to changes in body size is more complicated. The effects of increasing host mass or increasing the difference in mass between hosts are given by the derivatives

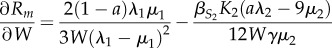
and

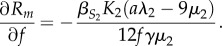


For both of these derivatives, the sign is determined by (*aλ*_2_ − 9*μ*_2_). Using the scaling functions for *λ*_2_ and *μ*_2_, we find that increasing host body size will increase *R_m_* if 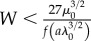.

This indicates that it will be easier for a generalist ectoparasite to invade when host body size increases, but only up to a point. Put another way, this predicts that there should be few generalist parasites of either very small bodied or very large-bodied hosts. If the primary host is very large, then it will be easier for a generalist to invade if the secondary host is much smaller (i.e. *f* is small). However, it is important to note that both of these predictions now depend on the values of the parameters, making these predictions somewhat more challenging to address.

The effect of temperature will be the same for both endo- and ectoparasites, as the parasite infection site has no effect on temperature scaling. For both, increasing temperature decreases *R_m_*:

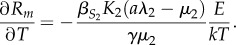


Thus we predict that generalism should be more likely in colder environments than in warmer ones. A corollary of this (which we cannot address using our current dataset) is that generalism should be more common among parasites of ectotherms than endotherms.

This model is intentionally simple. In appendix A (electronic supplementary material), we investigate the sensitivity of our predictions to the assumptions made by this model by considering nine alternative models presented in Cases 2–10 in table 2. We also considered how the predictions change for a trophically transmitted parasite, when there is a single intermediate host that consumes parasites in the environment, and then transmits those parasites to either of two definitive hosts (appendix B, electronic supplementary material). Table 3 shows the results for trophically transmitted parasites. For this analysis, we only considered endoparasites, since there are no trophically transmitted ectoparasites in our dataset.

**Table 3.** Effect of increasing body size and temperature on *R_m_* for trophically transmitted parasites (see appendix B, electronic supplementary material).

case	no. specialist parasites	co-infection?	parasite regulates population growth?	avoidance of already infected hosts?	effect of increased body size on *R_m_*	effect of increased temperature on *R_m_*
11	1	no	yes	yes	increase	decrease
12	2	no	yes	yes	variable	variable
13	2	no	yes	yes	variable	variable

What these analyses reveal is that, for direct life cycle parasites, the effect of host body size is almost always to increase the value of *R_m_*, thereby making it easier for generalists to invade. This is because larger hosts support a larger parasite population size, thereby increasing shedding, and larger hosts have lower mortality rates. Thus, the total parasite production increases with host body size. The effect of temperature on direct life cycle parasites is more complicated, and depends on the modelling assumptions. Interestingly, the results for cases 3 and 6 indicate that if parasites are removed from the environment by non-susceptible hosts, the generalist can never invade. For trophically transmitted parasites, on the other hand, the results are much more variable, suggesting that general patterns may be difficult to ascertain for trophically transmitted parasites.

### (b) Comparison of model predictions to data

#### (i) Data collection methods

The Fish Parasite Ecology Database contains more than 38 000 records of associations between 4650 host fish species and 11 802 helminth parasites, as well as ecological, biogeographical and phylogenetic information on the host species, including host body size and geographic region [38]. As the number of ectoparasite species was low, additional parasite–host records were included for 105 crustacean parasite species (a group not previously represented in the database), and for all parasites we included data on parasite life-history traits including reproductive strategy, life cycle stages and transmission routes from a range of primary literature sources. If there was any ambiguity regarding the taxonomic status of the parasites they were excluded from the database. To remove synonyms and other inconsistencies, host species names were quality-checked by Entrez Direct queries (www.ncbi.nlm.nih.gov/books/NBK179288/) to the NCBI taxonomy database and FishBase [41]. Parasite species names were checked against the NCBI taxonomy database in the same way and also checked against the NHM Host–parasite database (http://www.nhm.ac.uk/research-curation/scientific-resources/taxonomy-systematics/host-parasites/database) using a custom script and the World Register of Marine Species (WoRMS), Catalogue of Life (CoL), Integrated Taxonomic Information System (ITIS) and Global Names Index (GNI) databases through the Lifewatch Taxonomic Backbone (http://www.lifewatch.be/data-services/). All intermediate hosts were excluded, such that generalism in parasites with complex life cycles was based on the definitive hosts only. After data cleaning, we were left with 23 331 unique host–parasite associations between 8846 parasite species and 4237 fish hosts.

#### (ii) Generalism metrics

We defined each parasite's specialism/generalism according to four metrics, representing both structural (number of hosts) and phylogenetic diversity (table 4), without accounting for parasite abundance as this information was not available in the original database [38]. The structural metrics, degree (number of hosts) and *G* (binary measure), were calculated directly from the host–parasite database, while phylogenetic metrics SPD and standardized Faith's phylogenetic diversity (SES-PD) [37,44] were generated for all parasites based on the pairwise genetic distances between each parasite's hosts. Host mitochondrial DNA sequences (complete mitochondrial genomes and full or partial sequences from mitochondrial loci [appendix C, electronic supplementary material, figure S1]) were gathered from the NCBI nucleotide database and processed as described in appendix C, electronic supplementary material. To calculate SES-PD, we used the ses.pd() function implemented in the *picante* package in R [45] to generate the standardized effect size of Faith's phylogenetic diversity based on 1000 runs. SES-PD compares the actual Faith's phylogenetic diversity value for each parasite to a summary of the metric calculated after repeatedly shuffling taxa labels of all taxa in the phylogeny in order to assess if phylodiversity is high or low for a given number of hosts.

**Table 4.** Generalism metrics calculated from host–parasite database.

metric	description	facet
degree	number of hosts (links in host–parasite network [42])	structural
*G*	binary measure, *G* = 1 if degree >1	structural
SPD	mean pairwise phylogenetic distance between all hosts [43], SPD = 0 for *G* = 0	phylogenetic
SES-PD	standardized effect size of Faith's phylogenetic distance [44] based on 1000 runs, with a negative value indicating that the observed tree length (here, the length of the parasite's host tree with the root excluded) is smaller (the hosts are more closely related) than what you might find by chance	phylogenetic

#### (iii) Database meta-analysis

The generalism metrics for each parasite species were compared with parasite traits to test the model predictions. Because the generalism metrics come from very different distributions, we used GLMs with different error distributions for statistical analyses. For degree, we used negative binomial regression with a log link function (glm.nb() in the *MASS* package in R); for *G*, logistic regression (glm(family = ‘binomial’) in R); and for SPD and SES-PD, linear regression (lm() in R) [46]. For each of the generalism metrics (dependent variables), we conducted univariate and multivariate regression with host body size, life cycle (direct versus trophic), and geographic region (warm versus cool) as independent variables.

We included parasite life cycle as an independent variable since the modelling results show that life cycle strongly affects model predictions. We note, however, that the life cycle and the infection site are confounded in the dataset as nearly all of the direct life cycle parasites are ectoparasites (4216/4226), whereas all (3076) of the trophically transmitted parasites are endoparasites.

The modelling results present separate predictions for the effect of mean host body size, maximum host body size and CV, so these three independent variables are presented in separate models. In the multivariate regression, mean and maximum host body size scaled and centred. Note that CV of the host length is only calculated for parasites with more than one host.

Geographic region was included as a proxy for temperature. Regions were assessed as defined in table 1 and divided into two groups, where Antarctica (ANT), Nearctic (NEA) and Palearctic (PAL) were assumed to be colder than Africa (AFR), Australia (AUS), Indopacific (IND) and Neotropical (NEO) regions. Some host–parasite associations were reported in more than one region, so for the purposes of the univariate regression with region as an independent variable, and for all of the multivariate regressions, generalism metrics were calculated separately for parasites in each region.

## 3. Results

### (a) Host-generalism metrics

The majority of parasites examined in this database were specialists, with 61% of parasites having degree = 1 (G = 0). The distribution of degree (number of hosts) was highly overdispersed; 92% of parasites had five or fewer hosts, but the parasite with the most hosts, nematode *Hysterothylacium aduncum*, had degree 188. When metrics were calculated separately by geographic region, regional parasites with more than one host made up 35% of all regional parasites, and the parasite with the most hosts was *H. aduncum* in the PAL region, with 127 hosts.

A parasite's hosts were generally more related than expected by chance, as 88% of parasites with more than one host had negative values for SES-PD. In addition, while the mean pairwise genetic distance between all hosts in the database was 0.263 (standard deviation = 0.034; appendix C, electronic supplementary material, figure S2), the mean genetic distance (SPD) between hosts of each parasite with more than one host was 0.18.

### (b) Host body size

**Figure 1.**
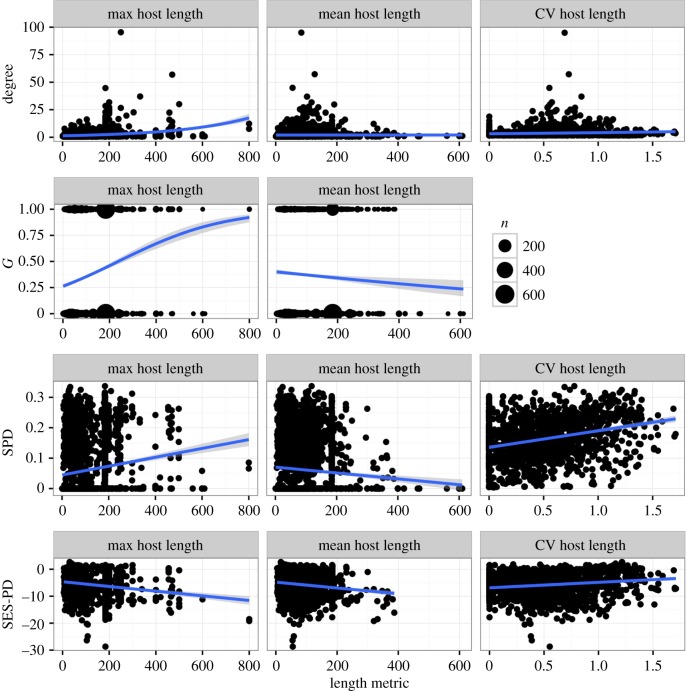
Relationship between generalism metrics (rows) and length metrics (columns) for directly transmitted parasites. Blue line shows fitted model with confidence intervals in grey. For *G*, size of points scale with number of parasite species having the same mean or max value of host lengths.

The model predicts that for direct life cycle parasites, there should be a positive correlation among parasites' generalism metrics and both the maximum and mean host body size, with a particularly strong positive correlation between generalism and the coefficient of variation in host body size. We observed a strong and significant positive correlation between many, but not all, body size metrics and generalism metrics (figure 1, table 5). In particular, the coefficient of variation in host length shows positive correlations with degree, SPD and SES-PD, while the mean host length is negatively correlated with SPD and SES-PD. Mean host length is not significantly correlated with degree, and shows a slight negative correlation with *G*. Maximum host length shows a small positive correlation with degree, *G* and SPD, and a small negative correlation with SES-PD.

**Table 5.** Relationship between generalism metrics and length metrics for directly transmitted parasites.

response (metric)	predictor	coefficient	confidence interval	*Z*-score (residual degrees of freedom)	unit
degree	mean host body length	4.72 × 10^−5^	−3.27 × 10^−4^, 4.21 × 10^−4^	0.258 (4217)	unit change in log degree per cm increase in length
	max host body length	0.00324	0.00295, 0.00353	22.3 (4217)	
	CV host body length	0.277	0.178, 0.376	5.80 (1554)	
*G*	mean host body length	−0.00127	−0.00210, −4.53 × 10^−4^	−3.03 (4217)	log odds ratio per cm increase in length
	max host body length	0.00436	0.00362, 0.00511	11.5 (4217)	
SPD	mean host body length	−9.68 × 10^−5^	−1.32 × 10^−4^, 6.11 × 10^−5^	−5.32 (4217)	unit change in SPD per cm increase in length
	max host body length	1.46 × 10^−4^	1.14 × 10^−4^, 1.77 × 10^−4^	9.16 (4217)	
	CV host body length	0.0547	0.0458, 0.0637	12.0 (1554)	
SES-PD	mean host body length	−0.0107	−0.0140, −0.00738	−6.35 (1565)	unit change in SES-PD per cm increase in length
	max host body length	−0.00867	−0.0110, −0.00630	−7.18 (1565)	
	CV host body length	2.00	1.458, 2.55	7.21 (1554)	

**Figure 2.**
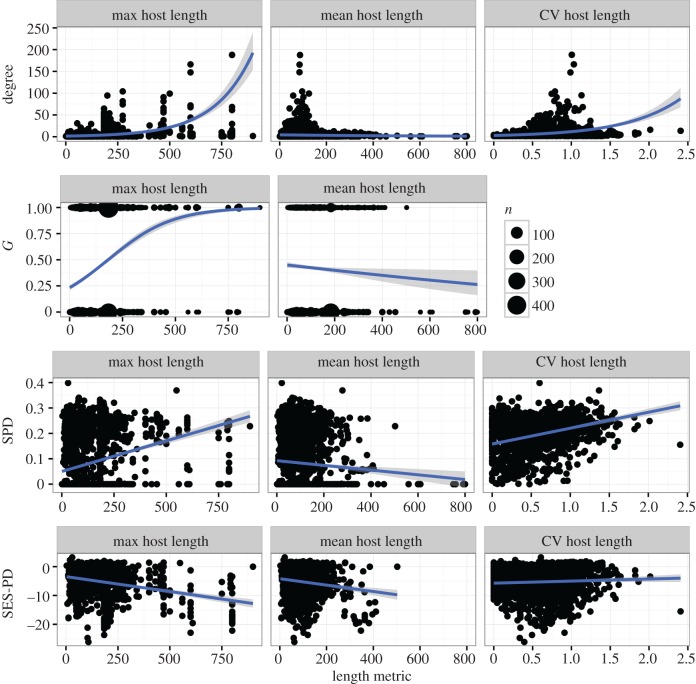
Relationship between generalism metrics (rows) and length metrics (columns) for trophically transmitted parasites. Blue line shows fitted model with confidence intervals in gray. For *G*, size of points scale with number of parasite species having the same mean or max value of host lengths.

For trophically transmitted parasites the model makes no definitive predictions, such that the correlation between a parasite's host-generalism and host body size can be positive or negative. Interestingly, however, we observe identical patterns of correlation between host-generalism metrics and host body size for trophically transmitted parasites as we did for direct life cycle parasites (figure 2, table 6), with the exception that mean host length is slightly negatively correlated with degree.

**Table 6.** Relationship between generalism metrics and length metrics for trophically transmitted parasites.

response (metric)	predictor	coefficient	confidence interval	*Z*-score (residual degrees of freedom)	unit
degree	mean host body length	−0.00152	−0.00211, −9.27 × 10^−4^	−5.77 (3075)	unit change in log degree per cm increase in length
	max host body length	0.00545	0.00508, 0.00583	36.2 (3075)	
	CV host body length	1.43	1.26, 1.60	19.8 (1301)	
*G*	mean host body length	−0.00104	−0.00193, −1.646 × 10^−4^	−2.31 (3075)	log odds ratio per cm increase in length
	max host body length	0.00657	0.00571, 0.00745	14.8 (3075)	
SPD	mean host body length	−9.31 × 10^−5^	−1.38 × 10^−4^, −4.78 × 10^−5^	−4.03 (3075)	unit change in SPD per cm increase in length
	max host body length	2.41 × 10^−4^	2.10 × 10^−4^, 2.72 × 10^−4^	15.3 (3075)	
	CV host body length	0.0625	0.0527, 0.0724	12.4 (1301)	
SES-PD	mean host body length	−0.0112	−0.0153, −0.00697	−5.24 (1301)	unit change in SES-PD per cm increase in length
	max host body length	−0.0104	−0.0123, −0.00841	−10.4 (1301)	
	CV host body length	0.722	−0.0138, 1.46	1.93 (1301)	

The same relationships are observed in the multivariate analysis (tables S1–S4 in appendix C, electronic supplementary material).

### (c) Temperature/geographic range of all parasites

**Figure 3.**
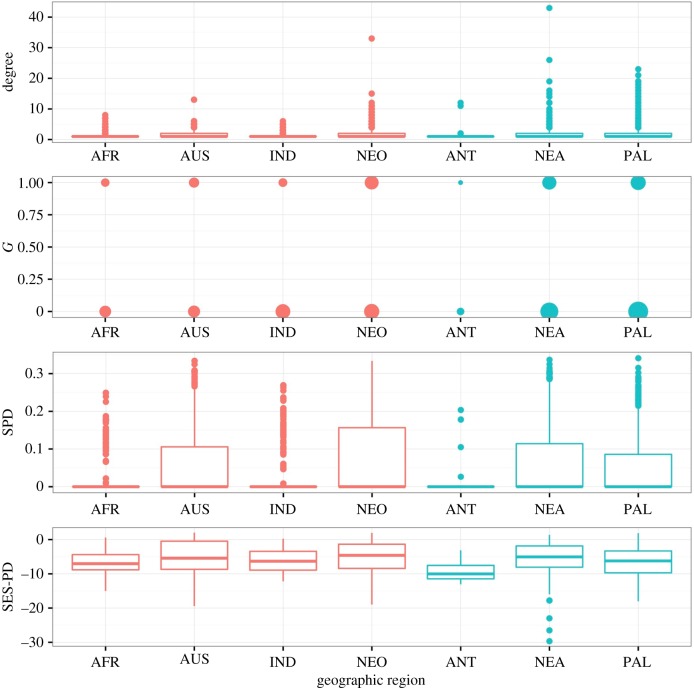
Generalism metric by geographic region, for directly transmitted parasites. Warm regions are shown in red and cool regions in blue. For *G*, which is binary, point size scales with the number of parasites that have each value shown.

**Figure 4.**
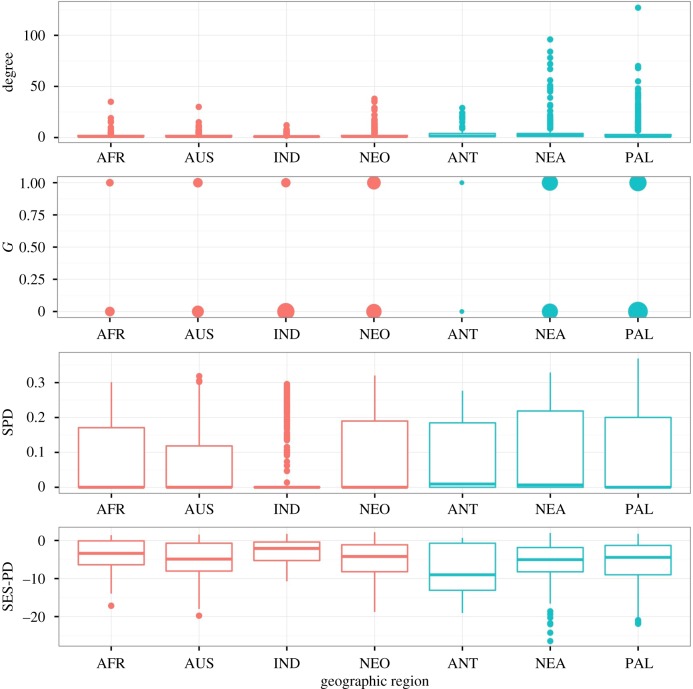
Generalism metrics by geographic region for trophically transmitted parasites. Warm regions are shown in red and cool regions in blue. For *G*, which is binary, point size scales with the number of parasites that have each value shown.

The models make very different predictions about how temperature affects the evolution of generalism (tables 2 and 3), including some models predicting that generalism is more likely in colder environments. We see higher generalism metric degree in cool regions for direct life cycle parasites (figure 3, table 7) and higher degree, *G* and SPD in cool regions for trophically transmitted parasites (figure 4, table 8). This is partly driven by the correlation between-host body size and generalism, as host body size is also positively correlated with cooler geographic regions, such that the relationship between host body size and generalism varies by region (appendix C, electronic supplementary material, figure S3 and figure S4). For example, for directly transmitted parasites there is a negative correlation between maximum host length and SES-PD in the warm regions, and a non-significant negative relationship between maximum host length and SES-PD in the cool regions, while for trophically transmitted parasites, there is a positive correlation between maximum host length and SES-PD in warm regions, but a negative relationship in cool regions (appendix C, electronic supplementary material, table S4).

**Table 7.** Generalism metrics by geographic regional group for directly transmitted parasites.

response (metric)	predictor	coefficient	confidence interval	*Z*-score (residual degrees of freedom)	unit
degree	geographic group (ref = ‘warm’)	0.0884	0.0396, 0.137	3.55 (4831)	unit change in log degree for cool group
*G*		0.0341	−0.0881, 0.157	0.547 (4831)	log odds ratio for cool group
SPD		−1.87 × 10^−4^	−0.00508, 0.00470	−0.07487 (4831)	unit change in SPD for cool group
SES-PD		−0.424	−0.867, 0.0185	−1.88 (1551)	unit change in SES-PD for cool group

**Table 8.** Generalism metrics by geographic regional group for trophically transmitted parasites.

response (metric)	predictor	coefficient	confidence interval	*Z*-score (residual degrees of freedom)	unit
degree	geographic group (ref = ‘warm’)	0.663	0.591, 0.734	18.19 (3768)	unit change in log degree for cool group
*G*		0.581	0.447, 0.716	8.49 (3768)	log odds ratio for cool group
SPD		0.0286	0.0219, 0.0352	8.43 (3768)	unit change in SPD for cool group
SES-PD		−1.09	−1.60, −0.570	−4.13 (1481)	unit change in SES-PD for cool group

### (d) Infection site of directly transmitted parasites

The allometric scaling model predicts that for parasites with a direct life cycle, generalism should be higher in endoparasites compared with ectoparasites. In the fish dataset of macroparasites, there are 4226 parasites with a direct life cycle, of which only 10 (0.2%) are endoparasites. Due to the small sample size for endoparasites, no significant difference is found for generalism metrics by the infection site (not shown). The 10 directly transmitted endoparasites all have degree ≤4, and while 94% of directly transmitted ectoparasites also have degree ≤4, the maximum degree for this group is 95.

## 4. Discussion

The number of hosts a parasite can infect has important epidemiological and evolutionary implications [1–4]. Previous authors have approached the study of host-generalism using a comparative approach, analysing groups of closely related parasites that differ in the number of hosts infected by species within the group, in an attempt to identify the key factors that influence host-generalism [24,25,27,29,30]. On the basis of these studies, verbal models have been developed that suggest how such factors might influence the evolution of host-generalism more generally (table 1, and see reviews [10,35]). For example, host-generalism might be influenced by phylogenetic constraints if the fitness cost of being a generalist is lower when the hosts are closely related [47]. However, while these verbal models are intuitively appealing, empirical tests of their predictions are often equivocal [10,35].

Here, we take a different approach, deriving simple mathematical models that incorporate host, parasite and environmental characteristics using principles from metabolic scaling theory [20,31]. This allows us to incorporate biologically feasible constraints on the epidemiological processes included in mathematical models of host–parasite interaction. We then use invasion analysis [36] to study how variation in host body size, temperature, infection site and parasite life cycle influence the evolution of host range, here quantified as the effect of these characteristics on the magnitude of a generalist parasite's invasion fitness. These analyses predict that parasites are more likely to evolve a generalist strategy when hosts are large-bodied, when variation in host body size is small, and, under some assumptions, in cooler environments.

This mathematical approach can help illuminate the strengths and the weaknesses of verbal models for the evolution of host range. In particular, the dynamical interaction between hosts and parasites can have counterintuitive outcomes that affect the validity of verbal model predictions. For example, previous authors have suggested that host specificity is more likely to evolve when hosts are abundant, because increased abundance increases the probability that a specialist will encounter its host [48,49]. Our model analyses reveal that host abundance is unlikely to be directly relevant to the evolution of host range. This is because, in our base model, parasite fitness depends not on the *total* abundance of hosts, but on the abundance of *susceptible* hosts. The dynamic interaction between the host and parasite causes the abundance of susceptible hosts to depend on parasite traits rather than host traits like carrying capacity. Thus the fitness of the generalist does not depend directly on host abundance, which can be seen from the fact that carrying capacity rarely appears in the generalist *R_m_* expressions (appendix A, electronic supplementary material). However, if parasites do not affect host population size, but cannot distinguish between susceptible and non-susceptible hosts (cases 8 and 10 in table 2), the expressions for *R_m_* do depend on host abundances, and specialist parasites will be favoured when it is likely that they are able to come in contact with a host, as suggested by the verbal theory. Thus, by analysing the question mathematically, we come to a more complete understanding of when an intuitive verbal prediction is likely to apply.

The results for cases 8 and 10 are an interesting contrast to the results for cases 3 and 6. If the parasite regulates host population size, then not being able to distinguish between susceptible and non-susceptible hosts is highly detrimental to generalist invasion success, with generalists not being able to invade at all. If host abundances are constant, however, generalists can still potentially invade.

There are, however, important challenges in attempting to test the predictions of mathematical models using data from real host–parasite systems. In particular, theory on the evolution of specialization indicates that the crucial determinant of host range is the trade-off between a parasite's ability to infect multiple hosts and its fitness on each host [7,50]. Here we quantified that trade-off using the parameter *a*, which reduced the shedding rate of a generalist parasite to a fraction of that of a specialist parasite. Such a reduction in shedding might be caused by a reduction in infection intensity, as other studies have shown that generalist parasites often have lower infection intensities than specialists [8,9]. Indeed, many experimental evolution studies have shown that as a parasite is forced to adapt to a novel host, it gradually loses its infectiousness and/or replication ability in the original host, such that, when the parasite is able to infect both the original and novel host, its fitness is lower in each than when it is specialized [51]. However, fitness trade-offs are notoriously challenging to measure, so assessing the importance of such trade-offs in the evolution of host range in any large host–parasite dataset is practically impossible. Using allometric scaling relationships to define model parameters in terms of easily measurable host traits like body size and temperature provided us with an opportunity to explicitly connect the model with data.

A second general issue with connecting the model results to data is that of phylogenetic relatedness. The model only makes predictions about the number of hosts that a parasite can infect. In reality, however, we want to distinguish between a parasite that infects *n* hosts within the same taxon and a parasite than infects *n* hosts across many taxa. Here we addressed that issue by using several measures of host-generalism (table 4). We measured ‘structural’ generalism using the number of hosts (degree and *G*), and we measured ‘phylogenetic’ generalism using metrics that account for the phylogenetic distance between hosts (SPD and SES-PD). SPD, which measures the mean pairwise phylogenetic distance between hosts, has been shown to correlate with degree [37], so we also included a measure of phylogenetic generalism that is scaled to remove the association with number of hosts (SES-PD). SES-PD therefore attempts to measure only the phylogenetic distinctiveness of the host range, so a parasite with only two hosts could have a much higher value of SES-PD than a parasite with 10 hosts. Although the model is directly making predictions about structural generalism we are assuming that phylogenetic generalism is more likely under the same conditions as structural generalism.

The models predict that for direct life cycle parasites, increasing host body size increases the fitness of the generalist parasite, suggesting that there should be positive correlation between host body size and a parasite's host-generalism. For trophically transmitted parasites, the model predictions were more complicated, suggesting that this correlation could be positive or negative, depending on model assumptions and the value of other parameters. Interestingly, previous verbal models for host range evolution have suggested the correlation between host-generalism and host body size should work in the opposite direction, with high host specificity evolving when hosts are large-bodied [27], supposedly because large-bodied species are longer-lived, and thus are more predictable in their availability. However, the predictability of a resource (in this case, the host) depends on the probability of the agent encountering that resource [48], which is determined not by resource lifespan but by abundance. Thus the observed allometric relationship between body size and abundance would seem to run counter to this verbal model. Nevertheless, a number of studies have shown a negative correlation between mean or maximum host body size and generalism [24,25,27]. We examined this correlation in our fish–macroparasite database using different metrics of host size (size of a parasite's largest host species, mean size of all hosts and the coefficient of variation in host size) and of host-generalism. By using summary metrics for the sizes of all hosts infected by each parasite, we again use imperfect measures that could affect the outcomes. In particular, mean body size is by definition a smaller number with less variation than maximum body size, and could be negatively correlated with the number of hosts simply due to the smaller number of large-bodied hosts in the distribution. For both direct and trophic life cycle parasites, we found a strong and significant positive correlation between the coefficient of variation in host body size and all metrics of host-generalism. The maximum host body size was positively correlated with all generalism metrics except SES-PD. There was a weak negative correlation between mean host body size and all metrics of host range (figures 1 and 2). Thus the data provide some support for the model predictions, especially when looking at structural generalism metrics. The negative correlation between mean host body size and generalism is interesting, as it has been observed in other studies with smaller datasets [25,27].

As it turns out, whether we interpret the model as predicting that the mean host body size for generalist parasites is larger than that for specialist parasites depends on the implicit assumption that if the generalist parasite can invade (its invasion fitness is greater than one), it displaces the specialist parasite. If we had instead assumed that the generalist parasite would coexist with any specialist parasites, our predictions would be affected. To see how, consider equation (2.13) above; a generalist parasite can invade if the entire *R_m_* expression is greater than one, whereas a parasite specialized on the smaller secondary host can invade if the second term of *R_m_* is greater than one. Thus, it is quite likely that a generalist parasite could invade even when a specialist could not because the generalist's fitness also depends on the primary host (the first term of the *R_m_* expression). A specialist parasite could invade when a generalist could not only when *a* is very small (the cost of generalism is very high). If generalists and specialists can coexist, this result suggests that both generalist and specialist parasites will infect large-bodied hosts, whereas only generalist parasites will infect small-bodied hosts. This would lead to a prediction that the correlation between mean host body size and host range should be negative, as we observed in our dataset. On the other hand, there would probably be no correlation between the maximum host body size and host range, which is not what we observed. Thus, there is no simple way to reconcile the differences between the model and data analyses, which underscores the importance of understanding how model results are translated into empirically testable predictions.

The models made very inconsistent predictions about the influence of temperature on host range evolution (tables 2 and 3). Perhaps unsurprisingly, the data are also somewhat ambivalent on this question. Our analysis suggests that the degree metric is higher in colder regions (figures 3 and 4) for both direct and trophic life cycle parasites, a result that has been observed before [21]. On the other hand, for direct life cycle parasites, the other metrics of host range do not show any significant differences between warm and cold regions (figure 3), whereas for trophically transmitted parasites, there are some positive and some negative correlations between host range and temperature. However, it is important to be aware that ectotherm body size also increases with decreasing temperature. In the database, hosts in colder waters are larger, which could be an important confounding influence on these patterns (appendix C, electronic supplementary material, figures S3, S4), and by using geographic region as a proxy for temperature we introduce measurement error due to the potential variation within regions; for example, some fish hosts in tropical regions may live in upwelling zones that are colder than the surrounding water.

Here we attempted to study ecological factors that influence host-generalism via effects on host characteristics by combining an invasion analysis of a class of simple epidemiological model with analysis of a large database of host–parasite associations. This revealed a number of places where model and data agree, as well as important areas of disagreement. We suggest that this approach is a valuable approach going forward, and highlight ways in which the models developed here could be productively extended.

In particular, previous authors have noted that important aspects of parasite fitness (in particular, abundances and shedding rates) are allometrically related to host body size [31,52]. In fact, strong positive relationships between host and parasite body size are often noted [24,25,52–54]. Because our dataset did not include any information on parasite body size, we did not incorporate such relationships into the model, but doing so would be relatively straightforward (though the analysis of such a model may not). Moreover, we have assumed that shedding rate is positively correlated with abundance, but for many parasites the opposite is true: increased within-host abundance increases density-dependence, thereby reducing parasite fecundity such that shedding is actually lower [32,55]. In that case, our parameter *λ*_0_ should be separated out into its component pieces that capture how abundance increases with body size and how shedding rate per parasite decreases with abundance.

Finally, if hosts, rather than parasites, control the contact process, then contact rates *β* may also be allometric functions of host size [56]. Indeed, in many ways, if hosts control the contact process, then *β* is very similar to the attack rate parameter of a Type I functional response, and foraging rate is well known to scale allometrically with body size [57].

Another important simplification is in our assumptions about the effect of the parasite on the host. Simple verbal models would suggest that more virulent parasites are more likely to be specialists, as the fitness trade-off for infecting multiple hosts should be steeper [35]. In our models, increasing the value of parasite-dependent host mortality *μ* would always reduce a generalist's *R_m_*, suggesting that specialism would be favoured. However, we have assumed that virulence depends only on host body size. If instead it depends upon within-host abundance, as it typically does for macroparasites, then parasite fecundity and virulence are linked. If shedding rate is a function of virulence, then whether increased virulence increases or decreases the generalist's *R_m_* depends on how quickly shedding increases with virulence: if it is large enough, then a virulent generalist can invade.

Understanding the processes that influence host range evolution is often highlighted as a key challenge for the evolutionary ecology of parasites [10,35,37], especially given that host range is closely linked to transmission, particularly in regards to reservoir hosts, spillover/emergence and changes in virulence [1–4]. Combining simple mathematical models with analysis of host–parasite databases may help reveal general principles shaping the evolution of host range.

## Supplementary Material

Appendix A: Direct life cycle model analyses

## Supplementary Material

Appendix B: Trophic transmission model analyses

## Supplementary Material

Appendix C: Supplementary Material

## Supplementary Material

Data supplementary

## Supplementary Material

Appendix E: Mathematica Analysis Script

## Data accessibility

The datasets supporting this article have been uploaded as part of the electronic supplementary material.
