# Supplementary material for: Host allometry influences the evolution of parasite host-generalism: theory and meta-analysis
Source: Philos Trans R Soc Lond B Biol Sci. 2017 Mar 13;372(1719):20160089. doi: 10.1098/rstb.2016.0089 (PMC5352816; doi:10.1098/rstb.2016.0089)
Supplement: Appendix A: Direct life cycle model analyses [file rstb20160089supp1.pdf]

Appendix A: Deriving the invasion condition and analysis of models in Table 2

Derivation of R_m from the main text

For two hosts, the general model, given by equations (5-11) in the main text, is:

```
(* Dynamics of susceptible individuals of host species 1*)
dS1dt =
  r1 (S1 + I1s + D1ss + I1g + C1sg) (1 - (S1 + I1s + D1ss + I1g + C1sg)/K1) - βS1 S1 (P1 + Pg);
(* Dynamics of individuals of host species 1 singly
 infected with its specialist parasite *)
dI1sdt = βS1 S1 P1 - σD1 βI1 I1s P1 - σC1 βI1 I1s Pg - μ1 I1s;
(* Dynamics of individuals of host species
 1 doubly infected with its specialist parasite *)
dD1ssdt = σD1 βI1 I1s P1 - μ1 D1ss;
(* Dynamics of the specialist parasite of host species 1 in the environment *)
dP1dt = λ1 (I1s + D1ss + x1 C1sg) - (βS1 S1 + βI1 I1s + βD1 D1ss + βI1 I1g + βC1 C1sg) P1 - γ P1;

(* Dynamics of susceptible individuals of host species 2 *)
dS2dt =
  r2 (S2 + I2s + D2ss + I2g + C2sg) (1 - (S2 + I2s + D2ss + I2g + C2sg)/K2) - βS2 S2 (P2 + Pg);
(* Dynamics of individuals of host species 2 singly
 infected with its specialist parasite *)
dI2sdt = βS2 S2 P2 - σD2 βI2 I2s P2 - σC2 βI2 I2s Pg - μ2 I2s;
(* Dynamics of individuals of host species
 2 doubly infected with its specialist parasite *)
dD2ssdt = σD2 βI2 I2s P2 - μ2 D2ss;
(* Dynamics of the specialist parasite of host species 2 in the environment *)
dP2dt = λ2 (I2s + D2ss + x2 C2sg) - (βS2 S2 + βI2 I2s + βD2 D2ss + βI2 I2g + βC2 C2sg) P2 - γ P2;

(* Dynamics of individuals of host species
 1 singly infected with the generalist parasite *)
dI1gdt = βS1 S1 Pg - σC1 βI1 I1g P1 - μ1 I1g;
(* Dynamics of individuals of host species
 2 singly infected with the generalist parasite *)
dI2gdt = βS2 S2 Pg - σC2 βI2 I2g P2 - μ2 I2g;
(* Dynamics of individuals of host species 1
 coinfected with its specialist and the generalist parasite *)
dC1sgdt = σC1 βI1 (I1s Pg + I1g P1) - μ1 C1sg;
(* Dynamics of individuals of host species 2
 coinfected with its specialist and the generalist parasite *)
dC2sgdt = σC2 βI2 (I2s Pg + I2g P2) - μ2 C2sg;
(* Dynamics of the generalist parasite in the environment *)
dPgdt = a λ1 (I1g + (1 - x1) C1sg) + a λ2 (I2g + (1 - x2) C2sg) -
    (βS1 S1 + βI1 I1s + βD1 D1ss + βS2 S2 + βI2 I2s + βD2 D2ss) Pg - γ Pg;
```

Whether the generalist parasite can invade will depend on the stability of the equilibrium $\left(\hat{S}_1, \hat{I}_{1,s}, \hat{D}_{1,s,s}, \hat{P}_1, \hat{S}_2, \hat{I}_{2,s}, \hat{D}_{2,s,s}, \hat{P}_2, 0, 0, 0, 0, 0\right)$. This can be evaluated by looking at the eigenvalues

of the Jacobian matrix for the full system. The Jacobian matrix at this equilibrium has a simple block upper triangular structure: $J = \begin{pmatrix} J_1 & 0 & M_1 \\ 0 & J_2 & M_2 \\ 0 & 0 & J_m \end{pmatrix}$, where J_1 is the submatrix that determines the stability of the (S_1, $I_{1,s}$, $D_{1,s,s}$, P_1) subsystem and J_2 is the submatrix that determines the stability of the (S_2, $I_{2,s}$, $D_{2,s,s}$, P_2) subsystem. J_m is the submatrix of partial derivatives involving the equations for the generalist. Because of its simple structure, the eigenvalues of the full system are given by the eigenvalues of the submatrices J_1, J_2 and J_m. Assuming that the (S_1, $I_{1,s}$, $D_{1,s,s}$, P_1) and (S_2, $I_{2,s}$, $D_{2,s,s}$, P_2) subsystems are both stable, all of the eigenvalues of J_1 and J_2 are negative. Therefore, we are interested only in the eigenvalues of J_m.

```
(* Calculating the Jacobian matrix and evaluating
  it at the equilibrium where I1,g=I2,g=C1,s,g=C2,s,g=Pg=0 *)
J = {{D[dS1dt, S1], D[dS1dt, I1s], D[dS1dt, D1ss], D[dS1dt, P1],
     D[dS1dt, S2], D[dS1dt, I2s], D[dS1dt, D2ss], D[dS1dt, P2],
     D[dS1dt, I1g], D[dS1dt, I2g], D[dS1dt, C1sg], D[dS1dt, C2sg], D[dS1dt, Pg]},
    {D[dI1sdt, S1], D[dI1sdt, I1s], D[dI1sdt, D1ss], D[dI1sdt, P1],
     D[dI1sdt, S2], D[dI1sdt, I2s], D[dI1sdt, D2ss], D[dI1sdt, P2],
     D[dI1sdt, I1g], D[dI1sdt, I2g],
     D[dI1sdt, C1sg], D[dI1sdt, C2sg], D[dI1sdt, Pg]},
    {D[dD1ssdt, S1], D[dD1ssdt, I1s], D[dD1ssdt, D1ss], D[dD1ssdt, P1],
     D[dD1ssdt, S2], D[dD1ssdt, I2s], D[dD1ssdt, D2ss], D[dD1ssdt, P2],
     D[dD1ssdt, I1g], D[dD1ssdt, I2g],
     D[dD1ssdt, C1sg], D[dD1ssdt, C2sg], D[dD1ssdt, Pg]},
    {D[dP1dt, S1], D[dP1dt, I1s], D[dP1dt, D1ss], D[dP1dt, P1],
     D[dP1dt, S2], D[dP1dt, I2s], D[dP1dt, D2ss], D[dP1dt, P2],
     D[dP1dt, I1g], D[dP1dt, I2g], D[dP1dt, C1sg], D[dP1dt, C2sg], D[dP1dt, Pg]},
    {D[dS2dt, S1], D[dS2dt, I1s], D[dS2dt, D1ss], D[dS2dt, P1],
     D[dS2dt, S2], D[dS2dt, I2s], D[dS2dt, D2ss], D[dS2dt, P2],
     D[dS2dt, I1g], D[dS2dt, I2g], D[dS2dt, C1sg], D[dS2dt, C2sg], D[dS2dt, Pg]},
    {D[dI2sdt, S1], D[dI2sdt, I1s], D[dI2sdt, D1ss], D[dI2sdt, P1],
     D[dI2sdt, S2], D[dI2sdt, I2s], D[dI2sdt, D2ss], D[dI2sdt, P2],
     D[dI2sdt, I1g], D[dI2sdt, I2g],
     D[dI2sdt, C1sg], D[dI2sdt, C2sg], D[dI2sdt, Pg]},
    {D[dD2ssdt, S1], D[dD2ssdt, I1s], D[dD2ssdt, D1ss], D[dD2ssdt, P1],
     D[dD2ssdt, S2], D[dD2ssdt, I2s], D[dD2ssdt, D2ss], D[dD2ssdt, P2],
     D[dD2ssdt, I1g], D[dD2ssdt, I2g],
     D[dD2ssdt, C1sg], D[dD2ssdt, C2sg], D[dD2ssdt, Pg]},
    {D[dP2dt, S1], D[dP2dt, I1s], D[dP2dt, D1ss], D[dP2dt, P1],
     D[dP2dt, S2], D[dP2dt, I2s], D[dP2dt, D2ss], D[dP2dt, P2],
     D[dP2dt, I1g], D[dP2dt, I2g], D[dP2dt, C1sg], D[dP2dt, C2sg], D[dP2dt, Pg]},
    {D[dI1gdt, S1], D[dI1gdt, I1s], D[dI1gdt, D1ss], D[dI1gdt, P1],
     D[dI1gdt, S2], D[dI1gdt, I2s], D[dI1gdt, D2ss], D[dI1gdt, P2],
     D[dI1gdt, I1g], D[dI1gdt, I2g],
     D[dI1gdt, C1sg], D[dI1gdt, C2sg], D[dI1gdt, Pg]},
    {D[dI2gdt, S1], D[dI2gdt, I1s], D[dI2gdt, D1ss], D[dI2gdt, P1],
     D[dI2gdt, S2], D[dI2gdt, I2s], D[dI2gdt, D2ss], D[dI2gdt, P2],
     D[dI2gdt, I1g], D[dI2gdt, I2g],
     D[dI2gdt, C1sg], D[dI2gdt, C2sg], D[dI2gdt, Pg]},
    {D[dC1sgdt, S1], D[dC1sgdt, I1s], D[dC1sgdt, D1ss], D[dC1sgdt, P1],
     D[dC1sgdt, S2], D[dC1sgdt, I2s], D[dC1sgdt, D2ss], D[dC1sgdt, P2],
     D[dC1sgdt, I1g], D[dC1sgdt, I2g],
     D[dC1sgdt, C1sg], D[dC1sgdt, C2sg], D[dC1sgdt, Pg]},
    {D[dC2sgdt, S1], D[dC2sgdt, I1s], D[dC2sgdt, D1ss], D[dC2sgdt, P1],
     D[dC2sgdt, S2], D[dC2sgdt, I2s], D[dC2sgdt, D2ss], D[dC2sgdt, P2],
     D[dC2sgdt, I1g], D[dC2sgdt, I2g],
     D[dC2sgdt, C1sg], D[dC2sgdt, C2sg], D[dC2sgdt, Pg]},
    {D[dPgdt, S1], D[dPgdt, I1s], D[dPgdt, D1ss], D[dPgdt, P1],
     D[dPgdt, S2], D[dPgdt, I2s], D[dPgdt, D2ss], D[dPgdt, P2],
     D[dPgdt, I1g], D[dPgdt, I2g], D[dPgdt, C1sg], D[dPgdt, C2sg], D[dPgdt, Pg]}} /.
   {I1g → 0, I2g → 0, C1sg → 0, C2sg → 0, Pg → 0};
(* The submatrices *)
(* J1 *)
MatrixForm[J1 = J[[1 ;; 4, 1 ;; 4]]]
```

$$\begin{pmatrix} -\frac{r1\,(D1ss+I1s+S1)}{K1}+r1\left(1-\frac{D1ss+I1s+S1}{K1}\right)-P1\,\beta S1 & -\frac{r1\,(D1ss+I1s+S1)}{K1}+r1\left(1-\frac{D1ss+I1s+S1}{K1}\right) & -\frac{r1\,(D1ss+I1s+S1)}{K1} \\ P1\,\beta S1 & -\mu 1-P1\,\beta I1\,\sigma D1 & \\ 0 & P1\,\beta I1\,\sigma D1 & \\ -P1\,\beta S1 & -P1\,\beta I1+\lambda 1 & -P \end{pmatrix}$$

```
(* Zeros *)
MatrixForm[J[[1 ;; 4, 5 ;; 8]]]
```

$$\begin{pmatrix} 0 & 0 & 0 & 0 \\ 0 & 0 & 0 & 0 \\ 0 & 0 & 0 & 0 \\ 0 & 0 & 0 & 0 \end{pmatrix}$$

```
(* M1 *)
MatrixForm[M1 = J[[1 ;; 4, 9 ;; 13]]]
```

$$\begin{pmatrix} 0 & 0 & 0 & 0 & 0 \\ 0 & 0 & 0 & 0 & -I1s\,\beta I1\,\sigma C1 \\ 0 & 0 & 0 & 0 & 0 \\ 0 & 0 & -P1\,\beta C1+x1\,\lambda 1 & 0 & 0 \end{pmatrix}$$

```
(* Zeros *)
MatrixForm[J[[5 ;; 8, 1 ;; 4]]]
```

$$\begin{pmatrix} 0 & 0 & 0 & 0 \\ 0 & 0 & 0 & 0 \\ 0 & 0 & 0 & 0 \\ 0 & 0 & 0 & 0 \end{pmatrix}$$

```
(* J2 *)
MatrixForm[J2 = J[[5 ;; 8, 5 ;; 8]]]
```

$$\begin{pmatrix} -\frac{r2\,(D2ss+I2s+S2)}{K2}+r2\left(1-\frac{D2ss+I2s+S2}{K2}\right)-P2\,\beta S2 & -\frac{r2\,(D2ss+I2s+S2)}{K2}+r2\left(1-\frac{D2ss+I2s+S2}{K2}\right) & -\frac{r2\,(D2ss+I2s+S2)}{K2} \\ P2\,\beta S2 & -\mu 2-P2\,\beta I2\,\sigma D2 & \\ 0 & P2\,\beta I2\,\sigma D2 & \\ -P2\,\beta S2 & -P2\,\beta I2+\lambda 2 & -P \end{pmatrix}$$

```
(* M2 *)
MatrixForm[M2 = J[[5 ;; 8, 9 ;; 13]]]
```

$$\begin{pmatrix} 0 & -\frac{r2\,(D2ss+I2s+S2)}{K2}+r2\left(1-\frac{D2ss+I2s+S2}{K2}\right) & 0 & -\frac{r2\,(D2ss+I2s+S2)}{K2}+r2\left(1-\frac{D2ss+I2s+S2}{K2}\right) & -S2\,\beta S2 \\ 0 & 0 & 0 & 0 & -I2s\,\beta I2\,\sigma C2 \\ 0 & 0 & 0 & 0 & 0 \\ 0 & 0 & 0 & -P2\,\beta C2+x2\,\lambda 2 & 0 \end{pmatrix}$$

```
(* Zeros *)
MatrixForm[J[[9 ;; 13, 1 ;; 4]]]
```

$$\begin{pmatrix} 0 & 0 & 0 & 0 \\ 0 & 0 & 0 & 0 \\ 0 & 0 & 0 & 0 \\ 0 & 0 & 0 & 0 \\ 0 & 0 & 0 & 0 \end{pmatrix}$$

```
(* Zeros *)
MatrixForm[J[[9 ;; 13, 5 ;; 8]]]
```

$$\begin{pmatrix} 0 & 0 & 0 & 0 \\ 0 & 0 & 0 & 0 \\ 0 & 0 & 0 & 0 \\ 0 & 0 & 0 & 0 \\ 0 & 0 & 0 & 0 \end{pmatrix}$$

```
(* Jm *)
MatrixForm[Jm = J[[9 ;; 13, 9 ;; 13]]]
```

$$\begin{pmatrix} -\mu 1 - P1\,\beta I1\,\sigma C1 & 0 & 0 & 0 & S1\,\beta S1 \\ 0 & -\mu 2 - P2\,\beta I2\,\sigma C2 & 0 & 0 & S2\,\beta S2 \\ P1\,\beta I1\,\sigma C1 & 0 & -\mu 1 & 0 & I1s\,\beta I1\,\text{[illegible]} \\ 0 & P2\,\beta I2\,\sigma C2 & 0 & -\mu 2 & I2s\,\beta I2\,\text{[illegible]} \\ a\,\lambda 1 & a\,\lambda 2 & a\,(1-x1)\,\lambda 1 & a\,(1-x2)\,\lambda 2 & -D1ss\,\beta D1 - D2ss\,\beta D2 - I1s\,\beta I1 - \text{[illegible]} \end{pmatrix}$$

The submatrix

$$J_m = \begin{pmatrix} -\mu_1 - \sigma_{C_1}\beta_{I_1}\hat{P}_1 & 0 & 0 & 0 & \beta_{S_1}\hat{S}_1 \\ 0 & -\mu_2 - \sigma_{C_2}\beta_{I_2}\hat{P}_2 & 0 & 0 & \beta_{S_2}\hat{S}_2 \\ \sigma_{C_1}\beta_{I_1}\hat{P}_1 & 0 & -\mu 1 & 0 & \sigma_{C_1}\beta_{I_1}\hat{I_{1,s}} \\ 0 & \sigma_{C_2}\beta_{I_2}\hat{P}_2 & 0 & -\mu 2 & \sigma_{C_2}\beta_{I_2}\hat{I_{2,s}} \\ a\lambda_1 & a\lambda_2 & a(1-x_1)\lambda_1 & a(1-x_2)\lambda_2 & -\beta_{S_1}\hat{S}_1 - \beta_{S_2}\hat{S}_2 - \beta_{I_1}\hat{I_{1,s}} - \beta_{I_2}\hat{I_{2,s}} - \beta_{D_1}\text{[illegible]} \end{pmatrix}$$

.

Rather than finding for the eigenvalues of this submatrix, we make use of the Next Generation Theorem and rewrite J_m as $F - V$, where $F = \begin{pmatrix} 0 & 0 & 0 & 0 & \beta_{S_1}\hat{S}_1 \\ 0 & 0 & 0 & 0 & \beta_{S_2}\hat{S}_2 \\ \sigma_{C_1}\beta_{I_1}\hat{P}_1 & 0 & 0 & 0 & \sigma_{C_1}\beta_{I_1}\hat{I_{1,s}} \\ 0 & \sigma_{C_2}\beta_{I_2}\hat{P}_2 & 0 & 0 & \sigma_{C_2}\beta_{I_2}\hat{I_{2,s}} \\ 0 & 0 & 0 & 0 & 0 \end{pmatrix}$ and

$$V = \begin{pmatrix} \mu_1 + \sigma_{C_1}\beta_{I_1}\hat{P}_1 & 0 & 0 & 0 & 0 \\ 0 & \mu_2 + \sigma_{C_2}\beta_{I_2}\hat{P}_2 & 0 & 0 & 0 \\ 0 & 0 & \mu 1 & 0 & 0 \\ 0 & 0 & 0 & \mu 2 & 0 \\ -a\lambda_1 & -a\lambda_2 & -a(1-x_1)\lambda_1 & -a(1-x_2)\lambda_2 & \beta_{S_1}\hat{S}_1 + \beta_{S_2}\hat{S}_2 + \beta_{I_1}\hat{I_{1,s}} + \beta_{I_2}\hat{I_{2,s}} + \beta_{D_1}\hat{D_{1,\text{[illegible]}}} \end{pmatrix}$$

```
F = {{0, 0, 0, 0, S1 βS1}, {0, 0, 0, 0, S2 βS2}, {P1 βI1 σC1, 0, 0, 0, I1s βI1 σC1},
   {0, P2 βI2 σC2, 0, 0, I2s βI2 σC2}, {0, 0, 0, 0, 0}};
V = {{μ1 + P1 βI1 σC1, 0, 0, 0, 0}, {0, μ2 + P2 βI2 σC2, 0, 0, 0}, {0, 0, μ1, 0, 0},
   {0, 0, 0, μ2, 0}, {-a λ1, -a λ2, -a (1 - x1) λ1, -a (1 - x2) λ2,
    D1ss βD1 + D2ss βD2 + I1s βI1 + I2s βI2 + S1 βS1 + S2 βS2 + γ}};
Jm ==
 F -
  V
```

```
True
```

The Next Generation Theorem states that, if a matrix J can be written $J = F - V$, where $F \geq 0$, $V^{-1} \geq 0$ and all of the eigenvalues of $-V$ are negative, then the dominant eigenvalue of J will be greater than zero whenever the spectral radius of $F.V^{-1} > 1$. Note that the spectral radius largest real part of all of the eigenvalues.

```
(* Verifying that all elements of V^-1≥0*)
Inverse[V] // Simplify
```

$$\left\{\left\{\frac{1}{\mu 1+\text{P1}\,\beta\text{I1}\,\sigma\text{C1}},0,0,0,0\right\},\left\{0,\frac{1}{\mu 2+\text{P2}\,\beta\text{I2}\,\sigma\text{C2}},0,0,0\right\},\left\{0,0,\frac{1}{\mu 1},0,0\right\},\left\{0,0,0,\frac{1}{\mu 2},0\right\},\right.$$

$$\{(\text{a}\,\lambda 1)/((\text{D1ss}\,\beta\text{D1}+\text{D2ss}\,\beta\text{D2}+\text{I1s}\,\beta\text{I1}+\text{I2s}\,\beta\text{I2}+\text{S1}\,\beta\text{S1}+\text{S2}\,\beta\text{S2}+\gamma)\,(\mu 1+\text{P1}\,\beta\text{I1}\,\sigma\text{C1})),$$
$$(\text{a}\,\lambda 2)/((\text{D1ss}\,\beta\text{D1}+\text{D2ss}\,\beta\text{D2}+\text{I1s}\,\beta\text{I1}+\text{I2s}\,\beta\text{I2}+\text{S1}\,\beta\text{S1}+\text{S2}\,\beta\text{S2}+\gamma)\,(\mu 2+\text{P2}\,\beta\text{I2}\,\sigma\text{C2})),$$
$$-((\text{a}\,(-1+\text{x1})\,\lambda 1)/((\text{D1ss}\,\beta\text{D1}+\text{D2ss}\,\beta\text{D2}+\text{I1s}\,\beta\text{I1}+\text{I2s}\,\beta\text{I2}+\text{S1}\,\beta\text{S1}+\text{S2}\,\beta\text{S2}+\gamma)\,\mu 1)),$$
$$-((\text{a}\,(-1+\text{x2})\,\lambda 2)/((\text{D1ss}\,\beta\text{D1}+\text{D2ss}\,\beta\text{D2}+\text{I1s}\,\beta\text{I1}+\text{I2s}\,\beta\text{I2}+\text{S1}\,\beta\text{S1}+\text{S2}\,\beta\text{S2}+\gamma)\,\mu 2)),$$
$$\left.1/(\text{D1ss}\,\beta\text{D1}+\text{D2ss}\,\beta\text{D2}+\text{I1s}\,\beta\text{I1}+\text{I2s}\,\beta\text{I2}+\text{S1}\,\beta\text{S1}+\text{S2}\,\beta\text{S2}+\gamma)\}\right\}$$

```
(* Verifying that all eigenvalues of -V<0 *)
Eigenvalues[-V] // Simplify
```

$$\{-\text{D1ss}\,\beta\text{D1}-\text{D2ss}\,\beta\text{D2}-\text{I1s}\,\beta\text{I1}-\text{I2s}\,\beta\text{I2}-\text{S1}\,\beta\text{S1}-\text{S2}\,\beta\text{S2}-\gamma,$$
$$-\mu 1,-\mu 2,-\mu 1-\text{P1}\,\beta\text{I1}\,\sigma\text{C1},-\mu 2-\text{P2}\,\beta\text{I2}\,\sigma\text{C2}\}$$

```
(* Eigenvalues of F.V^-1 *)
```

```
Eigenvalues[Dot[F, Inverse[V]]] // Simplify
```

```
{0, 0, 0,
 (a S2 βS2 λ2 μ1^2 μ2 + a S1 βS1 λ1 μ1 μ2^2 + a P1 S2 βI1 βS2 λ2 μ1 μ2 σC1 + a I1s βI1 λ1 μ1 μ2^2 σC1 -
    a I1s x1 βI1 λ1 μ1 μ2^2 σC1 + a I1s P1 βI1^2 λ1 μ2^2 σC1^2 - a I1s P1 x1 βI1^2 λ1 μ2^2 σC1^2 +
    a P2 S1 βI2 βS1 λ1 μ1 μ2 σC2 + a I2s βI2 λ2 μ1^2 μ2 σC2 - a I2s x2 βI2 λ2 μ1^2 μ2 σC2 +
    a I1s P2 βI1 βI2 λ1 μ1 μ2 σC1 σC2 - a I1s P2 x1 βI1 βI2 λ1 μ1 μ2 σC1 σC2 +
    a I2s P1 βI1 βI2 λ2 μ1 μ2 σC1 σC2 - a I2s P1 x2 βI1 βI2 λ2 μ1 μ2 σC1 σC2 +
    a I1s P1 P2 βI1^2 βI2 λ1 μ2 σC1^2 σC2 - a I1s P1 P2 x1 βI1^2 βI2 λ1 μ2 σC1^2 σC2 +
    a I2s P2 βI2^2 λ2 μ1^2 σC2^2 - a I2s P2 x2 βI2^2 λ2 μ1^2 σC2^2 +
    a I2s P1 P2 βI1 βI2^2 λ2 μ1 σC1 σC2^2 - a I2s P1 P2 x2 βI1 βI2^2 λ2 μ1 σC1 σC2^2 -
    √(a (-4 (D1ss βD1 + D2ss βD2 + I1s βI1 + I2s βI2 + S1 βS1 + S2 βS2 + γ) μ1 μ2
          (μ1 + P1 βI1 σC1) (μ2 + P2 βI2 σC2) (P2 S2 (-1 + x2) βI2 βS2 λ2 μ1^2 σC2 + P1 βI1 σC1
             (P2 S2 (-1 + x2) βI2 βS2 λ2 μ1 σC2 + S1 (-1 + x1) βS1 λ1 μ2 (μ2 + P2 βI2 σC2))) +
        a (S2 βS2 λ2 μ1 μ2 (μ1 + P1 βI1 σC1) + (μ2 + P2 βI2 σC2)
             (S1 βS1 λ1 μ1 μ2 - (μ1 + P1 βI1 σC1)
                (I1s (-1 + x1) βI1 λ1 μ2 σC1 + I2s (-1 + x2) βI2 λ2 μ1 σC2)))^2))) /
  (2 (D1ss βD1 + D2ss βD2 + I1s βI1 + I2s βI2 + S1 βS1 + S2 βS2 + γ) μ1 μ2
    (μ1 + P1 βI1 σC1) (μ2 + P2 βI2 σC2)),
 (a S2 βS2 λ2 μ1^2 μ2 + a S1 βS1 λ1 μ1 μ2^2 + a P1 S2 βI1 βS2 λ2 μ1 μ2 σC1 +
    a I1s βI1 λ1 μ1 μ2^2 σC1 - a I1s x1 βI1 λ1 μ1 μ2^2 σC1 +
    a I1s P1 βI1^2 λ1 μ2^2 σC1^2 - a I1s P1 x1 βI1^2 λ1 μ2^2 σC1^2 +
    a P2 S1 βI2 βS1 λ1 μ1 μ2 σC2 + a I2s βI2 λ2 μ1^2 μ2 σC2 -
    a I2s x2 βI2 λ2 μ1^2 μ2 σC2 + a I1s P2 βI1 βI2 λ1 μ1 μ2 σC1 σC2 -
    a I1s P2 x1 βI1 βI2 λ1 μ1 μ2 σC1 σC2 + a I2s P1 βI1 βI2 λ2 μ1 μ2 σC1 σC2 -
    a I2s P1 x2 βI1 βI2 λ2 μ1 μ2 σC1 σC2 + a I1s P1 P2 βI1^2 βI2 λ1 μ2 σC1^2 σC2 -
    a I1s P1 P2 x1 βI1^2 βI2 λ1 μ2 σC1^2 σC2 + a I2s P2 βI2^2 λ2 μ1^2 σC2^2 -
    a I2s P2 x2 βI2^2 λ2 μ1^2 σC2^2 + a I2s P1 P2 βI1 βI2^2 λ2 μ1 σC1 σC2^2 -
    a I2s P1 P2 x2 βI1 βI2^2 λ2 μ1 σC1 σC2^2 +
    √(a (-4 (D1ss βD1 + D2ss βD2 + I1s βI1 + I2s βI2 + S1 βS1 + S2 βS2 + γ) μ1 μ2
          (μ1 + P1 βI1 σC1) (μ2 + P2 βI2 σC2) (P2 S2 (-1 + x2) βI2 βS2 λ2 μ1^2 σC2 + P1 βI1 σC1
             (P2 S2 (-1 + x2) βI2 βS2 λ2 μ1 σC2 + S1 (-1 + x1) βS1 λ1 μ2 (μ2 + P2 βI2 σC2))) +
        a (S2 βS2 λ2 μ1 μ2 (μ1 + P1 βI1 σC1) + (μ2 + P2 βI2 σC2)
             (S1 βS1 λ1 μ1 μ2 - (μ1 + P1 βI1 σC1)
                (I1s (-1 + x1) βI1 λ1 μ2 σC1 + I2s (-1 + x2) βI2 λ2 μ1 σC2)))^2))) /
  (2 (D1ss βD1 + D2ss βD2 + I1s βI1 + I2s βI2 + S1 βS1 + S2 βS2 + γ) μ1 μ2
    (μ1 + P1 βI1 σC1) (μ2 + P2 βI2 σC2))}
```

The spectral bound condition is

$$R_m = \left(\left(\beta_{S_1}\hat{S}_1\right)\Big/\left(\beta_{S_1}\hat{S}_1+\beta_{S_2}\hat{S}_2+\beta_{I_1}\hat{I}_{1,s}+\beta_{I_2}\hat{I}_{2,s}+\beta_{D_1}\hat{D}_{1,s,s}+\beta_{D_2}\hat{D}_{2,s,s}+\gamma\right)\right)\left(\frac{\mu_1}{\mu_1+\sigma_{C_1}\beta_{I_1}\hat{P}_1}\frac{a\,\lambda_1}{\mu_1}+\frac{\sigma_{C_1}\beta_{I_1}\hat{P}_1}{\mu_1+\sigma_{C_1}\beta_{I_1}\hat{P}_1}\frac{a\,(1-x_1)\,\lambda_1}{\mu_1}\right)+$$
$$\left(\left(\beta_{I_1}\hat{I}_{1,s}\right)\Big/\left(\beta_{S_1}\hat{S}_1+\beta_{S_2}\hat{S}_2+\beta_{I_1}\hat{I}_{1,s}+\beta_{I_2}\hat{I}_{2,s}+\beta_{D_1}\hat{D}_{1,s,s}+\beta_{D_2}\hat{D}_{2,s,s}+\gamma\right)\right)\frac{a\,(1-x_1)\,\lambda_1}{\mu_1}+$$
$$\left(\left(\beta_{S_2}\hat{S}_2\right)\Big/\left(\beta_{S_1}\hat{S}_1+\beta_{S_2}\hat{S}_2+\beta_{I_1}\hat{I}_{1,s}+\beta_{I_2}\hat{I}_{2,s}+\beta_{D_1}\hat{D}_{1,s,s}+\beta_{D_2}\hat{D}_{2,s,s}+\gamma\right)\right)\left(\frac{\mu_2}{\mu_2+\sigma_{C_2}\beta_{I_2}\hat{P}_2}\frac{a\,\lambda_2}{\mu_2}+\frac{\sigma_{C_2}\beta_{I_2}\hat{P}_2}{\mu_2+\sigma_{C_2}\beta_{I_2}\hat{P}_2}\frac{a\,(1-x_2)\,\lambda_2}{\mu_2}\right)+$$
$$\left(\left(\beta_{I_2}\hat{I}_{2,s}\right)\Big/\left(\beta_{S_1}\hat{S}_1+\beta_{S_2}\hat{S}_2+\beta_{I_1}\hat{I}_{1,s}+\beta_{I_2}\hat{I}_{2,s}+\beta_{D_1}\hat{D}_{1,s,s}+\beta_{D_2}\hat{D}_{2,s,s}+\gamma\right)\right)\frac{a\,(1-x_2)\,\lambda_2}{\mu_2}>1.$$

The generalized R_m expression for any number of hosts (Eq. 12 in the main text) follows from this

expression.

```
(* The condition for instability of the generalist-
  free equilibrium is that the spectral bound > 1 *)
(a S2 βS2 λ2 μ1^2 μ2 + a S1 βS1 λ1 μ1 μ2^2 + a P1 S2 βI1 βS2 λ2 μ1 μ2 σC1 + a I1s βI1 λ1 μ1 μ2^2 σC1 -
    a I1s x1 βI1 λ1 μ1 μ2^2 σC1 + a I1s P1 βI1^2 λ1 μ2^2 σC1^2 - a I1s P1 x1 βI1^2 λ1 μ2^2 σC1^2 +
    a P2 S1 βI2 βS1 λ1 μ1 μ2 σC2 + a I2s βI2 λ2 μ1^2 μ2 σC2 - a I2s x2 βI2 λ2 μ1^2 μ2 σC2 +
    a I1s P2 βI1 βI2 λ1 μ1 μ2 σC1 σC2 - a I1s P2 x1 βI1 βI2 λ1 μ1 μ2 σC1 σC2 +
    a I2s P1 βI1 βI2 λ2 μ1 μ2 σC1 σC2 - a I2s P1 x2 βI1 βI2 λ2 μ1 μ2 σC1 σC2 +
    a I1s P1 P2 βI1^2 βI2 λ1 μ2 σC1^2 σC2 - a I1s P1 P2 x1 βI1^2 βI2 λ1 μ2 σC1^2 σC2 +
    a I2s P2 βI2^2 λ2 μ1^2 σC2^2 - a I2s P2 x2 βI2^2 λ2 μ1^2 σC2^2 +
    a I2s P1 P2 βI1 βI2^2 λ2 μ1 σC1 σC2^2 - a I2s P1 P2 x2 βI1 βI2^2 λ2 μ1 σC1 σC2^2 -
    √(a (-4 (D1ss βD1 + D2ss βD2 + I1s βI1 + I2s βI2 + S1 βS1 + S2 βS2 + γ)
          μ1 μ2 (μ1 + P1 βI1 σC1) (μ2 + P2 βI2 σC2)
          (P2 S2 (-1 + x2) βI2 βS2 λ2 μ1^2 σC2 + P1 βI1 σC1 (P2 S2 (-1 + x2) βI2 βS2 λ2 μ1
              σC2 + S1 (-1 + x1) βS1 λ1 μ2 (μ2 + P2 βI2 σC2))) + a (S2 βS2 λ2 μ1 μ2
            (μ1 + P1 βI1 σC1) + (μ2 + P2 βI2 σC2) (S1 βS1 λ1 μ1 μ2 - (μ1 + P1 βI1 σC1)
              (I1s (-1 + x1) βI1 λ1 μ2 σC1 + I2s (-1 + x2) βI2 λ2 μ1 σC2)))^2))) /
  (2 (D1ss βD1 + D2ss βD2 + I1s βI1 + I2s βI2 + S1 βS1 + S2 βS2 + γ) μ1 μ2
    (μ1 + P1 βI1 σC1) (μ2 + P2 βI2 σC2)) > 1;
(* Cross-multiplying *)
(a S2 βS2 λ2 μ1^2 μ2 + a S1 βS1 λ1 μ1 μ2^2 + a P1 S2 βI1 βS2 λ2 μ1 μ2 σC1 + a I1s βI1 λ1 μ1 μ2^2 σC1 -
   a I1s x1 βI1 λ1 μ1 μ2^2 σC1 + a I1s P1 βI1^2 λ1 μ2^2 σC1^2 - a I1s P1 x1 βI1^2 λ1 μ2^2 σC1^2 +
   a P2 S1 βI2 βS1 λ1 μ1 μ2 σC2 + a I2s βI2 λ2 μ1^2 μ2 σC2 - a I2s x2 βI2 λ2 μ1^2 μ2 σC2 +
   a I1s P2 βI1 βI2 λ1 μ1 μ2 σC1 σC2 - a I1s P2 x1 βI1 βI2 λ1 μ1 μ2 σC1 σC2 +
   a I2s P1 βI1 βI2 λ2 μ1 μ2 σC1 σC2 - a I2s P1 x2 βI1 βI2 λ2 μ1 μ2 σC1 σC2 +
   a I1s P1 P2 βI1^2 βI2 λ1 μ2 σC1^2 σC2 - a I1s P1 P2 x1 βI1^2 βI2 λ1 μ2 σC1^2 σC2 +
   a I2s P2 βI2^2 λ2 μ1^2 σC2^2 - a I2s P2 x2 βI2^2 λ2 μ1^2 σC2^2 +
   a I2s P1 P2 βI1 βI2^2 λ2 μ1 σC1 σC2^2 - a I2s P1 P2 x2 βI1 βI2^2 λ2 μ1 σC1 σC2^2 -
   √(a (-4 (D1ss βD1 + D2ss βD2 + I1s βI1 + I2s βI2 + S1 βS1 + S2 βS2 + γ) μ1 μ2
         (μ1 + P1 βI1 σC1) (μ2 + P2 βI2 σC2) (P2 S2 (-1 + x2) βI2 βS2 λ2 μ1^2 σC2 + P1 βI1 σC1
           (P2 S2 (-1 + x2) βI2 βS2 λ2 μ1 σC2 + S1 (-1 + x1) βS1 λ1 μ2 (μ2 + P2 βI2 σC2))) +
       a (S2 βS2 λ2 μ1 μ2 (μ1 + P1 βI1 σC1) + (μ2 + P2 βI2 σC2) (S1 βS1 λ1 μ1 μ2 -
             (μ1 + P1 βI1 σC1) (I1s (-1 + x1) βI1 λ1 μ2 σC1 +
                I2s (-1 + x2) βI2 λ2 μ1 σC2)))^2))) >
  (2 (D1ss βD1 + D2ss βD2 + I1s βI1 + I2s βI2 + S1 βS1 + S2 βS2 + γ) μ1 μ2
    (μ1 + P1 βI1 σC1) (μ2 + P2 βI2 σC2));

(* Isolating the square root term *)
-√(a (-4 (D1ss βD1 + D2ss βD2 + I1s βI1 + I2s βI2 + S1 βS1 + S2 βS2 + γ) μ1 μ2 (μ1 + P1 βI1 σC1)
        (μ2 + P2 βI2 σC2) (P2 S2 (-1 + x2) βI2 βS2 λ2 μ1^2 σC2 + P1 βI1 σC1
          (P2 S2 (-1 + x2) βI2 βS2 λ2 μ1 σC2 + S1 (-1 + x1) βS1 λ1 μ2 (μ2 + P2 βI2 σC2))) +
      a (S2 βS2 λ2 μ1 μ2 (μ1 + P1 βI1 σC1) + (μ2 + P2 βI2 σC2)
          (S1 βS1 λ1 μ1 μ2 - (μ1 + P1 βI1 σC1)
             (I1s (-1 + x1) βI1 λ1 μ2 σC1 + I2s (-1 + x2) βI2 λ2 μ1 σC2)))^2)) >
  (2 (D1ss βD1 + D2ss βD2 + I1s βI1 + I2s βI2 + S1 βS1 + S2 βS2 + γ)
     μ1
     μ2
     (μ1 + P1 βI1 σC1)
     (μ2 + P2 βI2 σC2)) -
   (a S2 βS2 λ2 μ1^2 μ2 + a S1 βS1 λ1 μ1 μ2^2 + a P1 S2 βI1 βS2 λ2 μ1 μ2 σC1 +
     a I1s βI1 λ1 μ1 μ2^2 σC1 -
     a I1s x1 βI1 λ1 μ1 μ2^2 σC1 +
```

```
a I1s P1 βI1^2 λ1 μ2^2 σC1^2 - a I1s P1 x1 βI1^2 λ1 μ2^2 σC1^2 +
a P2 S1 βI2 βS1 λ1 μ1 μ2 σC2 + a I2s βI2 λ2 μ1^2 μ2 σC2 -
a I2s x2 βI2 λ2 μ1^2 μ2 σC2 + a I1s P2 βI1 βI2 λ1 μ1 μ2 σC1 σC2 -
a I1s P2 x1 βI1 βI2 λ1 μ1 μ2 σC1 σC2 +
a I2s P1 βI1 βI2 λ2 μ1 μ2 σC1 σC2 -
a I2s P1 x2 βI1 βI2 λ2 μ1 μ2 σC1 σC2 +
a I1s P1 P2 βI1^2 βI2 λ1 μ2 σC1^2 σC2 -
a I1s P1 P2 x1 βI1^2 βI2 λ1 μ2 σC1^2 σC2 + a I2s P2 βI2^2 λ2 μ1^2 σC2^2 -
a I2s P2 x2 βI2^2 λ2 μ1^2 σC2^2 + a I2s P1 P2 βI1 βI2^2 λ2 μ1 σC1 σC2^2 -
a I2s P1 P2 x2 βI1 βI2^2 λ2 μ1 σC1 σC2^2);

(* Squaring both sides and simplifying, the condition becomes: *)
(-√(a (-4 (D1ss βD1 + D2ss βD2 + I1s βI1 + I2s βI2 + S1 βS1 + S2 βS2 + γ)
        μ1 μ2 (μ1 + P1 βI1 σC1) (μ2 + P2 βI2 σC2)
        (P2 S2 (-1 + x2) βI2 βS2 λ2 μ1^2 σC2 + P1 βI1 σC1 (P2 S2 (-1 + x2) βI2 βS2 λ2 μ1
              σC2 + S1 (-1 + x1) βS1 λ1 μ2 (μ2 + P2 βI2 σC2))) + a (S2 βS2 λ2 μ1 μ2
            (μ1 + P1 βI1 σC1) + (μ2 + P2 βI2 σC2) (S1 βS1 λ1 μ1 μ2 - (μ1 + P1 βI1 σC1)
              (I1s (-1 + x1) βI1 λ1 μ2 σC1 + I2s (-1 + x2) βI2 λ2 μ1 σC2)))^2)))^2 >
  ((2 (D1ss βD1 + D2ss βD2 + I1s βI1 + I2s βI2 + S1 βS1 + S2 βS2 + γ) μ1 μ2
       (μ1 + P1 βI1 σC1) (μ2 + P2 βI2 σC2)) -
     (a S2 βS2 λ2 μ1^2 μ2 + a S1 βS1 λ1 μ1 μ2^2 + a P1 S2 βI1 βS2 λ2 μ1 μ2 σC1 +
       a I1s βI1 λ1 μ1 μ2^2 σC1 - a I1s x1 βI1 λ1 μ1 μ2^2 σC1 +
       a I1s P1 βI1^2 λ1 μ2^2 σC1^2 - a I1s P1 x1 βI1^2 λ1 μ2^2 σC1^2 +
       a P2 S1 βI2 βS1 λ1 μ1 μ2 σC2 + a I2s βI2 λ2 μ1^2 μ2 σC2 -
       a I2s x2 βI2 λ2 μ1^2 μ2 σC2 + a I1s P2 βI1 βI2 λ1 μ1 μ2 σC1 σC2 -
       a I1s P2 x1 βI1 βI2 λ1 μ1 μ2 σC1 σC2 + a I2s P1 βI1 βI2 λ2 μ1 μ2 σC1 σC2 -
       a I2s P1 x2 βI1 βI2 λ2 μ1 μ2 σC1 σC2 + a I1s P1 P2 βI1^2 βI2 λ1 μ2 σC1^2 σC2 -
       a I1s P1 P2 x1 βI1^2 βI2 λ1 μ2 σC1^2 σC2 + a I2s P2 βI2^2 λ2 μ1^2 σC2^2 -
       a I2s P2 x2 βI2^2 λ2 μ1^2 σC2^2 + a I2s P1 P2 βI1 βI2^2 λ2 μ1 σC1 σC2^2 -
       a I2s P1 P2 x2 βI1 βI2^2 λ2 μ1 σC1 σC2^2))^2 // Simplify
```

```
(D1ss βD1 + D2ss βD2 + I1s βI1 + I2s βI2 + S1 βS1 + S2 βS2 + γ)
  μ1 μ2 (μ1 + P1 βI1 σC1) (μ2 + P2 βI2 σC2)
  ((D1ss βD1 + D2ss βD2 + I1s βI1 + I2s βI2 + S1 βS1 + S2 βS2 + γ) μ1 μ2 (μ1 + P1 βI1 σC1)
      (μ2 + P2 βI2 σC2) - a (S2 βS2 λ2 μ1 (μ1 + P1 βI1 σC1) (μ2 - P2 (-1 + x2) βI2 σC2) +
        (μ2 + P2 βI2 σC2) (S1 βS1 λ1 μ2 (μ1 - P1 (-1 + x1) βI1 σC1) -
            (μ1 + P1 βI1 σC1) (I1s (-1 + x1) βI1 λ1 μ2 σC1 + I2s (-1 + x2) βI2 λ2 μ1 σC2)))) < 0
```

```
(* Dividing the positive coefficient, the condition becomes *)
(D1ss βD1 + D2ss βD2 + I1s βI1 + I2s βI2 + S1 βS1 + S2 βS2 + γ) μ1 μ2 (μ1 + P1 βI1 σC1)
     (μ2 + P2 βI2 σC2) - a (S2 βS2 λ2 μ1 (μ1 + P1 βI1 σC1) (μ2 - P2 (-1 + x2) βI2 σC2) +
       (μ2 + P2 βI2 σC2) (S1 βS1 λ1 μ2 (μ1 - P1 (-1 + x1) βI1 σC1) -
          (μ1 + P1 βI1 σC1) (I1s (-1 + x1) βI1 λ1 μ2 σC1 + I2s (-1 + x2) βI2 λ2 μ1 σC2))) < 0;
(* Simplifying, the condition becomes *)
(D1ss βD1 + D2ss βD2 + I1s βI1 + I2s βI2 + S1 βS1 + S2 βS2 + γ) μ1 μ2 (μ1 + P1 βI1 σC1)
    (μ2 + P2 βI2 σC2) < a (S2 βS2 λ2 μ1 (μ1 + P1 βI1 σC1) (μ2 - P2 (-1 + x2) βI2 σC2) +
      (μ2 + P2 βI2 σC2) (S1 βS1 λ1 μ2 (μ1 - P1 (-1 + x1) βI1 σC1) -
         (μ1 + P1 βI1 σC1) (I1s (-1 + x1) βI1 λ1 μ2 σC1 + I2s (-1 + x2) βI2 λ2 μ1 σC2)));
(* Dividing through, the condition becomes *)
(1 / ((D1ss βD1 + D2ss βD2 + I1s βI1 + I2s βI2 + S1 βS1 + S2 βS2 + γ)
        μ1 μ2 (μ1 + P1 βI1 σC1) (μ2 + P2 βI2 σC2)))
    (a (S2 βS2 λ2 μ1 (μ1 + P1 βI1 σC1) (μ2 - P2 (-1 + x2) βI2 σC2) +
        (μ2 + P2 βI2 σC2) (S1 βS1 λ1 μ2 (μ1 - P1 (-1 + x1) βI1 σC1) - (μ1 + P1 βI1 σC1)
           (I1s (-1 + x1) βI1 λ1 μ2 σC1 + I2s (-1 + x2) βI2 λ2 μ1 σC2)))) > 1;
(* This expression is equivalent to *)
Rm = ((S1 βS1) / (D1ss βD1 + D2ss βD2 + I1s βI1 + I2s βI2 + S1 βS1 + S2 βS2 + γ) )
     (μ1/(μ1 + P1 βI1 σC1) a λ1/μ1 + (P1 βI1 σC1)/(μ1 + P1 βI1 σC1) (a (1 - x1) λ1)/μ1) +
    ((I1s βI1 σC1) / (D1ss βD1 + D2ss βD2 + I1s βI1 + I2s βI2 + S1 βS1 + S2 βS2 + γ) )
     (a (1 - x1) λ1)/μ1 + ((S2 βS2) / (D1ss βD1 + D2ss βD2 + I1s βI1 + I2s βI2 + S1 βS1 + S2 βS2 + γ) )
     (μ2/(μ2 + P2 βI2 σC2) a λ2/μ2 + (P2 βI2 σC2)/(μ2 + P2 βI2 σC2) (a (1 - x2) λ2)/μ2) +
    ((I2s βI2 σC2) / (D1ss βD1 + D2ss βD2 + I1s βI1 + I2s βI2 + S1 βS1 + S2 βS2 + γ) )
     (a (1 - x2) λ2)/μ2;
Rm ==
   (1 / ((D1ss βD1 + D2ss βD2 + I1s βI1 + I2s βI2 + S1 βS1 + S2 βS2 + γ)
        μ1 μ2 (μ1 + P1 βI1 σC1) (μ2 + P2 βI2 σC2)))
    (a (S2 βS2 λ2 μ1 (μ1 + P1 βI1 σC1) (μ2 - P2 (-1 + x2) βI2 σC2) +
        (μ2 + P2 βI2 σC2) (S1 βS1 λ1 μ2 (μ1 - P1 (-1 + x1) βI1 σC1) - (μ1 + P1 βI1 σC1)
           (I1s (-1 + x1) βI1 λ1 μ2 σC1 + I2s (-1 + x2) βI2 λ2 μ1 σC2)))) // Simplify

True
```

Calculating the response of R_m for Cases 1-6 in Table 2

Case 1: One specialist parasite; no coinfection; parasite regulation of host population size; avoidance of non-susceptible hosts

Case 2: Two specialist parasites; no coinfection; parasite regulation of host population size;avoidance of non-susceptible hosts

Case 3: Two specialist parasites; no coinfection; parasite regulation of host population size; no avoidance of non-susceptible hosts

Case 4: One specialist parasite; coinfection; parasite regulation of host

population size; avoidance of non-susceptible hosts

Based on the parameters presented in Table 1 in the main text, the R_m expression will simplify to the expression

$R_m =$

$$\frac{\beta_{S_1}\hat{S}_1}{\beta_{S_1}\hat{S}_1+\beta_{S_2}\hat{S}_2+\beta_{I_1}\hat{I_{1,s}}+\gamma}\left(\frac{\mu_1}{\mu_1+\beta_{I_1}\hat{P}_1}\frac{a\,\lambda_1}{\mu_1}+\frac{\beta_{I_1}\hat{P}_1}{\mu_1+\beta_{I_1}\hat{P}_1}\frac{a\,(1-x_1)\,\lambda_1}{\mu_1}\right)+\frac{\beta_{I_1}\hat{I_{1,s}}}{\beta_{S_1}\hat{S}_1+\beta_{S_2}\hat{S}_2+\beta_{I_1}\hat{I_{1,s}}+\gamma}\frac{a\,(1-x_1)\,\lambda_1}{\mu_1}+\frac{\beta_{S_2}\hat{S}_2}{\beta_{S_1}\hat{S}_1+\beta_{S_2}\hat{S}_2+\beta_{I_1}\hat{I_{1,s}}+\gamma}\left(\frac{a\,\lambda_2}{\mu_2}\right).$$

The parasite can invade if $R_m > 1$.

Unfortunately, it is analytically intractable to determine the sign of $\frac{\partial R_m}{\partial W}$ or $\frac{\partial R_m}{\partial T}$, so we use numerical exploration to determine the effect of host body size and environmental temperature on the R_m.

```
(* Rm at the parameters for this case from Table 1 *)
Rm /. {σC1 → 1, βD1 → 0, D2ss → 0, I2s → 0, P2 → 0}
```

$$\frac{a\,\text{I1s}\,(1-\text{x1})\,\beta\text{I1}\,\lambda1}{(\text{I1s}\,\beta\text{I1}+\text{S1}\,\beta\text{S1}+\text{S2}\,\beta\text{S2}+\gamma)\,\mu1}+\frac{\text{S1}\,\beta\text{S1}\left(\frac{a\,\lambda1}{\text{P1}\,\beta\text{I1}+\mu1}+\frac{a\,\text{P1}\,(1-\text{x1})\,\beta\text{I1}\,\lambda1}{\mu1\,(\text{P1}\,\beta\text{I1}+\mu1)}\right)}{\text{I1s}\,\beta\text{I1}+\text{S1}\,\beta\text{S1}+\text{S2}\,\beta\text{S2}+\gamma}+\frac{a\,\text{S2}\,\beta\text{S2}\,\lambda2}{(\text{I1s}\,\beta\text{I1}+\text{S1}\,\beta\text{S1}+\text{S2}\,\beta\text{S2}+\gamma)\,\mu2}$$

Endoparasites:

Many of the parameters of the model are set by allometric relationships and have been established previously (Gillooly et al. 2001, Allen et al. 2002, Savage et al. 2004, Poulin & George-Nascimento 2007). Values for \.08\.08E, k, r_0, K_0, and μ_0 that are appropriate for fish come from Gillooly et al. 2001 and Savage et al. 2004. The estimate of λ_0 is taken from Poulin & George-Nascimento 2007.

$$\text{allom} = \left\{\text{K1} \to \text{K0 Exp}\left[\frac{E}{k\,T}\right] W^{-3/4},\ \text{K2} \to \text{K0 Exp}\left[\frac{E}{k\,T}\right] (f\,W)^{-3/4},\ \mu1 \to \mu0\,\text{Exp}\left[-\frac{E}{k\,T}\right] W^{-1/4},\ \mu2 \to \mu0\,\text{Exp}\left[-\frac{E}{k\,T}\right] (f\,W)^{-1/4},\ \lambda1 \to \lambda0\,\text{Exp}\left[-\frac{E}{k\,T}\right] W^{3/4},\ \lambda2 \to \lambda0\,\text{Exp}\left[-\frac{E}{k\,T}\right] (f\,W)^{3/4},\ \text{r1} \to \text{r0 Exp}\left[-\frac{E}{k\,T}\right] W^{-1/4},\ \text{r2} \to \text{r0 Exp}\left[-\frac{E}{k\,T}\right] (f\,W)^{-1/4}\right\};$$

$$\text{allompars} = \left\{E \to 0.43,\ k \to \frac{8.617`}{10^5},\ \text{K0} \to \frac{2.984`}{10^9},\ \mu0 \to 1.785` \times 10^8,\ \lambda0 \to 2 \times 10^8,\ \text{r0} \to 2.21 \times 10^{10}\right\};$$

That leaves a much smaller subset of parameters that can be varied. For simplicity, we focus on parameters whose effects on invasion fitness are complex. This excludes parameters related to the costs associated with generalism, including a (the reduction in shedding rate for generalists), σ_{C_1} (the probability of coinfection, which we hold constant at 1), and x_1 (the fraction of host resources captured by the resident strains in coinfection, which we hold constant at 1/2). None of these parameters affect the equilibrium abundances of susceptible and singly infected hosts, so their effect on R_m are predictable and obvious - reducing a, reducing σ_{C_1}, or increasing x_1 will all reduce R_m, making invasion by the generalist more difficult. Thus the only parameters to explore (other than host mass W and temperature T), are the contact rates between hosts and parasites and γ (the loss rate of parasites from the environment). For simplicity, we assume that all contact rates are equal ($\beta_{S_1} = \beta_{I_1} = \beta_{S_2} = \beta$).

We can solve for the equilibria analytically, although the expression for $\hat{P}_1$ cannot be expressed simply.

```
(* Solving for S₁ in terms of I₁,s and P₁*)
S1Eq = Solve[(dI1sdt /. {σC1 → 1, σD1 → 1, Pg → 0}) == 0, S1];
(* Solving for I₁,s in terms of D₁,s,s and P₁ *)
I1sEq = Solve[(dD1ssdt /. σD1 → 1) == 0, I1s];
(* Solving for D₁,s,s in terms of P₁ *)
D1ssEq = Simplify[
  Solve[(dP1dt /. {βC1 → 0, βD1 → 0, C1sg → 0} /. S1Eq[[1]] /. I1sEq[[1]]) == 0, D1ss]]
(* Solving for I₁,s in terms of P₁ *)
I1sEq = Simplify[I1sEq /. D1ssEq[[1]]]
(* Solving for S₁ in terms of P₁ *)
S1Eq = Simplify[S1Eq /. I1sEq[[1]]]
(* Solving for P₁ *)
P1Eq = Solve[Simplify[dS1dt /. {C1sg → 0, I1g → 0, Pg → 0} /. S1Eq[[1]] /. I1sEq[[1]] /.
      D1ssEq[[1]]] == 0, P1];
```

$$\left\{\left\{D1ss \to \frac{P1^2\,\beta I1\,\gamma}{P1\,\beta I1\,(\lambda 1 - 2\,\mu 1) + (\lambda 1 - \mu 1)\,\mu 1}\right\}\right\}$$

$$\left\{\left\{I1s \to \frac{P1\,\gamma\,\mu 1}{P1\,\beta I1\,(\lambda 1 - 2\,\mu 1) + (\lambda 1 - \mu 1)\,\mu 1}\right\}\right\}$$

$$\{\{S1 \to (\gamma\,\mu 1\,(P1\,\beta I1 + \mu 1)) / (\beta S1\,(P1\,\beta I1\,(\lambda 1 - 2\,\mu 1) + (\lambda 1 - \mu 1)\,\mu 1))\}\}$$

With these equilibria, we can compute the value of R_m, varying host body size W and the contact rate β.

```
(* Rm, plugging in the parameter values from Table 1,
the equilibria calculated above, the allometric scaling relationships,
and the parameters of the allometric functions *)
RmV = Rm /. S1Eq[[1]] /. I1sEq[[1]] /. D1ssEq[[1]] /. P1Eq[[2]] /. S2 → K2 /.
     {σC1 → 1, βD1 → 0, D2ss → 0, x1 → 1 / 2, I2s → 0, P2 → 0,
      βS1 → β, βI1 → β, βS2 → β} /. allom /. allompars;
(* Compute Rm for a range of W and β values *)
RmAcrossWB = Table[Table[RmV /. {β → B, γ → 0.1, W → Wval, T → 270, a → 0.8, f → 0.8},
    {Wval, 10, 1010, 100}], {B, 1, 10, 1}];
```

Increasing host body size increases R_m, regardless of the value of β, thereby making it easier for the generalist to invade. This can be seen in Figs. S1-S4 below.

```
Wvals = Table[W, {W, 10, 1010, 100}];
Labeled[
 ListLinePlot[Table[{Wvals[[i]], Re[RmAcrossWB[[1, i]]]}, {i, 1, Length[Wvals]}],
  PlotLabel → "Fig. S1. Effect of body size W on Rm when β = 1"],
 {"Host mass W", "Generalist Rm"}, {Bottom, Left}, RotateLabel → True]
Labeled[ListLinePlot[
  Table[{Wvals[[i]], Re[RmAcrossWB[[3, i]]]}, {i, 1, Length[Wvals]}],
  PlotLabel → "Fig. S2. Effect of body size W on Rm when β = 3"],
 {"Host mass W", "Generalist Rm"}, {Bottom, Left}, RotateLabel → True]
Labeled[ListLinePlot[
  Table[{Wvals[[i]], Re[RmAcrossWB[[5, i]]]}, {i, 1, Length[Wvals]}],
  PlotLabel → "Fig. S3. Effect of body size W on Rm when β = 5"],
 {"Host mass W", "Generalist Rm"}, {Bottom, Left}, RotateLabel → True]
Labeled[ListLinePlot[
  Table[{Wvals[[i]], Re[RmAcrossWB[[10, i]]]}, {i, 1, Length[Wvals]}],
  PlotLabel → "Fig. S4. Effect of body size W on Rm when β = 10"],
 {"Host mass W", "Generalist Rm"}, {Bottom, Left}, RotateLabel → True]
```

Fig. S1. Effect of body size W on R_m when $\beta = 1$

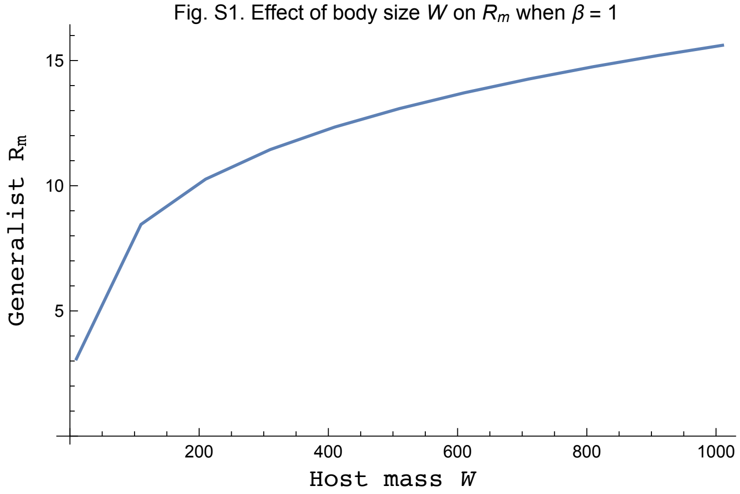

Fig. S2. Effect of body size W on R_m when $\beta = 3$

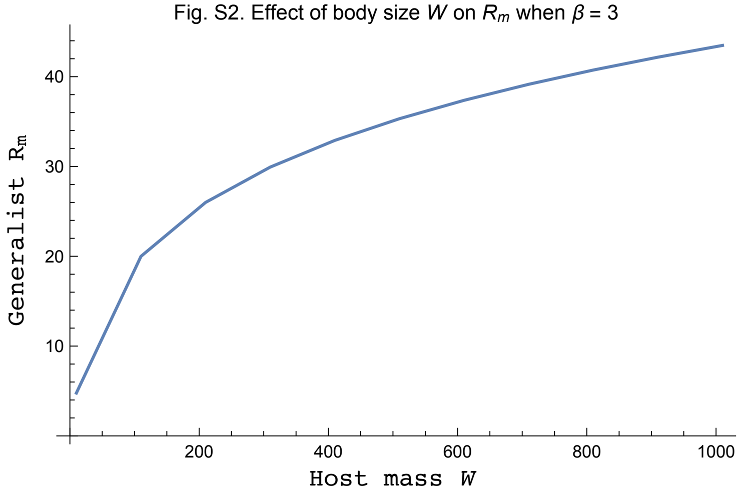

Fig. S3. Effect of body size W on R_m when $\beta = 5$

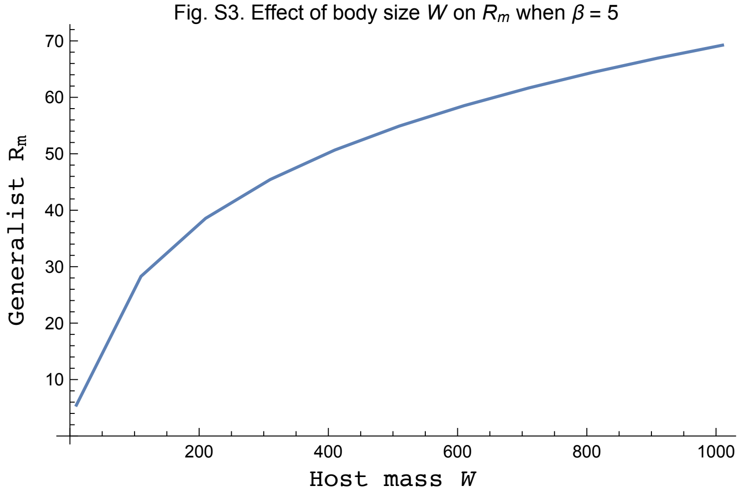

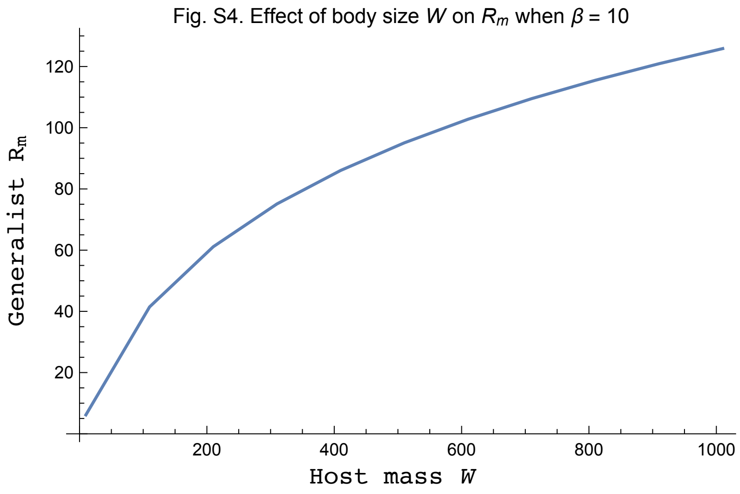

We can also compute the value of R_m, varying host body size W and the parasite loss rate from the environment γ.

```
(* Compute Rm for a range of W and γ values *)
RmAcrossWg = Table[Table[RmV /. {β → 1, γ → g, W → Wval, T → 270, a → 0.8, f → 0.8},
    {Wval, 10, 1010, 100}], {g, 0.01, 1.01, 0.1}];
```

Increasing host body size increases R_m, regardless of the value of γ, thereby making it easier for the generalist to invade. This can been seen in Figs. S5-S8 below.

```
Wvals = Table[W, {W, 10, 1010, 100}];
Labeled[
 ListLinePlot[Table[{Wvals[[i]], Re[RmAcrossWg[[1, i]]]}, {i, 1, Length[Wvals]}],
  PlotLabel → "Fig. S5 Effect of body size W on Rm when γ = 0.01"],
 {"Host mass W", "Generalist Rm"}, {Bottom, Left}, RotateLabel → True]
Labeled[ListLinePlot[
  Table[{Wvals[[i]], Re[RmAcrossWg[[3, i]]]}, {i, 1, Length[Wvals]}],
  PlotLabel → "Fig. S6. Effect of body size W on Rm when γ = 0.21"],
 {"Host mass W", "Generalist Rm"}, {Bottom, Left}, RotateLabel → True]
Labeled[ListLinePlot[
  Table[{Wvals[[i]], Re[RmAcrossWg[[6, i]]]}, {i, 1, Length[Wvals]}],
  PlotLabel → "Fig. S7. Effect of body size W on Rm when γ = 0.51"],
 {"Host mass W", "Generalist Rm"}, {Bottom, Left}, RotateLabel → True]
Labeled[ListLinePlot[
  Table[{Wvals[[i]], Re[RmAcrossWg[[11, i]]]}, {i, 1, Length[Wvals]}],
  PlotLabel → "Fig. S8. Effect of body size W on Rm when γ = 1.01"],
 {"Host mass W", "Generalist Rm"}, {Bottom, Left}, RotateLabel → True]
```

Fig. S5 Effect of body size W on R_m when γ = 0.01

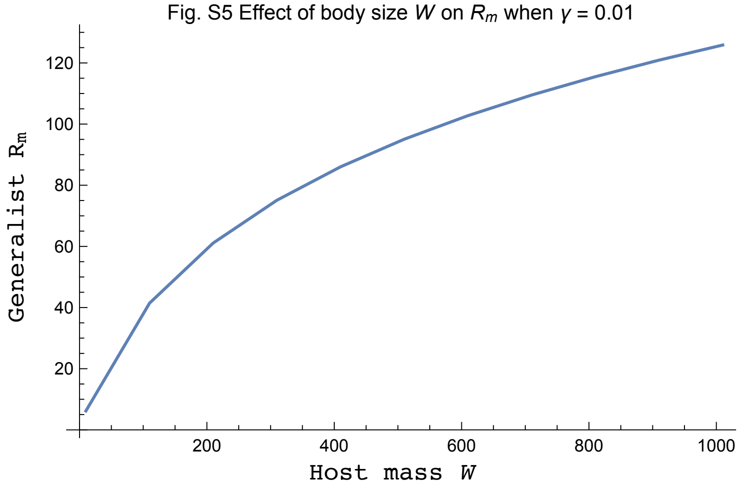

Fig. S6. Effect of body size W on R_m when γ = 0.21

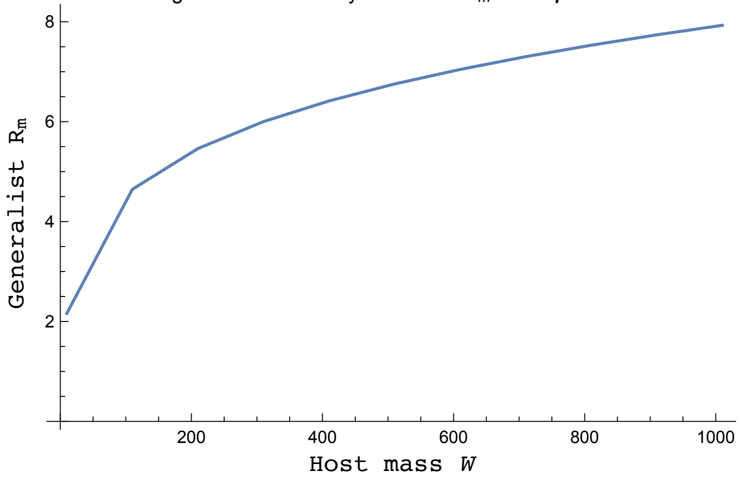

Fig. S7. Effect of body size W on R_m when γ = 0.51

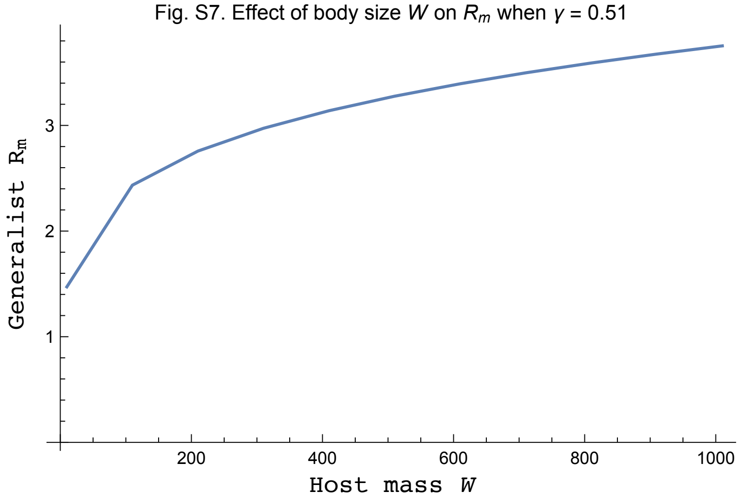

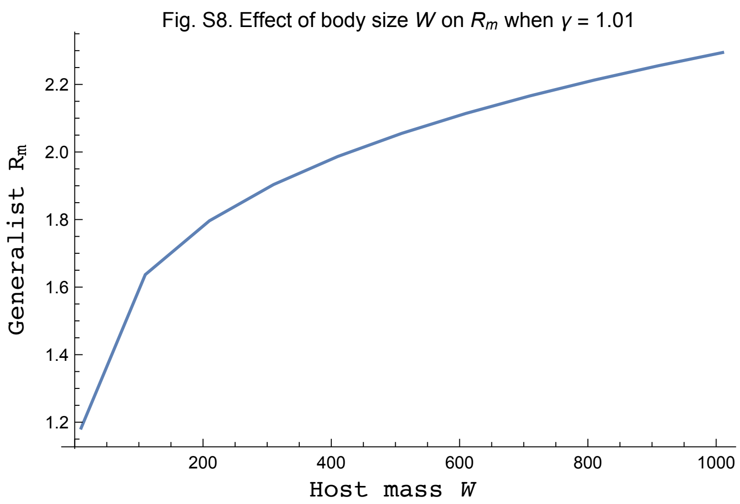

We can also compute the value of R_m, varying host body size W and the temperature T.

```
(* Compute Rm for a range of W and T values *)
RmAcrossWT = Table[Table[RmV /. {β → 1, γ → 0.2, W → Wval, T → Tval, a → 0.8, f → 0.8},
    {Tval, 270, 310, 2}], {Wval, 10, 1010, 100}];
```

Increasing temperature decreases R_m, regardless of host mass, thus making it more difficult for the generalist endoparasite to invade. This can be seen in Figs. S9-S12 below.

```
Tvals = Table[T, {T, 270, 310, 2}];
Labeled[
 ListLinePlot[Table[{Tvals[[i]], Re[RmAcrossWT[[1, i]]]}, {i, 1, Length[Tvals]}],
  PlotLabel → "Fig. S9. Effect of temperature when W = 10"],
 {"Temperature", "Generalist Rm"}, {Bottom, Left}, RotateLabel → True]
Labeled[ListLinePlot[
  Table[{Tvals[[i]], Re[RmAcrossWT[[2, i]]]}, {i, 1, Length[Tvals]}],
  PlotLabel → "Fig. S10. Effect of temperature when W = 110"],
 {"Temperature", "Generalist Rm"}, {Bottom, Left}, RotateLabel → True]
Labeled[ListLinePlot[
  Table[{Tvals[[i]], Re[RmAcrossWT[[6, i]]]}, {i, 1, Length[Tvals]}],
  PlotLabel → "Fig. S11. Effect of temperature when W = 510"],
 {"Temperature", "Generalist Rm"}, {Bottom, Left}, RotateLabel → True]
Labeled[ListLinePlot[
  Table[{Tvals[[i]], Re[RmAcrossWT[[11, i]]]}, {i, 1, Length[Tvals]}],
  PlotLabel → "Fig. S12. Effect of temperature when W = 1010"],
 {"Temperature", "Generalist Rm"}, {Bottom, Left}, RotateLabel → True]
```

Fig. S9. Effect of temperature when W = 10

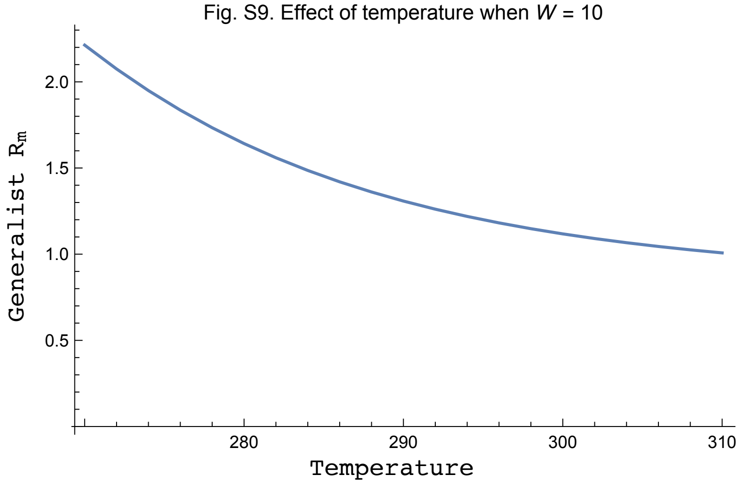

Fig. S10. Effect of temperature when W = 110

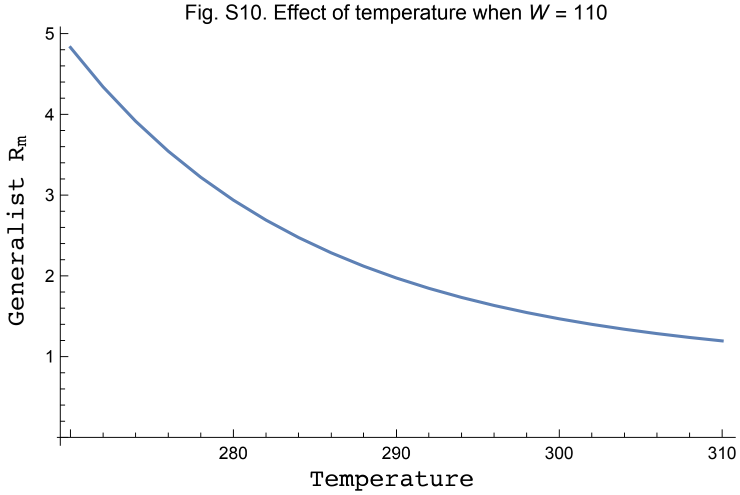

Fig. S11. Effect of temperature when W = 510

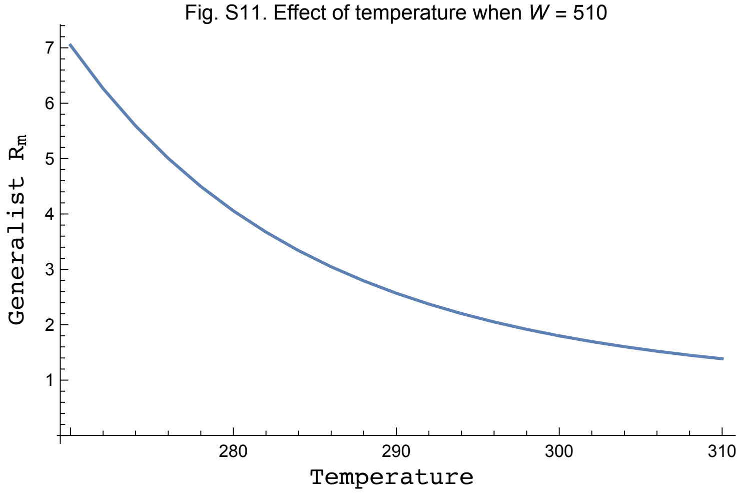

Fig. S12. Effect of temperature when W = 1010

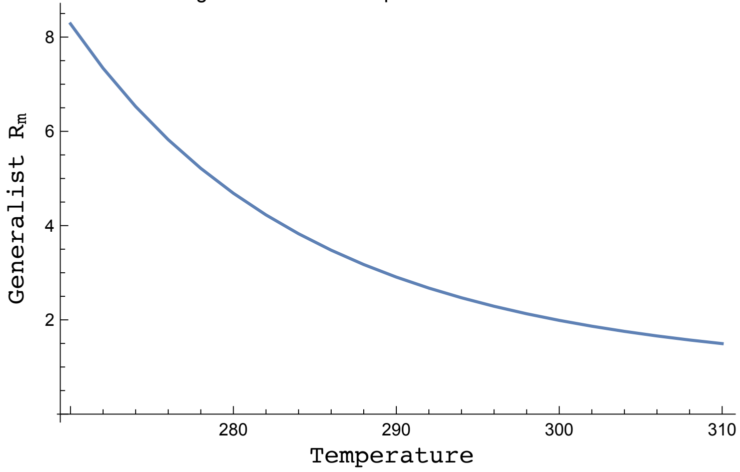

Ectoparasites:

The only change from the endoparasite case is with the scaling of λ:

```
allom = {K1 → K0 Exp[E/(k T)] W^(-3/4), K2 → K0 Exp[E/(k T)] (f W)^(-3/4),
   μ1 → μ0 Exp[-E/(k T)] W^(-1/4), μ2 → μ0 Exp[-E/(k T)] (f W)^(-1/4), λ1 → λ0 Exp[-E/(k T)] W^(5/12),
   λ2 → λ0 Exp[-E/(k T)] (f W)^(5/12), r1 → r0 Exp[-E/(k T)] W^(-1/4), r2 → r0 Exp[-E/(k T)] (f W)^(-1/4)};
allompars = {E → 0.43, k → 8.617`/10^5, K0 → 2.984`/10^9,
   μ0 → 1.785` × 10^8, λ0 → 2 × 10^8, r0 → 2.21 × 10^10};
```

We compute the value of R_m, varying host body size W and the contact rate β.

```
(* Rm, plugging in the parameter values from Table 1,
the equilibria calculated above, the allometric scaling relationships,
and the parameters of the allometric functions *)
RmV = Rm /. S1Eq[[1]] /. I1sEq[[1]] /. D1ssEq[[1]] /. P1Eq[[2]] /. S2 → K2 /.
      {σC1 → 1, βD1 → 0, D2ss → 0, x1 → 1 / 2, I2s → 0, P2 → 0,
       βS1 → β, βI1 → β, βS2 → β} /. allom /. allompars;
(* Compute Rm for a range of W and β values *)
RmAcrossWB = Table[Table[RmV /. {β → B, γ → 0.1, W → Wval, T → 270, a → 0.8, f → 0.8},
     {Wval, 10, 1010, 100}], {B, 1, 10, 1}];
```

The relationship between host body size and R_m depends on the value of β. For very low β, the generalist cannot invade. For values of β large enough to permit the generalist to invade, increasing host body size first increases, then decreases, R_m. Note that this is the same response as was the case for the model without coinfection. This can be seen in Figs. S13-S16 below.

```
Wvals = Table[W, {W, 10, 1010, 100}];
Labeled[
 ListLinePlot[Table[{Wvals[[i]], Re[RmAcrossWB[[1, i]]]}, {i, 1, Length[Wvals]}],
  PlotLabel → "Fig. S13. Effect of body size W on Rm when β = 1"],
 {"Host mass W", "Generalist Rm"}, {Bottom, Left}, RotateLabel → True]
Labeled[ListLinePlot[
  Table[{Wvals[[i]], Re[RmAcrossWB[[3, i]]]}, {i, 1, Length[Wvals]}],
  PlotLabel → "Fig. S14. Effect of body size W on Rm when β = 3"],
 {"Host mass W", "Generalist Rm"}, {Bottom, Left}, RotateLabel → True]
Labeled[ListLinePlot[
  Table[{Wvals[[i]], Re[RmAcrossWB[[5, i]]]}, {i, 1, Length[Wvals]}],
  PlotLabel → "Fig. S15. Effect of body size W on Rm when β = 5"],
 {"Host mass W", "Generalist Rm"}, {Bottom, Left}, RotateLabel → True]
Labeled[ListLinePlot[
  Table[{Wvals[[i]], Re[RmAcrossWB[[10, i]]]}, {i, 1, Length[Wvals]}],
  PlotLabel → "Fig. S16. Effect of body size W on Rm when β = 10"],
 {"Host mass W", "Generalist Rm"}, {Bottom, Left}, RotateLabel → True]
```

Fig. S13. Effect of body size W on R_m when $\beta = 1$

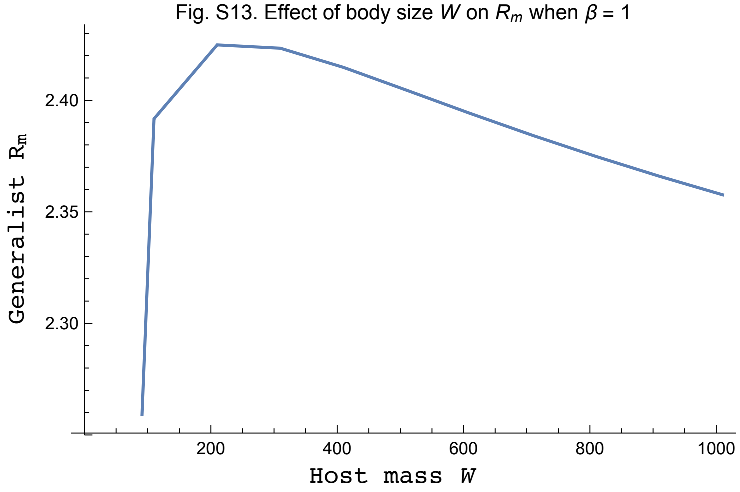

Fig. S14. Effect of body size W on R_m when $\beta = 3$

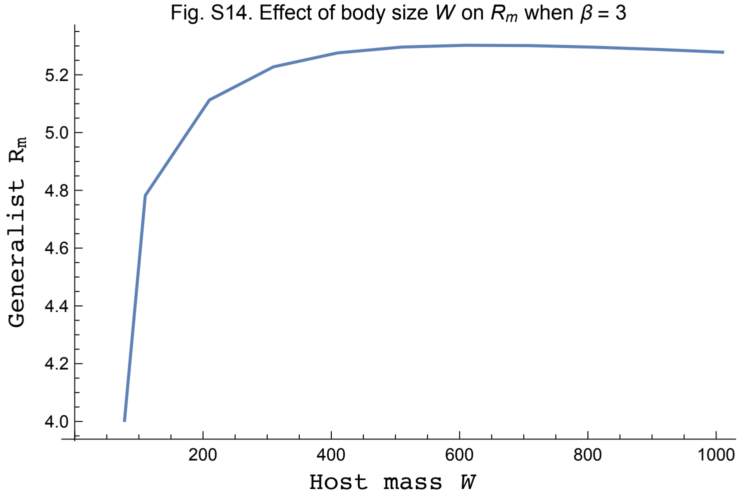

Fig. S15. Effect of body size W on R_m when $\beta = 5$

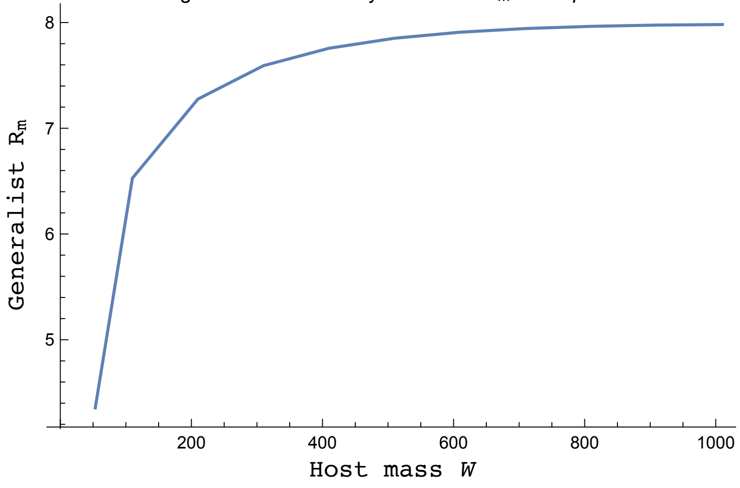

Fig. S16. Effect of body size W on R_m when $\beta = 10$

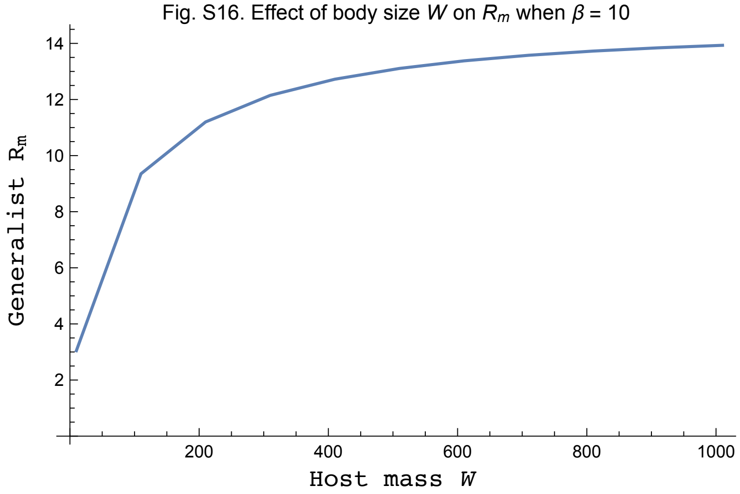

We can also compute the value of R_m, varying host body size W and the temperature T to determine the effect of temperature.

```
(* Compute Rm for a range of W and T values *)
RmAcrossWT = Table[Table[RmV /. {β → 0.5, γ → 0.1, W → Wval, T → Tval, a → 0.8, f → 0.8},
    {Tval, 270, 310, 2}], {Wval, 10, 1010, 100}];
```

Increasing temperature decreases R_m, regardless of host mass, thus making it more difficult for the generalist endoparasite to invade. This can be seen in Figs. S17-S20 below.

```
Tvals = Table[T, {T, 270, 310, 2}];
Labeled[
 ListLinePlot[Table[{Tvals[[i]], Re[RmAcrossWT[[1, i]]]}, {i, 1, Length[Tvals]}],
  PlotLabel → "Fig. S17. Effect of temperature when W = 10"],
 {"Temperature", "Generalist Rm"}, {Bottom, Left}, RotateLabel → True]
Labeled[ListLinePlot[
  Table[{Tvals[[i]], Re[RmAcrossWT[[2, i]]]}, {i, 1, Length[Tvals]}],
  PlotLabel → "Fig. S18. Effect of temperature when W = 110"],
 {"Temperature", "Generalist Rm"}, {Bottom, Left}, RotateLabel → True]
Labeled[ListLinePlot[
  Table[{Tvals[[i]], Re[RmAcrossWT[[6, i]]]}, {i, 1, Length[Tvals]}],
  PlotLabel → "Fig. S19. Effect of temperature when W = 510"],
 {"Temperature", "Generalist Rm"}, {Bottom, Left}, RotateLabel → True]
Labeled[ListLinePlot[
  Table[{Tvals[[i]], Re[RmAcrossWT[[11, i]]]}, {i, 1, Length[Tvals]}],
  PlotLabel → "Fig. S20. Effect of temperature when W = 1010"],
 {"Temperature", "Generalist Rm"}, {Bottom, Left}, RotateLabel → True]
```

Fig. S17. Effect of temperature when W = 10

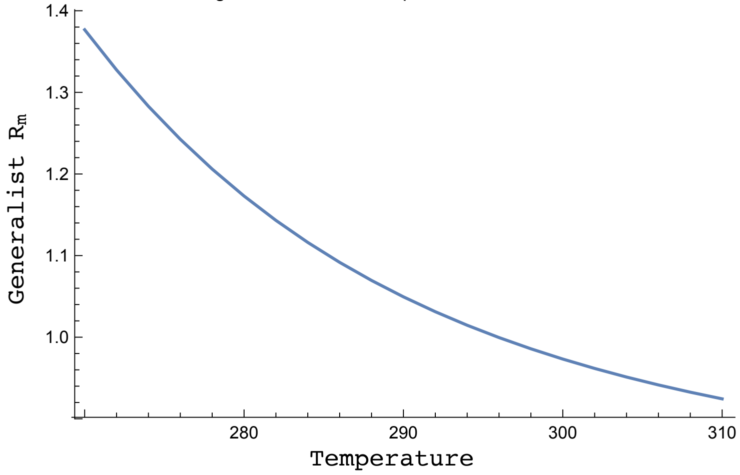

Fig. S18. Effect of temperature when W = 110

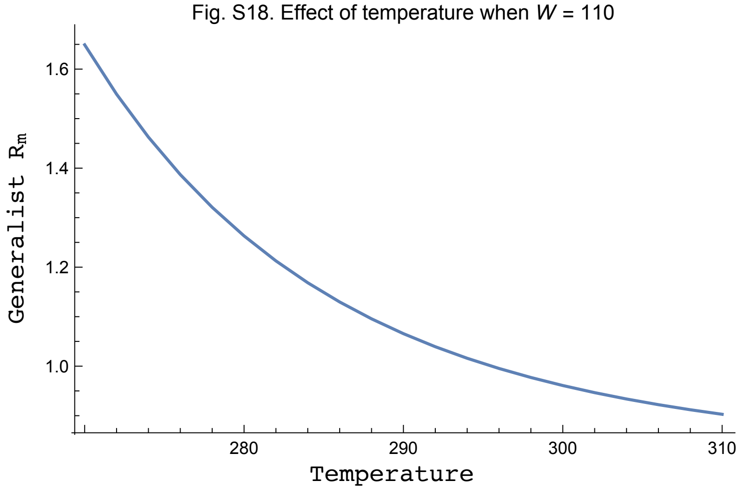

Fig. S19. Effect of temperature when W = 510

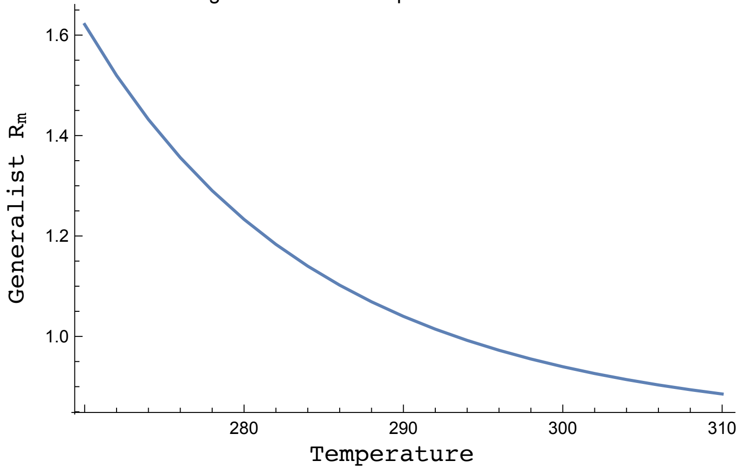

Fig. S20. Effect of temperature when W = 1010

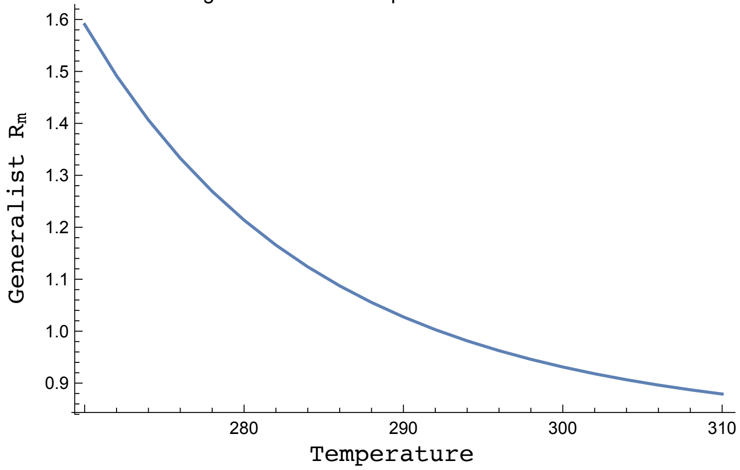

Case 5: Two specialist parasites, coinfection, parasite regulation of host population size; avoidance of non-susceptible hosts

Based on the parameters presented in Table 1 in the main text, the R_m expression will is nearly identical to Eqn. 12 in the main text:

$$R_m = \frac{\beta_{S_1}\hat{S}_1}{\beta_{S_1}\hat{S}_1+\beta_{S_2}\hat{S}_2+\beta_{I_1}\hat{I}_{1,s}+\beta_{I_2}\hat{I}_{2,s}+\gamma}\left(\frac{\mu_1}{\mu_1+\beta_{I_1}\hat{P}_1}\,\frac{a\,\lambda_1}{\mu_1}+\frac{\beta_{I_1}\hat{P}_1}{\mu_1+\beta_{I_1}\hat{P}_1}\,\frac{a\,(1-x_1)\,\lambda_1}{\mu_1}\right)+\frac{\beta_{I_1}\hat{I}_{1,s}}{\beta_{S_1}\hat{S}_1+\beta_{S_2}\hat{S}_2+\beta_{I_1}\hat{I}_{1,s}+\beta_{I_2}\hat{I}_{2,s}+\gamma}\,\frac{a\,(1-x_1)\,\lambda_1}{\mu_1}+ \frac{\beta_{S_2}\hat{S}_2}{\beta_{S_1}\hat{S}_1+\beta_{S_2}\hat{S}_2+\beta_{I_1}\hat{I}_{1,s}+\beta_{I_2}\hat{I}_{2,s}+\gamma}\left(\frac{\mu_2}{\mu_2+\beta_{I_2}\hat{P}_2}\,\frac{a\,\lambda_2}{\mu_2}+\frac{\beta_{I_2}\hat{P}_2}{\mu_2+\beta_{I_2}\hat{P}_2}\,\frac{a\,(1-x_2)\,\lambda_2}{\mu_2}\right)+\frac{\beta_{I_2}\hat{I}_{2,s}}{\beta_{S_1}\hat{S}_1+\beta_{S_2}\hat{S}_2+\beta_{I_1}\hat{I}_{1,s}+\beta_{I_2}\hat{I}_{2,s}+\gamma}\,\frac{a\,(1-x_2)\,\lambda_2}{\mu_2} > 1.$$

The generalized R_m expression for any number of hosts (Eq. 12 in the main text) follows from this expression.

Based on the parameters presented in Table 1 in the main text, the R_m expression will simplify to the expression

$$R_m = \frac{\beta_{S_1}\hat{S}_1}{\beta_{S_1}\hat{S}_1+\beta_{S_2}\hat{S}_2+\beta_{I_1}\hat{I}_{1,s}+\gamma}\left(\frac{\mu_1}{\mu_1+\beta_{I_1}\hat{P}_1}\,\frac{a\,\lambda_1}{\mu_1}+\frac{\beta_{I_1}\hat{P}_1}{\mu_1+\beta_{I_1}\hat{P}_1}\,\frac{a\,(1-x_1)\,\lambda_1}{\mu_1}\right)+\frac{\beta_{I_1}\hat{I}_{1,s}}{\beta_{S_1}\hat{S}_1+\beta_{S_2}\hat{S}_2+\beta_{I_1}\hat{I}_{1,s}+\gamma}\,\frac{a\,(1-x_1)\,\lambda_1}{\mu_1}+\frac{\beta_{S_2}\hat{S}_2}{\beta_{S_1}\hat{S}_1+\beta_{S_2}\hat{S}_2+\beta_{I_1}\hat{I}_{1,s}+\gamma}\left(\frac{a\,\lambda_2}{\mu_2}\right).$$

The parasite can invade if $R_m > 1$.

Unfortunately, it is analytically intractable to determine the sign of $\frac{\partial R_m}{\partial W}$ or $\frac{\partial R_m}{\partial T}$, so we use numerical exploration to determine the effect of host body size and environmental temperature on the R_m.

```
(* Rm at the parameters for this case from Table 1 *)
Rm /. {βD1 → 0, βD2 → 0, σC1 → 1, σC2 → 1}
```

$$(a\,I1s\,(1-x1)\,\beta I1\,\lambda 1)\,/\,((I1s\,\beta I1+I2s\,\beta I2+S1\,\beta S1+S2\,\beta S2+\gamma)\,\mu 1)+\left(S1\,\beta S1\left(\frac{a\,\lambda 1}{P1\,\beta I1+\mu 1}+\frac{a\,P1\,(1-x1)\,\beta I1\,\lambda 1}{\mu 1\,(P1\,\beta I1+\mu 1)}\right)\right)\Big/(I1s\,\beta I1+I2s\,\beta I2+S1\,\beta S1+S2\,\beta S2+\gamma)+(a\,I2s\,(1-x2)\,\beta I2\,\lambda 2)\,/\,((I1s\,\beta I1+I2s\,\beta I2+S1\,\beta S1+S2\,\beta S2+\gamma)\,\mu 2)+\left(S2\,\beta S2\left(\frac{a\,\lambda 2}{P2\,\beta I2+\mu 2}+\frac{a\,P2\,(1-x2)\,\beta I2\,\lambda 2}{\mu 2\,(P2\,\beta I2+\mu 2)}\right)\right)\Big/(I1s\,\beta I1+I2s\,\beta I2+S1\,\beta S1+S2\,\beta S2+\gamma)$$

Endoparasites:

Many of the parameters of the model are set by allometric relationships and have been established previously (Gillooly et al. 2001, Allen et al. 2002, Savage et al. 2004, Poulin & George-Nascimento 2007).

$$\text{allom} = \left\{K1 \to K0\,\text{Exp}\left[\frac{E}{k\,T}\right] W^{-3/4},\ K2 \to K0\,\text{Exp}\left[\frac{E}{k\,T}\right] (f\,W)^{-3/4},\ \mu 1 \to \mu 0\,\text{Exp}\left[-\frac{E}{k\,T}\right] W^{-1/4},\ \mu 2 \to \mu 0\,\text{Exp}\left[-\frac{E}{k\,T}\right] (f\,W)^{-1/4},\ \lambda 1 \to \lambda 0\,\text{Exp}\left[-\frac{E}{k\,T}\right] W^{3/4},\ \lambda 2 \to \lambda 0\,\text{Exp}\left[-\frac{E}{k\,T}\right] (f\,W)^{3/4},\ r1 \to r0\,\text{Exp}\left[-\frac{E}{k\,T}\right] W^{-1/4},\ r2 \to r0\,\text{Exp}\left[-\frac{E}{k\,T}\right] (f\,W)^{-1/4}\right\};$$

$$\text{allompars} = \left\{E \to 0.43,\ k \to \frac{8.617`}{10^5},\ K0 \to \frac{2.984`}{10^9},\ \mu 0 \to 1.785` \times 10^8,\ \lambda 0 \to 2 \times 10^8,\ r0 \to 2.21 \times 10^{10}\right\};$$

That leaves a much smaller subset of parameters that can be varied. For simplicity, we focus on parameters whose effects on invasion fitness are complex. This excludes parameters related to the costs associated with generalism, including a (the reduction in shedding rate for generalists), σ_{C_1} (the probability of coinfection, which we hold constant at 1), and x_1 (the fraction of host resources captured by the resident strains in coinfection, which we hold constant at 1/2). None of these parameters affect the equilibrium abundances of susceptible and singly infected hosts, so their effect on R_m are predictable and obvious - reducing a, reducing σ_{C_1}, or increasing x_1 will all reduce R_m, making invasion by the generalist more difficult. Thus the only parameters to explore (other than host mass W and temperature T), are the contact rates between hosts and parasites and γ (the loss rate of parasites from the environment). For simplicity, we assume that all contact rates are equal ($\beta_{S_1} = \beta_{I_1} = \beta_{S_2} = \beta$).

We can solve for the equilibria analytically, although the expression for $\hat{P}_1$ and $\hat{P}_2$cannot be expressed simply.

```
(* Solving for S₁ in terms of I₁,s and P₁*)
S1Eq = Solve[(dI1sdt /. {σC1 → 1, σD1 → 1, Pg → 0}) == 0, S1];
(* Solving for I₁,s in terms of D₁,s,s and P₁ *)
I1sEq = Solve[(dD1ssdt /. σD1 → 1) == 0, I1s];
(* Solving for D₁,s,s in terms of P₁ *)
D1ssEq = Simplify[
  Solve[(dP1dt /. {βC1 → 0, βD1 → 0, C1sg → 0} /. S1Eq[[1]] /. I1sEq[[1]]) == 0, D1ss]]
(* Solving for I₁,s in terms of P₁ *)
I1sEq = Simplify[I1sEq /. D1ssEq[[1]]]
(* Solving for S₁ in terms of P₁ *)
S1Eq = Simplify[S1Eq /. I1sEq[[1]]]
(* Solving for P₁ *)
P1Eq = Solve[Simplify[dS1dt /. {C1sg → 0, I1g → 0, Pg → 0} /. S1Eq[[1]] /. I1sEq[[1]] /.
      D1ssEq[[1]]] == 0, P1];
(* Solving for S₂ in terms of I₂,s and P₂*)
S2Eq = Solve[(dI2sdt /. {σC2 → 1, σD2 → 1, Pg → 0}) == 0, S2];
(* Solving for I₂,s in terms of D₂,s,s and P₂ *)
I2sEq = Solve[(dD2ssdt /. σD2 → 1) == 0, I2s];
(* Solving for D₂,s,s in terms of P₂ *)
D2ssEq = Simplify[
  Solve[(dP2dt /. {βC2 → 0, βD2 → 0, C2sg → 0} /. S2Eq[[1]] /. I2sEq[[1]]) == 0, D2ss]]
(* Solving for I₂,s in terms of P₂ *)
I2sEq = Simplify[I2sEq /. D2ssEq[[1]]]
(* Solving for S₂ in terms of P₂ *)
S2Eq = Simplify[S2Eq /. I2sEq[[1]]]
(* Solving for P₂ *)
P2Eq = Solve[Simplify[dS2dt /. {C2sg → 0, I2g → 0, Pg → 0} /. S2Eq[[1]] /. I2sEq[[1]] /.
      D2ssEq[[1]]] == 0, P2];
```

$$\left\{\left\{\text{D1ss} \to \frac{\text{P1}^2\,\beta\text{I1}\,\gamma}{\text{P1}\,\beta\text{I1}\,(\lambda 1 - 2\,\mu 1) + (\lambda 1 - \mu 1)\,\mu 1}\right\}\right\}$$

$$\left\{\left\{\text{I1s} \to \frac{\text{P1}\,\gamma\,\mu 1}{\text{P1}\,\beta\text{I1}\,(\lambda 1 - 2\,\mu 1) + (\lambda 1 - \mu 1)\,\mu 1}\right\}\right\}$$

$$\{\{\text{S1} \to (\gamma\,\mu 1\,(\text{P1}\,\beta\text{I1} + \mu 1))\,/\,(\beta\text{S1}\,(\text{P1}\,\beta\text{I1}\,(\lambda 1 - 2\,\mu 1) + (\lambda 1 - \mu 1)\,\mu 1))\}\}$$

$$\left\{\left\{\text{D2ss} \to \frac{\text{P2}^2\,\beta\text{I2}\,\gamma}{\text{P2}\,\beta\text{I2}\,(\lambda 2 - 2\,\mu 2) + (\lambda 2 - \mu 2)\,\mu 2}\right\}\right\}$$

$$\left\{\left\{\text{I2s} \to \frac{\text{P2}\,\gamma\,\mu 2}{\text{P2}\,\beta\text{I2}\,(\lambda 2 - 2\,\mu 2) + (\lambda 2 - \mu 2)\,\mu 2}\right\}\right\}$$

$$\{\{\text{S2} \to (\gamma\,\mu 2\,(\text{P2}\,\beta\text{I2} + \mu 2))\,/\,(\beta\text{S2}\,(\text{P2}\,\beta\text{I2}\,(\lambda 2 - 2\,\mu 2) + (\lambda 2 - \mu 2)\,\mu 2))\}\}$$

With these equilibria, we can compute the value of R_m, varying host body size W and the contact rate β.

```
(* Rm, plugging in the parameter values from Table 1,
the equilibria calculated above, the allometric scaling relationships,
and the parameters of the allometric functions *)
RmV = Rm /. S1Eq[[1]] /. I1sEq[[1]] /. D1ssEq[[1]] /. P1Eq[[2]] /. S2Eq[[1]] /.
       I2sEq[[1]] /. D2ssEq[[1]] /. P2Eq[[2]] /. {σC1 → 1, σC2 → 1, βD1 → 0, βD2 → 0,
      x1 → 1 / 2, x2 → 1 / 2, βS1 → β, βI1 → β, βS2 → β, βI2 → β} /. allom /. allompars;
(* Compute Rm for a range of W and β values *)
RmAcrossWB = Table[Table[RmV /. {β → B, γ → 0.1, W → Wval, T → 270, a → 0.8, f → 0.8},
    {Wval, 10, 1010, 100}], {B, 1, 10, 1}];
```

Increasing host body size increases R_m, regardless of the value of β, thereby making it easier for the generalist to invade. This can be seen in Figs. S21-S24 below.

```
Wvals = Table[W, {W, 10, 1010, 100}];
Labeled[
 ListLinePlot[Table[{Wvals[[i]], Re[RmAcrossWB[[1, i]]]}, {i, 1, Length[Wvals]}],
  PlotLabel → "Fig. S21. Effect of body size W on Rm when β = 1"],
 {"Host mass W", "Generalist Rm"}, {Bottom, Left}, RotateLabel → True]
Labeled[ListLinePlot[
  Table[{Wvals[[i]], Re[RmAcrossWB[[3, i]]]}, {i, 1, Length[Wvals]}],
  PlotLabel → "Fig. S22. Effect of body size W on Rm when β = 3"],
 {"Host mass W", "Generalist Rm"}, {Bottom, Left}, RotateLabel → True]
Labeled[ListLinePlot[
  Table[{Wvals[[i]], Re[RmAcrossWB[[5, i]]]}, {i, 1, Length[Wvals]}],
  PlotLabel → "Fig. S23. Effect of body size W on Rm when β = 5"],
 {"Host mass W", "Generalist Rm"}, {Bottom, Left}, RotateLabel → True]
Labeled[ListLinePlot[
  Table[{Wvals[[i]], Re[RmAcrossWB[[10, i]]]}, {i, 1, Length[Wvals]}],
  PlotLabel → "Fig. S24. Effect of body size W on Rm when β = 10"],
 {"Host mass W", "Generalist Rm"}, {Bottom, Left}, RotateLabel → True]
```

Fig. S21. Effect of body size W on R_m when $\beta = 1$

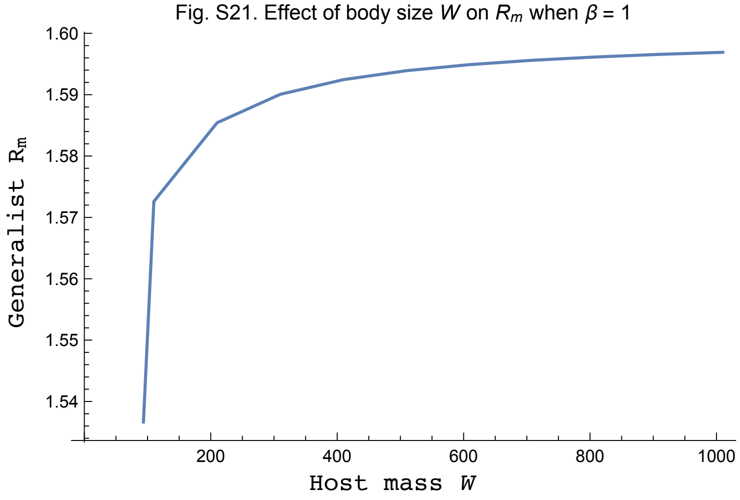

Fig. S22. Effect of body size W on R_m when $\beta = 3$

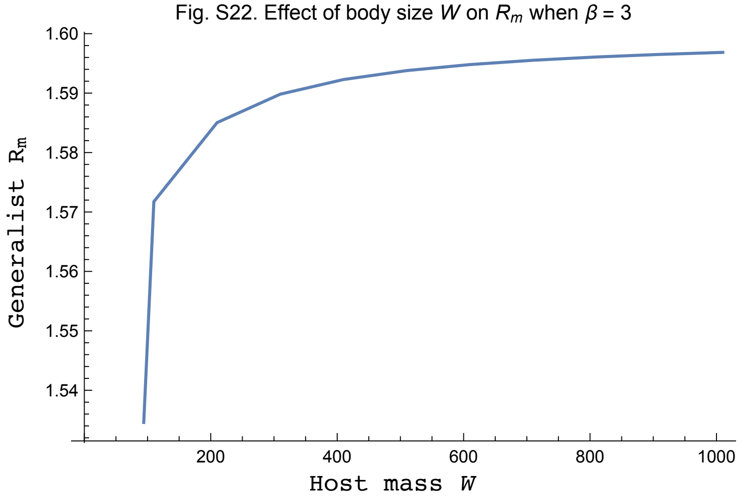

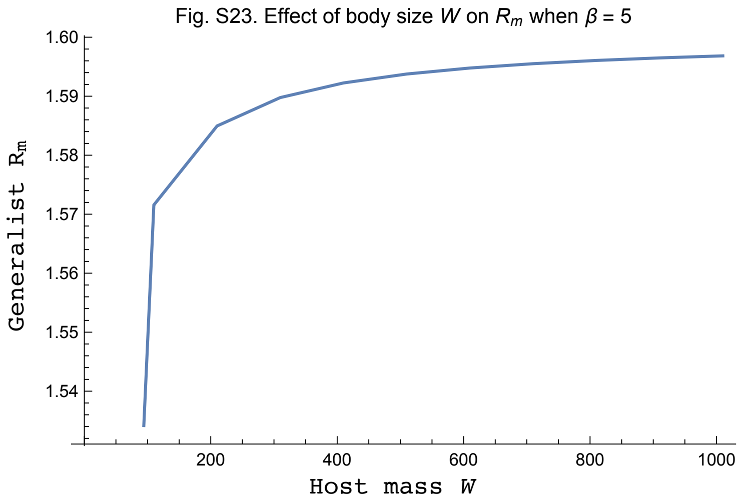

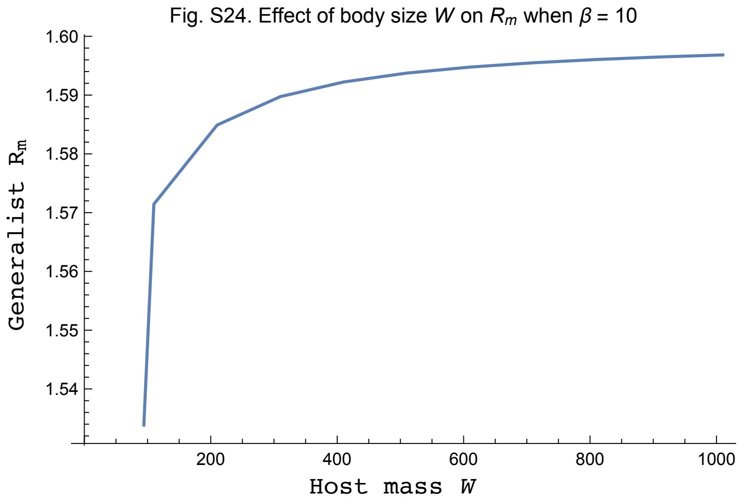

We can also compute the value of R_m, varying host body size W and the parasite loss rate from the environment γ.

```
(* Compute Rm for a range of W and γ values *)
RmAcrossWg = Table[Table[RmV /. {β → 1, γ → g, W → Wval, T → 270, a → 0.8, f → 0.8},
    {Wval, 10, 1010, 100}], {g, 0.01, 1.01, 0.1}];
```

Increasing host body size increases R_m, regardless of the value of γ, thereby making it easier for the generalist to invade. This can been seen in Figs. S25-S28 below.

```
Wvals = Table[W, {W, 10, 1010, 100}];
Labeled[
 ListLinePlot[Table[{Wvals[[i]], Re[RmAcrossWg[[1, i]]]}, {i, 1, Length[Wvals]}],
  PlotLabel → "Fig. S25 Effect of body size W on Rm when γ = 0.01"],
 {"Host mass W", "Generalist Rm"}, {Bottom, Left}, RotateLabel → True]
Labeled[ListLinePlot[
  Table[{Wvals[[i]], Re[RmAcrossWg[[3, i]]]}, {i, 1, Length[Wvals]}],
  PlotLabel → "Fig. S26. Effect of body size W on Rm when γ = 0.21"],
 {"Host mass W", "Generalist Rm"}, {Bottom, Left}, RotateLabel → True]
Labeled[ListLinePlot[
  Table[{Wvals[[i]], Re[RmAcrossWg[[6, i]]]}, {i, 1, Length[Wvals]}],
  PlotLabel → "Fig. S27. Effect of body size W on Rm when γ = 0.51"],
 {"Host mass W", "Generalist Rm"}, {Bottom, Left}, RotateLabel → True]
Labeled[ListLinePlot[
  Table[{Wvals[[i]], Re[RmAcrossWg[[11, i]]]}, {i, 1, Length[Wvals]}],
  PlotLabel → "Fig. S28. Effect of body size W on Rm when γ = 1.01"],
 {"Host mass W", "Generalist Rm"}, {Bottom, Left}, RotateLabel → True]
```

Fig. S25 Effect of body size W on R_m when γ = 0.01

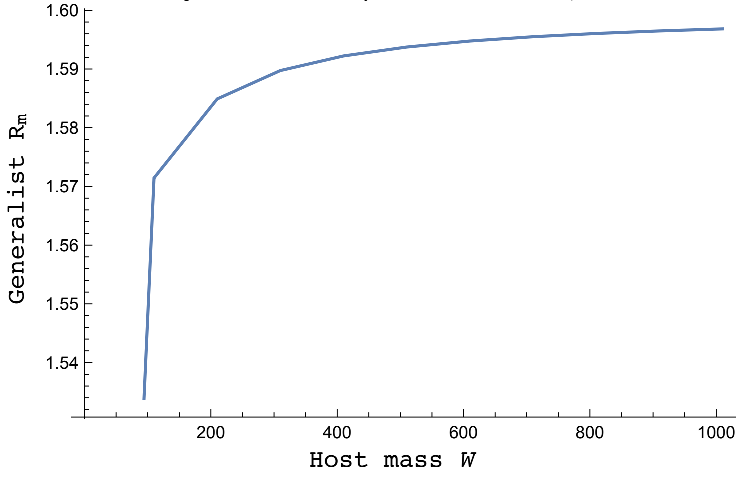

Fig. S26. Effect of body size W on R_m when γ = 0.21

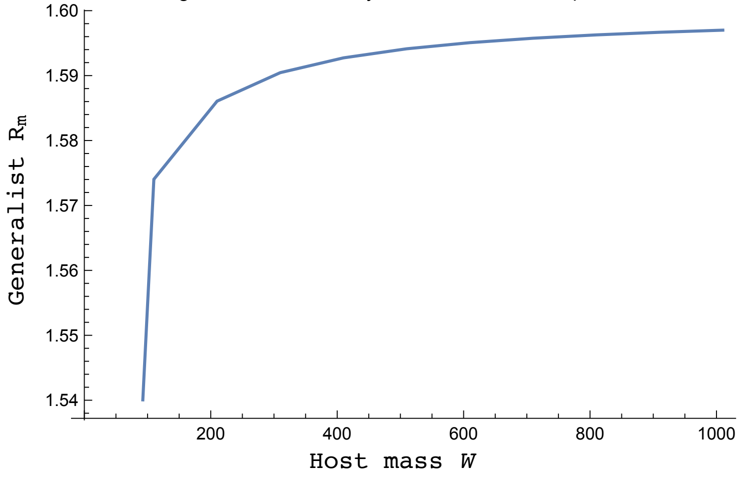

Fig. S27. Effect of body size W on R_m when γ = 0.51

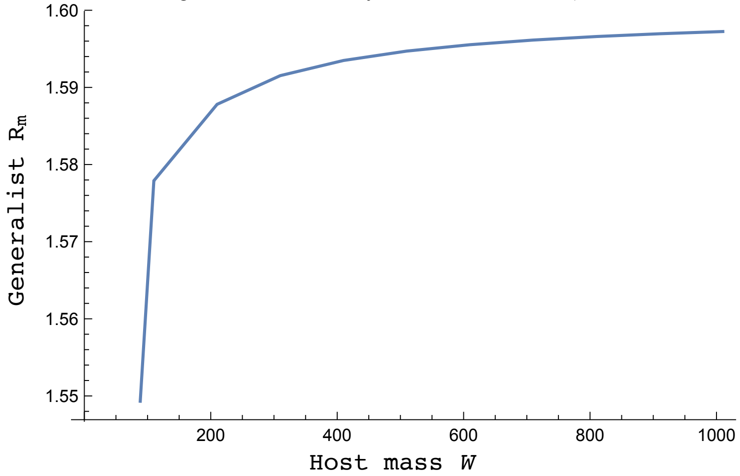

Fig. S28. Effect of body size W on R_m when γ = 1.01

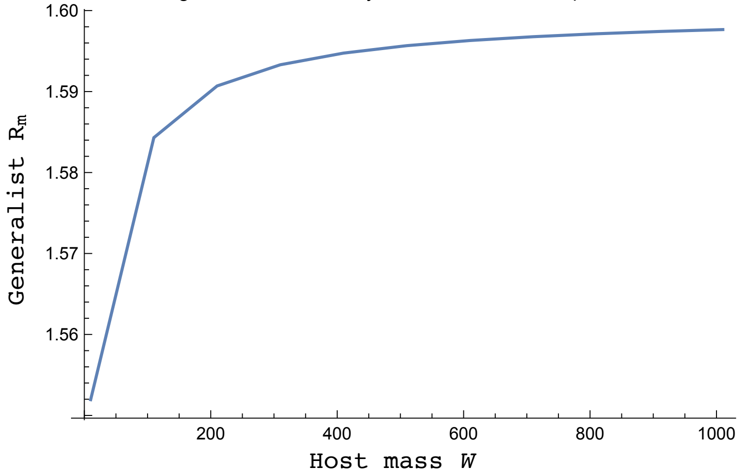

We can also compute the value of R_m, varying host body size W and the temperature T.

```
(* Compute Rm for a range of W and T values *)
RmAcrossWT = Table[Table[RmV /. {β → 1, γ → 0.2, W → Wval, T → Tval, a → 0.8, f → 0.8},
    {Tval, 270, 310, 2}], {Wval, 10, 1010, 100}];
```

Increasing temperature increases R_m, although the increase is very slight, and as host mass increases, the increase in R_m with temperature gets shallower. This can be seen in Figs. S29-S32 below.

```
Tvals = Table[T, {T, 270, 310, 2}];
Labeled[
 ListLinePlot[Table[{Tvals[[i]], Re[RmAcrossWT[[1, i]]]}, {i, 1, Length[Tvals]}],
  PlotLabel → "Fig. S29. Effect of temperature when W = 10"],
 {"Temperature", "Generalist Rm"}, {Bottom, Left}, RotateLabel → True]
Labeled[ListLinePlot[
  Table[{Tvals[[i]], Re[RmAcrossWT[[2, i]]]}, {i, 1, Length[Tvals]}],
  PlotLabel → "Fig. S30. Effect of temperature when W = 110"],
 {"Temperature", "Generalist Rm"}, {Bottom, Left}, RotateLabel → True]
Labeled[ListLinePlot[
  Table[{Tvals[[i]], Re[RmAcrossWT[[6, i]]]}, {i, 1, Length[Tvals]}],
  PlotLabel → "Fig. S31. Effect of temperature when W = 510"],
 {"Temperature", "Generalist Rm"}, {Bottom, Left}, RotateLabel → True]
Labeled[ListLinePlot[
  Table[{Tvals[[i]], Re[RmAcrossWT[[11, i]]]}, {i, 1, Length[Tvals]}],
  PlotLabel → "Fig. S32. Effect of temperature when W = 1010"],
 {"Temperature", "Generalist Rm"}, {Bottom, Left}, RotateLabel → True]
```

Fig. S9. Effect of temperature when W = 10

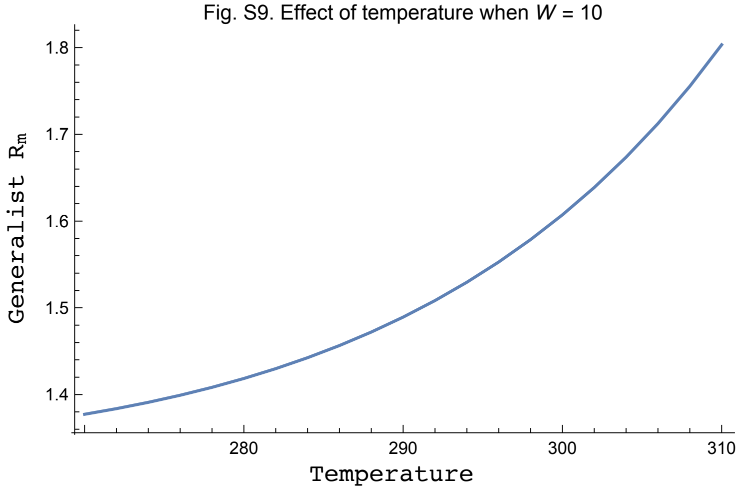

Fig. S10. Effect of temperature when W = 110

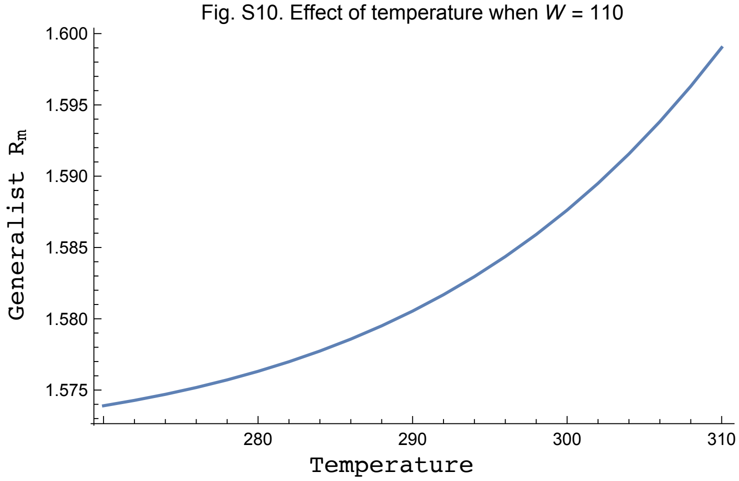

Fig. S11. Effect of temperature when W = 510

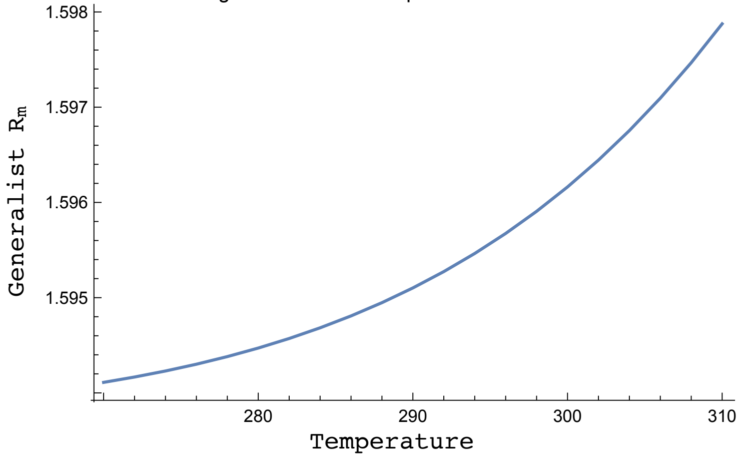

Fig. S12. Effect of temperature when W = 1010

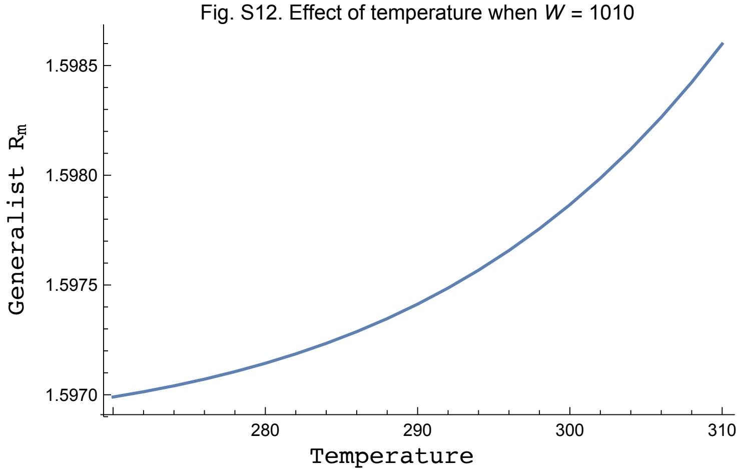

Ectoparasites:

All that needs to be changed from the previous case is the scaling of λ with body size.

$$\text{allom} = \left\{K1 \to K0\,\text{Exp}\left[\frac{E}{k\,T}\right] W^{-3/4},\ K2 \to K0\,\text{Exp}\left[\frac{E}{k\,T}\right] (f\,W)^{-3/4},\ \mu1 \to \mu0\,\text{Exp}\left[-\frac{E}{k\,T}\right] W^{-1/4},\ \mu2 \to \mu0\,\text{Exp}\left[-\frac{E}{k\,T}\right] (f\,W)^{-1/4},\ \lambda1 \to \lambda0\,\text{Exp}\left[-\frac{E}{k\,T}\right] W^{5/12},\ \lambda2 \to \lambda0\,\text{Exp}\left[-\frac{E}{k\,T}\right] (f\,W)^{5/12},\ r1 \to r0\,\text{Exp}\left[-\frac{E}{k\,T}\right] W^{-1/4},\ r2 \to r0\,\text{Exp}\left[-\frac{E}{k\,T}\right] (f\,W)^{-1/4}\right\};$$

$$\text{allompars} = \left\{E \to 0.43,\ k \to \frac{8.617`}{10^5},\ K0 \to \frac{2.984`}{10^9},\ \mu0 \to 1.785` \times 10^8,\ \lambda0 \to 2 \times 10^8,\ r0 \to 2.21 \times 10^{10}\right\};$$

We can compute the value of R_m, varying host body size W and the contact rate β.

```
(* Rm, plugging in the parameter values from Table 1,
the equilibria calculated above, the allometric scaling relationships,
and the parameters of the allometric functions *)
RmV = Rm /. S1Eq[[1]] /. I1sEq[[1]] /. D1ssEq[[1]] /. P1Eq[[2]] /. S2Eq[[1]] /.
        I2sEq[[1]] /. D2ssEq[[1]] /. P2Eq[[2]] /. {σC1 → 1, σC2 → 1, βD1 → 0, βD2 → 0,
      x1 → 1 / 2, x2 → 1 / 2, βS1 → β, βI1 → β, βS2 → β, βI2 → β} /. allom /. allompars;
(* Compute Rm for a range of W and β values *)
RmAcrossWB = Table[Table[RmV /. {β → B, γ → 0.1, W → Wval, T → 270, a → 0.8, f → 0.8},
    {Wval, 10, 1010, 100}], {B, 1, 10, 1}];
```

Increasing host body size increases R_m, regardless of the value of β, thereby making it easier for the generalist to invade. This can be seen in Figs. S33-S36 below.

```
Wvals = Table[W, {W, 10, 1010, 100}];
Labeled[
 ListLinePlot[Table[{Wvals[[i]], Re[RmAcrossWB[[1, i]]]}, {i, 1, Length[Wvals]}],
  PlotLabel → "Fig. S33. Effect of body size W on Rm when β = 1"],
 {"Host mass W", "Generalist Rm"}, {Bottom, Left}, RotateLabel → True]
Labeled[ListLinePlot[
  Table[{Wvals[[i]], Re[RmAcrossWB[[3, i]]]}, {i, 1, Length[Wvals]}],
  PlotLabel → "Fig. S34. Effect of body size W on Rm when β = 3"],
 {"Host mass W", "Generalist Rm"}, {Bottom, Left}, RotateLabel → True]
Labeled[ListLinePlot[
  Table[{Wvals[[i]], Re[RmAcrossWB[[5, i]]]}, {i, 1, Length[Wvals]}],
  PlotLabel → "Fig. S35. Effect of body size W on Rm when β = 5"],
 {"Host mass W", "Generalist Rm"}, {Bottom, Left}, RotateLabel → True]
Labeled[ListLinePlot[
  Table[{Wvals[[i]], Re[RmAcrossWB[[10, i]]]}, {i, 1, Length[Wvals]}],
  PlotLabel → "Fig. S36. Effect of body size W on Rm when β = 10"],
 {"Host mass W", "Generalist Rm"}, {Bottom, Left}, RotateLabel → True]
```

Fig. S33. Effect of body size W on R_m when β = 1

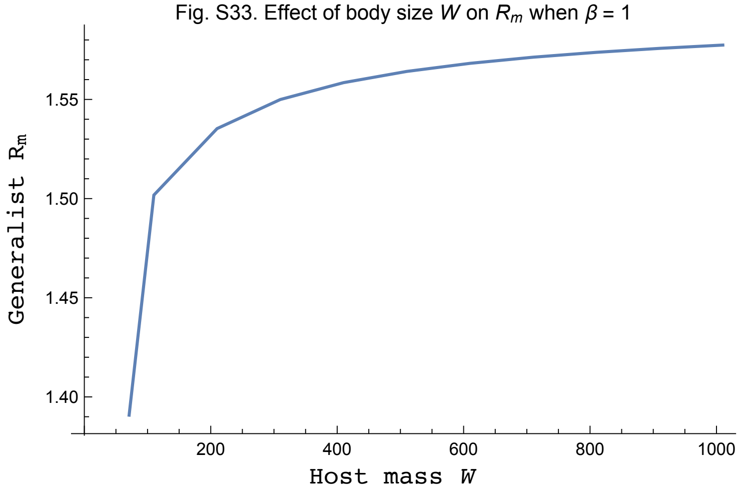

Fig. S34. Effect of body size W on R_m when $\beta = 3$

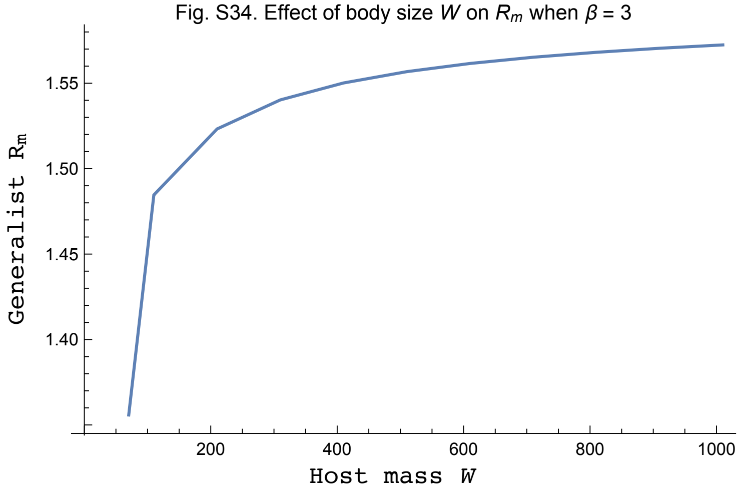

Fig. S35. Effect of body size W on R_m when $\beta = 5$

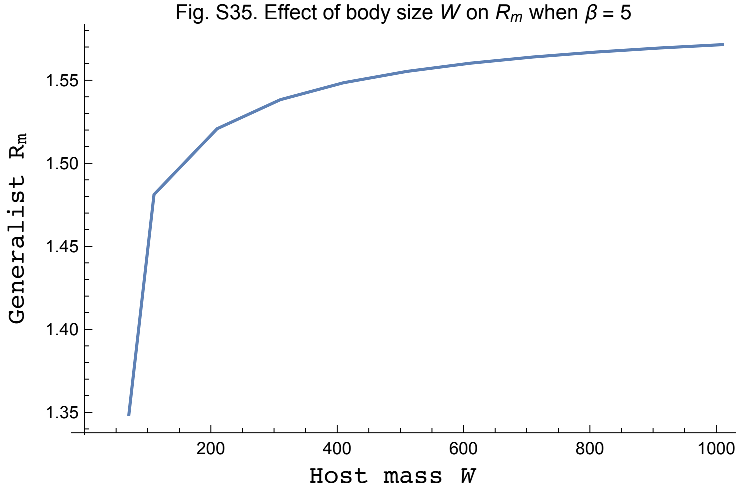

Fig. S36. Effect of body size W on R_m when $\beta = 10$

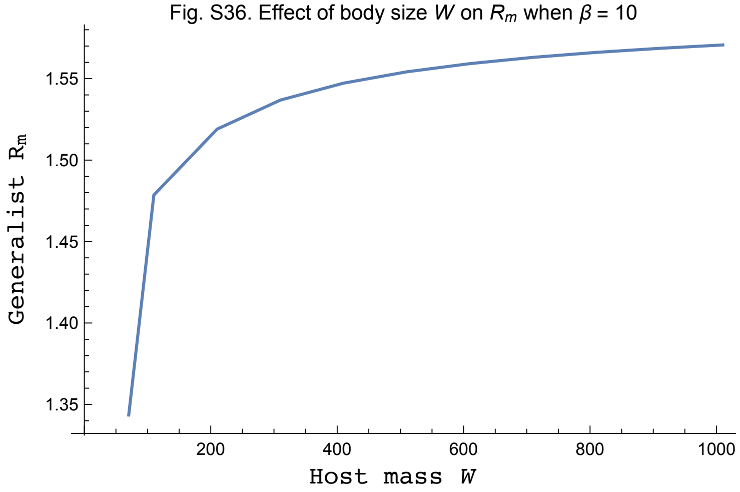

We can also compute the value of R_m, varying host body size W and the parasite loss rate from the environment γ.

```
(* Compute Rm for a range of W and γ values *)
RmAcrossWg = Table[Table[
    RmV /. {β → 1, γ → g, W → Wval, T → 270, a → 0.8, f → 0.8}, {Wval, 10, 1010, 100}],
   {g, 0.01, 0.1, 0.01}];
```

Increasing host body size increases R_m, regardless of the value of γ, thereby making it easier for the generalist to invade. This can been seen in Figs. S37-S40 below.

```
Wvals = Table[W, {W, 10, 1010, 100}];
Labeled[
 ListLinePlot[Table[{Wvals[[i]], Re[RmAcrossWg[[1, i]]]}, {i, 1, Length[Wvals]}],
  PlotLabel → "Fig. S37. Effect of body size W on Rm when γ = 0.01"],
 {"Host mass W", "Generalist Rm"}, {Bottom, Left}, RotateLabel → True]
Labeled[ListLinePlot[
  Table[{Wvals[[i]], Re[RmAcrossWg[[3, i]]]}, {i, 1, Length[Wvals]}],
  PlotLabel → "Fig. S38. Effect of body size W on Rm when γ = 0.03"],
 {"Host mass W", "Generalist Rm"}, {Bottom, Left}, RotateLabel → True]
Labeled[ListLinePlot[
  Table[{Wvals[[i]], Re[RmAcrossWg[[6, i]]]}, {i, 1, Length[Wvals]}],
  PlotLabel → "Fig. S39. Effect of body size W on Rm when γ = 0.06"],
 {"Host mass W", "Generalist Rm"}, {Bottom, Left}, RotateLabel → True]
Labeled[ListLinePlot[
  Table[{Wvals[[i]], Re[RmAcrossWg[[10, i]]]}, {i, 1, Length[Wvals]}],
  PlotLabel → "Fig. S40. Effect of body size W on Rm when γ = 0.1"],
 {"Host mass W", "Generalist Rm"}, {Bottom, Left}, RotateLabel → True]
```

Fig. S37. Effect of body size W on R_m when γ = 0.01

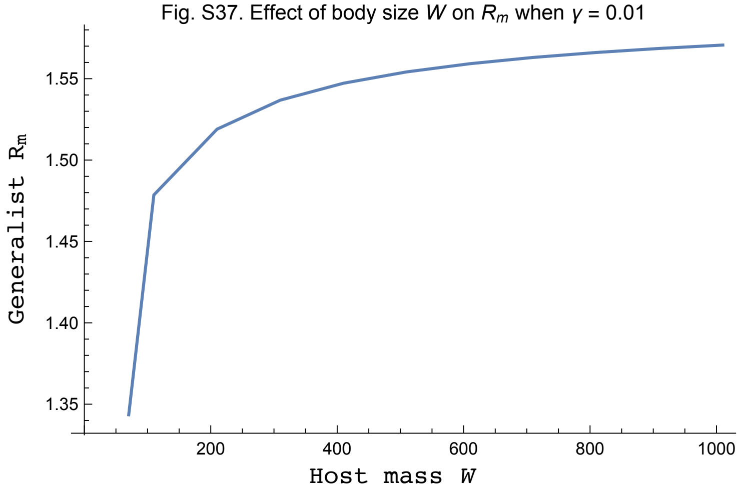

Fig. S38. Effect of body size *W* on R_m when γ = 0.03

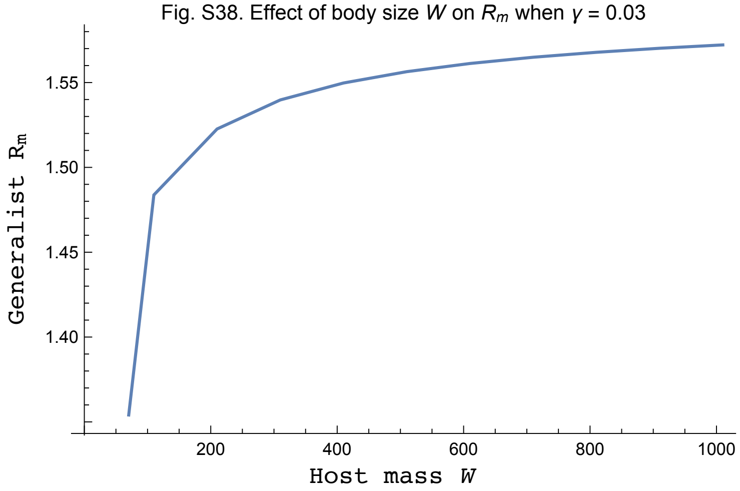

Fig. S39. Effect of body size *W* on R_m when γ = 0.06

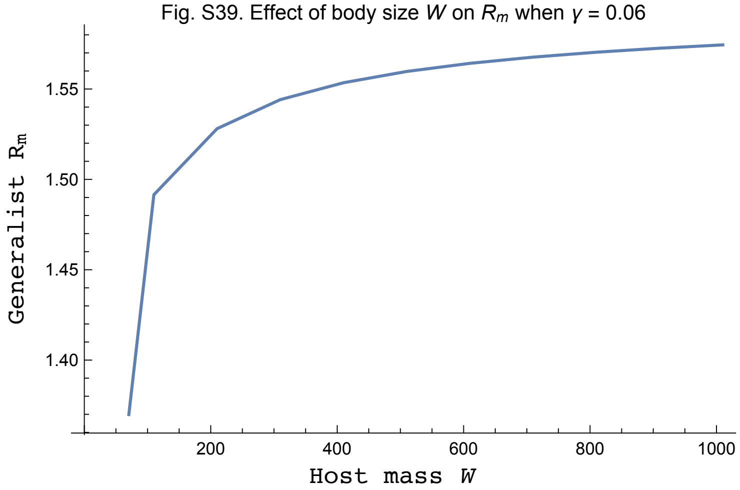

Fig. S40. Effect of body size *W* on R_m when γ = 0.1

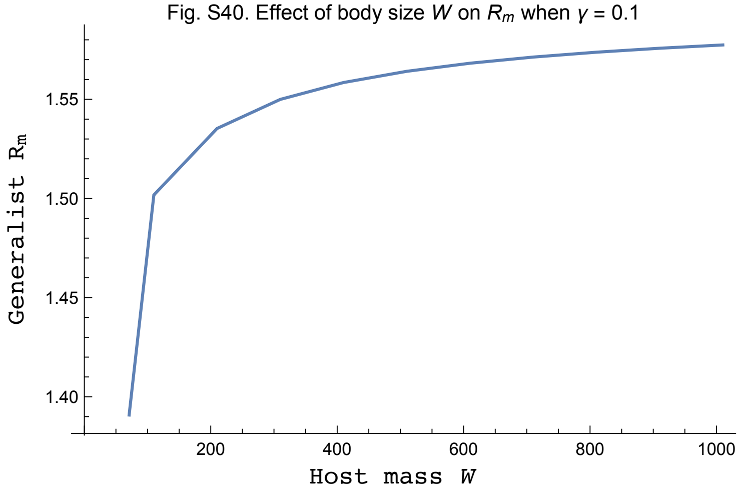

We can also compute the value of R_m, varying host body size *W* and the temperature *T.*

```
(* Compute Rm for a range of W and T values *)
RmAcrossWT = Table[Table[RmV /. {β → 1, γ → 0.1, W → Wval, T → Tval, a → 0.8, f → 0.8},
     {Tval, 270, 310, 2}], {Wval, 10, 1010, 100}];
```

Increasing temperature increases R_m, making it easier for the generalist to invade. This can be seen in Figs. S41-S44 below.

```
Tvals = Table[T, {T, 270, 310, 2}];
Labeled[
 ListLinePlot[Table[{Tvals[[i]], Re[RmAcrossWT[[1, i]]]}, {i, 1, Length[Tvals]}],
  PlotLabel → "Fig. S41. Effect of temperature when W = 10"],
 {"Temperature", "Generalist Rm"}, {Bottom, Left}, RotateLabel → True]
Labeled[ListLinePlot[
  Table[{Tvals[[i]], Re[RmAcrossWT[[2, i]]]}, {i, 1, Length[Tvals]}],
  PlotLabel → "Fig. S42. Effect of temperature when W = 110"],
 {"Temperature", "Generalist Rm"}, {Bottom, Left}, RotateLabel → True]
Labeled[ListLinePlot[
  Table[{Tvals[[i]], Re[RmAcrossWT[[6, i]]]}, {i, 1, Length[Tvals]}],
  PlotLabel → "Fig. S43. Effect of temperature when W = 510"],
 {"Temperature", "Generalist Rm"}, {Bottom, Left}, RotateLabel → True]
Labeled[ListLinePlot[
  Table[{Tvals[[i]], Re[RmAcrossWT[[11, i]]]}, {i, 1, Length[Tvals]}],
  PlotLabel → "Fig. S44. Effect of temperature when W = 1010"],
 {"Temperature", "Generalist Rm"}, {Bottom, Left}, RotateLabel → True]
```

Fig. S41. Effect of temperature when *W* = 10

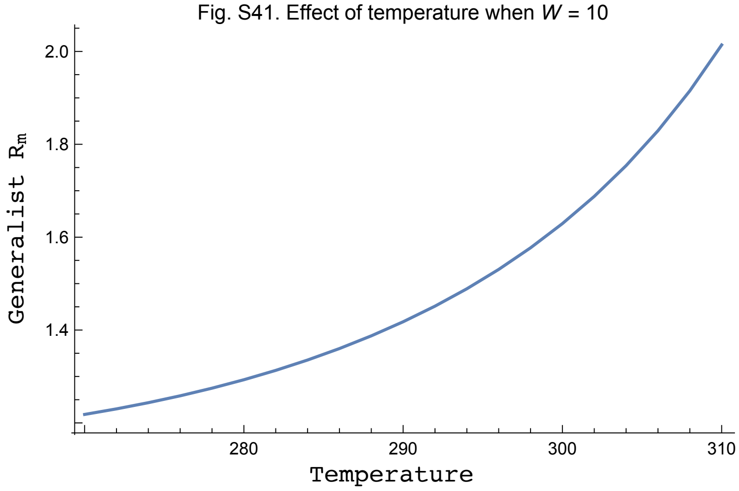

Fig. S42. Effect of temperature when *W* = 110

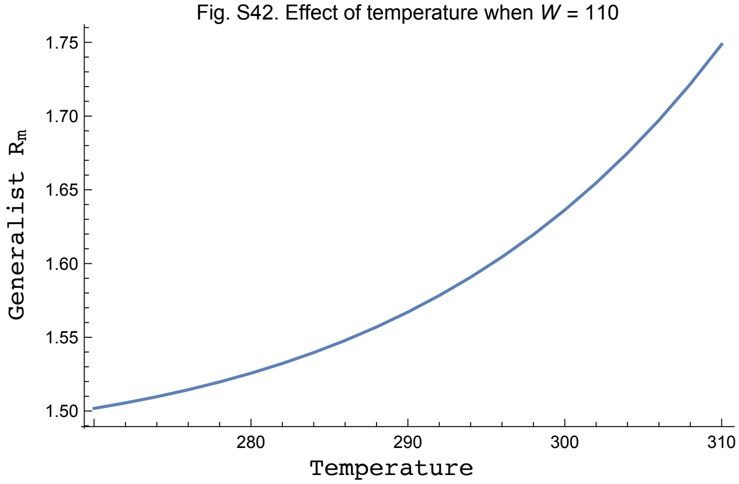

Fig. S43. Effect of temperature when W = 510

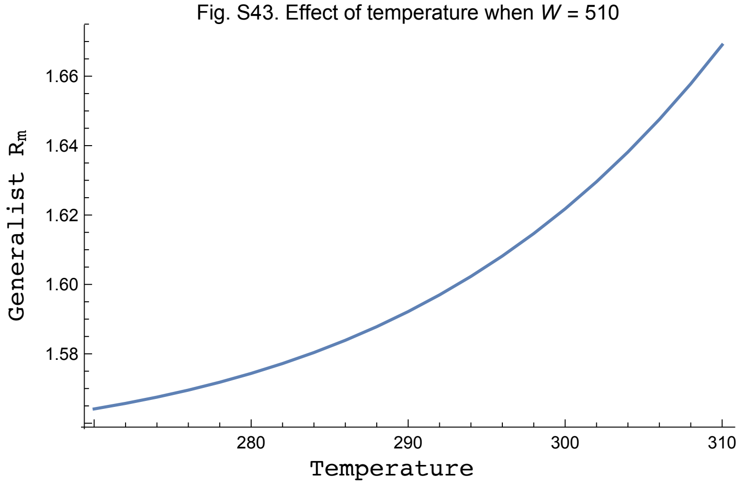

Fig. S44. Effect of temperature when W = 1010

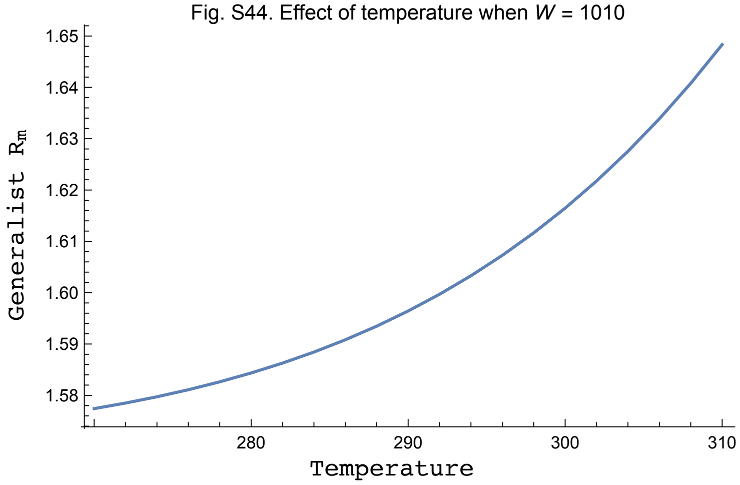

Case 6: Two specialist parasites, coinfection, parasite regulation of host population size; no avoidance of non-susceptible hosts

The R_m expression cannot be simplifed at all from the form presented in Eqn. 12 in the main text.

However, we can solve for $\hat{S}_1$, $\hat{I}_{1,s}$, $\hat{D}_{1,s,s}$ in terms of $\hat{P}_1$ and $\hat{S}_2$, $\hat{I}_{2,s}$, $\hat{D}_{2,s,s}$ in terms of $\hat{P}_2$.

```
Solve[(dP1dt /. {C1sg → 0} /. {S1 → (I1s P1 βI1 + I1s μ1)/(P1 βS1)} /. {I1s → (D1ss μ1)/(P1 βI1)}) == 0, D1ss] //
 Simplify
```

```
{{D1ss → -((P1^2 βI1 γ) / (P1^2 βD1 βI1 - P1 βI1 λ1 + 2 P1 βI1 μ1 - λ1 μ1 + μ1^2))}}
```

```
(* Solving for S₁ in terms of I₁,s and P₁*)
S1Eq = Solve[(dI1sdt /. {σC1 → 1, σD1 → 1, Pg → 0}) == 0, S1];
(* Solving for I₁,s in terms of D₁,s,s and P₁ *)
I1sEq = Solve[(dD1ssdt /. σD1 → 1) == 0, I1s];
(* Solving for D₁,s,s in terms of P₁ *)
D1ssEq = Simplify[Solve[(dP1dt /. {C1sg → 0} /. S1Eq[[1]] /. I1sEq[[1]]) == 0, D1ss]]
(* Solving for I₁,s in terms of P₁ *)
I1sEq = Simplify[I1sEq /. D1ssEq[[1]]]
(* Solving for S₁ in terms of P₁ *)
S1Eq = Simplify[S1Eq /. I1sEq[[1]]]
(* Solving for P₁ *)
P1Eq = Solve[Simplify[dS1dt /. {C1sg → 0, I1g → 0, Pg → 0} /. S1Eq[[1]] /. I1sEq[[1]] /.
      D1ssEq[[1]]] == 0, P1];
(* Solving for S₂ in terms of I₂,s and P₂*)
S2Eq = Solve[(dI2sdt /. {σC2 → 1, σD2 → 1, Pg → 0}) == 0, S2];
(* Solving for I₂,s in terms of D₂,s,s and P₂ *)
I2sEq = Solve[(dD2ssdt /. σD2 → 1) == 0, I2s];
(* Solving for D₂,s,s in terms of P₂ *)
D2ssEq = Simplify[Solve[(dP2dt /. {C2sg → 0} /. S2Eq[[1]] /. I2sEq[[1]]) == 0, D2ss]]
(* Solving for I₂,s in terms of P₂ *)
I2sEq = Simplify[I2sEq /. D2ssEq[[1]]]
(* Solving for S₂ in terms of P₂ *)
S2Eq = Simplify[S2Eq /. I2sEq[[1]]]
(* Solving for P₂ *)
P2Eq = Solve[Simplify[dS2dt /. {C2sg → 0, I2g → 0, Pg → 0} /. S2Eq[[1]] /. I2sEq[[1]] /.
      D2ssEq[[1]]] == 0, P2];
```

$$\{\{D1ss \to -((P1^2\,\beta I1\,\gamma)/(P1^2\,\beta D1\,\beta I1 - P1\,\beta I1\,\lambda 1 + 2\,P1\,\beta I1\,\mu 1 - \lambda 1\,\mu 1 + \mu 1^2))\}\}$$

$$\{\{I1s \to -((P1\,\gamma\,\mu 1)/(P1^2\,\beta D1\,\beta I1 - P1\,\beta I1\,\lambda 1 + 2\,P1\,\beta I1\,\mu 1 - \lambda 1\,\mu 1 + \mu 1^2))\}\}$$

$$\{\{S1 \to -((\gamma\,\mu 1\,(P1\,\beta I1 + \mu 1))/(\beta S1\,(P1^2\,\beta D1\,\beta I1 - P1\,\beta I1\,(\lambda 1 - 2\,\mu 1) + \mu 1\,(-\lambda 1 + \mu 1))))\}\}$$

$$\{\{D2ss \to -((P2^2\,\beta I2\,\gamma)/(P2^2\,\beta D2\,\beta I2 - P2\,\beta I2\,\lambda 2 + 2\,P2\,\beta I2\,\mu 2 - \lambda 2\,\mu 2 + \mu 2^2))\}\}$$

$$\{\{I2s \to -((P2\,\gamma\,\mu 2)/(P2^2\,\beta D2\,\beta I2 - P2\,\beta I2\,\lambda 2 + 2\,P2\,\beta I2\,\mu 2 - \lambda 2\,\mu 2 + \mu 2^2))\}\}$$

$$\{\{S2 \to -((\gamma\,\mu 2\,(P2\,\beta I2 + \mu 2))/(\beta S2\,(P2^2\,\beta D2\,\beta I2 - P2\,\beta I2\,(\lambda 2 - 2\,\mu 2) + \mu 2\,(-\lambda 2 + \mu 2))))\}\}$$

If we plug these equilibria into the expression for R_m, we can simplify it considerably, if we also make the assumption that $x_1 = x_2 = 0.5$, $\sigma_{C_1} = \sigma_{C_2} = 1$, and $\beta_{I_1} = \beta_{I_2} = \beta_{D_1} = \beta_{D_2} = \beta$.

```
(* Simplifying the expression for Rₘ *)
Simplify[
 Rm /. S1Eq[[1]] /. I1sEq[[1]] /. D1ssEq[[1]] /. S2Eq[[1]] /. I2sEq[[1]] /. D2ssEq[[
    1]] /. {x1 → 1 / 2, x2 → 1 / 2, σC1 → 1, σC2 → 1, βI1 → β, βI2 → β, βD1 → β, βD2 → β}]
```

$$(a\,(P2\,\beta\,\lambda 1 + P1\,\beta\,\lambda 2 - 2\,\lambda 1\,\lambda 2 + \lambda 2\,\mu 1 + \lambda 1\,\mu 2))\,/\,(-\lambda 1\,\lambda 2 + P1\,\beta\,(P2\,\beta + \mu 2) + \mu 1\,(P2\,\beta + \mu 2))$$

The generalist can only invade if the numerator is larger than then denominator when $a = 1$. This is only true if $(\beta\hat{P}_1 - \lambda_1 + \mu_1)(\beta\hat{P}_2 - \lambda_2 + \mu_2) < 0$. But both of these expressions must be negative to guarantee the positivity of $\hat{S}_1$ and $\hat{S}_2$, which means that the generalist can never invade.

```
(* Is the numerator ever larger than the
 denominator? This requires the following to be true *)
Simplify[Expand[(P2 β λ1 + P1 β λ2 - 2 λ1 λ2 + λ2 μ1 + λ1 μ2) >
   -λ1 λ2 + P1 β (P2 β + μ2) + μ1 (P2 β + μ2)]]
```

```
(P1 β - λ1 + μ1) (P2 β - λ2 + μ2) < 0
```

Derivation of R_m for a model with constant host population size

Here we must derive a new model, since we are no longer assuming that the parasite regulates the host population size. We assume that the population sizes for hosts 1 and 2 are constant at K_1 and K_2, respectively. As such, we do not need to keep track of the dynamics of both susceptible and infected hosts - we can simply track the prevalence of infection. For example, we define $I_{1,s}$ and $I_{2,s}$ as the fraction of the host populations that are singly-infected by the specialist parasites, respectively.

The dynamics of parasites in the environment are governed by shedding and loss, as before. The rate of shedding depends on the *number* of infected hosts, e.g., $I_{1\,r} K_1$. Similarly, loss due to contact with susceptible hosts depends on the *number* of susceptible hosts, e.g., $(1 - I_{1\,r} - I_{1\,m})\, K_1$.

The model is given below:

In[85]:=
```
(* Dynamics of individuals of host species
 1 singly infected with its specialist parasite *)
dI1sdt = βS1 (1 - I1s - D1ss - I1g - C1sg) P1 - σD1 βI1 I1s P1 - σC1 βI1 I1s Pg - μ1 I1s;
(* Dynamics of individuals of host species
 1 doubly infected with its specialist parasite *)
dD1ssdt = σD1 βI1 I1s P1 - μ1 D1ss;
(* Dynamics of the specialist parasite of host species 1 in the environment *)
dP1dt = λ1 (I1s + D1ss + x1 C1sg) K1 -
   (βS1 (1 - I1s - D1ss) + βI1 I1s + βD1 D1ss + βI1 I1g + βC1 C1sg) K1 P1 - γ P1;

(* Dynamics of individuals of host species
 2 singly infected with its specialist parasite *)
dI2sdt = βS2 (1 - I2s - D2ss - I2g - C2sg) P2 - σD2 βI2 I2s P2 - σC2 βI2 I2s Pg - μ2 I2s;
(* Dynamics of individuals of host species
 2 doubly infected with its specialist parasite *)
dD2ssdt = σD2 βI2 I2s P2 - μ2 D2ss;
(* Dynamics of the specialist parasite of host species 2 in the environment *)
dP2dt = λ2 (I2s + D2ss + x2 C2sg) K2 -
   (βS2 (1 - I2s - D2ss) + βI2 I2s + βD2 D2ss + βI2 I2g + βC2 C2sg) K2 P2 - γ P2;

(* Dynamics of individuals of host species
 1 singly infected with the generalist parasite *)
dI1gdt = βS1 (1 - I1s - D1ss - I1g - C1sg) Pg - σC1 βI1 I1g P1 - μ1 I1g;
(* Dynamics of individuals of host species
 2 singly infected with the generalist parasite *)
dI2gdt = βS2 (1 - I2s - D2ss - I2g - C2sg) Pg - σC2 βI2 I2g P2 - μ2 I2g;
(* Dynamics of individuals of host species 1
 coinfected with its specialist and the generalist parasite *)
dC1sgdt = σC1 βI1 (I1s Pg + I1g P1) - μ1 C1sg;
(* Dynamics of individuals of host species 2
 coinfected with its specialist and the generalist parasite *)
dC2sgdt = σC2 βI2 (I2s Pg + I2g P2) - μ2 C2sg;
(* Dynamics of the generalist parasite in the environment *)
dPgdt = a λ1 (I1g + (1 - x1) C1sg) K1 +
   a λ2 (I2g + (1 - x2) C2sg) K2 - (βS1 (1 - I1s - D1ss) + βI1 I1s + βD1 D1ss) K1 Pg -
   (βS2 (1 - I2s - D2ss) + βI2 I2s + βD2 D2ss) K2 Pg - γ Pg;
```

Whether the generalist parasite can invade will depend on the stability of the equilibrium $\left(\hat{I}_{1,s}, \hat{D}_{1,s,s}, \hat{P}_1, \hat{I}_{2,s}, \hat{D}_{2,s,s}, \hat{P}_2, 0, 0, 0, 0, 0\right)$. This can be evaluated by looking at the eigenvalues of the Jacobian matrix for the full system. The Jacobian matrix at this equilibrium has a simple block upper triangular structure: $J = \begin{pmatrix} J_1 & 0 & M_1 \\ 0 & J_2 & M_2 \\ 0 & 0 & J_m \end{pmatrix}$, where J_1 is the submatrix that determines the stability of the $(I_{1,s}, D_{1,s,s}, P_1)$ subsystem and J_2 is the submatrix that determines the stability of the $(I_{2,s}, D_{2,s,s}, P_2)$ subsystem. J_m is the submatrix of partial derivatives involving the equations for the generalist. Because of its simple structure, the eigenvalues of the full system are given by the eigenvalues of the submatrices J_1, J_2 and J_m. Assuming that the $(I_{1,s}, D_{1,s,s}, P_1)$ and $(I_{2,s}, D_{2,s,s}, P_2)$ subsystems are both stable, all of the eigenvalues of J_1 and J_2 are negative. Therefore, we are interested only in the eigenvalues of J_m.

In[96]:=
```
(* Calculating the Jacobian matrix and evaluating
  it at the equilibrium where I1,g=I2,g=C1,s,g=C2,s,g=Pg=0 *)
J = {{D[dI1sdt, I1s], D[dI1sdt, D1ss], D[dI1sdt, P1],
     D[dI1sdt, I2s], D[dI1sdt, D2ss], D[dI1sdt, P2],
     D[dI1sdt, I1g], D[dI1sdt, I2g],
     D[dI1sdt, C1sg], D[dI1sdt, C2sg], D[dI1sdt, Pg]},
    {D[dD1ssdt, I1s], D[dD1ssdt, D1ss], D[dD1ssdt, P1],
     D[dD1ssdt, I2s], D[dD1ssdt, D2ss], D[dD1ssdt, P2],
     D[dD1ssdt, I1g], D[dD1ssdt, I2g],
     D[dD1ssdt, C1sg], D[dD1ssdt, C2sg], D[dD1ssdt, Pg]},
    {D[dP1dt, I1s], D[dP1dt, D1ss], D[dP1dt, P1],
     D[dP1dt, I2s], D[dP1dt, D2ss], D[dP1dt, P2],
     D[dP1dt, I1g], D[dP1dt, I2g], D[dP1dt, C1sg], D[dP1dt, C2sg], D[dP1dt, Pg]},
    {D[dI2sdt, I1s], D[dI2sdt, D1ss], D[dI2sdt, P1],
     D[dI2sdt, I2s], D[dI2sdt, D2ss], D[dI2sdt, P2],
     D[dI2sdt, I1g], D[dI2sdt, I2g],
     D[dI2sdt, C1sg], D[dI2sdt, C2sg], D[dI2sdt, Pg]},
    {D[dD2ssdt, I1s], D[dD2ssdt, D1ss], D[dD2ssdt, P1],
     D[dD2ssdt, I2s], D[dD2ssdt, D2ss], D[dD2ssdt, P2],
     D[dD2ssdt, I1g], D[dD2ssdt, I2g],
     D[dD2ssdt, C1sg], D[dD2ssdt, C2sg], D[dD2ssdt, Pg]},
    {D[dP2dt, I1s], D[dP2dt, D1ss], D[dP2dt, P1],
     D[dP2dt, I2s], D[dP2dt, D2ss], D[dP2dt, P2],
     D[dP2dt, I1g], D[dP2dt, I2g], D[dP2dt, C1sg], D[dP2dt, C2sg], D[dP2dt, Pg]},
    {D[dI1gdt, I1s], D[dI1gdt, D1ss], D[dI1gdt, P1],
     D[dI1gdt, I2s], D[dI1gdt, D2ss], D[dI1gdt, P2],
     D[dI1gdt, I1g], D[dI1gdt, I2g],
     D[dI1gdt, C1sg], D[dI1gdt, C2sg], D[dI1gdt, Pg]},
    {D[dI2gdt, I1s], D[dI2gdt, D1ss], D[dI2gdt, P1],
     D[dI2gdt, I2s], D[dI2gdt, D2ss], D[dI2gdt, P2],
     D[dI2gdt, I1g], D[dI2gdt, I2g],
     D[dI2gdt, C1sg], D[dI2gdt, C2sg], D[dI2gdt, Pg]},
    {D[dC1sgdt, I1s], D[dC1sgdt, D1ss], D[dC1sgdt, P1],
     D[dC1sgdt, I2s], D[dC1sgdt, D2ss], D[dC1sgdt, P2],
     D[dC1sgdt, I1g], D[dC1sgdt, I2g],
     D[dC1sgdt, C1sg], D[dC1sgdt, C2sg], D[dC1sgdt, Pg]},
    {D[dC2sgdt, I1s], D[dC2sgdt, D1ss], D[dC2sgdt, P1],
     D[dC2sgdt, I2s], D[dC2sgdt, D2ss], D[dC2sgdt, P2],
     D[dC2sgdt, I1g], D[dC2sgdt, I2g],
     D[dC2sgdt, C1sg], D[dC2sgdt, C2sg], D[dC2sgdt, Pg]},
    {D[dPgdt, I1s], D[dPgdt, D1ss], D[dPgdt, P1],
     D[dPgdt, I2s], D[dPgdt, D2ss], D[dPgdt, P2],
     D[dPgdt, I1g], D[dPgdt, I2g], D[dPgdt, C1sg], D[dPgdt, C2sg], D[dPgdt, Pg]}} /.
   {I1g → 0, I2g → 0, C1sg → 0, C2sg → 0, Pg → 0};
(* The submatrices *)
(* J1 *)
MatrixForm[J1 = J[[1 ;; 3, 1 ;; 3]]]
```

Out[97]//MatrixForm=

$$\begin{pmatrix} -P1\,\beta S1-\mu 1-P1\,\beta I1\,\sigma D1 & -P1\,\beta S1 & (1-D1ss-I1s)\,\beta S1-I1s\,\beta I1\,\sigma D1 \\ P1\,\beta I1\,\sigma D1 & -\mu 1 & I1s\,\beta I1\,\sigma D1 \\ -K1\,P1\,(\beta I1-\beta S1)+K1\,\lambda 1 & -K1\,P1\,(\beta D1-\beta S1)+K1\,\lambda 1 & -K1\,(D1ss\,\beta D1+I1s\,\beta I1+(1-D1ss-I1s) \end{pmatrix}$$

In[68]:=
```
(* J2 *)
MatrixForm[J1 = J[[4 ;; 6, 4 ;; 6]]]
```

Out[68]//MatrixForm=

$$\begin{pmatrix} -P2\,\beta S2 - \mu 2 - P2\,\beta I2\,\sigma D2 & -P2\,\beta S2 & (1 - D2ss - I2s)\,\beta S2 - I2s\,\beta I2\,\sigma D2 \\ P2\,\beta I2\,\sigma D2 & -\mu 2 & I2s\,\beta I2\,\sigma D2 \\ -K2\,P2\,(\beta I2 - \beta S2) + K2\,\lambda 2 & -K2\,P2\,(\beta D2 - \beta S2) + K2\,\lambda 2 & -K2\,(D2ss\,\beta D2 + I2s\,\beta I2 + (1 - D2ss - I2s) \end{pmatrix}$$

In[69]:=
```
(* M1 *)
MatrixForm[J1 = J[[1 ;; 3, 7 ;; 11]]]
```

Out[69]//MatrixForm=

$$\begin{pmatrix} -P1\,\beta S1 & 0 & -P1\,\beta S1 & 0 & -I1s\,\beta I1\,\sigma C1 \\ 0 & 0 & 0 & 0 & 0 \\ -K1\,P1\,\beta I1 & 0 & -K1\,P1\,\beta C1 + K1\,x1\,\lambda 1 & 0 & 0 \end{pmatrix}$$

In[70]:=
```
(* M12*)
MatrixForm[J1 = J[[4 ;; 6, 7 ;; 11]]]
```

Out[70]//MatrixForm=

$$\begin{pmatrix} 0 & -P2\,\beta S2 & 0 & -P2\,\beta S2 & -I2s\,\beta I2\,\sigma C2 \\ 0 & 0 & 0 & 0 & 0 \\ 0 & -K2\,P2\,\beta I2 & 0 & -K2\,P2\,\beta C2 + K2\,x2\,\lambda 2 & 0 \end{pmatrix}$$

```
(* Jm *)
```

In[98]:=
```
MatrixForm[Jm = J[[7 ;; 11, 7 ;; 11]]]
```

Out[98]//MatrixForm=

$$\begin{pmatrix} -\mu 1 - P1\,\beta I1\,\sigma C1 & 0 & 0 & 0 \\ 0 & -\mu 2 - P2\,\beta I2\,\sigma C2 & 0 & 0 \\ P1\,\beta I1\,\sigma C1 & 0 & -\mu 1 & 0 \\ 0 & P2\,\beta I2\,\sigma C2 & 0 & -\mu 2 \\ a\,K1\,\lambda 1 & a\,K2\,\lambda 2 & a\,K1\,(1 - x1)\,\lambda 1 & a\,K2\,(1 - x2)\,\lambda 2 & -K1\,(D1ss\,\beta D1 + I1s\,\beta I1 + (\end{pmatrix}$$

The submatrix

$$J_m = \begin{pmatrix} -\mu_1 - \sigma_{C_1}\,\beta_{I_1}\,\hat{P}_1 & 0 & 0 & 0 \\ 0 & -\mu_2 - \sigma_{C_2}\,\beta_{I_2}\,\hat{P}_2 & 0 & 0 \\ \sigma_{C_1}\,\beta_{I_1}\,\hat{P}_1 & 0 & -\mu_1 & 0 \\ 0 & \sigma_{C_2}\,\beta_{I_2}\,\hat{P}_2 & 0 & -\mu_2 \\ a\,\lambda_1\,K_1 & a\,\lambda_2\,K_2 & a\,(1 - x_1)\,\lambda_1\,K_1 & a\,(1 - x_2)\,\lambda_2\,K_2 & -\left(\beta_{S_1}\left(1 - \hat{I}_{1,s} - \hat{D}_{1,s,s}\right) + \beta_{I_1}\,\hat{I}_{1,s} + \beta_D \end{pmatrix}$$

.

Rather than finding for the eigenvalues of this submatrix, we make use of the Next Generation Theorem

and rewrite J_m as $F - V$, where $F = \begin{pmatrix} 0 & 0 & 0 & 0 & \beta_{S_2}\left(1 - \hat{I}_{1,s} - \hat{D}_{1,s,s}\right) \\ 0 & 0 & 0 & 0 & \beta_{S_2}\left(1 - \hat{I}_{2,s} - \hat{D}_{2,s,s}\right) \\ \sigma_{C_1}\,\beta_{I_1}\,\hat{P}_1 & 0 & 0 & 0 & \sigma_{C_1}\,\beta_{I_1}\,\hat{I}_{1,s} \\ 0 & \sigma_{C_2}\,\beta_{I_2}\,\hat{P}_2 & 0 & 0 & \sigma_{C_2}\,\beta_{I_2}\,\hat{I}_{2,s} \\ 0 & 0 & 0 & 0 & 0 \end{pmatrix}$ and

$$V = \begin{pmatrix} \mu_1 + \sigma_{C_1}\,\beta_{I_1}\,\hat{P}_1 & 0 & 0 & 0 \\ 0 & \mu_2 + \sigma_{C_2}\,\beta_{I_2}\,\hat{P}_2 & 0 & 0 \\ 0 & 0 & \mu 1 & 0 \\ 0 & 0 & 0 & \mu 2 \\ -a\,\lambda_1\,K_1 & -a\,\lambda_2\,K_2 & -a\,(1 - x_1)\,\lambda_1\,K_1 & -a\,(1 - x_2)\,\lambda_2\,K_2 & \left(\beta_{S_1}\left(1 - \hat{I}_{1,s} - \hat{D}_{1,s,s}\right) + \beta_{I_1}\,\hat{I}_{1,s} + \beta_{D_1}\,D \end{pmatrix}$$

In[100]:=
```
F = {{0, 0, 0, 0, (1 - I1s - D1ss) βS1}, {0, 0, 0, 0, (1 - I2s - D2ss) βS2},
    {P1 βI1 σC1, 0, 0, 0, I1s βI1 σC1}, {0, P2 βI2 σC2, 0, 0, I2s βI2 σC2}, {0, 0, 0, 0, 0}};
V = {{μ1 + P1 βI1 σC1, 0, 0, 0, 0}, {0, μ2 + P2 βI2 σC2, 0, 0, 0},
    {0, 0, μ1, 0, 0}, {0, 0, 0, μ2, 0}, {-a λ1 K1, -a λ2 K2, -a (1 - x1) λ1 K1,
     -a (1 - x2) λ2 K2, ((1 - I1s - D1ss) βS1 + I1s βI1 + D1ss βD1) K1 +
      (D2ss βD2 + I2s βI2 + (1 - I2s - D2ss) βS2) K2 + γ}};
Jm == F - V // Simplify
```

Out[102]=
```
True
```

The Next Generation Theorem states that, if a matrix J can be written $J = F - V$, where $F \geq 0$, $V^{-1} \geq 0$ and all of the eigenvalues of $-V$ are negative, then the dominant eigenvalue of J will be greater than zero whenever the spectral radius of $F.V^{-1} > 1$. Note that the spectral radius largest real part of all of the eigenvalues.

In[103]:=
```
(* Verifying that all elements of V^-1≥0*)
Inverse[V] // Simplify
```

Out[103]=
```
{{1/(μ1 + P1 βI1 σC1), 0, 0, 0, 0},
 {0, 1/(μ2 + P2 βI2 σC2), 0, 0, 0}, {0, 0, 1/μ1, 0, 0}, {0, 0, 0, 1/μ2, 0},
 {(a K1 λ1) / ((I1s K1 βI1 + I2s K2 βI2 + D1ss K1 (βD1 - βS1) + K1 βS1 - I1s K1 βS1 +
        D2ss K2 (βD2 - βS2) + K2 βS2 - I2s K2 βS2 + γ) (μ1 + P1 βI1 σC1)),
   (a K2 λ2) / ((I1s K1 βI1 + I2s K2 βI2 + D1ss K1 (βD1 - βS1) + K1 βS1 - I1s K1 βS1 +
        D2ss K2 (βD2 - βS2) + K2 βS2 - I2s K2 βS2 + γ) (μ2 + P2 βI2 σC2)),
   (a K1 (1 - x1) λ1) / ((K1 (D1ss (βD1 - βS1) + I1s (βI1 - βS1) + βS1) +
        K2 (D2ss (βD2 - βS2) + I2s (βI2 - βS2) + βS2) + γ) μ1),
   (a K2 (1 - x2) λ2) / ((K1 (D1ss (βD1 - βS1) + I1s (βI1 - βS1) + βS1) +
        K2 (D2ss (βD2 - βS2) + I2s (βI2 - βS2) + βS2) + γ) μ2),
   1 / (K1 (D1ss (βD1 - βS1) + I1s (βI1 - βS1) + βS1) +
      K2 (D2ss (βD2 - βS2) + I2s (βI2 - βS2) + βS2) + γ)}}
```

In[104]:=
```
(* Verifying that all eigenvalues of -V<0 *)
Eigenvalues[-V] // Simplify
```

Out[104]=
```
{-K1 (D1ss (βD1 - βS1) + I1s (βI1 - βS1) + βS1) - K2 (D2ss (βD2 - βS2) + I2s (βI2 - βS2) + βS2) -
   γ, -μ1, -μ2, -μ1 - P1 βI1 σC1, -μ2 - P2 βI2 σC2}
```

```
(* Eigenvalues of F.V^-1 *)
```

```
In[105]:= Eigenvalues[Dot[F, Inverse[V]]] // Simplify

Out[105]= {0, 0, 0,
  -((-a K2 βS2 λ2 μ1^2 μ2 + a D2ss K2 βS2 λ2 μ1^2 μ2 + a I2s K2 βS2 λ2 μ1^2 μ2 - a K1 βS1 λ1 μ1 μ2^2 +
      a D1ss K1 βS1 λ1 μ1 μ2^2 + a I1s K1 βS1 λ1 μ1 μ2^2 - a K2 P1 βI1 βS2 λ2 μ1 μ2 σC1 +
      a D2ss K2 P1 βI1 βS2 λ2 μ1 μ2 σC1 + a I2s K2 P1 βI1 βS2 λ2 μ1 μ2 σC1 -
      a I1s K1 βI1 λ1 μ1 μ2^2 σC1 + a I1s K1 x1 βI1 λ1 μ1 μ2^2 σC1 - a I1s K1 P1 βI1^2 λ1 μ2^2 σC1^2 +
      a I1s K1 P1 x1 βI1^2 λ1 μ2^2 σC1^2 - a K1 P2 βI2 βS1 λ1 μ1 μ2 σC2 +
      a D1ss K1 P2 βI2 βS1 λ1 μ1 μ2 σC2 + a I1s K1 P2 βI2 βS1 λ1 μ1 μ2 σC2 -
      a I2s K2 βI2 λ2 μ1^2 μ2 σC2 + a I2s K2 x2 βI2 λ2 μ1^2 μ2 σC2 -
      a I1s K1 P2 βI1 βI2 λ1 μ1 μ2 σC1 σC2 + a I1s K1 P2 x1 βI1 βI2 λ1 μ1 μ2 σC1 σC2 -
      a I2s K2 P1 βI1 βI2 λ2 μ1 μ2 σC1 σC2 + a I2s K2 P1 x2 βI1 βI2 λ2 μ1 μ2 σC1 σC2 -
      a I1s K1 P1 P2 βI1^2 βI2 λ1 μ2 σC1^2 σC2 + a I1s K1 P1 P2 x1 βI1^2 βI2 λ1 μ2 σC1^2 σC2 -
      a I2s K2 P2 βI2^2 λ2 μ1^2 σC2^2 + a I2s K2 P2 x2 βI2^2 λ2 μ1^2 σC2^2 -
      a I2s K2 P1 P2 βI1 βI2^2 λ2 μ1 σC1 σC2^2 + a I2s K2 P1 P2 x2 βI1 βI2^2 λ2 μ1 σC1 σC2^2 -
      √(a (4 (I1s K1 βI1 + I2s K2 βI2 + D1ss K1 (βD1 - βS1) + K1 βS1 - I1s K1 βS1 +
              D2ss K2 (βD2 - βS2) + K2 βS2 - I2s K2 βS2 + γ) μ1 μ2 (μ1 + P1 βI1 σC1) (μ2 +
              P2 βI2 σC2) ((-1 + D2ss + I2s) K2 P2 (-1 + x2) βI2 βS2 λ2 μ1 (μ1 + P1 βI1 σC1)
               σC2 + (-1 + D1ss + I1s) K1 P1 (-1 + x1) βI1 βS1 λ1 μ2 σC1 (μ2 + P2 βI2 σC2)) +
          a (K1 λ1 μ2 ((-1 + D1ss + I1s) βS1 μ1 + I1s (-1 + x1) βI1 σC1 (μ1 + P1 βI1 σC1))
                (μ2 + P2 βI2 σC2) + K2 λ2 μ1 (μ1 + P1 βI1 σC1)
                ((-1 + D2ss + I2s) βS2 μ2 + I2s (-1 + x2) βI2 σC2 (μ2 + P2 βI2 σC2)))^2))) /
    (2 (I1s K1 βI1 + I2s K2 βI2 + D1ss K1 (βD1 - βS1) + K1 βS1 - I1s K1 βS1 +
        D2ss K2 (βD2 - βS2) + K2 βS2 - I2s K2 βS2 + γ)
      μ1 μ2 (μ1 + P1 βI1 σC1) (μ2 + P2 βI2 σC2))),
  -((-a K2 βS2 λ2 μ1^2 μ2 + a D2ss K2 βS2 λ2 μ1^2 μ2 + a I2s K2 βS2 λ2 μ1^2 μ2 -
      a K1 βS1 λ1 μ1 μ2^2 + a D1ss K1 βS1 λ1 μ1 μ2^2 + a I1s K1 βS1 λ1 μ1 μ2^2 -
      a K2 P1 βI1 βS2 λ2 μ1 μ2 σC1 + a D2ss K2 P1 βI1 βS2 λ2 μ1 μ2 σC1 +
      a I2s K2 P1 βI1 βS2 λ2 μ1 μ2 σC1 - a I1s K1 βI1 λ1 μ1 μ2^2 σC1 +
      a I1s K1 x1 βI1 λ1 μ1 μ2^2 σC1 - a I1s K1 P1 βI1^2 λ1 μ2^2 σC1^2 +
      a I1s K1 P1 x1 βI1^2 λ1 μ2^2 σC1^2 - a K1 P2 βI2 βS1 λ1 μ1 μ2 σC2 +
      a D1ss K1 P2 βI2 βS1 λ1 μ1 μ2 σC2 + a I1s K1 P2 βI2 βS1 λ1 μ1 μ2 σC2 -
      a I2s K2 βI2 λ2 μ1^2 μ2 σC2 + a I2s K2 x2 βI2 λ2 μ1^2 μ2 σC2 -
      a I1s K1 P2 βI1 βI2 λ1 μ1 μ2 σC1 σC2 + a I1s K1 P2 x1 βI1 βI2 λ1 μ1 μ2 σC1 σC2 -
      a I2s K2 P1 βI1 βI2 λ2 μ1 μ2 σC1 σC2 + a I2s K2 P1 x2 βI1 βI2 λ2 μ1 μ2 σC1 σC2 -
      a I1s K1 P1 P2 βI1^2 βI2 λ1 μ2 σC1^2 σC2 + a I1s K1 P1 P2 x1 βI1^2 βI2 λ1 μ2 σC1^2 σC2 -
      a I2s K2 P2 βI2^2 λ2 μ1^2 σC2^2 + a I2s K2 P2 x2 βI2^2 λ2 μ1^2 σC2^2 -
      a I2s K2 P1 P2 βI1 βI2^2 λ2 μ1 σC1 σC2^2 + a I2s K2 P1 P2 x2 βI1 βI2^2 λ2 μ1 σC1 σC2^2 +
      √(a (4 (I1s K1 βI1 + I2s K2 βI2 + D1ss K1 (βD1 - βS1) + K1 βS1 - I1s K1 βS1 +
              D2ss K2 (βD2 - βS2) + K2 βS2 - I2s K2 βS2 + γ) μ1 μ2 (μ1 + P1 βI1 σC1) (μ2 +
              P2 βI2 σC2) ((-1 + D2ss + I2s) K2 P2 (-1 + x2) βI2 βS2 λ2 μ1 (μ1 + P1 βI1 σC1)
               σC2 + (-1 + D1ss + I1s) K1 P1 (-1 + x1) βI1 βS1 λ1 μ2 σC1 (μ2 + P2 βI2 σC2)) +
          a (K1 λ1 μ2 ((-1 + D1ss + I1s) βS1 μ1 + I1s (-1 + x1) βI1 σC1 (μ1 + P1 βI1 σC1))
                (μ2 + P2 βI2 σC2) + K2 λ2 μ1 (μ1 + P1 βI1 σC1)
                ((-1 + D2ss + I2s) βS2 μ2 + I2s (-1 + x2) βI2 σC2 (μ2 + P2 βI2 σC2)))^2))) /
    (2 (I1s K1 βI1 + I2s K2 βI2 + D1ss K1 (βD1 - βS1) + K1 βS1 - I1s K1 βS1 +
        D2ss K2 (βD2 - βS2) + K2 βS2 - I2s K2 βS2 + γ)
      μ1 μ2 (μ1 + P1 βI1 σC1) (μ2 + P2 βI2 σC2)))}
```

The spectral bound condition is

$$R_m = \left(\beta_{S_1}\left(1 - \hat{I}_{1,s} - \hat{D}_{1,s,s}\right)\right) \Big/ \left(\left(\beta_{S_1}\left(1 - \hat{I}_{1,s} - \hat{D}_{1,s,s}\right) + \beta_{I_1} \hat{I}_{1,s} + \beta_{D_1} \hat{D}_{1,s,s}\right) K_1 + \left(\beta_{S_2}\left(1 - \hat{I}_{2,s} - \hat{D}_{2,s,s}\right) + \beta_{I_2} \hat{I}_{2,s} + \beta_{D_2} \hat{D}_{2,s,s}\right) K_2 + \gamma\right)$$

$$\left(\frac{\mu_1}{\mu_1+\sigma_{C_1} \beta_{I_1} \hat{P}_1} \frac{a \lambda_1 K_1}{\mu_1} + \frac{\sigma_{C_1} \beta_{I_1} \hat{P}_1}{\mu_1+\sigma_{C_1} \beta_{I_1} \hat{P}_1} \frac{a (1-x_1) \lambda_1 K_1}{\mu_1}\right) + \left(\left(\beta_{I_1} \hat{I}_{1,s}\right) \Big/ \left(\left(\beta_{S_1}\left(1 - \hat{I}_{1,s} - \hat{D}_{1,s,s}\right) + \beta_{I_1} \hat{I}_{1,s} + \beta_{D_1} \hat{D}_{1,s,s}\right) K_1 + \left(\beta_{S_2}\left(1 - \hat{I}_{2,s} - \hat{D}_{2,s,s}\right) + \beta_{I_2} \hat{I}_{2,s} + \beta_{D_2} \hat{D}_{2,s,s}\right) K_2 + \gamma\right)\right) \frac{a (1-x_1) \lambda_1 K_1}{\mu_1} +$$

$$\left(\beta_{S_2}\left(1 - \hat{I}_{2,s} - \hat{D}_{2,s,s}\right)\right) \Big/ \left(\left(\beta_{S_1}\left(1 - \hat{I}_{1,s} - \hat{D}_{1,s,s}\right) + \beta_{I_1} \hat{I}_{1,s} + \beta_{D_1} \hat{D}_{1,s,s}\right) K_1 + \right.$$

[illegible]

This expression has the same biological interpretation as Eq. (12) in the main text.

In[106]:=
```
(* The condition for instability of the generalist-
  free equilibrium is that the spectral bound > 1 *)
- ((-a K2 βS2 λ2 μ1^2 μ2 + a D2ss K2 βS2 λ2 μ1^2 μ2 + a I2s K2 βS2 λ2 μ1^2 μ2 - a K1 βS1 λ1 μ1 μ2^2 +
        a D1ss K1 βS1 λ1 μ1 μ2^2 + a I1s K1 βS1 λ1 μ1 μ2^2 - a K2 P1 βI1 βS2 λ2 μ1 μ2 σC1 +
        a D2ss K2 P1 βI1 βS2 λ2 μ1 μ2 σC1 + a I2s K2 P1 βI1 βS2 λ2 μ1 μ2 σC1 -
        a I1s K1 βI1 λ1 μ1 μ2^2 σC1 + a I1s K1 x1 βI1 λ1 μ1 μ2^2 σC1 - a I1s K1 P1 βI1^2 λ1 μ2^2 σC1^2 +
        a I1s K1 P1 x1 βI1^2 λ1 μ2^2 σC1^2 - a K1 P2 βI2 βS1 λ1 μ1 μ2 σC2 +
        a D1ss K1 P2 βI2 βS1 λ1 μ1 μ2 σC2 + a I1s K1 P2 βI2 βS1 λ1 μ1 μ2 σC2 -
        a I2s K2 βI2 λ2 μ1^2 μ2 σC2 + a I2s K2 x2 βI2 λ2 μ1^2 μ2 σC2 -
        a I1s K1 P2 βI1 βI2 λ1 μ1 μ2 σC1 σC2 + a I1s K1 P2 x1 βI1 βI2 λ1 μ1 μ2 σC1 σC2 -
        a I2s K2 P1 βI1 βI2 λ2 μ1 μ2 σC1 σC2 + a I2s K2 P1 x2 βI1 βI2 λ2 μ1 μ2 σC1 σC2 -
        a I1s K1 P1 P2 βI1^2 βI2 λ1 μ2 σC1^2 σC2 + a I1s K1 P1 P2 x1 βI1^2 βI2 λ1 μ2 σC1^2 σC2 -
        a I2s K2 P2 βI2^2 λ2 μ1^2 σC2^2 + a I2s K2 P2 x2 βI2^2 λ2 μ1^2 σC2^2 -
        a I2s K2 P1 P2 βI1 βI2^2 λ2 μ1 σC1 σC2^2 + a I2s K2 P1 P2 x2 βI1 βI2^2 λ2 μ1 σC1 σC2^2 -
        √(a (4 (I1s K1 βI1 + I2s K2 βI2 + D1ss K1 (βD1 - βS1) + K1 βS1 - I1s K1 βS1 + D2ss K2
                  (βD2 - βS2) + K2 βS2 - I2s K2 βS2 + γ) μ1 μ2 (μ1 + P1 βI1 σC1) (μ2 + P2 βI2
                  σC2) ((-1 + D2ss + I2s) K2 P2 (-1 + x2) βI2 βS2 λ2 μ1 (μ1 + P1 βI1 σC1) σC2 +
                 (-1 + D1ss + I1s) K1 P1 (-1 + x1) βI1 βS1 λ1 μ2 σC1 (μ2 + P2 βI2 σC2)) +
              a (K1 λ1 μ2 ((-1 + D1ss + I1s) βS1 μ1 + I1s (-1 + x1) βI1 σC1 (μ1 + P1 βI1 σC1))
                   (μ2 + P2 βI2 σC2) + K2 λ2 μ1 (μ1 + P1 βI1 σC1) ((-1 + D2ss + I2s) βS2 μ2 +
                    I2s (-1 + x2) βI2 σC2 (μ2 + P2 βI2 σC2)))^2))) /
    (2 (I1s K1 βI1 + I2s K2 βI2 + D1ss K1 (βD1 - βS1) + K1 βS1 - I1s K1 βS1 +
         D2ss K2 (βD2 - βS2) + K2 βS2 - I2s K2 βS2 + γ)
       μ1 μ2 (μ1 + P1 βI1 σC1) (μ2 + P2 βI2 σC2))) > 1;
(* Cross-multiplying *)
- ((-a K2 βS2 λ2 μ1^2 μ2 + a D2ss K2 βS2 λ2 μ1^2 μ2 + a I2s K2 βS2 λ2 μ1^2 μ2 - a K1 βS1 λ1 μ1 μ2^2 +
       a D1ss K1 βS1 λ1 μ1 μ2^2 + a I1s K1 βS1 λ1 μ1 μ2^2 - a K2 P1 βI1 βS2 λ2 μ1 μ2 σC1 +
       a D2ss K2 P1 βI1 βS2 λ2 μ1 μ2 σC1 + a I2s K2 P1 βI1 βS2 λ2 μ1 μ2 σC1 -
       a I1s K1 βI1 λ1 μ1 μ2^2 σC1 + a I1s K1 x1 βI1 λ1 μ1 μ2^2 σC1 - a I1s K1 P1 βI1^2 λ1 μ2^2 σC1^2 +
       a I1s K1 P1 x1 βI1^2 λ1 μ2^2 σC1^2 - a K1 P2 βI2 βS1 λ1 μ1 μ2 σC2 +
       a D1ss K1 P2 βI2 βS1 λ1 μ1 μ2 σC2 + a I1s K1 P2 βI2 βS1 λ1 μ1 μ2 σC2 -
       a I2s K2 βI2 λ2 μ1^2 μ2 σC2 + a I2s K2 x2 βI2 λ2 μ1^2 μ2 σC2 -
       a I1s K1 P2 βI1 βI2 λ1 μ1 μ2 σC1 σC2 + a I1s K1 P2 x1 βI1 βI2 λ1 μ1 μ2 σC1 σC2 -
       a I2s K2 P1 βI1 βI2 λ2 μ1 μ2 σC1 σC2 + a I2s K2 P1 x2 βI1 βI2 λ2 μ1 μ2 σC1 σC2 -
       a I1s K1 P1 P2 βI1^2 βI2 λ1 μ2 σC1^2 σC2 + a I1s K1 P1 P2 x1 βI1^2 βI2 λ1 μ2 σC1^2 σC2 -
       a I2s K2 P2 βI2^2 λ2 μ1^2 σC2^2 + a I2s K2 P2 x2 βI2^2 λ2 μ1^2 σC2^2 -
       a I2s K2 P1 P2 βI1 βI2^2 λ2 μ1 σC1 σC2^2 + a I2s K2 P1 P2 x2 βI1 βI2^2 λ2 μ1 σC1 σC2^2 -
       √(a (4 (I1s K1 βI1 + I2s K2 βI2 + D1ss K1 (βD1 - βS1) + K1 βS1 - I1s K1 βS1 +
                 D2ss K2 (βD2 - βS2) + K2 βS2 - I2s K2 βS2 + γ) μ1 μ2 (μ1 + P1 βI1 σC1) (μ2 +
                 P2 βI2 σC2) ((-1 + D2ss + I2s) K2 P2 (-1 + x2) βI2 βS2 λ2 μ1 (μ1 + P1 βI1 σC1)
                  σC2 + (-1 + D1ss + I1s) K1 P1 (-1 + x1) βI1 βS1 λ1 μ2 σC1 (μ2 + P2 βI2 σC2)) +
             a (K1 λ1 μ2 ((-1 + D1ss + I1s) βS1 μ1 + I1s (-1 + x1) βI1 σC1 (μ1 + P1 βI1 σC1))
                  (μ2 + P2 βI2 σC2) + K2 λ2 μ1 (μ1 + P1 βI1 σC1) ((-1 + D2ss + I2s) βS2 μ2 +
                   I2s (-1 + x2) βI2 σC2 (μ2 + P2 βI2 σC2)))^2)))) >
  (2 (I1s K1 βI1 + I2s K2 βI2 + D1ss K1 (βD1 - βS1) + K1 βS1 - I1s K1 βS1 +
       D2ss K2 (βD2 - βS2) + K2 βS2 - I2s K2 βS2 + γ)
     μ1 μ2 (μ1 + P1 βI1 σC1) (μ2 + P2 βI2 σC2));

(* Isolating the square root term *)
-√(a (4 (I1s K1 βI1 + I2s K2 βI2 + D1ss K1 (βD1 - βS1) + K1 βS1 - I1s K1 βS1 + D2ss K2
```

```
(βD2 - βS2) + K2 βS2 - I2s K2 βS2 + γ) μ1 μ2 (μ1 + P1 βI1 σC1) (μ2 + P2 βI2 σC2)
((-1 + D2ss + I2s) K2 P2 (-1 + x2) βI2 βS2 λ2 μ1 (μ1 + P1 βI1 σC1) σC2 +
(-1 + D1ss + I1s) K1 P1 (-1 + x1) βI1 βS1 λ1 μ2 σC1 (μ2 + P2 βI2 σC2)) +
a (K1 λ1 μ2 ((-1 + D1ss + I1s) βS1 μ1 + I1s (-1 + x1) βI1 σC1 (μ1 + P1 βI1 σC1))
(μ2 + P2 βI2 σC2) + K2 λ2 μ1 (μ1 + P1 βI1 σC1)
((-1 + D2ss + I2s) βS2 μ2 + I2s (-1 + x2) βI2 σC2 (μ2 + P2 βI2 σC2)))^2)) >
(2 (I1s K1 βI1 + I2s K2 βI2 + D1ss K1 (βD1 - βS1) + K1 βS1 - I1s K1 βS1 +
D2ss K2 (βD2 - βS2) + K2 βS2 - I2s K2 βS2 + γ)
μ1 μ2 (μ1 + P1 βI1 σC1) (μ2 + P2 βI2 σC2)) -
(-((-a K2 βS2 λ2 μ1^2 μ2 + a D2ss K2 βS2 λ2 μ1^2 μ2 + a I2s K2 βS2 λ2 μ1^2 μ2 -
a K1 βS1 λ1 μ1 μ2^2 + a D1ss K1 βS1 λ1 μ1 μ2^2 + a I1s K1 βS1 λ1 μ1 μ2^2 -
a K2 P1 βI1 βS2 λ2 μ1 μ2 σC1 + a D2ss K2 P1 βI1 βS2 λ2 μ1 μ2 σC1 +
a I2s K2 P1 βI1 βS2 λ2 μ1 μ2 σC1 - a I1s K1 βI1 λ1 μ1 μ2^2 σC1 +
a I1s K1 x1 βI1 λ1 μ1 μ2^2 σC1 - a I1s K1 P1 βI1^2 λ1 μ2^2 σC1^2 +
a I1s K1 P1 x1 βI1^2 λ1 μ2^2 σC1^2 - a K1 P2 βI2 βS1 λ1 μ1 μ2 σC2 +
a D1ss K1 P2 βI2 βS1 λ1 μ1 μ2 σC2 + a I1s K1 P2 βI2 βS1 λ1 μ1 μ2 σC2 -
a I2s K2 βI2 λ2 μ1^2 μ2 σC2 + a I2s K2 x2 βI2 λ2 μ1^2 μ2 σC2 -
a I1s K1 P2 βI1 βI2 λ1 μ1 μ2 σC1 σC2 + a I1s K1 P2 x1 βI1 βI2 λ1 μ1 μ2 σC1 σC2 -
a I2s K2 P1 βI1 βI2 λ2 μ1 μ2 σC1 σC2 + a I2s K2 P1 x2 βI1 βI2 λ2 μ1 μ2 σC1 σC2 -
a I1s K1 P1 P2 βI1^2 βI2 λ1 μ2 σC1^2 σC2 + a I1s K1 P1 P2 x1 βI1^2 βI2 λ1 μ2 σC1^2 σC2 -
a I2s K2 P2 βI2^2 λ2 μ1^2 σC2^2 + a I2s K2 P2 x2 βI2^2 λ2 μ1^2 σC2^2 -
a I2s K2 P1 P2 βI1 βI2^2 λ2 μ1 σC1 σC2^2 + a I2s K2 P1 P2 x2 βI1 βI2^2 λ2 μ1 σC1 σC2^2)));

(* Squaring both sides and simplifying, the condition becomes: *)
(-√(a (4 (I1s K1 βI1 + I2s K2 βI2 + D1ss K1 (βD1 - βS1) + K1 βS1 - I1s K1 βS1 + D2ss K2 (βD2 -
βS2) + K2 βS2 - I2s K2 βS2 + γ) μ1 μ2 (μ1 + P1 βI1 σC1) (μ2 + P2 βI2 σC2)
((-1 + D2ss + I2s) K2 P2 (-1 + x2) βI2 βS2 λ2 μ1 (μ1 + P1 βI1 σC1) σC2 +
(-1 + D1ss + I1s) K1 P1 (-1 + x1) βI1 βS1 λ1 μ2 σC1 (μ2 + P2 βI2 σC2)) +
a (K1 λ1 μ2 ((-1 + D1ss + I1s) βS1 μ1 + I1s (-1 + x1) βI1 σC1 (μ1 + P1 βI1 σC1))
(μ2 + P2 βI2 σC2) + K2 λ2 μ1 (μ1 + P1 βI1 σC1) ((-1 + D2ss + I2s) βS2 μ2 +
I2s (-1 + x2) βI2 σC2 (μ2 + P2 βI2 σC2)))^2)))^2 >
((2 (I1s K1 βI1 + I2s K2 βI2 + D1ss K1 (βD1 - βS1) + K1 βS1 - I1s K1 βS1 +
D2ss K2 (βD2 - βS2) + K2 βS2 - I2s K2 βS2 + γ)
μ1 μ2 (μ1 + P1 βI1 σC1) (μ2 + P2 βI2 σC2)) -
(-((-a K2 βS2 λ2 μ1^2 μ2 + a D2ss K2 βS2 λ2 μ1^2 μ2 + a I2s K2 βS2 λ2 μ1^2 μ2 -
a K1 βS1 λ1 μ1 μ2^2 + a D1ss K1 βS1 λ1 μ1 μ2^2 + a I1s K1 βS1 λ1 μ1 μ2^2 -
a K2 P1 βI1 βS2 λ2 μ1 μ2 σC1 + a D2ss K2 P1 βI1 βS2 λ2 μ1 μ2 σC1 +
a I2s K2 P1 βI1 βS2 λ2 μ1 μ2 σC1 - a I1s K1 βI1 λ1 μ1 μ2^2 σC1 + a I1s K1 x1 βI1
λ1 μ1 μ2^2 σC1 - a I1s K1 P1 βI1^2 λ1 μ2^2 σC1^2 + a I1s K1 P1 x1 βI1^2 λ1 μ2^2 σC1^2 -
a K1 P2 βI2 βS1 λ1 μ1 μ2 σC2 + a D1ss K1 P2 βI2 βS1 λ1 μ1 μ2 σC2 +
a I1s K1 P2 βI2 βS1 λ1 μ1 μ2 σC2 - a I2s K2 βI2 λ2 μ1^2 μ2 σC2 +
a I2s K2 x2 βI2 λ2 μ1^2 μ2 σC2 - a I1s K1 P2 βI1 βI2 λ1 μ1 μ2 σC1 σC2 +
a I1s K1 P2 x1 βI1 βI2 λ1 μ1 μ2 σC1 σC2 - a I2s K2 P1 βI1 βI2 λ2 μ1 μ2 σC1 σC2 +
a I2s K2 P1 x2 βI1 βI2 λ2 μ1 μ2 σC1 σC2 - a I1s K1 P1 P2 βI1^2 βI2 λ1 μ2 σC1^2 σC2 +
a I1s K1 P1 P2 x1 βI1^2 βI2 λ1 μ2 σC1^2 σC2 - a I2s K2 P2 βI2^2 λ2 μ1^2 σC2^2 +
a I2s K2 P2 x2 βI2^2 λ2 μ1^2 σC2^2 - a I2s K2 P1 P2 βI1 βI2^2 λ2 μ1 σC1 σC2^2 +
a I2s K2 P1 P2 x2 βI1 βI2^2 λ2 μ1 σC1 σC2^2))))^2 // Simplify
```

In[123]:=
```
(* Dividing the positive coefficient, the condition becomes *)
((I1s K1 βI1 + I2s K2 βI2 + D1ss K1 (βD1 - βS1) + K1 βS1 - I1s K1 βS1 +
      D2ss K2 (βD2 - βS2) + K2 βS2 - I2s K2 βS2 + γ) μ1 μ2 (μ1 + P1 βI1 σC1)
    (μ2 + P2 βI2 σC2) + a (K1 λ1 μ2 (I1s (-1 + x1) βI1 σC1 (μ1 + P1 βI1 σC1) +
        (-1 + D1ss + I1s) βS1 (μ1 - P1 (-1 + x1) βI1 σC1)) (μ2 + P2 βI2 σC2) +
      K2 λ2 μ1 (μ1 + P1 βI1 σC1) (I2s (-1 + x2) βI2 σC2 (μ2 + P2 βI2 σC2) +
        (-1 + D2ss + I2s) βS2 (μ2 - P2 (-1 + x2) βI2 σC2)))) < 0;
(* Simplifying, the condition becomes *)
(I1s K1 βI1 + I2s K2 βI2 + D1ss K1 (βD1 - βS1) + K1 βS1 - I1s K1 βS1 +
    D2ss K2 (βD2 - βS2) + K2 βS2 - I2s K2 βS2 + γ) μ1 μ2 (μ1 + P1 βI1 σC1)
  (μ2 + P2 βI2 σC2) < (a (K1 λ1 μ2 (I1s (1 - x1) βI1 σC1 (μ1 + P1 βI1 σC1) +
       (1 - D1ss - I1s) βS1 (μ1 + P1 (1 - x1) βI1 σC1)) (μ2 + P2 βI2 σC2) +
     K2 λ2 μ1 (μ1 + P1 βI1 σC1) (I2s (1 - x2) βI2 σC2 (μ2 + P2 βI2 σC2) +
       (1 - D2ss - I2s) βS2 (μ2 + P2 (1 - x2) βI2 σC2))));

(* Dividing through, the condition becomes *)
(1 / ((I1s K1 βI1 + I2s K2 βI2 + D1ss K1 (βD1 - βS1) + K1 βS1 - I1s K1 βS1 + D2ss K2 (βD2 - βS2) +
       K2 βS2 - I2s K2 βS2 + γ) μ1 μ2 (μ1 + P1 βI1 σC1) (μ2 + P2 βI2 σC2)))
  (a (K1 λ1 μ2 (I1s (1 - x1) βI1 σC1 (μ1 + P1 βI1 σC1) + (1 - D1ss - I1s)
         βS1 (μ1 + P1 (1 - x1) βI1 σC1)) (μ2 + P2 βI2 σC2) +
     K2 λ2 μ1 (μ1 + P1 βI1 σC1) (I2s (1 - x2) βI2 σC2 (μ2 + P2 βI2 σC2) +
       (1 - D2ss - I2s) βS2 (μ2 + P2 (1 - x2) βI2 σC2)))) > 1;
(* This expression is equivalent to *)
Rm = (((1 - I1s - D1ss) βS1) / (((1 - I1s - D1ss) βS1 + I1s βI1 + D1ss βD1) K1 +
        ((1 - I2s - D2ss) βS2 + I2s βI2 + D2ss βD2) K2 + γ) )
    (μ1/(μ1 + P1 βI1 σC1) a λ1 K1/μ1 + P1 βI1 σC1/(μ1 + P1 βI1 σC1) a (1 - x1) λ1 K1/μ1) +
  ((I1s βI1 σC1) / (((1 - I1s - D1ss) βS1 + I1s βI1 + D1ss βD1) K1 +
        ((1 - I2s - D2ss) βS2 + I2s βI2 + D2ss βD2) K2 + γ)) a (1 - x1) λ1 K1/μ1 +
  (((1 - I2s - D2ss) βS2) / (((1 - I1s - D1ss) βS1 + I1s βI1 + D1ss βD1) K1 +
        ((1 - I2s - D2ss) βS2 + I2s βI2 + D2ss βD2) K2 + γ) )
    (μ2/(μ2 + P2 βI2 σC2) a λ2 K2/μ2 + P2 βI2 σC2/(μ2 + P2 βI2 σC2) a (1 - x2) λ2 K2/μ2) +
  ((I2s βI2 σC2) / (((1 - I1s - D1ss) βS1 + I1s βI1 + D1ss βD1) K1 +
        ((1 - I2s - D2ss) βS2 + I2s βI2 + D2ss βD2) K2 + γ) ) a (1 - x2) λ2 K2/μ2;
Rm ==
  (1 / ((I1s K1 βI1 + I2s K2 βI2 + D1ss K1 (βD1 - βS1) + K1 βS1 - I1s K1 βS1 + D2ss K2
          (βD2 - βS2) + K2 βS2 - I2s K2 βS2 + γ) μ1 μ2 (μ1 + P1 βI1 σC1) (μ2 + P2 βI2 σC2)))
   (a (K1 λ1 μ2 (I1s (1 - x1) βI1 σC1 (μ1 + P1 βI1 σC1) + (1 - D1ss - I1s) βS1
          (μ1 + P1 (1 - x1) βI1 σC1)) (μ2 + P2 βI2 σC2) +
       K2 λ2 μ1 (μ1 + P1 βI1 σC1) (I2s (1 - x2) βI2 σC2 (μ2 + P2 βI2 σC2) +
         (1 - D2ss - I2s) βS2 (μ2 + P2 (1 - x2) βI2 σC2)))) // Simplify
```

Out[127]=
```
True
```

Calculating the response of R_m for Cases 7-10 in Table 2

Case 7: Two specialist parasites; no coinfection, constant host population size; avoidance of non-susceptible hosts

Using the R_m expression calculated above, to create this scenario we let $\beta_{I_1} = \beta_{I_2} = \beta_{C_1} = \beta_{C_2} = \beta_{D_1} = \beta_{D_2} = 0$ and $D_{1,s,s} = D_{2,s,s} = 0$. In this case, R_m simplifes to

$$R_m = \frac{\beta_{S_1}\left(1-\hat{I_{1,s}}\right)}{\beta_{S_1}\left(1-\hat{I_{1,s}}\right)K_1+\beta_{S_2}\left(1-\hat{I_{2,s}}\right)K_2+\gamma}\left(\frac{a\,\lambda_1\,K_1}{\mu_1}\right)+\frac{\beta_{S_2}\left(1-\hat{I_{2,s}}\right)}{\beta_{S_1}\left(1-\hat{I_{1,s}}\right)K_1+\beta_{S_2}\left(1-\hat{I_{2,s}}\right)K_2+\gamma}\left(\frac{a\,\lambda_2\,K_2}{\mu_2}\right) > 1.$$

In[130]:=
```
Rm /. {βI1 → 0, βI2 → 0, βC1 → 0, βC2 → 0, βD1 → 0, βD2 → 0, D1ss → 0, D2ss → 0}
```

Out[130]=
```
(a (1 - I1s) K1 βS1 λ1) / (((1 - I1s) K1 βS1 + (1 - I2s) K2 βS2 + γ) μ1) +
  (a (1 - I2s) K2 βS2 λ2) / (((1 - I1s) K1 βS1 + (1 - I2s) K2 βS2 + γ) μ2)
```

In the absence of the generalist parasite, the equilibrium prevalences of infection are $I_{1,r} = 1 - \frac{\gamma\,\mu_1}{\beta_{S_1}\,K_1(\lambda_1-\mu_1)}$ and $I_{2,r} = 1 - \frac{\gamma\,\mu_2}{\beta_{S_2}\,K_2(\lambda_2-\mu_2)}$, implying that the equilibrium number of susceptibles are $\frac{\gamma\,\mu_1}{\beta_{S_1}\,K_1(\lambda_1-\mu_1)}$ and $\frac{\gamma\,\mu_2}{\beta_{S_2}\,K_2(\lambda_2-\mu_2)}$.

In[137]:=
```
(* Solving for the equilibria of the I1,s-P1 system *)
Solve[{(dI1sdt /. {C1sg → 0, D1ss → 0, I1g → 0} /.
      {βI1 → 0, βI2 → 0, βC1 → 0, βC2 → 0, βD1 → 0, βD2 → 0, D1ss → 0, D2ss → 0}) == 0,
   (dP1dt /. {C1sg → 0, D1ss → 0, I1g → 0} /. {βI1 → 0, βI2 → 0, βC1 → 0,
       βC2 → 0, βD1 → 0, βD2 → 0, D1ss → 0, D2ss → 0}) == 0}, {I1s, P1}]
(* Solving for the equilibria of the I2,s-P2 system *)
Solve[{(dI2sdt /. {C2sg → 0, D2ss → 0, I2g → 0} /.
      {βI2 → 0, βI2 → 0, βC2 → 0, βC2 → 0, βD2 → 0, βD2 → 0, D2ss → 0, D2ss → 0}) == 0,
   (dP2dt /. {C2sg → 0, D2ss → 0, I2g → 0} /. {βI1 → 0, βI2 → 0, βC1 → 0,
       βC2 → 0, βD1 → 0, βD2 → 0, D1ss → 0, D2ss → 0}) == 0}, {I2s, P2}]
```

Out[137]= $\left\{\{I1s \to 0,\ P1 \to 0\},\ \left\{I1s \to \frac{K1\,\beta S1\,\lambda 1 - K1\,\beta S1\,\mu 1 - \gamma\,\mu 1}{K1\,\beta S1\,(\lambda 1 - \mu 1)},\ P1 \to \frac{K1\,\beta S1\,\lambda 1 - K1\,\beta S1\,\mu 1 - \gamma\,\mu 1}{\beta S1\,\gamma}\right\}\right\}$

Out[138]= $\left\{\{I2s \to 0,\ P2 \to 0\},\ \left\{I2s \to \frac{K2\,\beta S2\,\lambda 2 - K2\,\beta S2\,\mu 2 - \gamma\,\mu 2}{K2\,\beta S2\,(\lambda 2 - \mu 2)},\ P2 \to \frac{K2\,\beta S2\,\lambda 2 - K2\,\beta S2\,\mu 2 - \gamma\,\mu 2}{\beta S2\,\gamma}\right\}\right\}$

We can plug these equilibria into the R_m expression and arrive at a very simple expression for $R_m = \frac{a\,(2\,\lambda_1\,\lambda_2-\lambda_2\,\mu_1-\lambda_1\,\mu_2)}{\lambda_1\,\lambda_2-\mu_1\,\mu_2}$.

In[141]:=
```
(* Plug equilibria into Rm and simplify *)
Rm2 = Rm /. {βI1 → 0, βI2 → 0, βC1 → 0, βC2 → 0, βD1 → 0, βD2 → 0, D1ss → 0, D2ss → 0} /.
    {I1s → (K1 βS1 λ1 - K1 βS1 μ1 - γ μ1)/(K1 βS1 (λ1 - μ1)), I2s → (K2 βS2 λ2 - K2 βS2 μ2 - γ μ2)/(K2 βS2 (λ2 - μ2))} // Simplify
```

Out[141]= $\frac{a\,(2\,\lambda 1\,\lambda 2 - \lambda 2\,\mu 1 - \lambda 1\,\mu 2)}{\lambda 1\,\lambda 2 - \mu 1\,\mu 2}$

We can use this expression to investigate how changing host body size or temperature will affect R_m. If we account for the allometric relationships underlying λ and μ, we can calculate the partial derivative $\partial R_m/\partial W$ for both endoparasites and ectoparasites.

For endoparasites, $\frac{\partial R_m}{\partial W} = \frac{a\left(\lambda_1\,\mu_1(\lambda_2-\mu_2)^2+\lambda_2\,\mu_2(\lambda_1-\mu_1)^2\right)}{W(\lambda_1\,\lambda_2-\mu_1\,\mu_2)^2} > 0$, indicating that increasing body size will make it easier for the generalist to invade.

In[145]:=
```
(* Taking derivatives with respect to body size for an endoparasite *)
D[Rm2 /. {λ1 → λ1[W], λ2 → λ2[W], μ1 → μ1[W], μ2 → μ2[W]}, W] /.
  {λ1'[W] → (3 λ1[W])/(4 W), λ2'[W] → (3 λ2[W])/(4 W), μ1'[W] → (-μ1[W])/(4 W), μ2'[W] → (-μ2[W])/(4 W)} // Simplify
```

Out[145]=
```
(a (λ1[W]^2 λ2[W] μ2[W] + λ2[W] μ1[W]^2 μ2[W] +
    λ1[W] μ1[W] (λ2[W]^2 - 4 λ2[W] μ2[W] + μ2[W]^2))) / (W (λ1[W] λ2[W] - μ1[W] μ2[W])^2)
```

In[151]:=
```
(* This expression can be simplified somewhat
 by rewriting the numerator in the following way *)
(λ1[W]^2 λ2[W] μ2[W] + λ2[W] μ1[W]^2 μ2[W] + λ1[W] μ1[W] (λ2[W]^2 - 4 λ2[W] μ2[W] + μ2[W]^2)) ==
  (λ2[W] μ2[W] (λ1[W] - μ1[W])^2 + λ1[W] μ1[W] (λ2[W] - μ2[W])^2) // Simplify
```

Out[151]= True

For ectoparasites, $\frac{\partial R_m}{\partial W} = \frac{2\,a\,(\lambda_1\,\mu_1(\lambda_2-\mu_2)^2+\lambda_2\,\mu_2(\lambda_1-\mu_1)^2)}{3\,W(\lambda_1\,\lambda_2-\mu_1\,\mu_2)^2} > 0$, again indicating that increasing body size will make it easier for the generalist to invade.

In[152]:=
```
(* Taking derivatives with respect to body size for an endoparasite *)
D[Rm2 /. {λ1 → λ1[W], λ2 → λ2[W], μ1 → μ1[W], μ2 → μ2[W]}, W] /.
  {λ1'[W] → (5 λ1[W])/(12 W), λ2'[W] → (5 λ2[W])/(12 W), μ1'[W] → (-μ1[W])/(4 W), μ2'[W] → (-μ2[W])/(4 W)} // Simplify
```

Out[152]=
```
(2 a (λ1[W]^2 λ2[W] μ2[W] + λ2[W] μ1[W]^2 μ2[W] +
    λ1[W] μ1[W] (λ2[W]^2 - 4 λ2[W] μ2[W] + μ2[W]^2))) / (3 W (λ1[W] λ2[W] - μ1[W] μ2[W])^2)
```

In[153]:=
```
(* This expression can be simplified somewhat
 by rewriting the numerator in the following way *)
(λ1[W]^2 λ2[W] μ2[W] + λ2[W] μ1[W]^2 μ2[W] + λ1[W] μ1[W] (λ2[W]^2 - 4 λ2[W] μ2[W] + μ2[W]^2)) ==
  (λ2[W] μ2[W] (λ1[W] - μ1[W])^2 + λ1[W] μ1[W] (λ2[W] - μ2[W])^2) // Simplify
```

Out[153]= True

For both endoparasites and ectoparasites, increasing f will increase R_m (because increasing f increases the body size of the second host, so $\lambda_2'(f) > 0$ and $\mu_2'(f) < 0$).

```
(* Take the derivative with respect to f *)
D[Rm2 /. {λ2 → λ2[f], μ2 → μ2[f]}, f] // Simplify
```

Out[155]=
```
(a (λ1 - μ1)^2 (μ2[f] λ2′[f] - λ2[f] μ2′[f])) / (λ1 λ2[f] - μ1 μ2[f])^2
```

For both endoparasites and ectoparasites, there will be no change in R_m with temperature, because R_m is independent of environmental temperature.

```
(* Take the derivative with respect to T *)
D[Rm2 /. {λ1 → λ1[T], λ2 → λ2[T], μ1 → μ1[T], μ2 → μ2[T]}, T] /. {μ1'[T] → E/(k T^2) μ1[T],
   λ1'[T] → E/(k T^2) λ1[T], μ2'[T] → E/(k T^2) μ2[T], λ2'[T] → E/(k T^2) λ2[T]} // Simplify
```

Out[156]= 0

The Jacobian matrix for the full system is:

```
J = {{D[dI1rdt, I1r], D[dI1rdt, P1r], D[dI1rdt, I2r],
     D[dI1rdt, P2r], D[dI1rdt, I1m], D[dI1rdt, I2m], D[dI1rdt, P12m]},
    {D[dP1rdt, I1r], D[dP1rdt, P1r], D[dP1rdt, I2r], D[dP1rdt, P2r],
     D[dP1rdt, I1m], D[dP1rdt, I2m], D[dP1rdt, P12m]},
    {D[dI2rdt, I1r], D[dI2rdt, P1r], D[dI2rdt, I2r], D[dI2rdt, P2r],
     D[dI2rdt, I1m], D[dI2rdt, I2m], D[dI2rdt, P12m]},
    {D[dP2rdt, I1r], D[dP2rdt, P1r], D[dP2rdt, I2r], D[dP2rdt, P2r],
     D[dP2rdt, I1m], D[dP2rdt, I2m], D[dP2rdt, P12m]},
    {D[dI1mdt, I1r], D[dI1mdt, P1r], D[dI1mdt, I2r], D[dI1mdt, P2r],
     D[dI1mdt, I1m], D[dI1mdt, I2m], D[dI1mdt, P12m]},
    {D[dI2mdt, I1r], D[dI2mdt, P1r], D[dI2mdt, I2r], D[dI2mdt, P2r],
     D[dI2mdt, I1m], D[dI2mdt, I2m], D[dI2mdt, P12m]},
    {D[dP12mdt, I1r], D[dP12mdt, P1r], D[dP12mdt, I2r], D[dP12mdt, P2r], D[dP12mdt,
      I1m], D[dP12mdt, I2m], D[dP12mdt, P12m]}} /. {I1m → 0, I2m → 0, P12m → 0};
MatrixForm[
 J]
```

$$\begin{pmatrix} -P1r\,\beta1-\mu1 & (1-I1r)\,\beta1 & 0 & 0 & -P1r\,\beta1 & 0 \\ K1\,P1r\,\beta1+K1\,\lambda1 & -(1-I1r)\,K1\,\beta1-\gamma & 0 & 0 & K1\,P1r\,\beta1 & 0 \\ 0 & 0 & -P2r\,\beta2-\mu & (1-I2r)\,\beta2 & 0 & -P2r\,\beta\text{[illegible]} \\ 0 & 0 & K2\,P2r\,\beta2+K2\,\lambda2 & -(1-I2r)\,K2\,\beta2-\gamma & 0 & K2\,P2r\,\text{[illegible]} \\ 0 & 0 & 0 & 0 & -\mu1 & 0 \\ 0 & 0 & 0 & 0 & 0 & -\mu2 \\ 0 & 0 & 0 & 0 & a\,K1\,\lambda1 & a\,K2\,\lambda2 \end{pmatrix}$$

As before, this has a upper block triangular structure, and whether the generalist can invade is entirely determined by the bottom left submatrix:

```
MatrixForm[J[[5 ;; 7, 5 ;; 7]]]
```

$$\begin{pmatrix} -\mu1 & 0 & (1-I1r)\,\beta1 \\ 0 & -\mu2 & (1-I2r)\,\beta2 \\ a\,K1\,\lambda1 & a\,K2\,\lambda2 & -(1-I1r)\,K1\,\beta1-(1-I2r)\,K2\,\beta2-\gamma \end{pmatrix}$$

Applying the next generation matrix theorem, the generalist will be able to invade if the eigenvalue is greater than 1.

```
F = {{0, 0, (1 - I1r) β1}, {0, 0, (1 - I2r) β2}, {a λ1 K1, a λ2 K2, 0}};
V = {{μ1, 0, 0}, {0, μ2, 0}, {0, 0, (1 - I1r) K1 β1 + (1 - I2r) K2 β2 + γ}};
Eigenvalues[Dot[F, Inverse[V]]] // Simplify
```

$$\left\{0,\ -\left(\left(\sqrt{a}\,\sqrt{((-1+I2r)\,K2\,\beta2\,\lambda2\,\mu1+(-1+I1r)\,K1\,\beta1\,\lambda1\,\mu2)}\right)\Big/\left(\sqrt{((-1+I1r)\,K1\,\beta1+(-1+I2r)\,K2\,\beta2-\gamma)}\,\sqrt{\mu1}\,\sqrt{\mu2}\right)\right),\ \left(\sqrt{a}\,\sqrt{((-1+I2r)\,K2\,\beta2\,\lambda2\,\mu1+(-1+I1r)\,K1\,\beta1\,\lambda1\,\mu2)}\right)\Big/\left(\sqrt{((-1+I1r)\,K1\,\beta1+(-1+I2r)\,K2\,\beta2-\gamma)}\,\sqrt{\mu1}\,\sqrt{\mu2}\right)\right\}$$

This condition can be simplified to
$\frac{\beta_1 K_1(a\lambda_1-\mu_1)}{\gamma\mu_1}(1-I_{1,r})+\frac{\beta_2 K_2(a\lambda_2-\mu_2)}{\gamma\mu_2}(1-I_{2,r})>1$,
which, after plugging in the generalist-free endemic equilibria for $I_{1,r}$ and $I_{2,r}$, simplifies to
$\frac{a\lambda_1-\mu_1}{\lambda_1-\mu_1}+\frac{a\lambda_2-\mu_2}{\lambda_2-\mu_2}>1$

$$\text{Simplify}\left[\frac{\beta1\ K1\ (a\ \lambda1 - \mu1)}{\gamma\ \mu1}\ (1 - I1r)\ /.\ \left\{I1r \to \frac{K1\ \beta1\ \lambda1 - K1\ \beta1\ \mu1 - \gamma\ \mu1}{K1\ \beta1\ \lambda1 - K1\ \beta1\ \mu1}\right\}\right]$$

$$\text{Simplify}\left[\frac{\beta2\ K2\ (a\ \lambda2 - \mu2)}{\gamma\ \mu2}\ (1 - I2r)\ /.\ \left\{I2r \to \frac{K2\ \beta2\ \lambda2 - K2\ \beta2\ \mu2 - \gamma\ \mu2}{K2\ \beta2\ \lambda2 - K2\ \beta2\ \mu2}\right\}\right]$$

$$\frac{a\ \lambda1 - \mu1}{\lambda1 - \mu1}$$

$$\frac{a\ \lambda2 - \mu2}{\lambda2 - \mu2}$$

We can again see how changing host body size and temperature will affect invasion by looking at the derivatives of the invasion condition with respect to body size and temperature, after substituting in the allometric scaling relationships. Note that now you have a contravailing pressure of increasing body size on invasion fitness for the generalist: increasing host body size increases shedding rate, but decreases carrying capacity.

For an endoparasite, the derivative of the invasion fitness with respect to host body size is always positive because $\frac{dR_0}{dW} = \frac{(1-a)}{W}\left(\frac{\lambda_1 \mu_1}{(\lambda_1-\mu_1)^2} + \frac{\lambda_2 \mu_2}{(\lambda_2-\mu_2)^2}\right)$.

$$\left(D\left[\frac{a\ \lambda1[W] - \mu1[W]}{\lambda1[W] - \mu1[W]} + \frac{a\ \lambda2[W]\ - \mu2[W]}{\lambda2[W] - \mu2[W]}, W\right]\ /.\ \left\{\mu1'[W] \to \frac{-\mu1[W]}{4\ W}, \lambda1'[W] \to \frac{3\ \lambda1[W]}{4\ W}, \mu2'[W] \to \frac{-\mu2[W]}{4\ W}, \lambda2'[W] \to \frac{3\ \lambda2[W]}{4\ W}\right\}\right) == \frac{(1 - a)}{W}\left(\frac{\lambda1[W]\ \mu1[W]}{(\lambda1[W] - \mu1[W])^2} + \frac{\lambda2[W]\ \mu2[W]}{(\lambda2[W] - \mu2[W])^2}\right)\ //\ \text{Simplify}$$

True

Similarly, for an ectoparasite, the derivative of the invasion fitness with respect to host body size is always positive beacuse $\frac{dR_0}{dW} = \frac{2\,(1-a)}{3\,W}\left(\frac{\lambda_1 \mu_1}{(\lambda_1-\mu_1)^2} + \frac{\lambda_2 \mu_2}{(\lambda_2-\mu_2)^2}\right)$.

$$\text{Simplify}\left[D\left[\frac{a\ \lambda1[W] - \mu1[W]}{\lambda1[W] - \mu1[W]} + \frac{a\ \lambda2[W] - \mu2[W]}{\lambda2[W] - \mu2[W]}, W\right]\ /.\ \left\{\mu1'[W] \to \frac{-\mu1[W]}{4\ W}, \lambda1'[W] \to \frac{5\ \lambda1[W]}{12\ W}, \mu2'[W] \to \frac{-\mu2[W]}{4\ W}, \lambda2'[W] \to \frac{5\ \lambda2[W]}{12\ W}\right\}\right] == \frac{2\ (1 - a)}{3\ W}\left(\frac{\lambda1[W]\ \mu1[W]}{(\lambda1[W] - \mu1[W])^2} + \frac{\lambda2[W]\ \mu2[W]}{(\lambda2[W] - \mu2[W])^2}\right)\ //\ \text{Simplify}$$

True

For both endoparasites and ectoparasites, the derivative of the invasion fitness with respect to temperature is zero, as before.

$$D\left[\frac{a\ \lambda1[T]\ - \mu1[T]}{\lambda1[T]\ - \mu1[T]} + \frac{a\ \lambda2[T]\ - \mu2[T]}{\lambda2[T]\ - \mu2[T]}, T\right]\ /.\ \left\{\mu2'[T] \to \frac{E\ \mu2[T]}{k\ T^2}, \lambda2'[T] \to \frac{E\ \lambda2[T]}{k\ T^2}, \mu1'[T] \to \frac{E\ \mu1[T]}{k\ T^2}, \lambda1'[T] \to \frac{E\ \lambda1[T]}{k\ T^2}\right\}\ //\ \text{Simplify}$$

0

Case 8: Two specialist parasites; no coinfection, constant host population size;

avoidance of non-susceptible hosts

Using the R_m expression calculated above, to create this scenario we let $\sigma_{C_1} = \sigma_{C_2} = \sigma_{D_1} = \sigma_{D_2} = \beta_{C_1} = \beta_{C_2} = \beta_{D_1} = \beta_{D_2} = 0$ and $D_{1,s,s} = D_{2,s,s} = 0$. In this case, R_m simplifes to

$$R_m = \left(\beta_{S_1}\left(1 - \hat{I_{1,s}}\right) \Big/ \left(\left(\beta_{S_1}\left(1 - \hat{I_{1,s}}\right) + \beta_{I_1}\hat{I_{1,s}}\right)K_1 + \left(\beta_{S_2}\left(1 - \hat{I_{2,s}}\right) + \beta_{I_2}\hat{I_{2,s}}\right)K_2 + \gamma\right)\right)\left(\frac{a\lambda_1 K_1}{\mu_1}\right) + \left(\beta_{S_2}\left(1 - \hat{I_{2,s}}\right) \Big/ \left(\left(\beta_{S_1}\left(1 - \hat{I_{1,s}}\right) + \beta_{I_1}\hat{I_{1,s}}\right)K_1 + \left(\beta_{S_2}\left(1 - \hat{I_{2,s}}\right) + \beta_{I_2}\hat{I_{2,s}}\right)K_2 + \gamma\right)\right)\left(\frac{a\lambda_2 K_2}{\mu_2}\right) > 1.$$

```
In[166]:= Rm /. {σC1 → 0, σC2 → 0, σD1 → 0, σD2 → 0,
   βC1 → 0, βC2 → 0, βD1 → 0, βD2 → 0, D1ss → 0, D2ss → 0}

Out[166]= (a (1 - I1s) K1 βS1 λ1) / ((K1 (I1s βI1 + (1 - I1s) βS1) + K2 (I2s βI2 + (1 - I2s) βS2) + γ) μ1) +
  (a (1 - I2s) K2 βS2 λ2) / ((K1 (I1s βI1 + (1 - I1s) βS1) + K2 (I2s βI2 + (1 - I2s) βS2) + γ) μ2)
```

In the absence of the generalist parasite, the equilibrium prevalences of infection are $I_{1,r} = \frac{K_1\beta_{S_1}(\lambda_1 - \mu_1) - \gamma\mu_1}{K_1\beta_{S_1}(\lambda_1 - \mu_1) + K_1\beta_{I_1}\mu_1}$ and $I_{2,r} = \frac{K_2\beta_{S_2}(\lambda_2 - \mu_2) - \gamma\mu_2}{K_2\beta_{S_2}(\lambda_2 - \mu_2) + K_2\beta_{I_2}\mu_2}$, implying that the equilibrium number of susceptibles are $\frac{\gamma\mu_1}{\beta_{S_1}K_1(\lambda_1 - \mu_1)}$ and $\frac{\gamma\mu_2}{\beta_{S_2}K_2(\lambda_2 - \mu_2)}$.

```
In[167]:= (* Solving for the equilibria of the I1,s-P1 system *)
Solve[
  {(dI1sdt /. {C1sg → 0, D1ss → 0, I1g → 0} /. {σC1 → 0, σC2 → 0, σD1 → 0, σD2 → 0, βC1 → 0,
        βC2 → 0, βD1 → 0, βD2 → 0, D1ss → 0, D2ss → 0}) == 0,
   (dP1dt /. {C1sg → 0, D1ss → 0, I1g → 0} /. {σC1 → 0, σC2 → 0, σD1 → 0, σD2 → 0, βC1 → 0,
        βC2 → 0, βD1 → 0, βD2 → 0, D1ss → 0, D2ss → 0}) == 0}, {I1s, P1}] // Simplify
(* Solving for the equilibria of the I2,s-P2 system *)
Solve[
  {(dI2sdt /. {C2sg → 0, D2ss → 0, I2g → 0} /. {σC1 → 0, σC2 → 0, σD1 → 0, σD2 → 0, βC2 → 0,
        βC2 → 0, βD2 → 0, βD2 → 0, D2ss → 0, D2ss → 0}) == 0,
   (dP2dt /. {C2sg → 0, D2ss → 0, I2g → 0} /. {σC1 → 0, σC2 → 0, σD1 → 0, σD2 → 0, βC1 → 0,
        βC2 → 0, βD1 → 0, βD2 → 0, D1ss → 0, D2ss → 0}) == 0}, {I2s, P2}] // Simplify
```

Out[167]= $\left\{\{I1s \to 0,\ P1 \to 0\},\ \left\{I1s \to \frac{K1\,\beta S1\,\lambda 1 - K1\,\beta S1\,\mu 1 - \gamma\,\mu 1}{K1\,\beta S1\,\lambda 1 + K1\,\beta I1\,\mu 1 - K1\,\beta S1\,\mu 1},\ P1 \to \frac{K1\,\beta S1\,\lambda 1 - K1\,\beta S1\,\mu 1 - \gamma\,\mu 1}{K1\,\beta I1\,\beta S1 + \beta S1\,\gamma}\right\}\right\}$

Out[168]= $\left\{\{I2s \to 0,\ P2 \to 0\},\ \left\{I2s \to \frac{K2\,\beta S2\,\lambda 2 - K2\,\beta S2\,\mu 2 - \gamma\,\mu 2}{K2\,\beta S2\,\lambda 2 + K2\,\beta I2\,\mu 2 - K2\,\beta S2\,\mu 2},\ P2 \to \frac{K2\,\beta S2\,\lambda 2 - K2\,\beta S2\,\mu 2 - \gamma\,\mu 2}{K2\,\beta I2\,\beta S2 + \beta S2\,\gamma}\right\}\right\}$

We can plug these equilibria into the R_m expression, although that doesn't help to simplify the expression very much. If, however, we also make the assumption that contact rates are equal across host classes (e.g., $\beta_{S_1} = \beta_{I_1} = \beta_1$ and $\beta_{S_2} = \beta_{I_2} = \beta_2$), then the expression simplifies considerably, and $R_m = \frac{a(\beta_1 K_1 + \beta_2 K_2 + 2\gamma)}{\beta_1 K_1 + \beta_2 K_2 + \gamma} = a\left(1 - \frac{2\gamma}{\beta_1 K_1 + \beta_2 K_2 + \gamma}\right)$.

From this expression, it is immediately clear how changing body sizes or temperature will affect R_m, because R_m depends only on the abundances of each host. Since increasing body size reduces abundance, $\frac{\partial R_m}{\partial W} > 0$. Since increasing temperature decreases abundance, $\frac{\partial R_m}{\partial W} > 0$. This will be true for both endoparasites and ectoparasites.

```
(* Plug equilibria into Rm and simplify *)
Rm2 =
 Rm /. {σC1 → 0, σC2 → 0, σD1 → 0, σD2 → 0, βC1 → 0, βC2 → 0, βD1 → 0, βD2 → 0, D1ss → 0,
     D2ss → 0} /. {I1s → (K1 βS1 λ1 - K1 βS1 μ1 - γ μ1) / (K1 βS1 λ1 + K1 βI1 μ1 - K1 βS1 μ1),
    I2s → (K2 βS2 λ2 - K2 βS2 μ2 - γ μ2) / (K2 βS2 λ2 + K2 βI2 μ2 - K2 βS2 μ2)} // Simplify
(* Consider the special case where contact rates are equal
 within each host species *)
Rm2 = Rm /. {σC1 → 0, σC2 → 0, σD1 → 0, σD2 → 0, βC1 → 0, βC2 → 0, βD1 → 0, βD2 → 0, D1ss → 0,
      D2ss → 0} /. {I1s → (K1 βS1 λ1 - K1 βS1 μ1 - γ μ1) / (K1 βS1 λ1 + K1 βI1 μ1 - K1 βS1 μ1),
     I2s → (K2 βS2 λ2 - K2 βS2 μ2 - γ μ2) / (K2 βS2 λ2 + K2 βI2 μ2 - K2 βS2 μ2)} /.
   {βS1 → β1, βS2 → β2, βI1 → β1, βI2 → β2} // Simplify
```

Out[179]=
```
(a (K2 βI2 βS2 λ2 (βS1 (λ1 - μ1) + βI1 μ1) + K1 βI1 βS1 λ1 (βS2 (λ2 - μ2) + βI2 μ2) +
      γ (βI1 βS2 λ2 μ1 + βS1 (2 βS2 λ1 λ2 - βS2 λ2 μ1 + βI2 λ1 μ2 - βS2 λ1 μ2)))) /
  (K2 βI2 βS2 λ2 (βS1 (λ1 - μ1) + βI1 μ1) + K1 βI1 βS1 λ1 (βS2 (λ2 - μ2) + βI2 μ2) +
    γ (βI1 (-βI2 + βS2) μ1 μ2 + βS1 (βS2 λ1 λ2 + βI2 μ1 μ2 - βS2 μ1 μ2)))
```

Out[180]=
$$\frac{a\,(K1\,\beta 1 + K2\,\beta 2 + 2\,\gamma)}{K1\,\beta 1 + K2\,\beta 2 + \gamma}$$

Case 9: Two specialist parasites; coinfection, constant host population size; avoidance of non-susceptible hosts

Using the R_m expression calculated above, to create this scenario we let $\beta_{C_1} = \beta_{C_2} = \beta_{D_1} = \beta_{D_2} = 0$ and $D_{1,s,s} = D_{2,s,s} = 0$. In this case, R_m simplifes to

$$R_m = \left(\beta_{S_1}\left(1 - \hat{I}_{1,s} - \hat{D}_{1,s,s}\right)\right) \Big/ \left(\left(\beta_{S_1}\left(1 - \hat{I}_{1,s} - \hat{D}_{1,s,s}\right) + \beta_{I_1} \hat{I}_{1,s}\right) K_1 + \left(\beta_{S_2}\left(1 - \hat{I}_{2,s} - \hat{D}_{2,s,s}\right) + \beta_{I_2} \hat{I}_{2,s}\right) K_2 + \gamma\right)\right)$$
$$\left(\frac{\mu_1}{\mu_1 + \sigma_{C_1} \beta_{I_1} \hat{P}_1} \frac{a \lambda_1 K_1}{\mu_1} + \frac{\sigma_{C_1} \beta_{I_1} \hat{P}_1}{\mu_1 + \sigma_{C_1} \beta_{I_1} \hat{P}_1} \frac{a (1 - x_1) \lambda_1 K_1}{\mu_1}\right) +$$
$$\left(\left(\beta_{I_1} \hat{I}_{1,s}\right) \Big/ \left(\left(\beta_{S_1}\left(1 - \hat{I}_{1,s} - \hat{D}_{1,s,s}\right) + \beta_{I_1} \hat{I}_{1,s}\right) K_1 + \left(\beta_{S_2}\left(1 - \hat{I}_{2,s} - \hat{D}_{2,s,s}\right) + \beta_{I_2} \hat{I}_{2,s}\right) K_2 + \gamma\right)\right) \frac{a (1 - x_1) \lambda_1 K_1}{\mu_1} +$$
$$\left(\beta_{S_2}\left(1 - \hat{I}_{2,s} - \hat{D}_{2,s,s}\right)\right) \Big/ \left(\left(\beta_{S_1}\left(1 - \hat{I}_{1,s} - \hat{D}_{1,s,s}\right) + \beta_{I_1} \hat{I}_{1,s}\right) K_1 + \left(\beta_{S_2}\left(1 - \hat{I}_{2,s} - \hat{D}_{2,s,s}\right) + \beta_{I_2} \hat{I}_{2,s}\right) K_2 + \gamma\right)\right)$$
$$\left(\frac{\mu_2}{\mu_2 + \sigma_{C_2} \beta_{I_2} \hat{P}_2} \frac{a \lambda_2 K_2}{\mu_2} + \frac{\sigma_{C_2} \beta_{I_2} \hat{P}_2}{\mu_2 + \sigma_{C_2} \beta_{I_2} \hat{P}_2} \frac{a (1 - x_2) \lambda_2 K_2}{\mu_2}\right) +$$
$$\left(\left(\beta_{I_2} \hat{I}_{2,s}\right) \Big/ \left(\left(\beta_{S_1}\left(1 - \hat{I}_{1,s} - \hat{D}_{1,s,s}\right) + \beta_{I_1} \hat{I}_{1,s}\right) K_1 + \left(\beta_{S_2}\left(1 - \hat{I}_{2,s} - \hat{D}_{2,s,s}\right) + \beta_{I_2} \hat{I}_{2,s}\right) K_2 + \gamma\right)\right) \frac{a (1 - x_2) \lambda_2 K_2}{\mu_2} > 1.$$

In[268]:=
```
Rm2 = Rm /. {βC1 → 0, βC2 → 0, βD1 → 0, βD2 → 0}
```

Out[268]=
```
(a I1s K1 (1 - x1) βI1 λ1 σC1) /
   ((K1 (I1s βI1 + (1 - D1ss - I1s) βS1) + K2 (I2s βI2 + (1 - D2ss - I2s) βS2) + γ) μ1) +
  ((1 - D1ss - I1s) βS1 (a K1 λ1 / (μ1 + P1 βI1 σC1) + a K1 P1 (1 - x1) βI1 λ1 σC1 / (μ1 (μ1 + P1 βI1 σC1)))) /
   (K1 (I1s βI1 + (1 - D1ss - I1s) βS1) + K2 (I2s βI2 + (1 - D2ss - I2s) βS2) + γ) +
  (a I2s K2 (1 - x2) βI2 λ2 σC2) /
   ((K1 (I1s βI1 + (1 - D1ss - I1s) βS1) + K2 (I2s βI2 + (1 - D2ss - I2s) βS2) + γ) μ2) +
  ((1 - D2ss - I2s) βS2 (a K2 λ2 / (μ2 + P2 βI2 σC2) + a K2 P2 (1 - x2) βI2 λ2 σC2 / (μ2 (μ2 + P2 βI2 σC2)))) /
   (K1 (I1s βI1 + (1 - D1ss - I1s) βS1) + K2 (I2s βI2 + (1 - D2ss - I2s) βS2) + γ)
```

In the absence of the generalist parasite, the equilibria can be found, but they are complex enough to

make analytical progress intractable.

In[260]:=
```
(* Solving for the equilibria of the I1,s-D1,s,s-P1 system *)
(* D1ss in terms of P1 and I1,s *)
D1ssEq = Solve[(dI1sdt /. {C1sg → 0, Pg → 0, I1g → 0}) == 0, D1ss];
(* I1,s in terms of P1 *)
 I1sEq = Solve[(dD1ssdt /. D1ssEq[[1]]) == 0, I1s]
(* D1,s,s in terms of P1 *)
D1ssEq = Simplify[D1ssEq /. I1sEq[[1]]]
(* P1 equilibrium *)
P1Eq =
 Solve[(dP1dt /. {C1sg → 0, Pg → 0, I1g → 0} /. I1sEq[[1]] /. D1ssEq[[1]]) == 0, P1] //
  Simplify
(* Solving for the equilibria of the I2,s-D2,s,s-P2 system *)
(* D2ss in terms of P2 and I2,s *)
D2ssEq = Solve[(dI2sdt /. {C2sg → 0, Pg → 0, I2g → 0}) == 0, D2ss];
(* I2,s in terms of P2 *)
 I2sEq = Solve[(dD2ssdt /. D2ssEq[[1]]) == 0, I2s]
(* D2,s,s in terms of P2 *)
D2ssEq = Simplify[D2ssEq /. I2sEq[[1]]]
(* P2 equilibrium *)
P2Eq =
 Solve[(dP2dt /. {C2sg → 0, Pg → 0, I2g → 0} /. I2sEq[[1]] /. D2ssEq[[1]]) == 0, P2] //
  Simplify
```

Out[261]= $\left\{\left\{\text{I1s} \to \frac{\text{P1}\,\beta\text{S1}\,\mu 1}{(\text{P1}\,\beta\text{S1}+\mu 1)\,(\mu 1+\text{P1}\,\beta\text{I1}\,\sigma\text{D1})}\right\}\right\}$

Out[262]= $\left\{\left\{\text{D1ss} \to \frac{\text{P1}^2\,\beta\text{I1}\,\beta\text{S1}\,\sigma\text{D1}}{(\text{P1}\,\beta\text{S1}+\mu 1)\,(\mu 1+\text{P1}\,\beta\text{I1}\,\sigma\text{D1})}\right\}\right\}$

Out[263]= $\{\{\text{P1}\to 0\},$

$\left\{\text{P1}\to -\frac{1}{2\,\beta\text{I1}\,\beta\text{S1}\,(\text{K1}\,\beta\text{D1}+\gamma)\,\sigma\text{D1}}\left(\text{K1}\,\beta\text{I1}\,\beta\text{S1}\,\mu 1+\beta\text{S1}\,\gamma\,\mu 1-\text{K1}\,\beta\text{I1}\,\beta\text{S1}\,\lambda 1\,\sigma\text{D1}+\text{K1}\,\beta\text{I1}\,\beta\text{S1}\,\mu 1\,\sigma\text{D1}+\beta\text{I1}\,\gamma\,\mu 1\,\sigma\text{D1}-\sqrt{\left(-4\,\beta\text{I1}\,\beta\text{S1}\,(\text{K1}\,\beta\text{D1}+\gamma)\,\mu 1\,(\gamma\,\mu 1+\text{K1}\,\beta\text{S1}\,(-\lambda 1+\mu 1))\,\sigma\text{D1}+(\gamma\,\mu 1\,(\beta\text{S1}+\beta\text{I1}\,\sigma\text{D1})+\text{K1}\,\beta\text{I1}\,\beta\text{S1}\,(\mu 1-\lambda 1\,\sigma\text{D1}+\mu 1\,\sigma\text{D1}))^2\right)}\right)\right\},$

$\left\{\text{P1}\to -\frac{1}{2\,\beta\text{I1}\,\beta\text{S1}\,(\text{K1}\,\beta\text{D1}+\gamma)\,\sigma\text{D1}}\left(\text{K1}\,\beta\text{I1}\,\beta\text{S1}\,\mu 1+\beta\text{S1}\,\gamma\,\mu 1-\text{K1}\,\beta\text{I1}\,\beta\text{S1}\,\lambda 1\,\sigma\text{D1}+\text{K1}\,\beta\text{I1}\,\beta\text{S1}\,\mu 1\,\sigma\text{D1}+\beta\text{I1}\,\gamma\,\mu 1\,\sigma\text{D1}+\sqrt{\left(-4\,\beta\text{I1}\,\beta\text{S1}\,(\text{K1}\,\beta\text{D1}+\gamma)\,\mu 1\,(\gamma\,\mu 1+\text{K1}\,\beta\text{S1}\,(-\lambda 1+\mu 1))\,\sigma\text{D1}+(\gamma\,\mu 1\,(\beta\text{S1}+\beta\text{I1}\,\sigma\text{D1})+\text{K1}\,\beta\text{I1}\,\beta\text{S1}\,(\mu 1-\lambda 1\,\sigma\text{D1}+\mu 1\,\sigma\text{D1}))^2\right)}\right)\right\}\}$

Out[265]= $\left\{\left\{\text{I2s} \to \frac{\text{P2}\,\beta\text{S2}\,\mu 2}{(\text{P2}\,\beta\text{S2}+\mu 2)\,(\mu 2+\text{P2}\,\beta\text{I2}\,\sigma\text{D2})}\right\}\right\}$

Out[266]= $\left\{\left\{\text{D2ss} \to \frac{\text{P2}^2\,\beta\text{I2}\,\beta\text{S2}\,\sigma\text{D2}}{(\text{P2}\,\beta\text{S2}+\mu 2)\,(\mu 2+\text{P2}\,\beta\text{I2}\,\sigma\text{D2})}\right\}\right\}$

Out[267]= $\{\{P2 \to 0\},$
$\{P2 \to -\frac{1}{2\,\beta I2\,\beta S2\,(K2\,\beta D2+\gamma)\,\sigma D2}\big(K2\,\beta I2\,\beta S2\,\mu 2+\beta S2\,\gamma\,\mu 2-K2\,\beta I2\,\beta S2\,\lambda 2\,\sigma D2+K2\,\beta I2\,\beta S2\,\mu 2\,\sigma D2+\beta I2\,\gamma\,\mu 2\,\sigma D2-\sqrt{\left(-4\,\beta I2\,\beta S2\,(K2\,\beta D2+\gamma)\,\mu 2\,(\gamma\,\mu 2+K2\,\beta S2\,(-\lambda 2+\mu 2))\,\sigma D2+(\gamma\,\mu 2\,(\beta S2+\beta I2\,\sigma D2)+K2\,\beta I2\,\beta S2\,(\mu 2-\lambda 2\,\sigma D2+\mu 2\,\sigma D2))^2\right)}\big)\},$
$\{P2 \to -\frac{1}{2\,\beta I2\,\beta S2\,(K2\,\beta D2+\gamma)\,\sigma D2}\big(K2\,\beta I2\,\beta S2\,\mu 2+\beta S2\,\gamma\,\mu 2-K2\,\beta I2\,\beta S2\,\lambda 2\,\sigma D2+K2\,\beta I2\,\beta S2\,\mu 2\,\sigma D2+\beta I2\,\gamma\,\mu 2\,\sigma D2+\sqrt{\left(-4\,\beta I2\,\beta S2\,(K2\,\beta D2+\gamma)\,\mu 2\,(\gamma\,\mu 2+K2\,\beta S2\,(-\lambda 2+\mu 2))\,\sigma D2+(\gamma\,\mu 2\,(\beta S2+\beta I2\,\sigma D2)+K2\,\beta I2\,\beta S2\,(\mu 2-\lambda 2\,\sigma D2+\mu 2\,\sigma D2))^2\right)}\big)\}\}$

We can proceed numerically, however. We consider the cases of endoparasites and ectoparasites separately.

Endoparasites:

Many of the parameters of the model are set by the allometric relationships (Gillooly et al. 2001, Allen et al. 2002, Savage et al. 2004, Poulin & George-Nascimento 2007), and thus can be set to biologically reasonable values.

In[350]:=
$$\text{allom} = \{K1 \to K0\,\text{Exp}[\tfrac{E}{k\,T}]\,W^{-3/4},\ K2 \to K0\,\text{Exp}[\tfrac{E}{k\,T}]\,(f\,W)^{-3/4},\ \mu 1 \to \mu 0\,\text{Exp}[-\tfrac{E}{k\,T}]\,W^{-1/4},\ \mu 2 \to \mu 0\,\text{Exp}[-\tfrac{E}{k\,T}]\,(f\,W)^{-1/4},\ \lambda 1 \to \lambda 0\,\text{Exp}[-\tfrac{E}{k\,T}]\,W^{3/4},\ \lambda 2 \to \lambda 0\,\text{Exp}[-\tfrac{E}{k\,T}]\,(f\,W)^{3/4},\ r1 \to r0\,\text{Exp}[-\tfrac{E}{k\,T}]\,W^{-1/4},\ r2 \to r0\,\text{Exp}[-\tfrac{E}{k\,T}]\,(f\,W)^{-1/4}\};$$
$$\text{allompars} = \{k \to \tfrac{8.617`}{10^5},\ K0 \to \tfrac{2.984`}{10^9},\ \mu 0 \to 1.785` \times 10^8,\ \lambda 0 \to 2 \times 10^8,\ r0 \to 2.21 \times 10^{10},\ E \to 0.43\};$$

That leaves a much smaller subset of parameters that can be varied. For simplicity, we focus on parameters whose effects on invasion fitness are indeterminate. This excludes parameters related to the costs associated with generalism, including *c* (the reduction in shedding rate for generalists), σ_1 (the probability of coinfection, which hold constant at 1), and x_1 (the fraction of host resources captured by the resident strains in coinfection). None of these parameters affect the equilibrium abundances of susceptible and singly infected hosts, so their effect on R_m are predictable and obvious - reducing *c,* reducing σ_1, or increasing x_1 will all reduce R_m, making invasion more difficult. Thus the only parameters to explore (other than host mass *W* and temperature *T*), are β (the contact rate between hosts and parasites, which is assumed to be dependent on the parasite and thus equal for both hosts) and γ (the loss rate of parasites from the environment).

Varying *W* and β:

In[352]:=
```
RmSimp = Rm2 /. I1sEq[[1]] /. I2sEq[[1]] /. D1ssEq[[1]] /. D2ssEq[[1]] /. P1Eq[[2]] /.
    P2Eq[[2]] /. allom /. allompars;
```

In[333]:=
```
RmAcrossWB =
  Table[Table[RmSimp /. allom /. allompars /. {βS1 → B, βS2 → B, βI1 → B, βI2 → B,
      βD1 → B, βD2 → B, σD1 → 1, σD2 → 1, σC1 → 1, σC2 → 1, γ → 0.1, W → Wval, T → 270,
      f → 0.9, a → 0.8, x1 → 1 / 2, x2 → 1 / 2}, {Wval, 25, 1000, 25}], {B, 0.2, 1, 0.2}];
```

Interestingly, here we see a complex response of R_m to increasing mass (Fig. S45). The response is highly dependent on the value of β: when β is large, increasing mass decreases R_m. However, when β is small, increasing *W* increases R_m.

In[334]:=
```
Labeled[ListLinePlot[Table[Table[
    {Table[W, {W, 25, 1000, 25}][[i]], RmAcrossWB[[j, i]]}, {i, 1, 40}], {j, 1, 5}],
  PlotLegends → {"β=0.2", "β=0.4", "β=0.6", "β=0.8", "β=1.0"}, PlotLabel →
   "Fig. S45. Effect of body size W on Rm \nas the contact rate β is varied",
  PlotRange → All],
 {"Host mass W", "Generalist Rm"}, {Bottom, Left}, RotateLabel → True]
```

Out[334]=

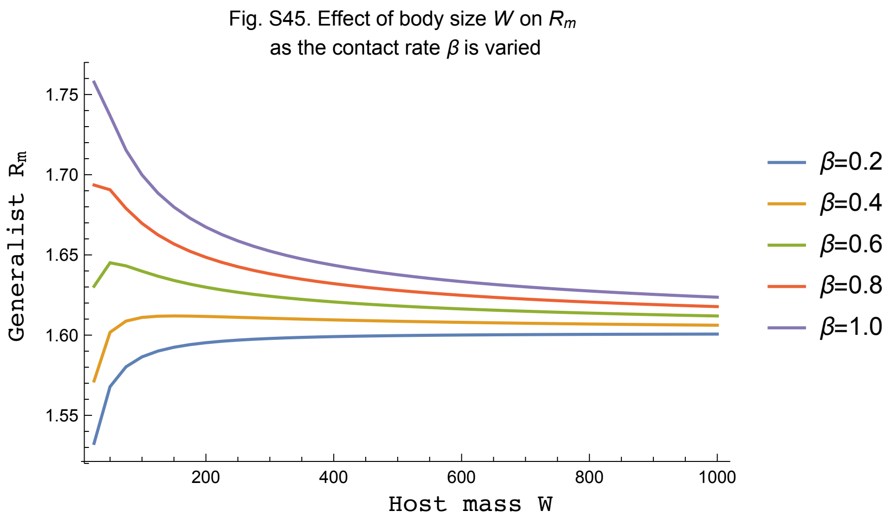

Varying *W* and *γ*:

In[335]:=
```
RmAcrossWg = Table[
   Table[RmSimp /. allom /. allompars /. {βS1 → 1, βS2 → 1, βI1 → 1, βI2 → 1, βD1 → 1,
      βD2 → 1, σD1 → 1, σD2 → 1, σC1 → 1, σC2 → 1, γ → g, W → Wval, T → 270, f → 0.9,
      a → 0.8, x1 → 1 / 2, x2 → 1 / 2}, {Wval, 25, 1000, 25}], {g, 0.02, 0.1, 0.02}];
```

Now, increasing host body size decreases R_m (Fig. S46) across the range of *γ* values.

In[338]:=
```
Labeled[ListLinePlot[Table[Table[
     {Table[W, {W, 25, 1000, 25}][[i]], RmAcrossWg[[j, i]]}, {i, 1, 40}], {j, 1, 5}],
   PlotLegends → {"γ=0.02", "γ=0.04", "γ=0.06", "γ=0.08", "γ=0.1"}, PlotLabel →
    "Fig. S46. Effect of body size W on Rm \nas the loss rate γ is varied",
   PlotRange → All],
  {"Host mass W", "Generalist Rm"}, {Bottom, Left}, RotateLabel → True]
```

Out[338]=

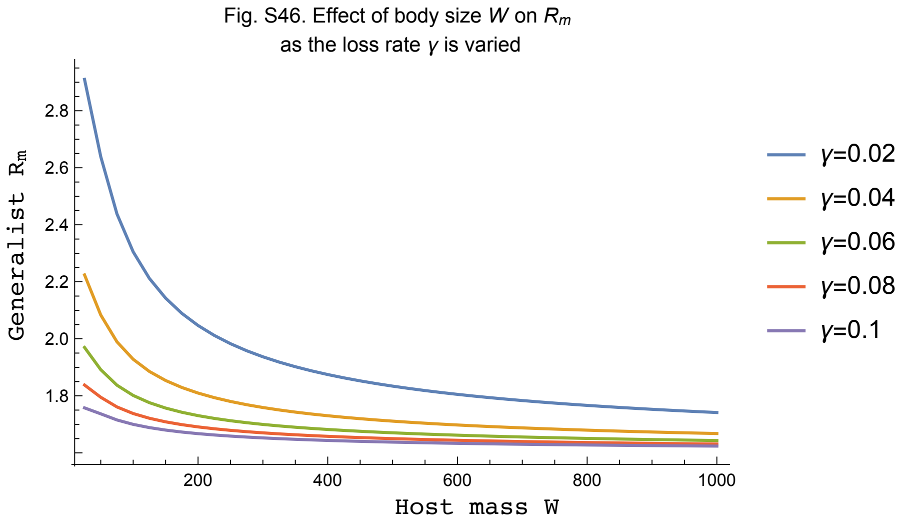

The effect of mass and temperature:

In[339]:=
```
RmAcrossWT =
  Table[Table[RmSimp /. allom /. allompars /. {βS1 → 1, βS2 → 1, βI1 → 1, βI2 → 1,
      βD1 → 1, βD2 → 1, σD1 → 1, σD2 → 1, σC1 → 1, σC2 → 1, γ → 0.05, σ2 → 1,
      W → Wval, T → Tval, σ1 → 1, f → 0.9, a → 0.8, x1 → 1 / 2, x2 → 1 / 2},
    {Wval, 25, 1000, 25}], {Tval, 270, 310, 10}];
```

Again, we see a complex relationship between body mass and R_m, but increasing temperature always decreases R_m, regardless of the value of W (Fig. S47).

In[340]:=
```
Labeled[ListLinePlot[Table[Table[
    {Table[W, {W, 25, 1000, 25}][[i]], RmAcrossWT[[j, i]]}, {i, 1, 40}], {j, 1, 5}],
  PlotLegends → {"T=270", "T=280", "T=290", "T=300", "T=310"}, PlotLabel →
   "Fig. S47. Effect of body size W on Rm \nas the temperature T is varied"],
 {"Host mass W", "Generalist Rm"}, {Bottom, Left}, RotateLabel → True]
```

Out[340]=

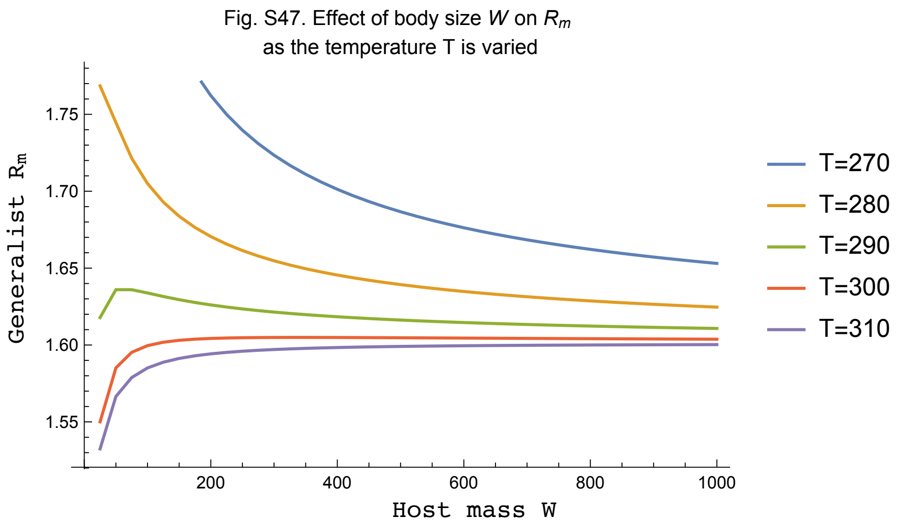

Ectoparasites:

Many of the parameters of the model are set by the allometric relationships (Gillooly et al. 2001, Allen et al. 2002, Savage et al. 2004, Poulin & George-Nascimento 2007), and thus can be set to biologically reasonable values.

In[357]:=
```
allom = {K1 → K0 Exp[E/(k T)] W^(-3/4), K2 → K0 Exp[E/(k T)] (f W)^(-3/4),
   μ1 → μ0 Exp[-E/(k T)] W^(-1/4), μ2 → μ0 Exp[-E/(k T)] (f W)^(-1/4), λ1 → λ0 Exp[-E/(k T)] W^(5/12),
   λ2 → λ0 Exp[-E/(k T)] (f W)^(5/12), r1 → r0 Exp[-E/(k T)] W^(-1/4), r2 → r0 Exp[-E/(k T)] (f W)^(-1/4)};
allompars = {k → 8.617`/10^5, K0 → 2.984`/10^9, μ0 → 1.785` × 10^8,
   λ0 → 2 × 10^8, r0 → 2.21 × 10^10, E → 0.43};
```

That leaves a much smaller subset of parameters that can be varied. For simplicity, we focus on parameters whose effects on invasion fitness are indeterminate. This excludes parameters related to the costs associated with generalism, including *c* (the reduction in shedding rate for generalists), σ_1 (the probability of coinfection, which hold constant at 1), and x_1 (the fraction of host resources captured by the resident strains in coinfection). None of these parameters affect the equilibrium abundances of susceptible and singly infected hosts, so their effect on R_m are predictable and obvious - reducing *c,* reducing σ_1, or increasing x_1 will all reduce R_m, making invasion more difficult. Thus the only parameters to explore (other than host mass *W* and temperature *T*), are β (the contact rate between hosts and parasites, which is assumed to be dependent on the parasite and thus equal for both hosts) and γ (the loss rate of parasites from the environment).

Varying *W* and β:

```
In[359]:= RmSimp = Rm2 /. I1sEq[[1]] /. I2sEq[[1]] /. D1ssEq[[1]] /. D2ssEq[[1]] /. P1Eq[[2]] /.
      P2Eq[[2]] /. allom /. allompars;
```

```
In[360]:= RmAcrossWB =
   Table[Table[RmSimp /. allom /. allompars /. {βS1 → B, βS2 → B, βI1 → B, βI2 → B,
      βD1 → B, βD2 → B, σD1 → 1, σD2 → 1, σC1 → 1, σC2 → 1, γ → 0.1, W → Wval, T → 270,
      f → 0.9, a → 0.8, x1 → 1 / 2, x2 → 1 / 2}, {Wval, 25, 1000, 25}], {B, 2, 10, 2}];
```

Interestingly, here we see a unimodal response of R_m to increasing mass (Fig. S48).

```
In[361]:= Labeled[ListLinePlot[
   Table[Table[{Table[W, {W, 25, 1000, 25}][[i]], RmAcrossWB[[j, i]]}, {i, 1, 40}],
    {j, 1, 5}], PlotLegends → {"β=2", "β=4", "β=6", "β=8", "β=10"}, PlotLabel →
    "Fig. S48. Effect of body size W on Rm \nas the contact rate β is varied",
   PlotRange → All],
  {"Host mass W", "Generalist Rm"}, {Bottom, Left}, RotateLabel → True]
```

Out[361]=

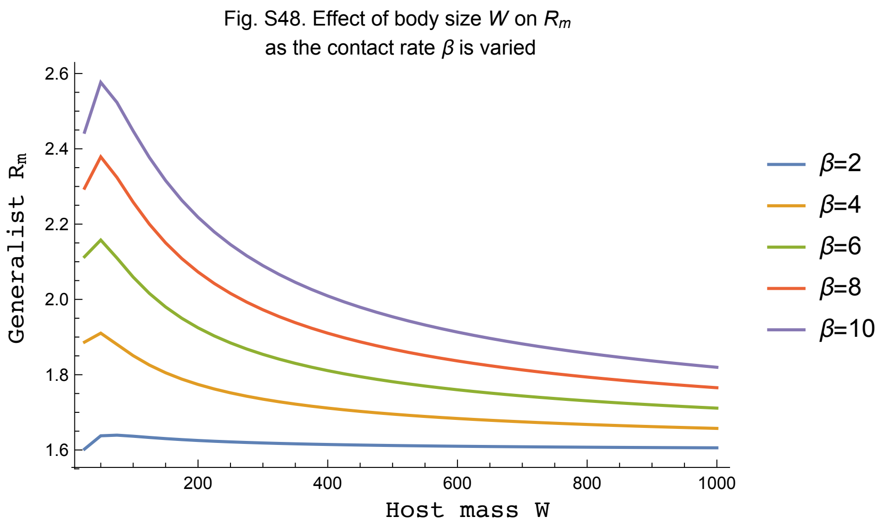

Varying W and γ:

```
In[367]:= RmAcrossWg = Table[
    Table[RmSimp /. allom /. allompars /. {βS1 → 5, βS2 → 5, βI1 → 5, βI2 → 5, βD1 → 5,
      βD2 → 5, σD1 → 1, σD2 → 1, σC1 → 1, σC2 → 1, γ → g, W → Wval, T → 270, f → 0.9,
      a → 0.8, x1 → 1 / 2, x2 → 1 / 2}, {Wval, 25, 1000, 25}], {g, 0.02, 0.1, 0.02}];
```

We see a similar unimodal response of R_m to increasing host body size (Fig. S49) across the range of γ values.

In[369]:=
```
Labeled[ListLinePlot[Table[Table[
    {Table[W, {W, 25, 1000, 25}][[i]], RmAcrossWg[[j, i]]}, {i, 1, 40}], {j, 1, 5}],
  PlotLegends → {"γ=0.02", "γ=0.04", "γ=0.06", "γ=0.08", "γ=0.1"}, PlotLabel →
   "Fig. S49. Effect of body size W on Rm \nas the loss rate γ is varied",
  PlotRange → All],
 {"Host mass W", "Generalist Rm"}, {Bottom, Left}, RotateLabel → True]
```

Out[369]=

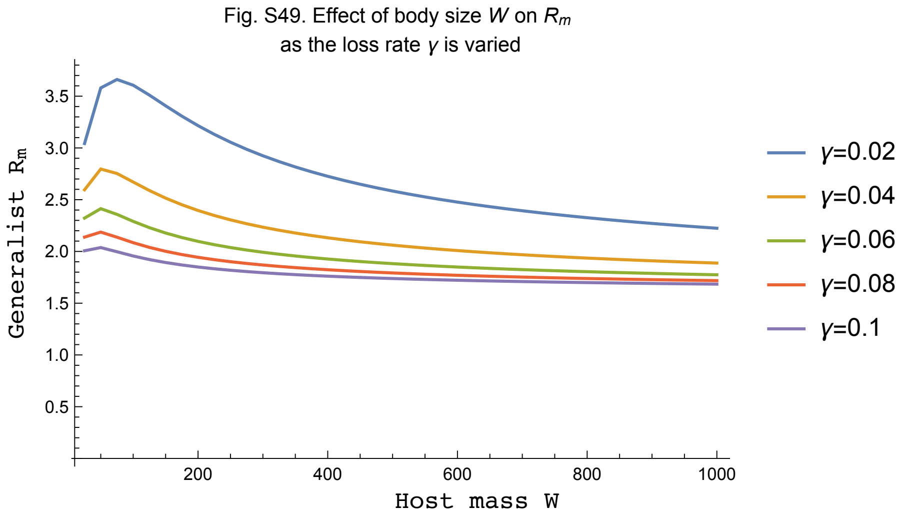

The effect of mass and temperature:

In[370]:=
```
RmAcrossWT =
  Table[Table[RmSimp /. allom /. allompars /. {βS1 → 5, βS2 → 5, βI1 → 5, βI2 → 5,
      βD1 → 5, βD2 → 5, σD1 → 1, σD2 → 1, σC1 → 1, σC2 → 1, γ → 0.05, σ2 → 1,
      W → Wval, T → Tval, σ1 → 1, f → 0.9, a → 0.8, x1 → 1 / 2, x2 → 1 / 2},
    {Wval, 25, 1000, 25}], {Tval, 270, 310, 10}];
```

Again, we see a complex relationship between body mass and R_m, but increasing temperature always decreases R_m, regardless of the value of W (Fig. S50).

In[371]:=
```
Labeled[ListLinePlot[Table[Table[
    {Table[W, {W, 25, 1000, 25}][[i]], RmAcrossWT[[j, i]]}, {i, 1, 40}], {j, 1, 5}],
  PlotLegends → {"T=270", "T=280", "T=290", "T=300", "T=310"}, PlotLabel →
   "Fig. S50. Effect of body size W on Rm \nas the temperature T is varied"],
 {"Host mass W", "Generalist Rm"}, {Bottom, Left}, RotateLabel → True]
```

Out[371]=

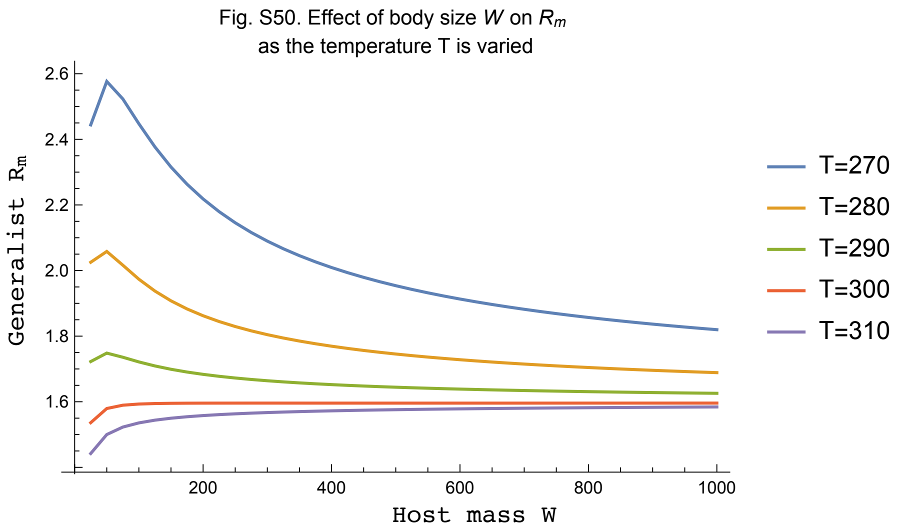

Case 10: Two specialist parasites; coinfection, constant host population size; no avoidance of non-susceptible hosts

Here we cannot simplify the R_m expression unless we are willing to make some simplifying assumptions. In particular, if we assume that contact rates are host species-specific, but not host class-specific, so $\beta_{S_1} = \beta_{I_1} = \beta_{D_1} = \beta_1$ and $\beta_{S_2} = \beta_{I_2} = \beta_{D_2} = \beta_2$, then we can get a lot of nice simplification.

$$R_m = \frac{\beta_1\left(1-\hat{I}_{1,s}-\hat{D}_{1,s,s}\right)}{\beta_1 K_1+\beta_2 K_2+\gamma}\left(\frac{\mu_1}{\mu_1+\sigma_{C_1}\beta_1\hat{P}_1}\frac{a\lambda_1 K_1}{\mu_1}+\frac{\sigma_{C_1}\beta_1\hat{P}_1}{\mu_1+\sigma_{C_1}\beta_1\hat{P}_1}\frac{a(1-x_1)\lambda_1 K_1}{\mu_1}\right)+\frac{\beta_1\hat{I}_{1,s}}{\beta_1 K_1+\beta_2 K_2+\gamma}\frac{a(1-x_1)\lambda_1 K_1}{\mu_1}+$$
$$\frac{\beta_2\left(1-\hat{I}_{2,s}-\hat{D}_{2,s,s}\right)}{\beta_2 K_1+\beta_2 K_2+\gamma}\left(\frac{\mu_2}{\mu_2+\sigma_{C_2}\beta_2\hat{P}_2}\frac{a\lambda_2 K_2}{\mu_2}+\frac{\sigma_{C_2}\beta_2\hat{P}_2}{\mu_2+\sigma_{C_2}\beta_2\hat{P}_2}\frac{a(1-x_2)\lambda_2 K_2}{\mu_2}\right)+\frac{\beta_2\hat{I}_{2,s}}{\beta_1 K_1+\beta_2 K_2+\gamma}\frac{a(1-x_2)\lambda_2 K_2}{\mu_2}$$

In[373]:=
```
Rm2 = Rm /. {βS1 → β1, βI1 → β1, βD1 → β1, βS2 → β2, βI2 → β2, βD2 → β2} // Simplify
```

Out[373]=

$$\left(a\left(-\frac{\mathrm{I1s}\,\mathrm{K1}\,(-1+\mathrm{x1})\,\beta 1\,\lambda 1\,\sigma\mathrm{C1}}{\mu 1}+((-1+\mathrm{D1ss}+\mathrm{I1s})\,\mathrm{K1}\,\beta 1\,\lambda 1\,(-\mu 1+\mathrm{P1}\,(-1+\mathrm{x1})\,\beta 1\,\sigma\mathrm{C1}))\,/\,(\mu 1\,(\mu 1+\mathrm{P1}\,\beta 1\,\sigma\mathrm{C1}))-\frac{\mathrm{I2s}\,\mathrm{K2}\,(-1+\mathrm{x2})\,\beta 2\,\lambda 2\,\sigma\mathrm{C2}}{\mu 2}+((-1+\mathrm{D2ss}+\mathrm{I2s})\,\mathrm{K2}\,\beta 2\,\lambda 2\,(-\mu 2+\mathrm{P2}\,(-1+\mathrm{x2})\,\beta 2\,\sigma\mathrm{C2}))\,/\,(\mu 2\,(\mu 2+\mathrm{P2}\,\beta 2\,\sigma\mathrm{C2}))\right)\right)\Big/\,(\mathrm{K1}\,\beta 1+\mathrm{K2}\,\beta 2+\gamma)$$

In the absence of the generalist parasite, the equilibria are much less complicated than before.

In[434]:=
```
(* Solving for the equilibria of the I1,s-D1,s,s-P1 system *)
(* D1ss in terms of P1 and I1,s *)
D1ssEq = Solve[
   (dI1sdt /. {C1sg → 0, Pg → 0, I1g → 0} /. {βS1 → β1, βI1 → β1, βD1 → β1}) == 0, D1ss];
(* I1,s in terms of P1 *)
I1sEq = Solve[(dD1ssdt /. D1ssEq[[1]] /. {βS1 → β1, βI1 → β1, βD1 → β1}) == 0, I1s];
(* D1,s,s in terms of P1 *)
D1ssEq = Simplify[D1ssEq /. I1sEq[[1]]];
(* P1 equilibrium *)
P1Eq = Solve[
   (dP1dt /. {C1sg → 0, Pg → 0, I1g → 0} /. {βS1 → β1, βI1 → β1, βD1 → β1} /. I1sEq[[1]] /.
      D1ssEq[[1]]) == 0, P1] // Simplify
(* D1,s,s equilibrium *)
D1ssEq = Simplify[D1ssEq /. P1Eq[[2]]]
(* I1,s equilibrium *)
I1sEq = Simplify[I1sEq /. P1Eq[[2]]]
(* Solving for the equilibria of the I2,s-D2,s,s-P2 system *)
(* D2ss in terms of P2 and I2,s *)
D2ssEq = Solve[
   (dI2sdt /. {C2sg → 0, Pg → 0, I2g → 0} /. {βS2 → β2, βI2 → β2, βD2 → β2}) == 0, D2ss];
(* I2,s in terms of P2 *)
I2sEq = Solve[(dD2ssdt /. D2ssEq[[1]] /. {βS2 → β2, βI2 → β2, βD2 → β2}) == 0, I2s];
(* D2,s,s in terms of P2 *)
D2ssEq = Simplify[D2ssEq /. I2sEq[[1]] /. {βS2 → β2, βI2 → β2, βD2 → β2}];
(* P2 equilibrium *)
P2Eq = Solve[
   (dP2dt /. {C2sg → 0, Pg → 0, I2g → 0} /. {βS2 → β2, βI2 → β2, βD2 → β2} /. I2sEq[[1]] /.
      D2ssEq[[1]]) == 0, P2] // Simplify
(* D2,s,s equilibrium *)
D2ssEq = Simplify[D2ssEq /. P2Eq[[2]]]
(* I2,s equilibrium *)
I2sEq = Simplify[I2sEq /. P2Eq[[2]]]
```

Out[437]= $\left\{\{P1 \to 0\}, \left\{P1 \to \frac{K1\,\beta1\,(\lambda1 - \mu1) - \gamma\,\mu1}{\beta1\,(K1\,\beta1 + \gamma)}\right\}\right\}$

Out[438]= $\{\{D1ss \to ((K1\,\beta1\,(\lambda1 - \mu1) - \gamma\,\mu1)^2\,\sigma D1) / (K1\,\beta1\,\lambda1\,(-\gamma\,\mu1\,(-1 + \sigma D1) + K1\,\beta1\,(\mu1 + \lambda1\,\sigma D1 - \mu1\,\sigma D1)))\}\}$

Out[439]= $\{\{I1s \to -(((K1\,\beta1 + \gamma)\,\mu1\,(\gamma\,\mu1 + K1\,\beta1\,(-\lambda1 + \mu1))) / (K1\,\beta1\,\lambda1\,(-\gamma\,\mu1\,(-1 + \sigma D1) + K1\,\beta1\,(\mu1 + \lambda1\,\sigma D1 - \mu1\,\sigma D1))))\}\}$

Out[443]= $\left\{\{P2 \to 0\}, \left\{P2 \to \frac{K2\,\beta2\,(\lambda2 - \mu2) - \gamma\,\mu2}{\beta2\,(K2\,\beta2 + \gamma)}\right\}\right\}$

Out[444]= $\{\{D2ss \to ((K2\,\beta2\,(\lambda2 - \mu2) - \gamma\,\mu2)^2\,\sigma D2) / (K2\,\beta2\,\lambda2\,(-\gamma\,\mu2\,(-1 + \sigma D2) + K2\,\beta2\,(\mu2 + \lambda2\,\sigma D2 - \mu2\,\sigma D2)))\}\}$

Out[445]= $\{\{I2s \to -(((K2\,\beta2 + \gamma)\,\mu2\,(\gamma\,\mu2 + K2\,\beta2\,(-\lambda2 + \mu2))) / (K2\,\beta2\,\lambda2\,(-\gamma\,\mu2\,(-1 + \sigma D2) + K2\,\beta2\,(\mu2 + \lambda2\,\sigma D2 - \mu2\,\sigma D2))))\}\}$

We can plug these equilibria into the expression and are again stuck unless we are willing to make some simplifying assumptions.

In[447]:=
```
Simplify[Rm2 /. P1Eq[[2]] /. D1ssEq[[1]] /. I1sEq[[1]] /. P2Eq[[2]] /. D2ssEq[[1]] /.
   I2sEq[[1]]]
```

Out[447]=
$$\frac{1}{K1\,\beta 1 + K2\,\beta 2 + \gamma}$$
```
a (- (((K1 β1 + γ) (γ μ1 (1 + (-1 + x1) σC1) + K1 β1 (μ1 + λ1 σC1 - x1 λ1 σC1 - μ1 σC1 +
            x1 μ1 σC1))) / (γ μ1 (-1 + σC1) + K1 β1 (μ1 (-1 + σC1) - λ1 σC1))) -
    ((K2 β2 + γ) (γ μ2 (1 + (-1 + x2) σC2) + K2 β2 (μ2 + λ2 σC2 - x2 λ2 σC2 - μ2 σC2 + x2 μ2 σC2))) /
     (γ μ2 (-1 + σC2) + K2 β2 (μ2 (-1 + σC2) - λ2 σC2)) +
    ((-1 + x1) (K1 β1 + γ) (γ μ1 + K1 β1 (-λ1 + μ1)) σC1) /
     (-γ μ1 (-1 + σD1) + K1 β1 (μ1 + λ1 σD1 - μ1 σD1)) +
    ((-1 + x2) (K2 β2 + γ) (γ μ2 + K2 β2 (-λ2 + μ2)) σC2) /
     (-γ μ2 (-1 + σD2) + K2 β2 (μ2 + λ2 σD2 - μ2 σD2)))
```

In particular, we assume that $\sigma_{C_1} = \sigma_{D_1} = \sigma_{C_2} = \sigma_{D_2} = 1$ and that host resources are partitioned equally between coinfecting strains ($x_1 = x_2 = 0.5$), then the R_m expression simplifies considerably, to $R_m = a\left(1 + \frac{2\gamma}{\beta_1 K_1 + \beta_2 K_2 + \gamma}\right)$.

This is the same expression as we encountered in Case 6 above. From this expression, it is immediately clear how changing body sizes or temperature will affect R_m, because R_m depends only on the abundances of each host. Since increasing body size reduces abundance, $\frac{\partial R_m}{\partial W} > 0$. Since increasing temperature decreases abundance, $\frac{\partial R_m}{\partial W} > 0$. This will be true for both endoparasites and ectoparasites.

```
Simplify[Rm2 /. P1Eq[[2]] /. D1ssEq[[1]] /. I1sEq[[1]] /. P2Eq[[2]] /. D2ssEq[[1]] /.
    I2sEq[[1]] /. {σD2 → 1, σD1 → 1, σC1 → 1, σC2 → 1, x1 → 1 / 2, x2 → 1 / 2}]
```

Out[449]=
$$\frac{a\,(K1\,\beta 1 + K2\,\beta 2 + 2\,\gamma)}{K1\,\beta 1 + K2\,\beta 2 + \gamma}$$
