# Supplementary material for: Host allometry influences the evolution of parasite host-generalism: theory and meta-analysis
Source: Philos Trans R Soc Lond B Biol Sci. 2017 Mar 13;372(1719):20160089. doi: 10.1098/rstb.2016.0089 (PMC5352816; doi:10.1098/rstb.2016.0089)

Appendix B: Deriving the invasion condition and analysis of models in Table 3

Appendix B: Trophically transmitted parasites

Case 1: One specialist parasite; avoidance of infected intermediate hosts

Let D_1 and D_2 be two definitive hosts and N be prey of both and the intermediate host. One touchy bit is how to deal with the effect of ingestion on both the dynamics of predator (the definitive host) and prey (the intermediate host). One possibility is that infection is embedded within a classic predator-prey model, where both predator and prey growth are impacted by one another. Such a model is quite different from the direct life cycle model studied in the main text, and is also very difficult to analyze. A second possibility is that prey density is constant; in this model you cannot assume that predator growth is entirely determined by prey ingestion (as in a classic predator-prey model) because the predator population will either grow or decay exponentially.

Here we assume that the intermediate host (the prey) has a constant population size. We let N_T be the total population size, and $N_{I,r}$ and $N_{I,m}$ be the abundance of intermedate host infected with the resident (specialist) and mutant (generalist) parasites, respectively. We don't need to track the number of susceptible intermediate hosts. The two definitive hosts both grow logistically in the absence of infection, with no direct effect of prey ingestion on their growth rate. One way to justify this assumption is to assume that the predators are eating lots of different prey items, so that their dynamics are largely independent of this particular prey item. However, infection is assumed to have an effect on the growth of the definitive host. We let $D_{1,S}$ and $D_{2,S}$ to be the number of primary and secondary definitive hosts that are susceptible to infection; $D_{1,I,r}$ is the number of primary definitive hosts infected by the specialist (resident) parasite; we assume that the secondary definitive host is not infected by its own specialist parasite. $D_{1,I,m}$ and $D_{2,I,m}$ are the numbers of primary and secondary definitive hosts infected by the generalist (mutant) parasite. Definitive hosts shed parasite back into the environment, with P_r and P_m the abundance of specialist and generalist in the environment. These parasites are consumed by the intermediate host, which can then transmit the parasite to the definitive host upon ingestion.

Note that there is no need to consider active vs. passive host seeking here, as there is only a single intermediate host that is assumed to contact parasites in the environment.

The full system is given below.

```
dNirdt = β (NT - Nir - Nim) Pr - a1 (D1s + D1ir + D1im) Nir - a2 (D2s + D2im) Nir;
dNimdt = β (NT - Nir - Nim) Pm - a1 (D1s + D1ir + D1im) Nim - a2 (D2s + D2im) Nim;
dD1sdt = r1 (D1s + D1ir + D1im) (1 - (D1s + D1ir + D1im)/K1) - a1 D1s (Nir + Nim);
dD2sdt = r2 (D2s + D2im) (1 - (D2s + D2im)/K2) - a2 D2s Nim;
dD1irdt = a1 D1s Nir - μ1 D1ir;
dD1imdt = a1 D1s Nim - μ1 D1im;
dD2imdt = a2 D2s Nim - μ2 D2im;
dPrdt = λ1 D1ir - β (NT - Nir - Nim) Pr - γ Pr;
dPmdt = c λ1 D1im + c λ2 D2im - β (NT - Nir - Nim) Pm - γ Pm;
```

The Jacobian matrix for this system is quite large, but has the same block triangular structure that we have observed previously.

```
(* The Jacobian matrix of partial derivatives *)
J = {{D[dD1sdt, D1s], D[dD1sdt, D2s], D[dD1sdt, Nir], D[dD1sdt, D1ir], D[dD1sdt, Pr],
     D[dD1sdt, Nim], D[dD1sdt, D1im], D[dD1sdt, D2im], D[dD1sdt, Pm]},
    {D[dD2sdt, D1s], D[dD2sdt, D2s], D[dD2sdt, Nir], D[dD2sdt, D1ir], D[dD2sdt, Pr],
     D[dD2sdt, Nim], D[dD2sdt, D1im], D[dD2sdt, D2im], D[dD2sdt, Pm]},
    {D[dNirdt, D1s], D[dNirdt, D2s], D[dNirdt, Nir], D[dNirdt, D1ir], D[dNirdt, Pr],
     D[dNirdt, Nim], D[dNirdt, D1im], D[dNirdt, D2im], D[dNirdt, Pm]},
    {D[dD1irdt, D1s], D[dD1irdt, D2s], D[dD1irdt, Nir], D[dD1irdt, D1ir], D[dD1irdt,
      Pr], D[dD1irdt, Nim], D[dD1irdt, D1im], D[dD1irdt, D2im], D[dD1irdt, Pm]},
    {D[dPrdt, D1s], D[dPrdt, D2s], D[dPrdt, Nir], D[dPrdt, D1ir], D[dPrdt, Pr],
     D[dPrdt, Nim], D[dPrdt, D1im], D[dPrdt, D2im], D[dPrdt, Pm]},
    {D[dNimdt, D1s], D[dNimdt, D2s], D[dNimdt, Nir], D[dNimdt, D1ir], D[dNimdt, Pr],
     D[dNimdt, Nim], D[dNimdt, D1im], D[dNimdt, D2im], D[dNimdt, Pm]},
    {D[dD1imdt, D1s], D[dD1imdt, D2s], D[dD1imdt, Nir], D[dD1imdt, D1ir], D[dD1imdt,
      Pr], D[dD1imdt, Nim], D[dD1imdt, D1im], D[dD1imdt, D2im], D[dD1imdt, Pm]},
    {D[dD2imdt, D1s], D[dD2imdt, D2s], D[dD2imdt, Nir], D[dD2imdt, D1ir], D[dD2imdt,
      Pr], D[dD2imdt, Nim], D[dD2imdt, D1im], D[dD2imdt, D2im], D[dD2imdt, Pm]},
    {D[dPmdt, D1s], D[dPmdt, D2s], D[dPmdt, Nir], D[dPmdt, D1ir], D[dPmdt, Pr],
     D[dPmdt, Nim], D[dPmdt, D1im], D[dPmdt, D2im], D[dPmdt, Pm]}
  };
(* The Jacobian, evaluated at the equilibrium where the generalist is absent *)
MatrixForm[J /. {Nim → 0, D1im → 0, D2im → 0, Pm → 0}]
```

$$\begin{pmatrix}
-a1\,Nir + \left(1 - \frac{D1ir+D1s}{K1}\right) r1 - \frac{(D1ir+D1s)\,r1}{K1} & 0 & -a1\,D1s & (1 \\
0 & \left(1 - \frac{D2s}{K2}\right) r2 - \frac{D2s\,r2}{K2} & 0 & \\
-a1\,Nir & -a2\,Nir & -a1\,(D1ir + D1s) - a2\,D2s - Pr\,\beta & \\
a1\,Nir & 0 & a1\,D1s & \\
0 & 0 & Pr\,\beta & \\
0 & 0 & 0 & \\
0 & 0 & 0 & \\
0 & 0 & 0 & \\
0 & 0 & 0 &
\end{pmatrix}$$

Because **J** is upper block triangular, the eigenvalues are given by the eigenvalues of the submatrices that fall on the diagonal of **J**. Whether invasion can happen or not depends entirely on the eigenvalues of the lower triangular matrix, given below.

```
MatrixForm[J[[6 ;; 9, 6 ;; 9]] /. {Nim → 0, D1im → 0, D2im → 0, Pm → 0}]
```

$$\begin{pmatrix} -a1\,(D1ir + D1s) - a2\,D2s & 0 & 0 & (-Nir + NT)\,\beta \\ a1\,D1s & -\mu 1 & 0 & 0 \\ a2\,D2s & 0 & -\mu 2 & 0 \\ 0 & c\,\lambda 1 & c\,\lambda 2 & -(-Nir + NT)\,\beta - \gamma \end{pmatrix}$$

We can apply the next generation matrix theorem to determine the stability by rewriting $J = F - V$ and looking at the spectral radius of $F.V^{-1}$.

```
(* Define the F and V matrices *)
F = {{0, 0, 0, β (NT - Nir)}, {a1 D1s, 0, 0, 0}, {a2 D2s, 0, 0, 0}, {0, c λ1, c λ2, 0}};
V = {{a1 (D1ir + D1s) + a2 D2s, 0, 0, 0},
    {0, μ1, 0, 0}, {0, 0, μ2, 0}, {0, 0, 0, β (NT - Nir) + γ}};
(* Confirming that J=F-V *)
(J[[6 ;; 9, 6 ;; 9]] /. {Nim → 0, D1im → 0, D2im → 0, Pm → 0}) == F - V // Simplify
(* Calculating the spectral radius *)
Eigenvalues[Dot[F, Inverse[V]]]
```

True

$$\left\{0,\ \left(c^{1/3}\,(Nir - NT)^{1/3}\,\beta^{1/3}\,(a2\,D2s\,\lambda 2\,\mu 1 + a1\,D1s\,\lambda 1\,\mu 2)^{1/3}\right) \Big/ \left((a1\,D1ir + a1\,D1s + a2\,D2s)^{1/3}\,(Nir\,\beta - NT\,\beta - \gamma)^{1/3}\,\mu 1^{1/3}\,\mu 2^{1/3}\right),\right.$$
$$-\left(\left((-1)^{1/3}\,c^{1/3}\,(Nir - NT)^{1/3}\,\beta^{1/3}\,(a2\,D2s\,\lambda 2\,\mu 1 + a1\,D1s\,\lambda 1\,\mu 2)^{1/3}\right) \Big/ \left((a1\,D1ir + a1\,D1s + a2\,D2s)^{1/3}\,(Nir\,\beta - NT\,\beta - \gamma)^{1/3}\,\mu 1^{1/3}\,\mu 2^{1/3}\right)\right),$$
$$\left.\left((-1)^{2/3}\,c^{1/3}\,(Nir - NT)^{1/3}\,\beta^{1/3}\,(a2\,D2s\,\lambda 2\,\mu 1 + a1\,D1s\,\lambda 1\,\mu 2)^{1/3}\right) \Big/ \left((a1\,D1ir + a1\,D1s + a2\,D2s)^{1/3}\,(Nir\,\beta - NT\,\beta - \gamma)^{1/3}\,\mu 1^{1/3}\,\mu 2^{1/3}\right)\right\}$$

Note that $-(-1)^{1/3} = -0.5 - 0.866025\,i$ and $(-1)^{2/3} = -0.5 + 0.866025\,i$, so whether the parasite-free equilibrium is stable or not depends entirely on the second eigenvalue, which can be rewritten as $R_m = \frac{\beta N_s N_T}{\beta N_s N_T + \gamma}\left(\frac{a_1 D_{1,S}}{a_1 D_{1,S} + a_1 D_{1,I,r} + a_2 D_{2,S}} \frac{c\lambda_1}{\mu_1} + \frac{a_2 D_{2,S}}{a_1 D_{1,S} + a_1 D_{1,I,r} + a_2 D_{2,S}} \frac{c\lambda_2}{\mu_2}\right)$, which has a nice intuitive meaning: $\frac{\beta(N_T - N_{Ir})}{\beta(N_T - N_{Ir}) + \gamma}$ is the probability that a parasite in the environment is ingested by a susceptible intermediate host; $\frac{a_1 D_{1,S}}{a_1 D_{1,S} + a_1 D_{1,I,r} + a_2 D_{2,S}}$ is the probability that an infected intermediate host is ingested by a susceptible primary definitive host; $\frac{a_2 D_{2,S}}{a_1 D_{1,S} + a_1 D_{1,I,r} + a_2 D_{2,S}}$ is the probability that an infected intermediate host is ingested by a suceptible secondary definitive host; $\frac{c\lambda_1}{\mu_1}$ and $\frac{c\lambda_2}{\mu_2}$ are the expected number of parasites shed from infected primary and secondary definitive hosts, respectively.

```
(* Confirming the biologically meaningful form of Rm *)
(Eigenvalues[Dot[F, Inverse[V]]][[2]])^3 ==
  β (NT - Nir) / (β (NT - Nir) + γ) (a1 D1s / (a1 (D1s + D1ir) + a2 D2s) c λ1 / μ1 + a2 D2s / (a1 (D1s + D1ir) + a2 D2s) c λ2 / μ2) // Simplify
```

True

To determine how changing parameters affects R_0 for this model, we need to know the equilibrium values of N_s, $D_{1,S}$, $D_{1,I,r}$, and $D_{2,S}$ when the generalist parasite is not present. We know that $D_{2,S} = K_2$, the carrying capacity for the secondary host, but the other equilibria are too complex to allow for simple analysis. Instead, we will use numerical exploration to see whether changing mass/temperature have any effect on invasion fitness. As before, we use simple allometric scaling relationships to relate key

processes to host body size and temperature. Additionally, we assume that the growth rate of the definitive host (r) depends on body size as well.

$r = r_0\, e^{E/k\,T}\, W^{-0.25}$

$K = K_0\, e^{E/k\,T}\, W^{-0.75}$

$\mu = \mu_0\, e^{-E/k\,T}\, W^{-0.25}$

$\lambda = \lambda_0\, e^{-E/k\,T}\, W^{0.75}$ (for endoparasites)

$\lambda = \lambda_0\, e^{-E/k\,T}\, W^{5/12}$ (for ectoparasites).

Values for \.08\.08E, k, r_0, K_0, and μ_0 that are appropriate for fish come from Savage et al. 2004. The estimate of λ_0 is modified from Poulin & George-Nascimento 2007.

The function below uses numerical simulation to determine the equilibrium values of N_s, $D_{1,S}$, $D_{1,I,r}$, and $D_{2,S}$ for the specified parameters. These values are then plugged into the R_m expression to calculate the invasion fitness.

```
NumSolInvFit = Function[{W, T, c, f, NTot, B, g, a},
  allom = {K1 → K0 Exp[E/(k T)] W^(-3/4), K2 → K0 Exp[E/(k T)] (f W)^(-3/4),
    μ1 → μ0 Exp[-E/(k T)] W^(-1/4), μ2 → μ0 Exp[-E/(k T)] (f W)^(-1/4), λ1 → λ0 Exp[-E/(k T)] W^(3/4),
    λ2 → λ0 Exp[-E/(k T)] (f W)^(3/4), r1 → r0 Exp[-E/(k T)] W^(-1/4)};
  pars = {E → 0.45`, k → 8.617`/10^5, K0 → 2.984`/10^9, μ0 → 1.785` × 10^8,
    λ0 → 2 × 10^8, r0 → 2.21 × 10^10, β → B, γ → g, a1 → a, a2 → a, NT → NTot};
  DOPRIamat = {{1 / 5}, {3 / 40, 9 / 40}, {44 / 45, -56 / 15, 32 / 9}, {19 372 / 6561,
     -25 360 / 2187, 64 448 / 6561, -212 / 729}, {9017 / 3168, -355 / 33, 46 732 / 5247, 49 /
      176, -5103 / 18 656}, {35 / 384, 0, 500 / 1113, 125 / 192, -2187 / 6784, 11 / 84}};
  DOPRIbvec = {35 / 384, 0, 500 / 1113, 125 / 192, -2187 / 6784, 11 / 84, 0};
  DOPRIcvec = {1 / 5, 3 / 10, 4 / 5, 8 / 9, 1, 1};
  DOPRIevec = {71 / 57 600, 0, -71 / 16 695, 71 / 1920, -17 253 / 339 200, 22 / 525, -1 / 40};
  DOPRICoefficients[5, p_] := N[{DOPRIamat, DOPRIbvec, DOPRIcvec, DOPRIevec}, p];
  Soln = NDSolve[({
        Nir'[t] == β (NT - Nir[t]) Pr[t] - a1 (D1s[t] + D1ir[t]) Nir[t] - a2 K2 Nir[t],
        D1s'[t] == r1 (D1s[t] + D1ir[t]) (1 - (D1s[t] + D1ir[t])/K1) - a1 D1s[t] Nir[t],
        D1ir'[t] == a1 D1s[t] Nir[t] - μ1 D1ir[t],
        Pr'[t] == λ1 D1ir[t] - β (NT - Nir[t]) Pr[t] - γ Pr[t],
        Nir[0] == 0,
        D1s[0] == 0.1,
        D1ir[0] == 0,
        Pr[0] == 1} /. allom /. pars),
    {Ns, Nir, D1s, D1ir, Pr}, {t, 0, 1000},
    Method → {"ExplicitRungeKutta", "DifferenceOrder" → 5,
      "Coefficients" → DOPRICoefficients, "StiffnessTest" → False}];
  (* Print[{Ns→(Ns[1000]/.Soln)[[1]],Nir→(Nir[1000]/.Soln)[[1]],
     D1s→(D1s[1000]/.Soln)[[1]],D1ir→(D1ir[1000]/.Soln)[[1]],
     Pr→(Pr[1000]/.Soln)[[1]]}];*)
  β (NT - Nir)/(β (NT - Nir) + γ) (a1 D1s/(a1 (D1s + D1ir) + a2 D2s) c λ1/μ1 + a2 D2s/(a1 (D1s + D1ir) + a2 D2s) c λ2/μ2) /.
      {Nir → (Nir[1000] /. Soln)[[1]], D1s → (D1s[1000] /. Soln)[[1]],
       D1ir → (D1ir[1000] /. Soln)[[1]]} /. D2s → K2 /. allom /. pars
 ];
```

For these parameters, increasing body size increases R_0:

```
Labeled[ListLinePlot[Table[{Table[W, {W, 10, 1000, 10}][[i]], InvFitAcrossW[[i]]},
   {i, 1, Length[InvFitAcrossW]}],
  PlotLabel → "Fig. S1. Effect of body size W on Rm"],
 {"Host mass W", "Generalist Rm"}, {Bottom, Left}, RotateLabel → True]
```

Fig. S1. Effect of body size *W* on R_m

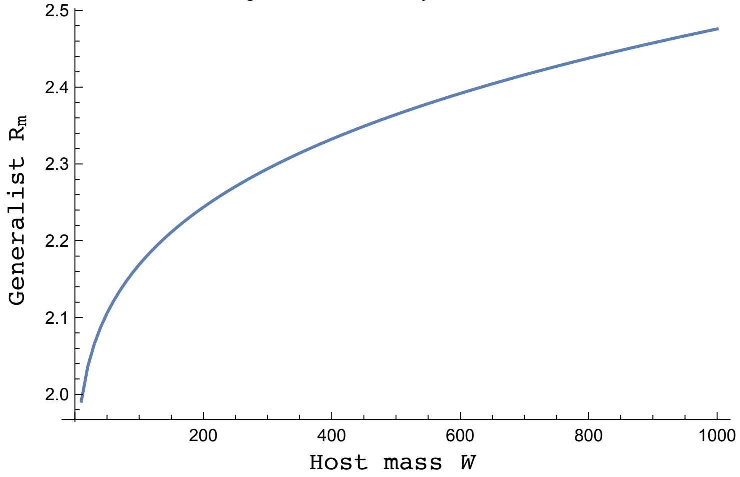

This is true if you increase the temperature (here increasing temperature from 270 to 310, Fig. S2). Notice, however, that R_m is lower for higher temperatures, indicating that increasing temperature negatively affects R_0.

```
InvFitAcrossWT =
  Table[Table[NumSolInvFit[W, T, 0.9, 0.9, 1, 0.1, 0.01, 0.1], {W, 25, 1000, 25}],
   {T, 270, 310, 10}];
```

```
Labeled[ListLinePlot[
  Table[Table[{Table[W, {W, 25, 1000, 25}][[i]], InvFitAcrossWT[[j, i]]}, {i, 1, 40}],
   {j, 1, 5}],
  PlotLegends → {"T=270", "T=280", "T=290", "T=300", "T=310"}, PlotLabel →
   "Fig. S2. Effect of body size W on Rm \nas the temperature T is varied"],
 {"Host mass W", "Generalist Rm"}, {Bottom, Left}, RotateLabel → True]
```

Fig. S2. Effect of body size *W* on R_m
as the temperature *T* is varied

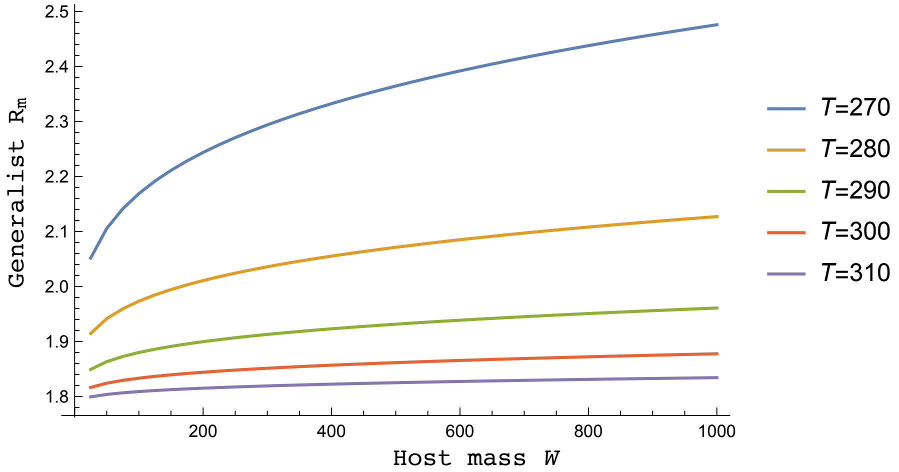

It also holds if you increase the cost of generalism (here decreasing *c* from 0.9 to 0.5; Fig. S3):

```
InvFitAcrossWc =
  Table[Table[NumSolInvFit[W, 270, c, 0.9, 1, 0.1, 0.01, 0.1], {W, 25, 1000, 25}],
   {c, 0.5, 0.9, 0.1}];
```

```
Labeled[ListLinePlot[
  Table[Table[{Table[W, {W, 25, 1000, 25}][[i]], InvFitAcrossWc[[j, i]]}, {i, 1, 40}],
   {j, 1, 5}], PlotLegends → {"c=0.5", "c=0.6", "c=0.7", "c=0.8", "c=0.9"},
  PlotLabel → "Fig. S3. Effect of body size W on Rm
     \nas the cost of generalism c is varied"],
 {"Host mass W", "Generalist Rm"}, {Bottom, Left}, RotateLabel → True]
```

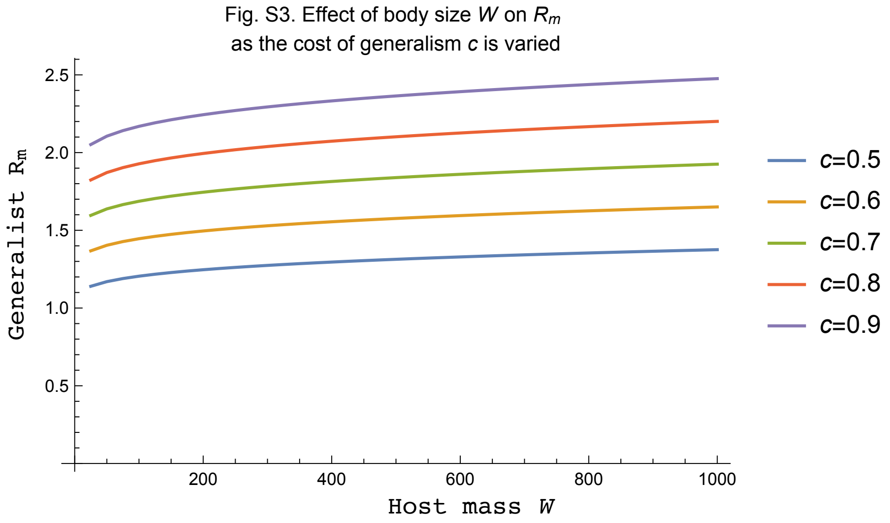

It also holds if you reduce the size of the secondary host (here decreasing *f* from 0.9 to 0.5; Fig. S4):

```
InvFitAcrossWf =
  Table[Table[NumSolInvFit[W, 270, 0.9, f, 1, 0.1, 0.01, 0.1], {W, 25, 1000, 25}],
   {f, 0.5, 0.9, 0.1}];
```

```
Labeled[ListLinePlot[
  Table[Table[{Table[W, {W, 25, 1000, 25}][[i]], InvFitAcrossWf[[j, i]]}, {i, 1, 40}],
   {j, 1, 5}], PlotLegends → {"f=0.5", "f=0.6", "f=0.7", "f=0.8", "f=0.9"},
  PlotLabel → "Fig. S4. Effect of body size W on Rm
     \nas the size of the second host f is varied"],
 {"Host mass W", "Generalist Rm"}, {Bottom, Left}, RotateLabel → True]
```

Fig. S4. Effect of body size W on R_m
as the size of the second host f is varied

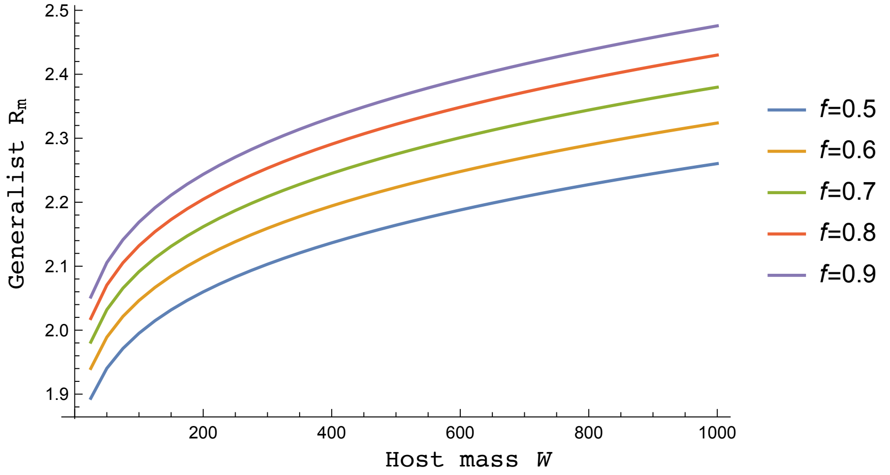

It also holds if you reduce the number of intermediate hosts (here N_T ranges from 0.25 to 2; Fig. S5):

```
InvFitAcrossWNT =
  Table[Table[NumSolInvFit[W, 270, 0.9, 0.9, NT, 0.1, 0.01, 0.1], {W, 25, 1000, 25}],
   {NT, 0.5, 2, 0.5}];
```

```
Labeled[ListLinePlot[Table[
    Table[{Table[W, {W, 25, 1000, 25}][[i]], InvFitAcrossWNT[[j, i]]}, {i, 1, 40}],
   {j, 1, 4}], PlotLegends → {"NT=0.5", "NT=1.0", "NT=1.5", "NT=2.0"},
  PlotLabel → "Fig. S5. Effect of body size W on Rm \nas
     the number of intermediate hosts NT is varied"],
 {"Host mass W", "Generalist Rm"}, {Bottom, Left}, RotateLabel → True]
```

Fig. S5. Effect of body size W on R_m
as the number of intermediate hosts N_T is varied

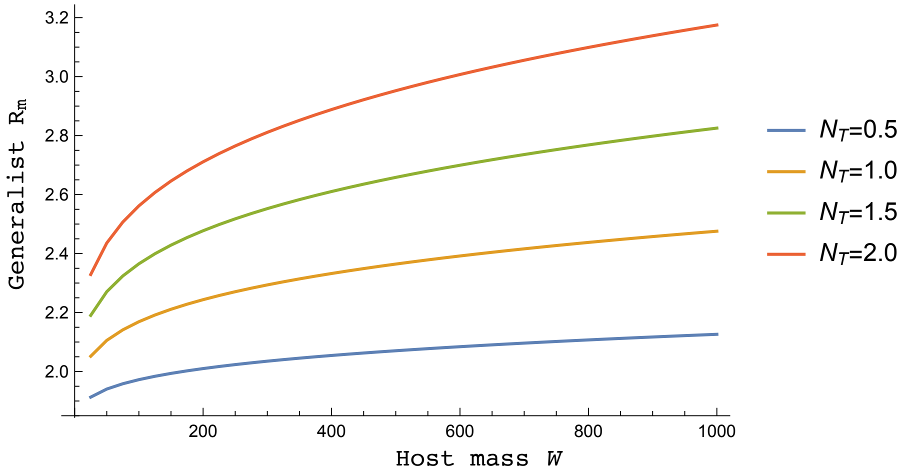

It also holds if you change the transmission rate to intermediate hosts (here β ranges from 0.05 to 0.55;

Fig. S6):

```
InvFitAcrossWB =
  Table[Table[NumSolInvFit[W, 270, 0.9, 0.9, 1, B, 0.01, 0.1], {W, 25, 1000, 25}],
   {B, 0.05, 0.55, 0.1}];
```

```
Labeled[ListLinePlot[
  Table[Table[{Table[W, {W, 25, 1000, 25}][[i]], InvFitAcrossWB[[j, i]]}, {i, 1, 40}],
   {j, 1, 6}], PlotLegends →
   {"β=0.05", "β=0.15", "β=0.25", "β=0.35", "β=0.45", "β=0.55"}, PlotLabel →
   "Fig. S6. Effect of body size W on Rm \nas the contact rate β is varied",
  PlotRange → All],
 {"Host mass W", "Generalist Rm"}, {Bottom, Left}, RotateLabel → True]
```

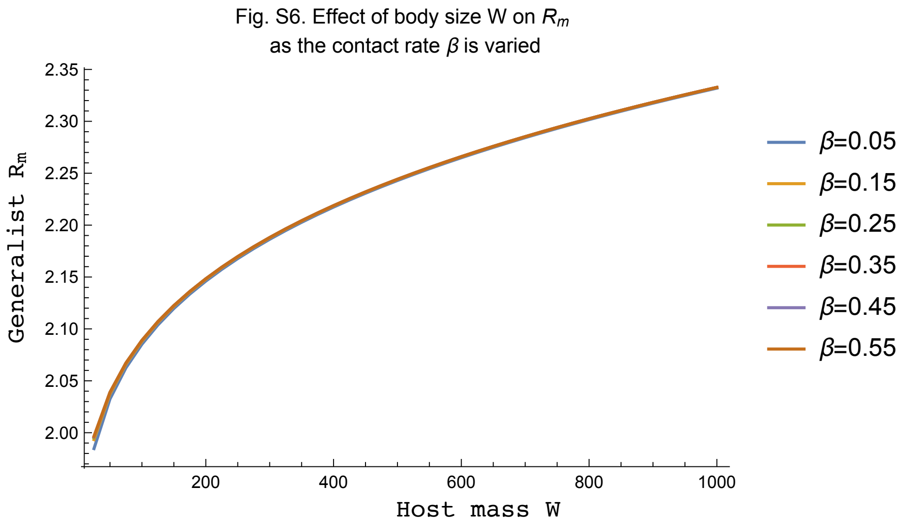

It also holds if you change the rate parasites are lost from the environment (here γ ranges from 0.01 to 0.1; Fig. S7):

```
InvFitAcrossWg =
  Table[Table[NumSolInvFit[W, 270, 0.9, 0.9, 1, 0.1, g, 0.1], {W, 25, 1000, 25}],
   {g, 0.02, 0.1, 0.02}];
```

```
Labeled[ListLinePlot[
  Table[Table[{Table[W, {W, 25, 1000, 25}][[i]], InvFitAcrossWg[[j, i]]}, {i, 1, 40}],
   {j, 1, 5}], PlotLegends →
   {"γ=0.02", "γ=0.04", "γ=0.06", "γ=0.08", "γ=0.1"}, PlotLabel →
   "Fig. S7. Effect of body size W on Rm \nas the parasite loss rate γ is varied",
  PlotRange → All],
 {"Host mass W", "Generalist Rm"}, {Bottom, Left}, RotateLabel → True]
```

Fig. S7. Effect of body size W on R_m
as the parasite loss rate γ is varied

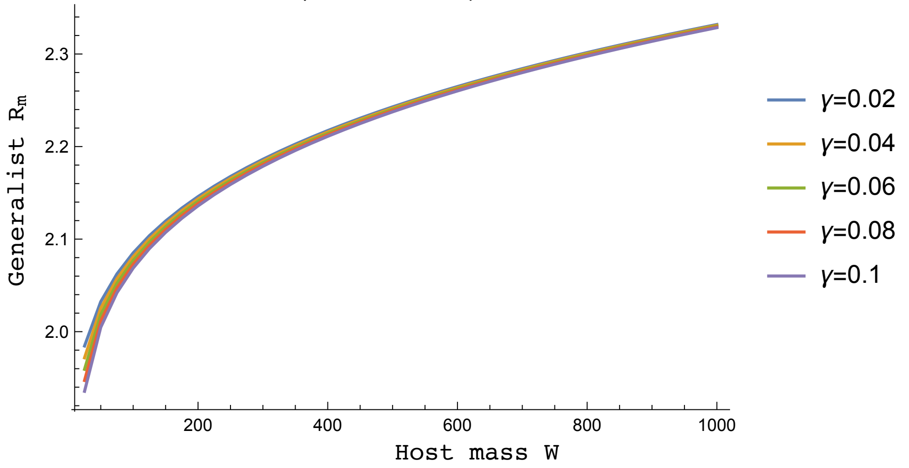

It also holds if you change the ingestion rate of the definitive hosts:

```
InvFitAcrossWa =
  Table[Table[NumSolInvFit[W, 270, 0.9, 0.9, 1, 0.1, 0.01, a], {W, 25, 1000, 25}],
   {a, 0.1, 0.5, 0.1}];
```

```
Labeled[ListLinePlot[
  Table[Table[{Table[W, {W, 25, 1000, 25}][[i]], InvFitAcrossWa[[j, i]]}, {i, 1, 40}],
   {j, 1, 5}],
  PlotLegends → {"a=0.1", "a=0.2", "a=0.3", "a=0.4", "a=0.5"}, PlotLabel →
   "Fig. S8. Effect of body size W on Rm \nas the ingestion rate a is varied",
  PlotRange → All],
 {"Host mass W", "Generalist Rm"}, {Bottom, Left}, RotateLabel → True]
```

Fig. S8. Effect of body size W on R_m
as the ingestion rate a is varied

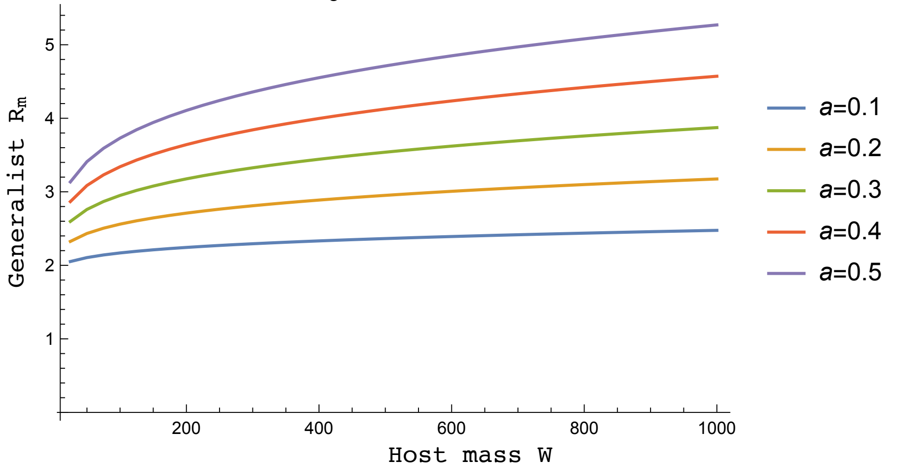

Case 2: Two specialist parasites; avoidance of infected intermediate hosts

Now we assume that there are two specialist parasites exploiting the same intermediate host, but infecting different definitive hosts. We let N_{2Ir} track the number of intermediate hosts infected with the second specialist parasite and D_{2Ir} track the number of secondary definitive hosts infected by the second specialist parasite.

```
dN1irdt =
  β (NT - N1ir - N2ir - Nim) P1r - a1 (D1s + D1ir + D1im) N1ir - a2 (D2s + D2ir + D2im) N1ir;
dN2irdt = β (NT - N1ir - N2ir - Nim) P2r - a1 (D1s + D1ir + D1im) N2ir -
   a2 (D2s + D2ir + D2im) N2ir;
dNimdt = β (NT - N1ir - N2ir - Nim) Pm - a1 (D1s + D1ir + D1im) Nim - a2 (D2s + D2ir + D2im) Nim;
```

$$dD1sdt = r1\ (D1s + D1ir + D1im) \left(1 - \frac{D1s + D1ir + D1im}{K1}\right) - a1\ D1s\ (N1ir + Nim);$$

$$dD2sdt = r2\ (D2s + D2ir + D2im) \left(1 - \frac{D2s + D2ir + D2im}{K2}\right) - a2\ D2s\ (N2ir + Nim);$$

```
dD1irdt = a1 D1s N1ir - μ1 D1ir;
dD2irdt = a2 D2s N2ir - μ2 D2ir;
dD1imdt = a1 D1s Nim - μ1 D1im;
dD2imdt = a2 D2s Nim - μ2 D2im;

dP1rdt = λ1 D1ir - β (NT - N1ir - N2ir - Nim) P1r - γ P1r;
dP2rdt = λ2 D2ir - β (NT - N1ir - N2ir - Nim) P2r - γ P2r;
dPmdt = c λ1 D1im + c λ2 D2im - β (NT - N1ir - N2ir - Nim) Pm - γ Pm;
```

To determine whether the generalist can invade this system, we again look at the Jacobian.

```
J = {{D[dN1irdt, N1ir], D[dN1irdt, D1s], D[dN1irdt, D1ir], D[dN1irdt, P1r],
     D[dN1irdt, N2ir], D[dN1irdt, D2s], D[dN1irdt, D2ir], D[dN1irdt, P2r],
     D[dN1irdt, Nim], D[dN1irdt, D1im], D[dN1irdt, D2im], D[dN1irdt, Pm]},
    {D[dD1sdt, N1ir], D[dD1sdt, D1s], D[dD1sdt, D1ir], D[dD1sdt, P1r],
     D[dD1sdt, N2ir], D[dD1sdt, D2s], D[dD1sdt, D2ir], D[dD1sdt, P2r],
     D[dD1sdt, Nim], D[dD1sdt, D1im], D[dD1sdt, D2im], D[dD1sdt, Pm]},
    {D[dD1irdt, N1ir], D[dD1irdt, D1s], D[dD1irdt, D1ir], D[dD1irdt, P1r],
     D[dD1irdt, N2ir], D[dD1irdt, D2s], D[dD1irdt, D2ir], D[dD1irdt, P2r],
     D[dD1irdt, Nim], D[dD1irdt, D1im], D[dD1irdt, D2im], D[dD1irdt, Pm]},
    {D[dP1rdt, N1ir], D[dP1rdt, D1s], D[dP1rdt, D1ir], D[dP1rdt, P1r],
     D[dP1rdt, N2ir], D[dP1rdt, D2s], D[dP1rdt, D2ir], D[dP1rdt, P2r],
     D[dP1rdt, Nim], D[dP1rdt, D1im], D[dP1rdt, D2im], D[dP1rdt, Pm]},
    {D[dN2irdt, N1ir], D[dN2irdt, D1s], D[dN2irdt, D1ir], D[dN2irdt, P1r],
     D[dN2irdt, N2ir], D[dN2irdt, D2s], D[dN2irdt, D2ir], D[dN2irdt, P2r],
     D[dN2irdt, Nim], D[dN2irdt, D1im], D[dN2irdt, D2im], D[dN2irdt, Pm]},
    {D[dD2sdt, N1ir], D[dD2sdt, D1s], D[dD2sdt, D1ir], D[dD2sdt, P1r],
     D[dD2sdt, N2ir], D[dD2sdt, D2s], D[dD2sdt, D2ir], D[dD2sdt, P2r],
     D[dD2sdt, Nim], D[dD2sdt, D1im], D[dD2sdt, D2im], D[dD2sdt, Pm]},
    {D[dD2irdt, N1ir], D[dD2irdt, D1s], D[dD2irdt, D1ir], D[dD2irdt, P1r],
     D[dD2irdt, N2ir], D[dD2irdt, D2s], D[dD2irdt, D2ir], D[dD2irdt, P2r],
     D[dD2irdt, Nim], D[dD2irdt, D1im], D[dD2irdt, D2im], D[dD2irdt, Pm]},
    {D[dP2rdt, N1ir], D[dP2rdt, D1s], D[dP2rdt, D1ir], D[dP2rdt, P1r],
     D[dP2rdt, N2ir], D[dP2rdt, D2s], D[dP2rdt, D2ir], D[dP2rdt, P2r],
     D[dP2rdt, Nim], D[dP2rdt, D1im], D[dP2rdt, D2im], D[dP2rdt, Pm]},
    {D[dNimdt, N1ir], D[dNimdt, D1s], D[dNimdt, D1ir], D[dNimdt, P1r],
     D[dNimdt, N2ir], D[dNimdt, D2s], D[dNimdt, D2ir], D[dNimdt, P2r],
     D[dNimdt, Nim], D[dNimdt, D1im], D[dNimdt, D2im], D[dNimdt, Pm]},
    {D[dD1imdt, N1ir], D[dD1imdt, D1s], D[dD1imdt, D1ir], D[dD1imdt, P1r],
     D[dD1imdt, N2ir], D[dD1imdt, D2s], D[dD1imdt, D2ir], D[dD1imdt, P2r],
     D[dD1imdt, Nim], D[dD1imdt, D1im], D[dD1imdt, D2im], D[dD1imdt, Pm]},
    {D[dD2imdt, N1ir], D[dD2imdt, D1s], D[dD2imdt, D1ir], D[dD2imdt, P1r],
     D[dD2imdt, N2ir], D[dD2imdt, D2s], D[dD2imdt, D2ir], D[dD2imdt, P2r],
     D[dD2imdt, Nim], D[dD2imdt, D1im], D[dD2imdt, D2im], D[dD2imdt, Pm]},
    {D[dPmdt, N1ir], D[dPmdt, D1s], D[dPmdt, D1ir], D[dPmdt, P1r],
     D[dPmdt, N2ir], D[dPmdt, D2s], D[dPmdt, D2ir], D[dPmdt, P2r],
     D[dPmdt, Nim], D[dPmdt, D1im], D[dPmdt, D2im], D[dPmdt, Pm]}} /.
  {Nim → 0, D1im → 0, D2im → 0, Pm → 0};
```

As before, this matrix is upper block triangular. The upper left submatrix determines the stability of the system that doesn't include the generalist parasite. Whether the generalist can invade the system depends on the eigenvalues of the lower right submatrix of **J**:

```
MatrixForm[J[[9 ;; 12, 9 ;; 12]]]
```

$$\begin{pmatrix} -\mathrm{a1}\,(\mathrm{D1ir}+\mathrm{D1s})-\mathrm{a2}\,(\mathrm{D2ir}+\mathrm{D2s}) & 0 & 0 & (-\mathrm{N1ir}-\mathrm{N2ir}+\mathrm{NT})\,\beta \\ \mathrm{a1}\,\mathrm{D1s} & -\mu 1 & 0 & 0 \\ \mathrm{a2}\,\mathrm{D2s} & 0 & -\mu 2 & 0 \\ 0 & \mathrm{c}\,\lambda 1 & \mathrm{c}\,\lambda 2 & -(-\mathrm{N1ir}-\mathrm{N2ir}+\mathrm{NT})\,\beta-\gamma \end{pmatrix}$$

Using the next generation matrix theorem, the Jacobian will have a positive eigenvalue whenever the spectral radius, given by the second value below, is greater than 1.

```
(* Define F and V *)
F = {{0, 0, 0, (NT - N1ir - N2ir) β},
    {a1 D1s, 0, 0, 0}, {a2 D2s, 0, 0, 0}, {0, c λ1, c λ2, 0}};
V = {{a1 (D1ir + D1s) + a2 (D2ir + D2s), 0, 0, 0}, {0, μ1, 0, 0},
    {0, 0, μ2, 0}, {0, 0, 0, (NT - N1ir - N2ir) β + γ}};
(* Confirming that J=F-V *)
J[[9 ;; 12, 9 ;; 12]] == F - V // Simplify
(* Stability is determined by the spectral radius of F.V^-1*)
Eigenvalues[Dot[F, Inverse[V]]]
```

```
True
```

```
{0, (c^(1/3) (N1ir + N2ir - NT)^(1/3) β^(1/3) (a2 D2s λ2 μ1 + a1 D1s λ1 μ2)^(1/3)) /
   ((a1 D1ir + a1 D1s + a2 D2ir + a2 D2s)^(1/3) (N1ir β + N2ir β - NT β - γ)^(1/3) μ1^(1/3) μ2^(1/3)),
 - (((-1)^(1/3) c^(1/3) (N1ir + N2ir - NT)^(1/3) β^(1/3) (a2 D2s λ2 μ1 + a1 D1s λ1 μ2)^(1/3)) /
     ((a1 D1ir + a1 D1s + a2 D2ir + a2 D2s)^(1/3) (N1ir β + N2ir β - NT β - γ)^(1/3) μ1^(1/3) μ2^(1/3))),
 ((-1)^(2/3) c^(1/3) (N1ir + N2ir - NT)^(1/3) β^(1/3) (a2 D2s λ2 μ1 + a1 D1s λ1 μ2)^(1/3)) /
   ((a1 D1ir + a1 D1s + a2 D2ir + a2 D2s)^(1/3) (N1ir β + N2ir β - NT β - γ)^(1/3) μ1^(1/3) μ2^(1/3))}
```

The spectral radius is equivalent to $R_m = \frac{\beta N_s N_T}{\beta N_s N_T+\gamma}\left(\frac{a_1 D_{1,S}}{a_1 D_{1,S}+a_1 D_{1,I,r}+a_2 D_{2,S}} \frac{c\lambda_1}{\mu_1} + \frac{a_2 D_{2,S}}{a_1 D_{1,S}+a_1 D_{1,I,r}+a_2 D_{2,S}} \frac{c\lambda_2}{\mu_2}\right)$.

```
(Eigenvalues[Dot[F, Inverse[V]]][[2]])^3 ==
  ((NT - N1ir - N2ir) β)/((NT - N1ir - N2ir) β + γ) (((a1 D1s) / (a1 (D1ir + D1s) + a2 (D2ir + D2s))) (c λ1)/μ1 +
    ((a2 D2s) / (a1 (D1ir + D1s) + a2 (D2ir + D2s))) (c λ2)/μ2) // Simplify
```

```
True
```

However, as before, it is impossible to make headway analytically, so we must resort to numerical solutions. The code below simulates the ODE system and computes the value of R_m.

```
NumSolInvFit = Function[{W, T, c, f, NTot, B, g, a},
   allom = {K1 → K0 Exp[E/(k T)] W^(-3/4), K2 → K0 Exp[E/(k T)] (f W)^(-3/4),
     μ1 → μ0 Exp[-E/(k T)] W^(-1/4), μ2 → μ0 Exp[-E/(k T)] (f W)^(-1/4), λ1 → λ0 Exp[-E/(k T)] W^(3/4),
     λ2 → λ0 Exp[-E/(k T)] (f W)^(3/4), r1 → r0 Exp[-E/(k T)] W^(-1/4), r2 → r0 Exp[-E/(k T)] (f W)^(-1/4)};
   pars = {E → 0.45`, k → 8.617`/10^5, K0 → 2.984`/10^9, μ0 → 1.785` × 10^8,
     λ0 → 2 × 10^8, r0 → 2.21 × 10^10, β → B, γ → g, a1 → a, a2 → a, NT → NTot};
   (*Print[(β NT)/(β NT+γ) ((a1 K1)/(a1 K1+a2 K2) λ1/μ1)/.allom/.pars];*)
   (*Print[(β NT)/(β NT+γ) ((a2 K2)/(a1 K1+a2 K2) λ2/μ2)/.allom/.pars];*)
   DOPRIamat = {{1 / 5}, {3 / 40, 9 / 40}, {44 / 45, -56 / 15, 32 / 9},
     {19372 / 6561, -25360 / 2187, 64448 / 6561, -212 / 729},
     {9017 / 3168, -355 / 33, 46732 / 5247, 49 / 176, -5103 / 18656},
     {35 / 384, 0, 500 / 1113, 125 / 192, -2187 / 6784, 11 / 84}};
   DOPRIbvec = {35 / 384, 0, 500 / 1113, 125 / 192, -2187 / 6784, 11 / 84, 0};
```

```
DOPRIcvec = {1 / 5, 3 / 10, 4 / 5, 8 / 9, 1, 1};
DOPRIevec = {71 / 57 600, 0, -71 / 16 695, 71 / 1920, -17 253 / 339 200, 22 / 525, -1 / 40};
DOPRICoefficients[5, p_] := N[{DOPRIamat, DOPRIbvec, DOPRIcvec, DOPRIevec}, p];
Soln = NDSolve[({
    N1ir'[t] == β (NT - N1ir[t] - N2ir[t]) P1r[t] -
      a1 (D1s[t] + D1ir[t]) N1ir[t] - a2 (D2s[t] + D2ir[t]) N1ir[t],
    N2ir'[t] == β (NT - N1ir[t] - N2ir[t]) P2r[t] -
      a1 (D1s[t] + D1ir[t]) N2ir[t] - a2 (D2s[t] + D2ir[t]) N2ir[t],
    D1s'[t] == r1 (D1s[t] + D1ir[t]) (1 - (D1s[t] + D1ir[t])/K1) - a1 D1s[t] N1ir[t],
    D2s'[t] == r2 (D2s[t] + D2ir[t]) (1 - (D2s[t] + D2ir[t])/K2) - a2 D2s[t] N2ir[t],
    D1ir'[t] == a1 D1s[t] N1ir[t] - μ1 D1ir[t],
    D2ir'[t] == a2 D2s[t] N2ir[t] - μ1 D2ir[t],
    P1r'[t] == λ1 D1ir[t] - β (NT - N1ir[t] - N2ir[t]) P1r[t] - γ P1r[t],
    P2r'[t] == λ1 D1ir[t] - β (NT - N1ir[t] - N2ir[t]) P2r[t] - γ P2r[t],
    N1ir[0] == 0, N2ir[0] == 0,
    D1s[0] == 0.1, D2s[0] == 0.1,
    D1ir[0] == 0, D2ir[0] == 0,
    P1r[0] == 1, P2r[0] == 1} /. allom /. pars),
  {N1ir, N2ir, D1s, D1ir, D2s, D2ir, P1r, P2r}, {t, 0, 1000},
  Method -> {"ExplicitRungeKutta", "DifferenceOrder" -> 5,
    "Coefficients" -> DOPRICoefficients, "StiffnessTest" -> False}];
(* Print[{N1ir->(N1ir[1000]/.Soln)[[1]],N2ir->(N2ir[1000]/.Soln)[[1]],
  D1s->(D1s[1000]/.Soln)[[1]],D1ir->(D1ir[1000]/.Soln)[[1]],
  D2s->(D2s[1000]/.Soln)[[1]],D2ir->(D2ir[1000]/.Soln)[[1]]}] *);
((NT - N1ir - N2ir) β)/((NT - N1ir - N2ir) β + γ) (((a1 D1s) / (a1 (D1ir + D1s) + a2 (D2ir + D2s))) (c λ1)/μ1 +
      ((a2 D2s) / (a1 (D1ir + D1s) + a2 (D2ir + D2s))) (c λ2)/μ2) /.
    {N1ir -> (N1ir[1000] /. Soln)[[1]], N2ir -> (N2ir[1000] /. Soln)[[1]],
     D1s -> (D1s[1000] /. Soln)[[1]], D1ir -> (D1ir[1000] /. Soln)[[1]], D2s ->
      (D2s[1000] /. Soln)[[1]], D2ir -> (D2ir[1000] /. Soln)[[1]]} /. allom /. pars
];
```

One case is sufficient to demonstrate that the response of the generalist's R_m to changes in host body size is much more complex here. Consider the effect of changing the definitive host body sizes across a gradient of intermediate host abundance. You can see very clearly that the responses depend on the value of N_T: when N_T is small, increasing host mass increases R_m; when N_T is large, increasing host mass first increases, then decreases R_m (Fig. S9).

In reality, the abundance of the definitive host's prey is likely to be related to the size of the definitive host: in general, larger-bodied hosts are more likely to consume larger-bodied prey, whose carrying capacities would decrease commensurately. That is, as definitive host body size goes up, you would expect intermediate host carrying capacity to go down.

```
InvFitAcrossWNT =
  Table[Table[NumSolInvFit[W, 270, 0.9, 0.9, NT, 0.01, 0.1, 0.01], {W, 25, 1000, 25}],
   {NT, {0.1, 0.2, 0.5, 1, 1.5, 2}}];
```

```
Labeled[ListLinePlot[
  Table[Table[{Table[W, {W, 25, 1000, 25}][[i]], InvFitAcrossWNT[[j, i]]},
    {i, 1, 40}], {j, 1, 6}],
  PlotLegends → {"NT=0.1", "NT=0.2", "NT=0.5", "NT=1.0", "NT=1.5", "NT=2.0"},
  PlotLabel → "Fig. S9. Effect of body size W on Rm \nas the
     abundance of intermediate hosts NT is varied", PlotRange → All],
 {"Host mass W", "Generalist Rm"}, {Bottom, Left}, RotateLabel → True]
```

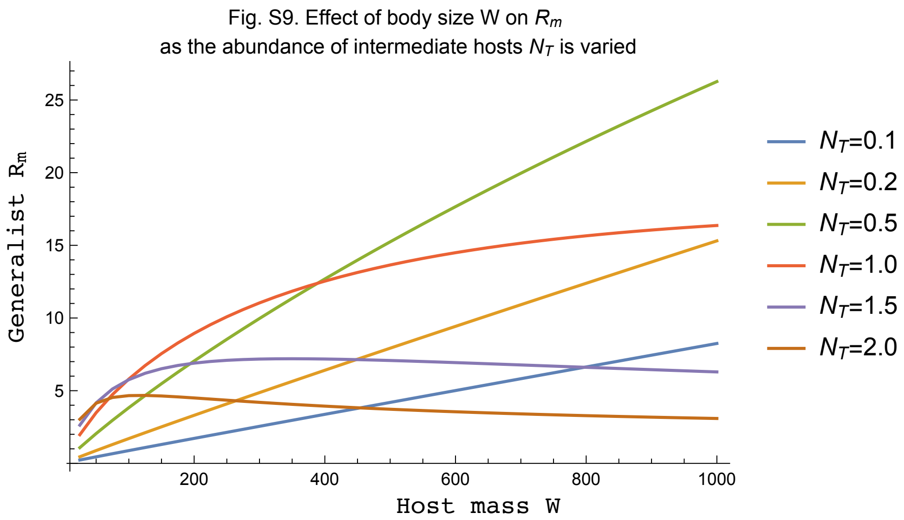

Increasing temperature also has a much more complex effect on R_m: when N_T is large, increasing temperature increases R_m (Fig. S10), but when N_T is small, increasing temperature decreases R_m (Fig. S11).

```
(* Variation in Rm as T varies when NT=2 *)
InvFitAcrossWT =
  Table[NumSolInvFit[200, T, 0.9, 0.9, 2, 0.01, 0.1, 0.01], {T, 270, 310, 2}];
```

```
Labeled[ListLinePlot[
  Table[{Table[T, {T, 270, 310, 2}][[i]], InvFitAcrossWT[[i]]}, {i, 1, 21}],
  PlotLabel → "Fig. S10. Effect of T on Rm when NT is large"],
 {"Temperature T", "Generalist Rm"}, {Bottom, Left}, RotateLabel → True]
```

Fig. S10. Effect of T on R_m when N_T is large

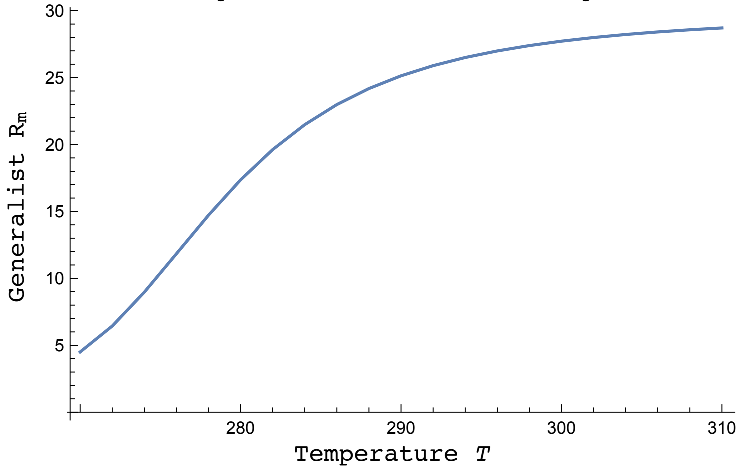


```
(* Variation in Rm as T varies when NT=0.1 *)
InvFitAcrossWT =
  Table[NumSolInvFit[200, T, 0.9, 0.9, 0.1, 0.01, 0.1, 0.01], {T, 270, 310, 2}];
```

```
Labeled[ListLinePlot[
  Table[{Table[T, {T, 270, 310, 2}][[i]], InvFitAcrossWT[[i]]}, {i, 1, 21}],
  PlotLabel → "Fig. S11. Effect of T on Rm when NT is small"],
 {"Temperature T", "Generalist Rm"}, {Bottom, Left}, RotateLabel → True]
```

Fig. S11. Effect of T on R_m when N_T is small

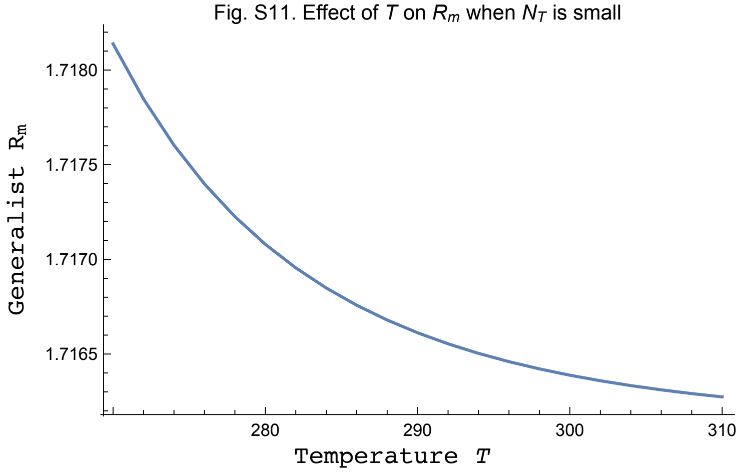

Case 3: Two specialist parasites; no avoidance of infected intermediate hosts

This case assumes that the parasite cannot determine whether an intermediate host is infected or not. Thus both susceptible and infected intermediate hosts remove parasites from the environment, but only consumption by a susceptible host can produce a new infection.

```
dN1irdt =
  β (NT - N1ir - N2ir - Nim) P1r - a1 (D1s + D1ir + D1im) N1ir - a2 (D2s + D2ir + D2im) N1ir;
dN2irdt = β (NT - N1ir - N2ir - Nim) P2r - a1 (D1s + D1ir + D1im) N2ir -
   a2 (D2s + D2ir + D2im) N2ir;
dNimdt = β (NT - N1ir - N2ir - Nim) Pm - a1 (D1s + D1ir + D1im) Nim - a2 (D2s + D2ir + D2im) Nim;
```

$$dD1sdt = r1\ (D1s + D1ir + D1im)\ \left(1 - \frac{D1s + D1ir + D1im}{K1}\right) - a1\ D1s\ (N1ir + Nim);$$

$$dD2sdt = r2\ (D2s + D2ir + D2im)\ \left(1 - \frac{D2s + D2ir + D2im}{K2}\right) - a2\ D2s\ (N2ir + Nim);$$

```
dD1irdt = a1 D1s N1ir - μ1 D1ir;
dD2irdt = a2 D2s N2ir - μ2 D2ir;
dD1imdt = a1 D1s Nim - μ1 D1im;
dD2imdt = a2 D2s Nim - μ2 D2im;

dP1rdt = λ1 D1ir - β NT P1r - γ P1r;
dP2rdt = λ2 D2ir - β NT P2r - γ P2r;
dPmdt = c λ1 D1im + c λ2 D2im - β NT Pm - γ Pm;
```

To determine whether the generalist can invade this system, we again look at the Jacobian.

```
J = {{D[dN1irdt, N1ir], D[dN1irdt, D1s], D[dN1irdt, D1ir], D[dN1irdt, P1r],
     D[dN1irdt, N2ir], D[dN1irdt, D2s], D[dN1irdt, D2ir], D[dN1irdt, P2r],
     D[dN1irdt, Nim], D[dN1irdt, D1im], D[dN1irdt, D2im], D[dN1irdt, Pm]},
    {D[dD1sdt, N1ir], D[dD1sdt, D1s], D[dD1sdt, D1ir], D[dD1sdt, P1r],
     D[dD1sdt, N2ir], D[dD1sdt, D2s], D[dD1sdt, D2ir], D[dD1sdt, P2r],
     D[dD1sdt, Nim], D[dD1sdt, D1im], D[dD1sdt, D2im], D[dD1sdt, Pm]},
    {D[dD1irdt, N1ir], D[dD1irdt, D1s], D[dD1irdt, D1ir], D[dD1irdt, P1r],
     D[dD1irdt, N2ir], D[dD1irdt, D2s], D[dD1irdt, D2ir], D[dD1irdt, P2r],
     D[dD1irdt, Nim], D[dD1irdt, D1im], D[dD1irdt, D2im], D[dD1irdt, Pm]},
    {D[dP1rdt, N1ir], D[dP1rdt, D1s], D[dP1rdt, D1ir], D[dP1rdt, P1r],
     D[dP1rdt, N2ir], D[dP1rdt, D2s], D[dP1rdt, D2ir], D[dP1rdt, P2r],
     D[dP1rdt, Nim], D[dP1rdt, D1im], D[dP1rdt, D2im], D[dP1rdt, Pm]},
    {D[dN2irdt, N1ir], D[dN2irdt, D1s], D[dN2irdt, D1ir], D[dN2irdt, P1r],
     D[dN2irdt, N2ir], D[dN2irdt, D2s], D[dN2irdt, D2ir], D[dN2irdt, P2r],
     D[dN2irdt, Nim], D[dN2irdt, D1im], D[dN2irdt, D2im], D[dN2irdt, Pm]},
    {D[dD2sdt, N1ir], D[dD2sdt, D1s], D[dD2sdt, D1ir], D[dD2sdt, P1r],
     D[dD2sdt, N2ir], D[dD2sdt, D2s], D[dD2sdt, D2ir], D[dD2sdt, P2r],
     D[dD2sdt, Nim], D[dD2sdt, D1im], D[dD2sdt, D2im], D[dD2sdt, Pm]},
    {D[dD2irdt, N1ir], D[dD2irdt, D1s], D[dD2irdt, D1ir], D[dD2irdt, P1r],
     D[dD2irdt, N2ir], D[dD2irdt, D2s], D[dD2irdt, D2ir], D[dD2irdt, P2r],
     D[dD2irdt, Nim], D[dD2irdt, D1im], D[dD2irdt, D2im], D[dD2irdt, Pm]},
    {D[dP2rdt, N1ir], D[dP2rdt, D1s], D[dP2rdt, D1ir], D[dP2rdt, P1r],
     D[dP2rdt, N2ir], D[dP2rdt, D2s], D[dP2rdt, D2ir], D[dP2rdt, P2r],
     D[dP2rdt, Nim], D[dP2rdt, D1im], D[dP2rdt, D2im], D[dP2rdt, Pm]},
    {D[dNimdt, N1ir], D[dNimdt, D1s], D[dNimdt, D1ir], D[dNimdt, P1r],
     D[dNimdt, N2ir], D[dNimdt, D2s], D[dNimdt, D2ir], D[dNimdt, P2r],
     D[dNimdt, Nim], D[dNimdt, D1im], D[dNimdt, D2im], D[dNimdt, Pm]},
    {D[dD1imdt, N1ir], D[dD1imdt, D1s], D[dD1imdt, D1ir], D[dD1imdt, P1r],
     D[dD1imdt, N2ir], D[dD1imdt, D2s], D[dD1imdt, D2ir], D[dD1imdt, P2r],
     D[dD1imdt, Nim], D[dD1imdt, D1im], D[dD1imdt, D2im], D[dD1imdt, Pm]},
    {D[dD2imdt, N1ir], D[dD2imdt, D1s], D[dD2imdt, D1ir], D[dD2imdt, P1r],
     D[dD2imdt, N2ir], D[dD2imdt, D2s], D[dD2imdt, D2ir], D[dD2imdt, P2r],
     D[dD2imdt, Nim], D[dD2imdt, D1im], D[dD2imdt, D2im], D[dD2imdt, Pm]},
    {D[dPmdt, N1ir], D[dPmdt, D1s], D[dPmdt, D1ir], D[dPmdt, P1r],
     D[dPmdt, N2ir], D[dPmdt, D2s], D[dPmdt, D2ir], D[dPmdt, P2r],
     D[dPmdt, Nim], D[dPmdt, D1im], D[dPmdt, D2im], D[dPmdt, Pm]}} /.
  {Nim → 0, D1im → 0, D2im → 0, Pm → 0};
```

As before, this matrix is upper block triangular. The upper left submatrix determines the stability of the system that doesn't include the generalist parasite. Whether the generalist can invade the system depends on the eigenvalues of the lower right submatrix of **J**:

```
MatrixForm[J[[9 ;; 12, 9 ;; 12]]]
```

$$\begin{pmatrix} -\text{a1}\,(\text{D1ir}+\text{D1s})-\text{a2}\,(\text{D2ir}+\text{D2s}) & 0 & 0 & (-\text{N1ir}-\text{N2ir}+\text{NT})\,\beta \\ \text{a1 D1s} & -\mu 1 & 0 & 0 \\ \text{a2 D2s} & 0 & -\mu 2 & 0 \\ 0 & \text{c}\,\lambda 1 & \text{c}\,\lambda 2 & -\text{NT}\,\beta-\gamma \end{pmatrix}$$

Using the next generation matrix theorem, the invasion of the generalist requires that one of the following eigenvalues be larger than 1 in magnitude.

```
(* Defining F and V *)
 F = {{0, 0, 0, (NT - N1ir - N2ir) β},
    {a1 D1s, 0, 0, 0}, {a2 D2s, 0, 0, 0}, {0, c λ1, c λ2, 0}};
 V = {{a1 (D1ir + D1s) + a2 (D2ir + D2s), 0, 0, 0}, {0, μ1, 0, 0},
    {0, 0, μ2, 0}, {0, 0, 0, β NT + γ}};
 (* Confirming that J = F-V *)
 J[[9 ;; 12, 9 ;; 12]] == F - V // Simplify
 (* Calculating the spectral radius of F.V^-1 *)]
(Eigenvalues[Dot[F, Inverse[V]]][[2]])^3 ==
  (c (NT - N1ir - N2ir) β (a2 D2s λ2 μ1 + a1 D1s λ1 μ2)) /
   ((a1 D1ir + a1 D1s + a2 D2ir + a2 D2s) (NT β + γ) μ1 μ2) // Simplify
```

The invasion condition can be rewritten as:

```
(c (NT - N1ir - N2ir) β (a2 D2s λ2 μ1 + a1 D1s λ1 μ2)) /
   ((a1 D1ir + a1 D1s + a2 D2ir + a2 D2s) (NT β + γ) μ1 μ2) ==
  β (NT - N1ir - N2ir) / (β NT + γ) (((a1 D1s) / (a1 (D1ir + D1s) + a2 (D2ir + D2s))) c λ1 / μ1 +
    ((a2 D2s) / (a1 (D1ir + D1s) + a2 (D2ir + D2s))) c λ2 / μ2) // Simplify
```

```
True
```

As before, it is impossible to make headway analytically, so we must resort to numerical solutions.

```
NumSolInvFit = Function[{W, T, c, f, NTot, B, g, a},
   allom = {K1 → K0 Exp[E / (k T)] W^(-3/4), K2 → K0 Exp[E / (k T)] (f W)^(-3/4),
     μ1 → μ0 Exp[- E / (k T)] W^(-1/4), μ2 → μ0 Exp[- E / (k T)] (f W)^(-1/4), λ1 → λ0 Exp[- E / (k T)] W^(3/4),
     λ2 → λ0 Exp[- E / (k T)] (f W)^(3/4), r1 → r0 Exp[- E / (k T)] W^(-1/4), r2 → r0 Exp[- E / (k T)] (f W)^(-1/4)};
   pars = {E → 0.45`, k → 8.617` / 10^5, K0 → 2.984` / 10^9, μ0 → 1.785` × 10^8,
     λ0 → 2 × 10^8, r0 → 2.21 × 10^10, β → B, γ → g, a1 → a, a2 → a, NT → NTot};
   (*Print[β NT / (β NT+γ) (a1 K1 / (a1 K1+a2 K2) λ1 / μ1)/.allom/.pars];*)
   (*Print[β NT / (β NT+γ) (a2 K2 / (a1 K1+a2 K2) λ2 / μ2)/.allom/.pars];*)
   DOPRIamat = {{1 / 5}, {3 / 40, 9 / 40}, {44 / 45, -56 / 15, 32 / 9},
     {19 372 / 6561, -25 360 / 2187, 64 448 / 6561, -212 / 729},
     {9017 / 3168, -355 / 33, 46 732 / 5247, 49 / 176, -5103 / 18 656},
     {35 / 384, 0, 500 / 1113, 125 / 192, -2187 / 6784, 11 / 84}};
   DOPRIbvec = {35 / 384, 0, 500 / 1113, 125 / 192, -2187 / 6784, 11 / 84, 0};
   DOPRIcvec = {1 / 5, 3 / 10, 4 / 5, 8 / 9, 1, 1};
   DOPRIevec = {71 / 57 600, 0, -71 / 16 695, 71 / 1920, -17 253 / 339 200, 22 / 525, -1 / 40};
   DOPRICoefficients[5, p_] := N[{DOPRIamat, DOPRIbvec, DOPRIcvec, DOPRIevec}, p];
   Soln = NDSolve[({

       N1ir'[t] == β (NT - N1ir[t] - N2ir[t]) P1r[t] -
         a1 (D1s[t] + D1ir[t]) N1ir[t] - a2 (D2s[t] + D2ir[t]) N1ir[t],
       N2ir'[t] == β (NT - N1ir[t] - N2ir[t]) P2r[t] -
         a1 (D1s[t] + D1ir[t]) N2ir[t] - a2 (D2s[t] + D2ir[t]) N2ir[t],
```

```
          D1s'[t] == r1 (D1s[t] + D1ir[t]) (1 - (D1s[t] + D1ir[t])/K1) - a1 D1s[t] N1ir[t],
          D2s'[t] == r2 (D2s[t] + D2ir[t]) (1 - (D2s[t] + D2ir[t])/K2) - a2 D2s[t] N2ir[t],
          D1ir'[t] == a1 D1s[t] N1ir[t] - μ1 D1ir[t],
          D2ir'[t] == a2 D2s[t] N2ir[t] - μ1 D2ir[t],
          P1r'[t] == λ1 D1ir[t] - β NT P1r[t] - γ P1r[t],
          P2r'[t] == λ1 D1ir[t] - β NT P2r[t] - γ P2r[t],
          N1ir[0] == 0, N2ir[0] == 0,
          D1s[0] == 0.1, D2s[0] == 0.1,
          D1ir[0] == 0, D2ir[0] == 0,
          P1r[0] == 1, P2r[0] == 1} /. allom /. pars),
      {N1ir, N2ir, D1s, D1ir, D2s, D2ir, P1r, P2r}, {t, 0, 1000},
      Method → {"ExplicitRungeKutta", "DifferenceOrder" → 5,
        "Coefficients" → DOPRICoefficients, "StiffnessTest" → False}];
  (* Print[{N1ir→(N1ir[1000]/.Soln)[[1]],N2ir→(N2ir[1000]/.Soln)[[1]],
      D1s→(D1s[1000]/.Soln)[[1]],D1ir→(D1ir[1000]/.Soln)[[1]],
      D2s→(D2s[1000]/.Soln)[[1]],D2ir→(D2ir[1000]/.Soln)[[1]]}] *);
  β (NT - N1ir - N2ir)/(β NT + γ) (((a1 D1s) / (a1 (D1ir + D1s) + a2 (D2ir + D2s))) (c λ1)/μ1 +
        ((a2 D2s) / (a1 (D1ir + D1s) + a2 (D2ir + D2s))) (c λ2)/μ2) /.
      {N1ir → (N1ir[1000] /. Soln)[[1]], N2ir → (N2ir[1000] /. Soln)[[1]],
       D1s → (D1s[1000] /. Soln)[[1]], D1ir → (D1ir[1000] /. Soln)[[1]], D2s →
        (D2s[1000] /. Soln)[[1]], D2ir → (D2ir[1000] /. Soln)[[1]]} /. allom /. pars
  ];
```

Again, one case is sufficient to demonstrate that the response of the generalist's R_0 to changes in host body size is much more complex here by looking at the effect of changing the definitive host body sizes across a gradient of intermediate host abundance. You can see very clearly that the responses depend on the value of N_T: when N_T is small, increasing host mass increases R_0; when N_T is large, increasing host mass first increases, then decreases R_0 (Fig. S12).

```
InvFitAcrossWNT =
  Table[Table[NumSolInvFit[W, 270, 0.9, 0.9, NT, 0.01, 0.1, 0.01], {W, 25, 1000, 25}],
   {NT, {0.1, 0.2, 0.5, 1, 1.5, 2}}];
```

```
Labeled[ListLinePlot[
  Table[Table[{Table[W, {W, 25, 1000, 25}][[i]], InvFitAcrossWNT[[j, i]]},
    {i, 1, 40}], {j, 1, 6}],
  PlotLegends → {"NT=0.1", "NT=0.2", "NT=0.5", "NT=1.0", "NT=1.5", "NT=2.0"},
  PlotLabel → "Fig. S12. Effect of body size W on Rm \nas the
     abundance of intermediate hosts NT is varied", PlotRange → All],
 {"Host mass W", "Generalist Rm"}, {Bottom, Left}, RotateLabel → True]
```

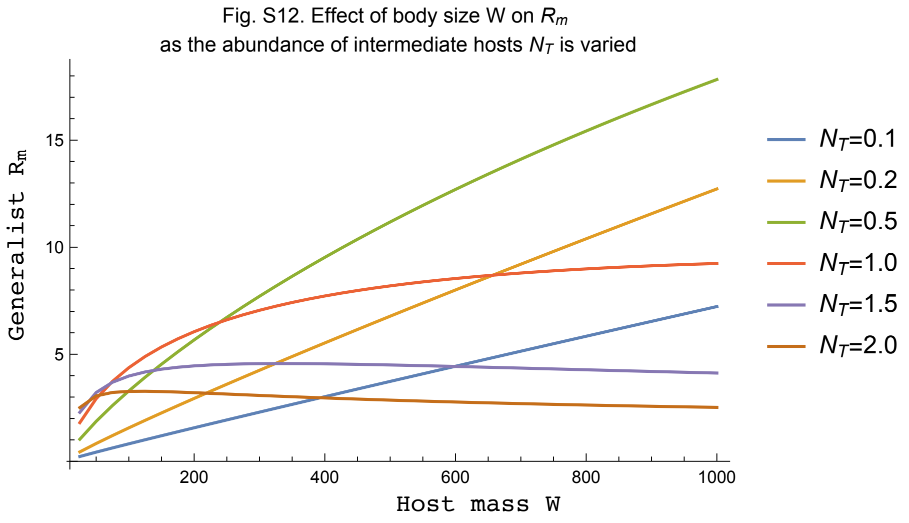

Increasing temperature also has a much more complex effect on R_0: when N_T is large, increasing temperature increases R_0, but when N_T is small, increasing temperature decreases R_0.

Increasing temperature also has a much more complex effect on R_m: when N_T is large, increasing temperature increases R_m (Fig. S13), but when N_T is small, increasing temperature decreases R_m (Fig. S14).

```
(* Variation in Rm as T varies when NT=2 *)
InvFitAcrossWT =
  Table[NumSolInvFit[200, T, 0.9, 0.9, 2, 0.01, 0.1, 0.01], {T, 270, 310, 2}];
```

```
Labeled[ListLinePlot[
  Table[{Table[T, {T, 270, 310, 2}][[i]], InvFitAcrossWT[[i]]}, {i, 1, 21}],
  PlotLabel → "Fig. S13. Effect of T on Rm when NT is large"],
 {"Temperature T", "Generalist Rm"}, {Bottom, Left}, RotateLabel → True]
```

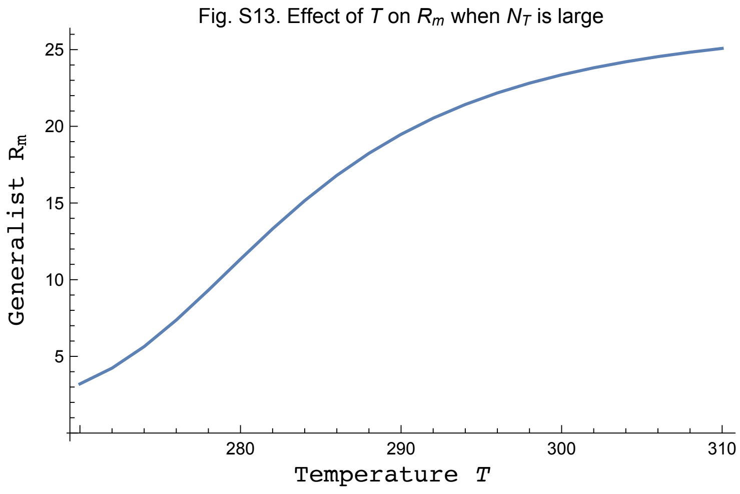


```
(* Variation in Rm as T varies when NT=0.1 *)
InvFitAcrossWT =
  Table[NumSolInvFit[200, T, 0.9, 0.9, 0.1, 0.01, 0.1, 0.01], {T, 270, 310, 2}];
```

```
Labeled[ListLinePlot[
  Table[{Table[T, {T, 270, 310, 2}][[i]], InvFitAcrossWT[[i]]}, {i, 1, 21}],
  PlotLabel → "Fig. S14. Effect of T on Rm when NT is small"],
 {"Temperature T", "Generalist Rm"}, {Bottom, Left}, RotateLabel → True]
```

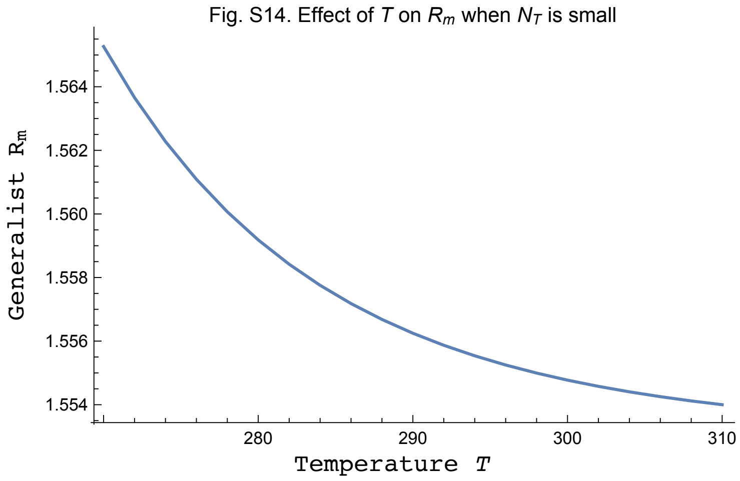

Supplement: Appendix B: Trophic transmission model analyses [file rstb20160089supp2.pdf]
