# Supplementary material for: Host allometry influences the evolution of parasite host-generalism: theory and meta-analysis
Source: Philos Trans R Soc Lond B Biol Sci. 2017 Mar 13;372(1719):20160089. doi: 10.1098/rstb.2016.0089 (PMC5352816; doi:10.1098/rstb.2016.0089)
Supplement: Appendix C: Supplementary Material [file rstb20160089supp3.doc]

**Appendix C**

**Host genetic distances**

Methods

Host mitochondrial DNA sequences (complete mitochondrial genomes and full or partial sequences from mitochondrial loci) were downloaded in fasta format from the NCBI nucleotide database using an Entrez Direct query. Sequences were discarded if the sequence header did not contain the species name (either full or abbreviated scientific name). They were then sorted by locus (limited to cytochrome oxidase 1, cytochrome B, 12s and 16s) based on regular expression matches to the sequence header and the assignment to loci checked by local BLASTN [1] searches (default settings, version 2.2.29) to databases of representative sequences from the relevant locus. When multiple sequences were available for a given locus and host, a consensus sequence was generated using the EMBOSS program *cons* [2]*.* The consensus sequence was used in downstream analyses except in cases when just two sequences had contributed to the consensus and the result contained more than 1% of variable positions. In such cases the first sequence was used instead of the consensus if a megaBLAST [1] search against the NCBI nucleotide database hit a member of the same genus with percentage identity greater than 90%, and the second sequence used otherwise. For hosts without sequence data for a particular locus, sequences were extracted from mitochondrial complete genomes when available using BLASTN. Consensus sequences for all available host species were combined and aligned - adjusting for direction - using MAFFT for each locus. Alignments were then trimmed with trimAl [3] to include only those columns where less than 50% of taxa had a gap and those taxa where 50% of the nucleotide positions had the same ‘element’ (e.g. a gap or a residue) as more than half of the other taxa in the alignment.

Trimmed alignments were used to compute pairwise genetic distances using the dist.dna function and the K80 model of DNA evolution [4] in the R package *ape* [5]. Since different taxa were represented among loci, the consistency of pairwise distances among loci across a range of divergence times was assessed. The pairwise genetic distances between those host taxa with sequences for all loci were extracted. For each locus these pairwise genetic distances were plotted against their corresponding pairwise distance generated from cytochrome oxidase 1 sequences. The point patterns were compared to the one-to-one line (complete correspondence), and correspondence to this line used to select the loci to be concatenated using *ape*. Pairwise genetic distances were recalculated on this final concatenated alignment. Missing pairs were imputed using a custom R script by averaging according to the following relationships between taxa: 1) pairs from different genera – the mean of the genetic distances of one member of the pair (determined by the data available) to congeners of the remaining pair member was calculated, 2) pairs from the same genera – the mean genetic distance of all the other pairwise comparisons within that genus was calculated. Where no data were available for any member of a genus, the mean within genus average for all genera was used. If suitable data were unavailable the same principals were applied at increasing taxonomic levels (family, order, class) until values were obtained for all pairwise comparisons.

A UPGMA tree was calculated from the full distance matrix using *phangorn* in R [6] and the tree reordered from root to tip so that edges from the root node were listed first (‘cladewise’ reordering).

The total number of host species in our dataset was 4,621, but sequence data from a single gene were not available for all hosts. The pairwise genetic distances calculated using data from the cytochrome oxidase 1 gene corresponded to those from cytochrome B (Fig. S1) and these loci were selected for concatenation and subsequent calculation of pairwise genetic distances. Overall, 3,253 host species were represented by cytochrome oxidase 1 sequences and 2,193 host species by cytochrome B, 1,915 host species had sequences from both loci whilst a total of 3,531 host species had representative sequences from one or both loci. The mean pairwise genetic distance between hosts was 0.263 (standard deviation=0.034; Fig. S2).

**Supplementary Figure 1:** Comparison of pairwise genetic distances between hosts generated from cytochrome oxidase 1 (CO1) sequences and cytochrome B (cytB) sequences plotted against one to one line.

**Supplementary Figure 2:** Distribution of genetic distances from all pairwise comparisons of hosts.


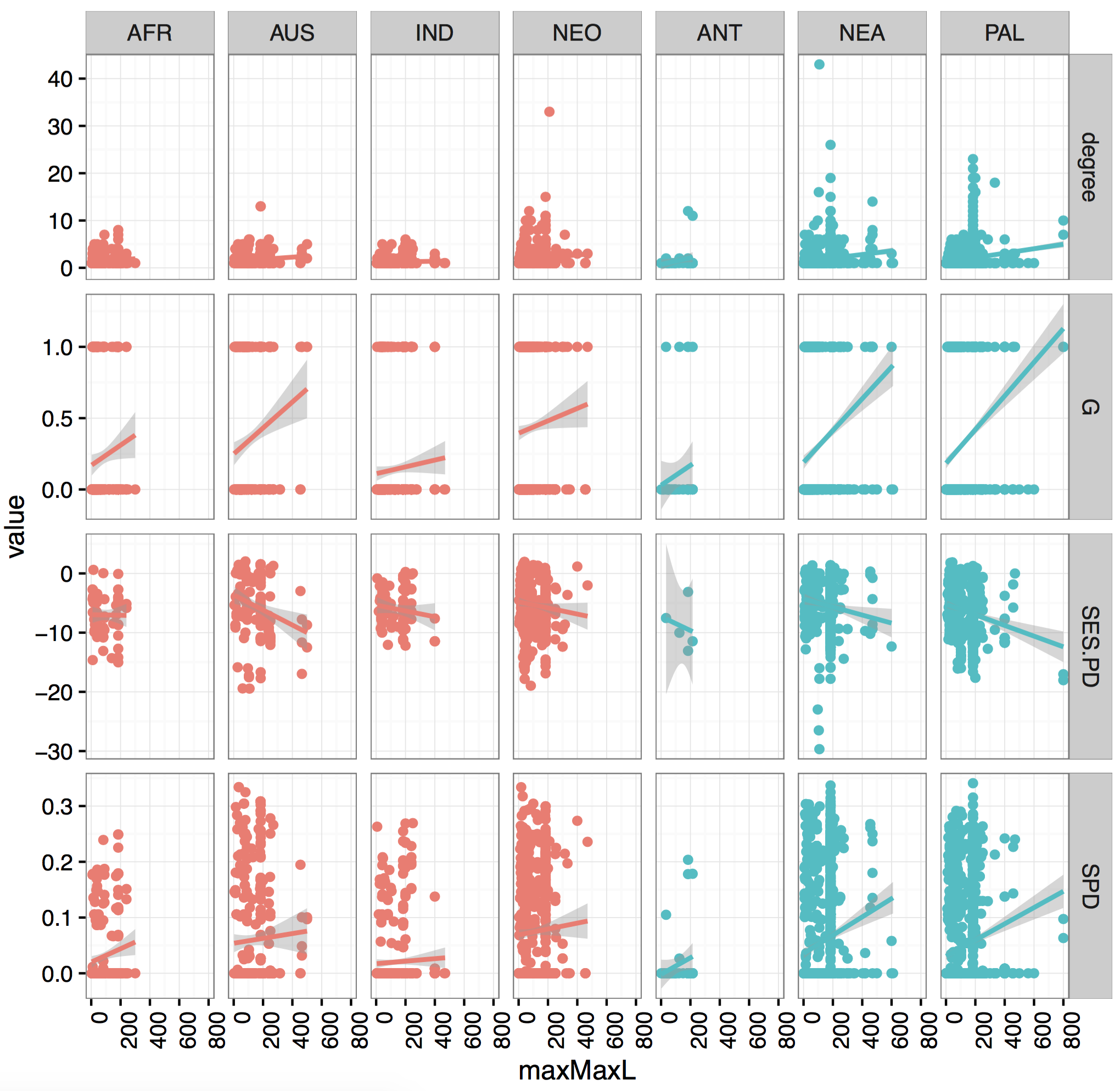


**Supplementary Figure 3:** Correlation between generalism metrics and maximum host length for directly transmitted parasites in each geographic region, with warm regions in red and cool regions in blue. Lines are linear models with grey 95% confidence bounds.


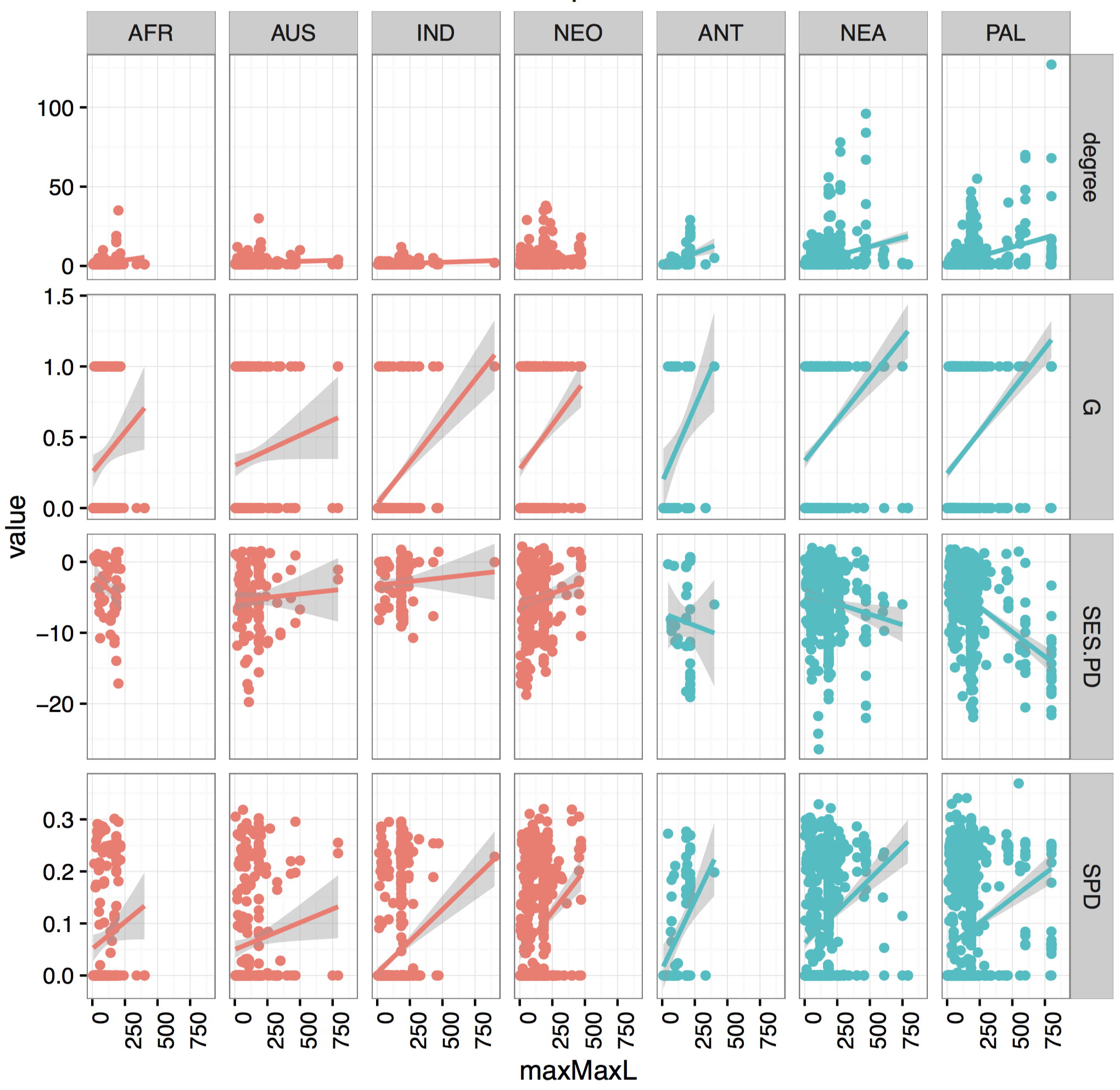


**Supplementary Figure 4:** Correlation between generalism metrics and maximum host length for trophically transmitted parasites in each geographic region, with warm regions in red and cool regions in blue. Lines are linear models with grey 95% confidence bounds.

**Supplementary Table 1:** Results of multivariate negative binomial regression (degree ~ Length * Transmission * GeoGroup), with each of three measures of length (mean, max, CV) separately. For mean and max length the scale of the coefficient is unit change in log degree per SD increase in length measure, for CV length the scale is unit change in log degree per cm increase in CV.

| Predictor | Coefficient | Confidence Interval | | Z-score | Residual degrees of freedom |
| --- | --- | --- | --- | --- | --- |
| Intercept | 0.462 | 0.415 | 0.509 | 19.314 | 8595 |
| Mean length | -0.102 | -0.152 | -0.052 | -4.011 |
| Trophic transmission (ref = direct) | 0.239 | 0.171 | 0.306 | 6.946 |
| Geo group cool (ref = warm) | 0.106 | 0.045 | 0.167 | 3.414 |
| Length*trophic | -0.068 | -0.141 | 0.004 | -1.868 |
| Length*cool | 0.092 | 0.028 | 0.158 | 2.848 |
| Trophic*cool | 0.576 | 0.49 | 0.663 | 13.098 |
| Length*trophic*cool | -0.066 | -0.16 | 0.029 | -1.416 |
| Intercept | 0.493 | 0.448 | 0.538 | 21.52 | 8595 |
| Max length | 0.086 | 0.034 | 0.139 | 3.276 |
| Trophic transmission (ref = direct) | 0.208 | 0.143 | 0.272 | 6.321 |
| Geo group cool (ref = warm) | 0.055 | -0.003 | 0.114 | 1.855 |
| Length*trophic | 0.178 | 0.106 | 0.249 | 5.154 |
| Length*cool | 0.154 | 0.087 | 0.221 | 4.632 |
| Trophic*cool | 0.355 | 0.271 | 0.439 | 8.318 |
| Length*trophic*cool | 0.065 | -0.025 | 0.154 | 1.531 |
| Intercept | 0.971 | 0.855 | 1.087 | 16.714 | 3028 |
| CV length | 0.192 | 0.02 | 0.365 | 2.25 |
| Trophic transmission (ref = direct) | -0.025 | -0.201 | 0.151 | -0.29 |
| Geo group cool (ref = warm) | 0.085 | -0.059 | 0.23 | 1.178 |
| Length*trophic | 0.638 | 0.384 | 0.893 | 5.1 |
| Length*cool | 0.118 | -0.102 | 0.338 | 1.083 |
| Trophic*cool | 0.126 | -0.096 | 0.349 | 1.153 |
| Length*trophic*cool | 0.322 | -0.001 | 0.645 | 2.065 |

**Supplementary Table 2:** Results of multivariate logistic regression (G ~ Length * Transmission * GeoGroup), with each of two measures of length (mean, max) separately. For both length measures the scale of the coefficient is unit change in log odds ratio per SD increase in length measure.

| Predictor | Coefficient | Confidence Interval | | Z-score | Residual degrees of freedom |
| --- | --- | --- | --- | --- | --- |
| Intercept | -0.826 | -0.925 | -0.73 | -16.625 | 8595 |
| Mean length | -0.329 | -0.438 | -0.222 | -5.976 |
| Trophic transmission (ref = direct) | 0.048 | -0.095 | 0.19 | 0.657 |
| Geo group cool (ref = warm) | 0.092 | -0.033 | 0.218 | 1.44 |
| Length*trophic | 0.146 | -0.009 | 0.301 | 1.856 |
| Length*cool | 0.34 | 0.206 | 0.476 | 4.935 |
| Trophic*cool | 0.507 | 0.322 | 0.691 | 5.382 |
| Length*trophic*cool | -0.255 | -0.45 | -0.06 | -2.57 |
| Intercept | -0.742 | -0.837 | -0.648 | -15.352 | 8595 |
| Max length | 0.148 | 0.039 | 0.258 | 2.656 |
| Trophic transmission (ref = direct) | -0.028 | -0.169 | 0.114 | -0.382 |
| Geo group cool (ref = warm) | -0.017 | -0.142 | 0.109 | -0.263 |
| Length*trophic | 0.311 | 0.154 | 0.47 | 3.856 |
| Length*cool | 0.402 | 0.256 | 0.549 | 5.374 |
| Trophic*cool | 0.485 | 0.298 | 0.672 | 5.074 |
| Length*trophic*cool | -0.193 | -0.408 | 0.021 | -1.771 |

**Supplementary Table 3:** Results of multivariate linear regression (SPD~ Length * Transmission * GeoGroup), with each of three measures of length (mean, max, CV) separately. For mean and max length the scale of the coefficient is unit change in SPD per SD increase in length measure, for CV length the scale is unit change in SPD per cm increase in CV.

| Predictor | Coefficient | Confidence Interval | | Z-score | Residual degrees of freedom |
| --- | --- | --- | --- | --- | --- |
| Intercept | 0.049 | 0.045 | 0.054 | 23.781 | 8595 |
| Mean length | -0.014 | -0.019 | -0.01 | -6.679 |
| Trophic transmission (ref = direct) | 0.01 | 0.004 | 0.017 | 3.388 |
| Geo group cool (ref = warm) | 0.002 | -0.004 | 0.007 | 0.637 |
| Length*trophic | 0.008 | 0.002 | 0.014 | 2.454 |
| Length*cool | 0.011 | 0.006 | 0.017 | 4.035 |
| Trophic*cool | 0.028 | 0.02 | 0.036 | 6.74 |
| Length*trophic*cool | -0.015 | -0.023 | -0.007 | -3.644 |
| Intercept | 0.052 | 0.047 | 0.056 | 24.726 | 8595 |
| Max length | 0.002 | -0.003 | 0.007 | 0.776 |
| Trophic transmission (ref = direct) | 0.01 | 0.004 | 0.016 | 3.129 |
| Geo group cool (ref = warm) | 0 | -0.006 | 0.005 | -0.127 |
| Length*trophic | 0.021 | 0.014 | 0.028 | 6.179 |
| Length*cool | 0.014 | 0.008 | 0.02 | 4.498 |
| Trophic*cool | 0.023 | 0.015 | 0.031 | 5.669 |
| Length*trophic*cool | -0.015 | -0.023 | -0.007 | -3.561 |
| Intercept | 0.126 | 0.117 | 0.135 | 26.634 | 3028 |
| CV length | 0.065 | 0.051 | 0.079 | 9.188 |
| Trophic transmission (ref = direct) | 0.029 | 0.015 | 0.043 | 3.967 |
| Geo group cool (ref = warm) | 0.017 | 0.006 | 0.029 | 2.933 |
| Length*trophic | -0.005 | -0.026 | 0.017 | -0.438 |
| Length*cool | -0.036 | -0.054 | -0.018 | -3.941 |
| Trophic*cool | -0.014 | -0.032 | 0.005 | -1.469 |
| Length*trophic*cool | 0.035 | 0.008 | 0.062 | 2.539 |

**Supplementary Table 4:** Results of multivariate linear regression (SES.PD ~ Length * Transmission * GeoGroup), with each of three measures of length (mean, max, CV) separately. For mean and max length the scale of the coefficient is unit change in SES.PD per SD increase in length measure, for CV length the scale is unit change in SES.PD per cm increase in CV.

| Predictor | Coefficient | Confidence Interval | | Z-score | Residual degrees of freedom |
| --- | --- | --- | --- | --- | --- |
| Intercept | -5.816 | -6.194 | -5.439 | -30.226 | 3028 |
| Mean length | -0.72 | -1.158 | -0.282 | -3.221 |
| Trophic transmission (ref = direct) | 1.336 | 0.788 | 1.883 | 4.785 |
| Geo group cool (ref = warm) | -0.156 | -0.637 | 0.326 | -0.634 |
| Length*trophic | 1.412 | 0.752 | 2.071 | 4.197 |
| Length*cool | 0.039 | -0.542 | 0.62 | 0.133 |
| Trophic*cool | -1.033 | -1.721 | -0.345 | -2.943 |
| Length*trophic*cool | -1.94 | -2.789 | -1.091 | -4.478 |
| Intercept | -5.674 | -6.025 | -5.324 | -31.733 | 3028 |
| Max length | -0.693 | -1.1 | -0.286 | -3.338 |
| Trophic transmission (ref = direct) | 0.994 | 0.466 | 1.522 | 3.692 |
| Geo group cool (ref = warm) | -0.133 | -0.6 | 0.334 | -0.556 |
| Length*trophic | 1.08 | 0.532 | 1.628 | 3.864 |
| Length*cool | -0.096 | -0.629 | 0.437 | -0.354 |
| Trophic*cool | -0.126 | -0.814 | 0.562 | -0.36 |
| Length*trophic*cool | -1.48 | -2.162 | -0.799 | -4.261 |
| Intercept | -7.007 | -7.617 | -6.398 | -22.547 | 3028 |
| CV length | 2.6 | 1.687 | 3.512 | 5.586 |
| Trophic transmission (ref = direct) | 1.662 | 0.72 | 2.604 | 3.458 |
| Geo group cool (ref = warm) | 0.722 | -0.044 | 1.488 | 1.848 |
| Length*trophic | -1.267 | -2.663 | 0.13 | -1.779 |
| Length*cool | -2.034 | -3.215 | -0.853 | -3.376 |
| Trophic*cool | -1.306 | -2.502 | -0.111 | -2.142 |
| Length*trophic*cool | 1.118 | -0.655 | 2.892 | 1.236 |

**References**

1. McGinnis, S. & Madden, T. L. 2004 BLAST: at the core of a powerful and diverse set of sequence analysis tools. *Nucleic Acids Res.* **32**, W20–W25. (doi:10.1093/nar/gkh435)

2. Rice, P., Longden, I. & Bleasby, A. 2000 EMBOSS: The European Molecular Biology Open Software Suite. *Trends Genet.* **16**, 276–277. (doi:10.1016/S0168-9525(00)02024-2)

3. Capella-Gutiérrez, S., Silla-Martínez, J. M. & Gabaldón, T. 2009 trimAl: a tool for automated alignment trimming in large-scale phylogenetic analyses. *Bioinformatics* **25**, 1972–1973. (doi:10.1093/bioinformatics/btp348)

4. Kimura, M. 1980 A simple method for estimating evolutionary rates of base substitutions through comparative studies of nucleotide sequences. *J. Mol. Evol.* **16**, 111–120.

5. Paradis, E., Claude, J. & Strimmer, K. 2004 APE: Analyses of phylogenetics and evolution in R language. *Bioinformatics* **20**, 289–290. (doi:10.1093/bioinformatics/btg412)

6. Schliep, K. P. 2011 phangorn: phylogenetic analysis in R. *Bioinformatics* **27**, 592–593. (doi:10.1093/bioinformatics/btq706)
